# Research Activity and Subspecialty Imbalance of Artificial Intelligence in Orthopaedics: A Bibliometric Analysis

**DOI:** 10.3390/healthcare14193183

**Published:** 2026-09-25

**Authors:** Nezih Ziroglu, Samet Engin Ucar, Berk Celik, Ali Can Koluman, Bertan Cem Yavasoglu

**Affiliations:** 1 Department of Orthopedic Prosthetics and Orthotics, Vocational School of Health Services, Acibadem Mehmet Ali Aydinlar University, 34638 Istanbul, Turkey; 2 Department of Orthopaedics and Traumatology, Acibadem University Atakent Hospital, 34303 Istanbul, Turkey; 3 Acibadem University School of Medicine, 34752 Istanbul, Turkey; 4 Department of Orthopaedics and Traumatology, Bakirkoy Dr. Sadi Konuk Training and Research Hospital, 34147 Istanbul, Turkey; 5 Department of Orthopaedics and Traumatology, Medipol Mega University Hospital, 34214 Istanbul, Turkey

**Keywords:** artificial intelligence, orthopaedics, bibliometric analysis, machine learning, deep learning, orthopaedic subspecialties, research trends, translational research, spine surgery, arthroplasty

## Abstract

**Background**: Artificial intelligence (AI) has rapidly emerged as a major research domain in orthopaedics, driven by advances in computational methods and the widespread availability of digital clinical data. Although AI-related orthopaedic publications have increased substantially, the extent to which this growth reflects a balanced distribution of research activity across clinical subspecialties and AI modalities remains unclear. **Purpose:** The aim of this study was to evaluate temporal trends, subspecialty distribution, and methodological characteristics of AI-related orthopaedic research activity using a bibliometric approach. **Methods:** A bibliometric analysis was performed using publications indexed in two major scientific databases between 2016 and 2025. AI-related orthopaedic articles were identified using predefined search terms. Publications were analyzed according to annual publication trends, orthopaedic subspecialty classification, AI methodology, and proxy indicators of study design and evidence development. Subspecialty assignment was performed using a standardized taxonomy, and descriptive analyses were used to characterize research patterns. **Results:** AI-related orthopaedic publications increased exponentially during the study period. Research output was unevenly distributed across subspecialties, with adult reconstruction and spine surgery accounting for the largest share of publications, whereas foot and ankle surgery, pediatric orthopaedics, hand & wrist, and shoulder & elbow surgery remained underrepresented. Deep learning–based imaging studies predominated, while studies incorporating structured clinical data, longitudinal outcomes, and external validation were comparatively limited. Much of the literature remains exploratory, primarily focusing on early-phase model development. **Conclusions:** AI research in orthopaedics has expanded rapidly but remains methodologically heterogeneous, with research activity heavily concentrated in data-rich subspecialties such as adult reconstruction and spine surgery. The predominance of early-phase studies indicates that current publication volumes are largely driven by data accessibility rather than a balanced distribution across all orthopaedic domains. Future research should encourage multicenter collaboration, standardized reporting, and targeted studies in underrepresented subspecialties to ensure a more balanced development of orthopaedic AI tools.

## 1. Introduction

Artificial intelligence (AI) has become an increasingly influential force in medical research over the past decade, driven by advances in machine learning (ML) and deep learning (DL), together with rapid growth in computational power and data availability [1,2,3]. In orthopaedics, these developments have coincided with widespread digitization of clinical workflows, routine use of advanced imaging modalities, and the availability of large-scale musculoskeletal datasets [4,5]. As a result, AI-related research in orthopaedics has expanded substantially, progressing from early exploratory studies to a broad literature encompassing diagnostic support, treatment planning, outcome prediction, and postoperative or rehabilitative assessment [6,7].

Despite this growth, the development of AI research in orthopaedics has been largely fragmented. Rather than evolving as a cohesive field, studies have tended to emerge within specific clinical and anatomical domains, often reflecting the subspecialty-based structure of orthopaedic practice [6,7,8]. This fragmentation has complicated efforts to gain an overarching understanding of how AI research has evolved within orthopaedics, which subspecialties have driven research activity, and how thematic priorities have shifted over time [8,9]. Recent bibliometric analyses published in early 2026 have provided valuable macro-level insights into global research trends, collaboration networks, and journal productivity in orthopaedic AI [10,11,12]. However, these excellent foundational studies have predominantly analyzed orthopaedics as a single aggregated domain. A granular, cross-sectional evaluation mapping specific AI modalities against distinct clinical subspecialties remains a critical gap in the literature. Consequently, there is a growing need for analytical approaches that can synthesize this expanding literature and clarify its structure and progression. Bibliometric analysis provides a systematic and reproducible method for quantitatively assessing publication trends, citation patterns, and thematic relationships within large bodies of scientific work [13].

Orthopaedic research and clinical practice are inherently structured according to subspecialties formally defined by the American Academy of Orthopaedic Surgeons (AAOS), including shoulder and elbow, knee, hip, foot and ankle, hand and wrist, spine, pediatric orthopaedics, trauma, sports orthopaedics, and orthopaedic oncology. However, most bibliometric studies to date have examined artificial intelligence in orthopaedics as a single aggregated research domain, without systematically evaluating differences across these clinically relevant subspecialties [9,13]. Given the substantial variation among these areas in data standardization, imaging characteristics, and digital workflows, a subspecialty-oriented bibliometric perspective is essential to accurately interpret the distribution of AI within orthopaedic research. Therefore, the purpose of this study is to provide a comprehensive bibliometric analysis of AI-related publications in orthopaedics over the past decade to characterize publication trends, thematic evolution, and subspecialty-specific differences in research activity.

## 2. Materials and Methods

### 2.1. Study Design, Data Sources, and Temporal Scope

This study was conceptualized as a systematic bibliometric analysis and mapping review. To ensure comprehensive coverage of high-impact literature, data were retrieved from two primary indices: Web of Science Core Collection (WoS) (Clarivate Analytics, Philadelphia, PA, USA; (https://www.webofscience.com, accessed on 5 January 2026) and Scopus (Elsevier, Amsterdam, The Netherlands, https://www.scopus.com/pages/home, accessed on 5 January 2026). These databases were selected for their rigorous content curation and widely used citation databases compared to non-indexed search engines [14]. The analysis encompassed a ten-year longitudinal period from 1 January 2016 to 31 December 2025. To ensure temporal consistency of citation metrics, all data extraction was performed on 5 January 2026.

### 2.2. Structured Search Strategy and Syntax Construction

The search strategy was constructed in strict adherence to the PRESS 2015 (Peer Review of Electronic Search Strategies) guideline recommendations [15]. Specifically, the electronic search strings, including Boolean operators and field tags, were formulated and subsequently peer-reviewed by an independent co-author with expertise in systematic search strategies prior to final execution to optimize recall and precision ratios. Detailed Boolean query strings were developed to capture the intersection of two distinct domains:

- Artificial Intelligence: Including terms such as “machine learning”, “deep learning”, “neural networks”, and “natural language processing”.
- Orthopaedics: Including terms such as “orthopaedic surgery”, “musculoskeletal system”, “arthroplasty”, “spine surgery”. The exact syntax, including field tags and Boolean operators for each database, is provided in Supplementary Table S1.

### 2.3. Algorithmic Data Processing and Structured Exclusion Protocol

Raw bibliographic records were exported in RIS format and consolidated into a unified dataset. To mitigate the risk of human error inherent in manual curation of large-scale datasets, a custom Python-based automated workflow (Python Software Foundation, Wilmington, DE, USA; https://www.python.org, accessed on 14 January 2026) was developed using the Pandas library (v2.0.3) (NumFOCUS, Austin, TX, USA) for data manipulation [16] (Supplementary File S1). The screening workflow was adapted from PRISMA 2020 reporting principles [17] and involved three distinct stages of filtration:

#### 2.3.1. Deterministic Deduplication

- Primary Merge: Records were initially merged based on unique Digital Object Identifiers (DOI).
- Secondary Normalization: For records lacking a DOI, a string normalization algorithm was applied (converting titles to lowercase, stripping punctuation/whitespaces) to identify and merge duplicates based on Title and Publication Year.

#### 2.3.2. Data Quality and Publication Type Control

To ensure the analysis focused on peer-reviewed original literature, non-original publication types including editorials, letters to the editor, commentaries, notes, and short surveys were algorithmically excluded. Conference proceedings and book chapters were removed to restrict the cohort to peer-reviewed journal articles.

- Data Integrity Check: A rigorous screening for data integrity identified five retracted publications, which were flagged and permanently removed from the dataset to ensure the reliability of the bibliometric analysis.

#### 2.3.3. Content and Language Filtering

- Language: The dataset was restricted to articles published in the English language to ensure accurate semantic processing and comparability.
- Relevance Assessment: Relevance was determined through an automated algorithmic screening of titles, abstracts, and keywords. Because this study utilizes a macro-level bibliometric design intended to capture broad thematic and subspecialty trends across thousands of records, manual full-text screening was not conducted. Instead, automated semantic rules were applied to exclude tangential studies. For instance, based on the Centre for Evidence-Based Medicine (CEBM) criteria for therapeutic studies [18], studies focusing on veterinary medicine and pure animal models were excluded by algorithmically filtering out specific keywords (e.g., equine, bovine, canine, porcine, veterinary).
- The final study corpus comprised 4970 original peer-reviewed articles (Figure 1).

#### 2.3.4. Standardization of Clinical Subspecialties (AAOS Taxonomy)

Included studies were categorized into 9 distinct sub-specialties, strictly adopting the organizational taxonomy of the AAOS used for fellowship accreditation [19].

To ensure terminological validity and eliminate subjective classification bias, keyword dictionaries for each sub-field were derived from two standardized ontologies:

- MeSH (Medical Subject Headings) descriptors maintained by the National Library of Medicine [20].
- ICD-11 (International Classification of Diseases) codes for musculoskeletal disorders [21].

Before full-scale analysis, the classification algorithms underwent a pilot validation phase on a random sample of 50 articles, achieving high concordance with manual review. The specific MeSH Unique IDs and structured search strings are detailed in Supplementary Table S2.

Since clinical scenarios in orthopaedics often overlap (e.g., a trauma case managed with an arthroplasty approach), the subspecialty assignment categories were not mutually exclusive. A single article containing relevant keywords from multiple domains could be assigned to more than one subspecialty. Consequently, the cumulative count across subspecialties exceeds the total number of unique articles (*n* = 4970) in the corpus.

To validate the classification algorithms, a pilot phase was conducted on a purely random sample of 50 articles. The manually adjudicated gold standard was established by two independent reviewers blinded to the algorithm’s output. The inter-rater agreement achieved a Cohen’s kappa of 0.88, indicating substantial concordance. Category-specific performance, including positive/negative examples and confusion matrices (TP, TN, FP, FN), as well as sensitivity and specificity—defined as TP/(TP + FN) and TN/(TN + FP), respectively—are detailed in Supplementary Table S3. The reported overall metrics (accuracy: 92.0%, sensitivity: 90.5%, specificity: 93.1%) were calculated using a macro-averaged approach across all categories to account for class imbalances. However, due to the random sampling method, less frequent subspecialties may have contained limited positive examples, precluding reliable category-specific confidence intervals.

### 2.4. Multi-Dimensional Classification: Technologies and Objectives

A multi-dimensional text-mining approach was implemented to stratify each article across technological and clinical axes:

- AI Taxonomy (Technological Dimension): To capture the inherent overlap in modern computational methods, AI modalities were treated as non-mutually exclusive labels (multi-label classification). The categories were aligned with the Association for Computing Machinery (ACM) Computing Classification System (CCS) hierarchy [22] and foundational definitions in the field [23], comprising Machine Learning (ML), Deep Learning (DL), Natural Language Processing (NLP), and Robotics. It is conceptually recognized that DL is a subset of ML, and NLP frequently employs ML/DL algorithms. However, DL was analyzed as a distinct subset to isolate the specific growth trajectory of deep neural architectures. Furthermore, the robotics category was strictly limited to systems incorporating autonomous, algorithmic, or AI-driven control, explicitly excluding purely mechanical or manual navigation systems. Therefore, a single article could simultaneously receive multiple technological tags (e.g., both DL and NLP).
- Clinical Objective (Methodological Dimension): Rather than relying on reporting guidelines, the clinical objectives were derived empirically based on the most prevalent application domains identified in the corpus. Studies were stratified into four primary functional categories: Diagnosis & Detection, Prognosis & Prediction, Segmentation & Measurement, and Surgical Planning. A representative dataset extract demonstrating these study-level classifications alongside persistent identifiers is provided in Supplementary Table S4.

### 2.5. Bibliometric Indicators and Statistical Analysis

To unveil the intellectual architecture, thematic sub-domains, and collaboration dynamics of artificial intelligence research in orthopaedics, advanced science mapping was performed using VOSviewer software (v1.6.20, Leiden University, Leiden, The Netherlands)). A keyword co-occurrence analysis was conducted based on the most frequent terms extracted from titles, abstracts, and keywords to identify primary thematic clusters. Furthermore, a co-authorship network analysis was executed based on authors to identify the core research groups, prolific investigators, and academic collaborations shaping the international orthopaedic AI landscape. Descriptive statistics and growth trend analyses were computed using Python (v3.11) (Python Software Foundation, Wilmington, DE, USA).

- Geographic Standardization: Author affiliations were computationally parsed and standardized according to the ISO 3166-1 international standard to resolve variations in country names (e.g., mapping “UK” and “Great Britain” to “United Kingdom”) [24]. To prevent double counting of internationally co-authored publications and maintain a total count equal to the corpus size (*n* = 4970), country attribution was based exclusively on the primary institutional affiliation of the corresponding author.
- Growth Kinetics: The annual growth rate of publications was quantified using the Compound Annual Growth Rate (CAGR) formula [25] over *t* = 9 annual intervals (from 2016 to 2025):

$$CAGR={\left(\frac{V_{final}}{V_{initial}}\right)}^{\frac{1}{t}}-1$$

where $V_{final}$ = 1502 and $V_{initial}$ = 21. The exact unrounded calculation yields (1502/21)^1/9^ − 1 = 0.60499…, establishing the reported approximate *CAGR* of 60.4%.

- Network Analysis Parameters: Science mapping and network visualizations were conducted using VOSviewer software (v1.6.20). For the keyword co-occurrence analysis, a “full counting” method was applied with a minimum occurrence threshold of 20, resulting in 170 nodes. Node clustering and spatial distribution were calculated utilizing the software’s default “Association Strength” normalization. To ensure the network accurately reflected the intellectual and clinical structure of the field, a custom VOSviewer thesaurus file was applied. This procedure performed synonym cleaning (e.g., merging “artificial intelligence” and “ai”) and explicitly excluded uninformative generic indexing terms (e.g., “human,” “article,” “male,” “female,” “adult,” “retrospective study”). For the co-authorship network, a “fractional counting” approach was utilized to mitigate the disproportionate impact of hyper-authored publications, and author disambiguation was performed manually by cross-referencing institutional affiliations.

## 3. Results

### 3.1. Temporal Evolution and Growth Trajectory

A total of 4970 original peer-reviewed articles were included in the final analysis following the rigorous exclusion of duplicate records, non-peer-reviewed content, and retracted publications as detailed in the PRISMA workflow (Figure 1). The bibliometric analysis revealed a definitive rapid growth in the application of artificial intelligence within orthopaedics over the studied decade (2016–2025).

The annual publication volume surged from 21 articles in 2016 to 1502 articles in 2025, representing a Compound Annual Growth Rate (CAGR) of 60.4%. Notably, the accumulation of literature accelerated significantly in the post-pandemic era; 52.6% of the entire corpus (*n* = 2615) was published in the last two years (2024 and 2025) alone. This steep upward trend indicates that AI integration in orthopaedics has transitioned from an experimental phase to a widespread clinical research priority (Table 1, Figure 2).

### 3.2. Geographic Distribution and Global Productivity

Geospatial analysis of author affiliations identified 86 distinct countries contributing to the field. The research landscape was heavily polarized, with two nations driving the global output: the United States (*n* = 1606) and China (*n* = 1006). Together, these two countries accounted for 52.5% of the total global literature.

They were followed by Germany (*n* = 222), South Korea (*n* = 202), and Canada (*n* = 161), forming a secondary cluster of highly productive research hubs. The remaining 23.5% of the output was distributed across 76 other nations, indicating a concentration of AI resources in high-income economies (Table 2, Figure 3).

### 3.3. Journal Analysis and Publication Venues

The dataset spanned a diverse array of publication venues, ranging from specialized orthopaedic journals to technical computer science periodicals. However, the publication distribution across these venues exhibited a highly concentrated core-scatter pattern, indicating that a small fraction of specialized journals published the vast majority of the relevant literature.

The European Spine Journal (*n* = 107) and the International Journal of Computer Assisted Radiology and Surgery (*n* = 104) emerged as the leading venues, reflecting the interdisciplinary nature of the field. High-impact subspecialty journals such as *Osteoarthritis and Cartilage* (*n* = 101) and *The Journal of Arthroplasty* (*n* = 87) also demonstrated high receptivity to AI-focused research. The top 10 journals accounted for 17.1% of the total output (*n* = 852) (Table 3).

### 3.4. Analysis by Clinical Subspecialties (AAOS Taxonomy)

When stratified according to the AAOS taxonomy defined in the methodology, the distribution of AI research was heterogeneous across sub-specialties (Table 4).

- Adult Reconstruction (Arthroplasty) was the most dominant sub-field, featuring in 3491 articles. This prevalence is largely driven by the high volume of radiographic data available for image-based deep learning models in hip and knee replacements.
- Spine Surgery ranked second (*n* = 2292), with a heavy focus on predictive modeling for surgical outcomes and automated segmentation of vertebrae.
- Trauma (*n* = 1495) and Sports Medicine (*n* = 831) followed, while niche fields such as Foot & Ankle (*n* = 250) remained relatively underrepresented compared to major joint surgery.

### 3.5. Distribution of AI Modalities (ACM Taxonomy)

In accordance with the ACM Computing Classification System, the technological landscape was analyzed to determine the specific AI modalities employed (Table 4).

- Deep Learning (DL) was the most utilized methodology, identified in 2286 articles. Its dominance is attributed to the widespread use of Convolutional Neural Networks (CNNs) for medical image analysis (X-ray, magnetic resonance imaging (MRI), and computed tomography (CT).
- Machine Learning (ML) (traditional algorithms) remained a core technology, utilized in 2110 articles, primarily for prognostic modeling using tabular clinical data.
- NLP showed increasing representation (*n* = 672), in the later years of the study period, driven by the adoption of Large Language Models (LLMs) for analyzing electronic health records.
- Robotics and Navigation (*n* = 259) represented a specialized segment focused on intraoperative assistance.

### 3.6. Intersection of Clinical Domains and AI Modalities

A cross-tabulation analysis (Figure 4) revealed distinct technological preferences within clinical sub-specialties.

- Arthroplasty exhibited the strongest affinity for Deep Learning (DL), which was the primary modality in 46.1% of arthroplasty-related studies. This correlation aligns with the heavy reliance on radiographic image analysis for implant sizing and loosening detection.
- In contrast, Spine Surgery and Trauma demonstrated a more balanced distribution between DL (image segmentation) and traditional Machine Learning (ML) models (prognostic tabular data analysis).
- NLP was ubiquitously distributed but showed a specific concentration in Spine Surgery, primarily applied to the automated extraction of complications and patient-reported outcomes from clinical notes.
- Robotics was almost exclusively concentrated in Arthroplasty (knee/hip navigation) and Spine Surgery (screw placement), with minimal application in other sub-fields.

### 3.7. Thematic Networks and Co-Authorship Landscapes

After applying the minimum occurrence threshold and utilizing a custom thesaurus to exclude uninformative generic terms (such as ‘human’, ‘male’, and ‘adult’) and consolidate synonyms, the final keyword co-occurrence network consisted of 170 nodes (items) distributed across 5 thematic clusters. The network demonstrated high interconnectivity, featuring 14,345 links with a total link strength of 258,836. The keyword co-occurrence network generated via VOSviewer revealed 5 distinct, highly interconnected thematic clusters that define the research landscape (Figure 5):

- The Green Cluster (Deep Learning & Diagnostic Imaging): Focuses heavily on complex computational architectures and radiology. Key nodes include “deep learning,” “convolutional neural network,” and “artificial neural network,” which are densely connected to “diagnostic imaging,” “radiography,” “image processing,” and specific targets like “fracture” and “bone.”
- The Red Cluster (Predictive Analytics & Clinical Outcomes): This largest cluster centers around “machine learning,” “artificial intelligence,” and “predictive model.” It is strongly anchored to clinical applications and risk factor analysis, featuring prominent nodes such as “total knee arthroplasty,” “hip replacement,” “osteoarthritis,” and “spine surgery.”
- The Blue Cluster (Diagnostic Accuracy & Performance Metrics): Represents the statistical evaluation of AI models. It is dominated by methodological and performance evaluation terms, including “sensitivity and specificity,” “receiver operating characteristic,” “diagnostic test accuracy study,” and “algorithm.”
- The Yellow Cluster (Algorithm Training & Oncology): A specialized sub-network located between the imaging and metrics clusters, highlighting “training,” “algorithms,” and targeted pathological applications such as “bone tumor.”
- The Purple Cluster (Anatomical Classification & Reliability): A compact cluster blending into the core network, featuring terms associated with anatomical segmentation and validation, including “spine,” “classification,” and “reproducibility.”

Concurrently, the co-authorship network illuminated the collaborative infrastructure of the field, identifying prominent, highly centralized research hubs (Figure 6). Key clusters are anchored by highly prolific investigators such as Schwab, JH; Kim, JS; Kunze, KN; and Ramkumar, PN. The map reveals that while tightly knit collaborative sub-networks dominate the core of orthopaedic AI research, several isolated clinical groups operate on the periphery, highlighting opportunities for broader international multicenter integration.

## 4. Discussion

The global landscape of orthopaedic artificial intelligence research has experienced definitive rapid growth over the past decade. However, framing this trajectory through a subspecialty lens reveals that academic output remains highly fragmented, unevenly distributed [1,2,3]. While adult reconstruction and spine surgery heavily dominate the literature due to data accessibility, critical fields like foot and ankle surgery, pediatric orthopaedics, hand & wrist and shoulder & elbow surgeries remain markedly underrepresented [4,5,9]. The key insight generated by this mapping study is the substantial imbalance in research activity across orthopaedic domains [8,13]. This structural imbalance indicates that the contemporary orthopaedic AI agenda is heavily influenced by technical feasibility and localized digital convenience rather than a uniform distribution across all subspecialties. Placed in a broader biomedical context, this outpacing of rigorous clinical validation by rapid methodological innovation mirrors systemic trends observed across general medical artificial intelligence domains [1,2,3]. Crucially, this observed subspecialty imbalance must not be interpreted solely as a direct reflection of clinical priority or disease burden. Because this study evaluates research activity through publication volume a higher publication count in domains like arthroplasty indicates technical convenience and data accessibility rather than an absolute alignment with clinical priorities. Our findings align with several recent bibliometric analyses demonstrating exponential publication growth and polarized geographic productivity within orthopaedic AI [10,11,12]. However, while these recent reports provide excellent macro-level overviews of global trends, the principal distinctive contribution of the present study lies in its multi-dimensional taxonomy. Rather than treating orthopaedics as a monolithic entity, our granular mapping of validated clinical subspecialties (AAOS) against specific AI modalities (ACM) reveals a stark internal polarization. This subspecialty-by-technology analysis explicitly demonstrates that algorithmic advancement is not uniformly distributed across the musculoskeletal spectrum, but rather heavily isolated within data-rich domains.

### 4.1. Growth of AI Research and Contextual Interpretation

The rapid expansion of orthopaedic AI research observed across the evaluated decade parallels broader advancements in computational infrastructure and healthcare digitization. Lowered technical barriers have catalyzed the swift adoption of complex machine learning models [4,5]. However, as underscored by foundational bibliometric frameworks, accelerated publication velocity must not be conflated with genuine scientific maturity or therapeutic readiness [13]. The current expansionary wave represents an early exploratory developmental phase rather than a milestone of established bedside integration. Without deliberate, corrective research prioritizations, technologically convenient questions will continue to eclipse core clinical dilemmas [9,13].

### 4.2. Subspecialty Distribution and Data-Driven Patterns

The stark discrepancy in research distribution across the standardized subspecialty taxonomies exposes the limitations of purely data-driven research paradigms. Arthroplasty and spine surgery command a disproportionate share of the literature, a trend that is highly consistent with patterns observed in general musculoskeletal imaging research [4,7,9]. These subspecialties directly benefit from highly standardized imaging protocols, anatomical homogeneity, and massive, well-curated patient registries that facilitate rapid neural network training and internal validation.

Conversely, subspecialties defined by severe anatomical variability, cross-sectional distortion, and heterogeneous clinical presentations—such as pediatric deformities or complex foot and ankle trauma—suffer from systemic academic exclusion. By failing to account for these non-standardized pathologies, the prevailing literature presents an asymmetric and incomplete reflection of the true global orthopaedic disease burden. We hypothesize that the pronounced disparity in publication volumes across orthopaedic subspecialties may be fundamentally attributed to differences in data standardization and clinical imaging density; however, it is important to note that publication volume alone does not directly establish model generalizability or clinical readiness.

### 4.3. AI Modalities and Methodological Trends

In alignment with clinical radiology benchmarks, deep learning architectures—specifically convolutional neural networks (CNNs)—remain the dominant paradigm within orthopaedic AI literature [5,7]. These models have demonstrated strong performance in high-throughput image classification, diagnostic screening, and automated segmentation tasks. Nevertheless, an overreliance on computer vision risks bottlenecking methodological diversity. Traditional machine learning algorithms maintain a vital, complementary role, especially in risk stratification and prognostic survival modeling using structured clinical variables [6,8]. Preserving this methodological pluralism is essential, as traditional models often yield superior interpretability and align more closely with clinical decision-making frameworks.

Furthermore, the recent proliferation of generative AI and Large Language Models (LLMs) has introduced a novel research paradigm focused on patient communication and health literacy. A growing body of literature is currently evaluating the accuracy, reliability, and readability of AI chatbots in generating patient education materials and rehabilitation guidance across diverse orthopaedic domains, including sports medicine, foot and ankle surgery, and adult reconstruction [26,27,28,29,30]. Integrating these generative tools safely requires balancing clinical accuracy with patient-level comprehension, further highlighting the need for methodological diversification beyond traditional image-based algorithms.

### 4.4. Data Standardization and Algorithmic Performance

As often observed in rapidly emerging digital health fields, much of the current orthopaedic AI literature appears predominantly localized to early-phase, proof-of-concept designs. The current literature is overwhelmingly localized to retrospective, single-center, proof-of-concept designs. In a surgical discipline where physical interventions carry irreversible anatomical consequences, this gap between technical feasibility and clinical validation demands further attention and targeted future investigations. Future research paradigms must prioritize absolute robustness, algorithmic reproducibility, and cross-institutional validation over superficial technological novelty.

### 4.5. Strengths and Limitations

This study benefits from a comprehensive bibliometric mapping approach spanning a decade of orthopaedic AI research and integrating data from two premier bibliographic indices. The implementation of a standardized subspecialty framework maximizes clinical interpretability, while automated data workflows minimize subjective extraction bias.

Several limitations must be acknowledged. Bibliometric metrics capture academic volume and citation velocity but cannot directly measure real-world clinical efficacy or bedside implementation. Additionally, a key conceptual limitation of this study is that the identified subspecialty imbalances were evaluated based on academic literature volume rather than being directly correlated with external metrics such as global disease burden, surgical volume, or clinical registry sizes. Consequently, fields labeled as ‘underrepresented’ (e.g., pediatric orthopaedics and foot & ankle surgery) reflect a lower relative publication density within indexed AI literature, which may not fully represent their true clinical demand or socio-economic weight in orthopaedic practice. Future research combining bibliometric trends with quantitative healthcare utilization or disability-adjusted life years (DALYs) data is warranted to establish a more rigorous measure of authentic clinical alignment. In this context, due to the large scale of the dataset, a granular evaluation of the Journal of Bone and Joint Surgery (JBJS) Levels of Evidence was not feasible. Future investigations employing manual text curation or advanced multi-label classification are required to analyze exact study designs at a population level.

Finally, subspecialty classification relied on a keyword-based framework aligned with standardized taxonomies. While this intentional simplification enabled reproducible analysis across a massive corpus, it may not fully capture interdisciplinary studies spanning multiple anatomical boundaries or overlapping surgical domains. In addition, because this macro-level bibliometric design relies on broad keyword matching rather than manual full-text screening, it is possible that studies mentioning arthroplasty-related terminology secondarily—rather than as the primary clinical focus—were inadvertently captured. As highlighted by the complexity of the corpus, this inherent limitation of automated search strategies requires careful evaluation, as it may potentially inflate the publication representation within certain subspecialties. Additionally, the automated classification algorithm was validated using a purely random 50-article pilot sample. While this sample size is adequate for identifying macro-level corpus trends, it may lack sufficient statistical power to precisely evaluate algorithm sensitivity for heavily underrepresented clinical domains.

## 5. Conclusions

This study highlights a decade of remarkable quantitative growth in orthopaedic artificial intelligence research, alongside a critical structural polarization. While highly standardized, image-heavy subspecialties like arthroplasty and spine surgery account for the vast majority of research activity, data-sparse domains such as pediatric orthopaedics and foot and ankle surgery remain technologically underserved. This uneven evolution risks creating a potential research disparity across the orthopaedic landscape. Moving forward, the true value of artificial intelligence will not be realized by merely compounding publications in already data-dense fields. Instead, a paradigm shift is required: academic consortia, clinical researchers, and software engineers must cross-collaborate to establish multi-institutional, standardized registries for underrepresented subspecialties. By prioritizing cross-disciplinary data integration and targeting research toward these underrepresented domains, the orthopaedic community can foster a more equitable and comprehensive development of AI tools across all subspecialties. Furthermore, to overcome the inherent boundaries of large-scale algorithmic keyword scanning, future bibliometric frameworks should aim to integrate manual full-text appraisals, JBJS evidence grading, and global disease burden metrics to provide a more granular assessment of AI maturity in orthopaedics.

## Figures and Tables

**Figure 1:**
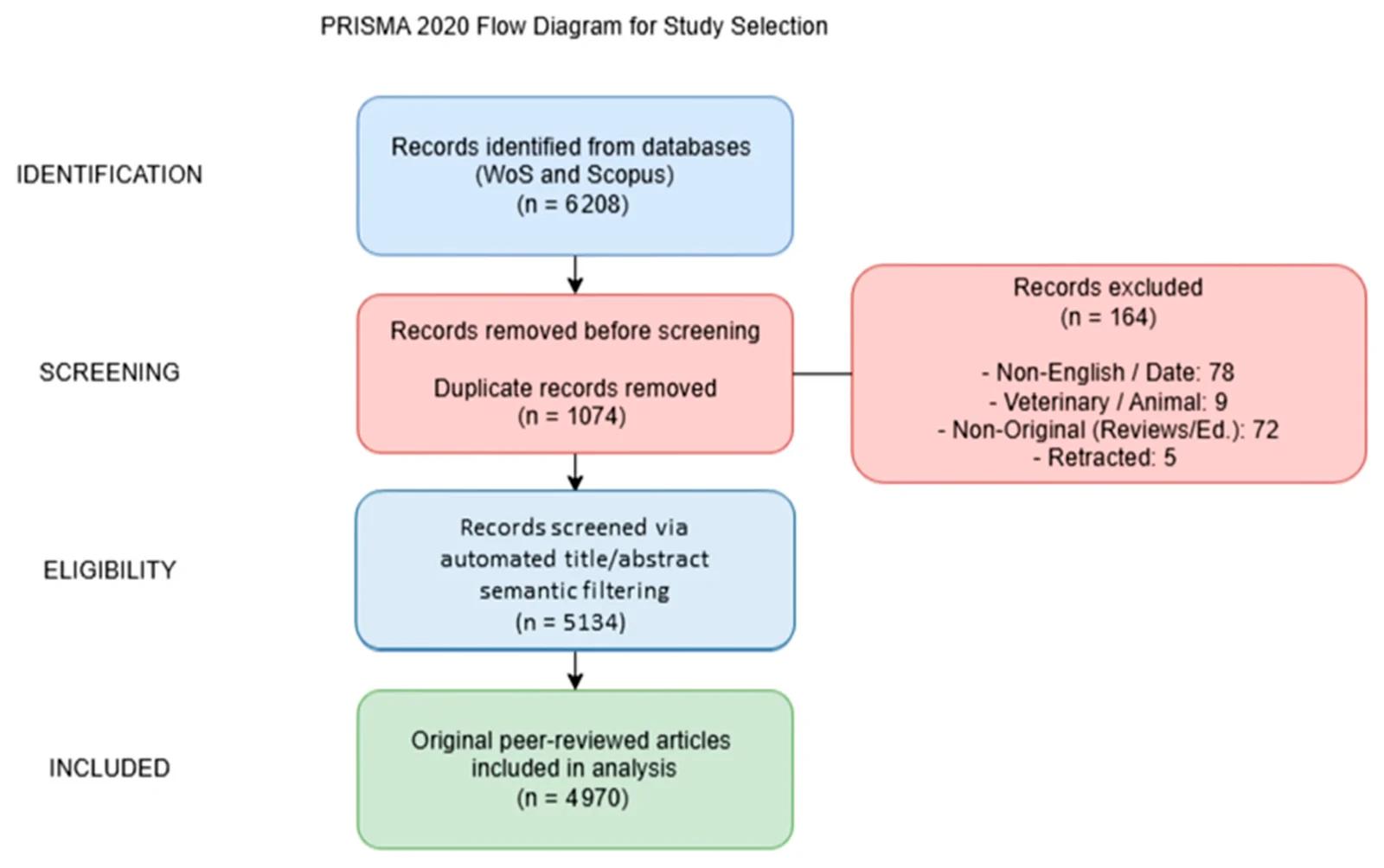
PRISMA 2020 flow diagram illustrating the selection process of the study cohort. The diagram details the identification, screening, eligibility assessment, and final inclusion of articles, along with specific reasons for exclusion at each stage (e.g., non-English language, veterinary studies, and retracted publications). (*n* = number of records).

**Figure 2:**
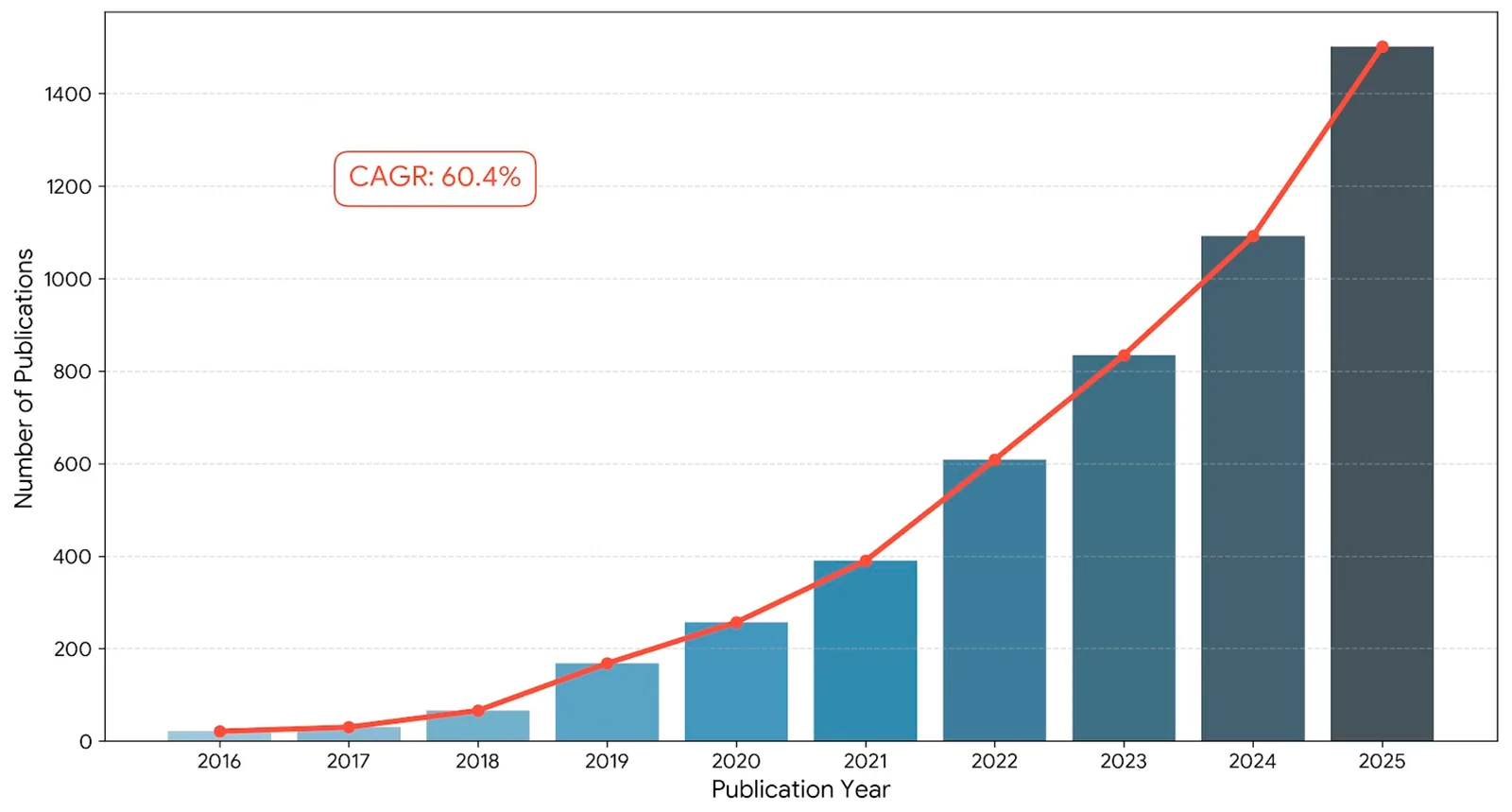
Annual scientific production growth (2016–2025). The bar chart illustrates the exponential increase in publication volume, with the red line indicating the trend trajectory. A Compound Annual Growth Rate (CAGR) of 60.4% was observed over the decade, with a significant surge in the post-pandemic era.

**Figure 3:**
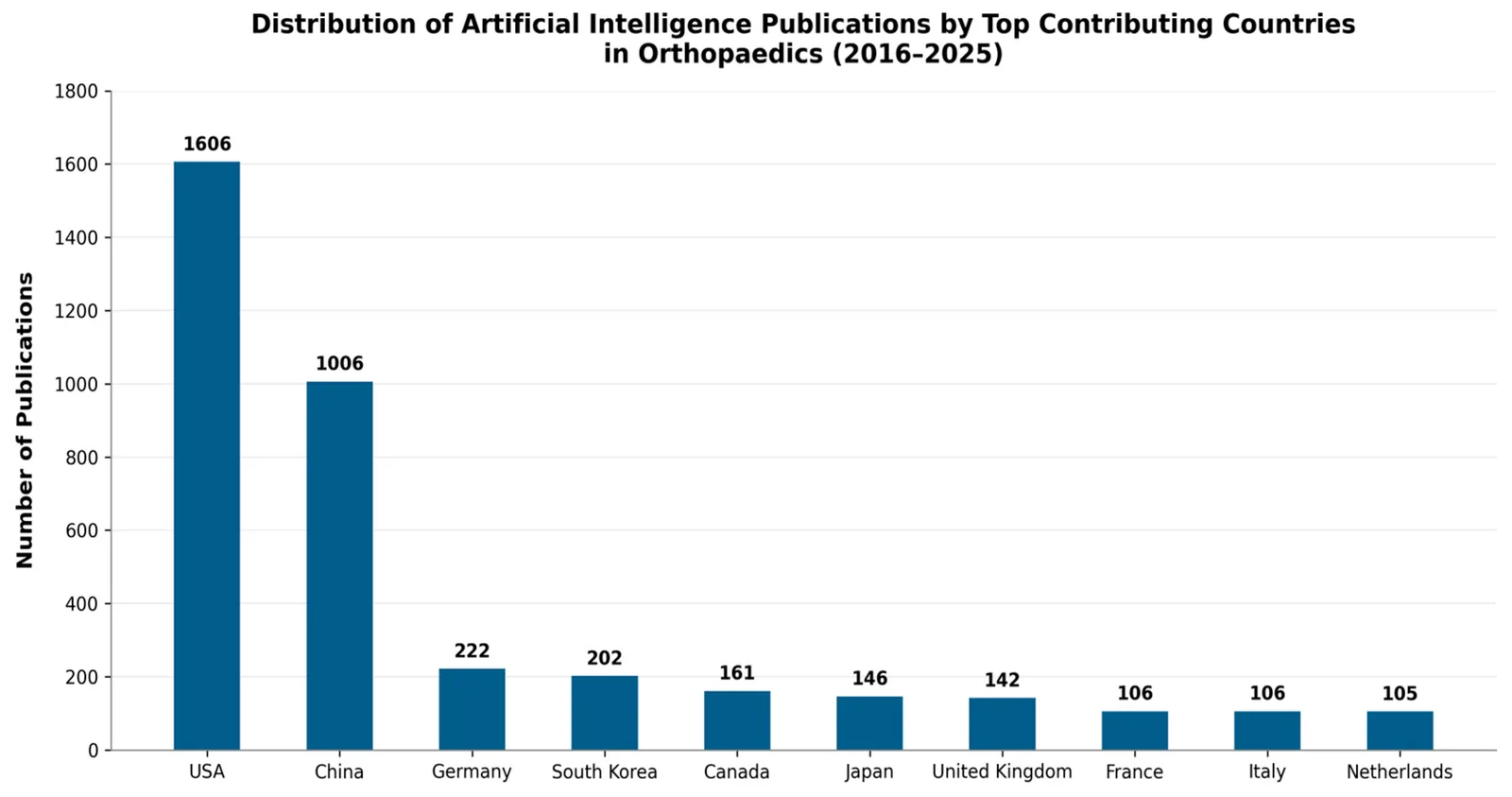
Top 10 most productive countries. The horizontal bar chart displays the leading nations in AI-orthopaedics research. The USA and China dominate the field, collectively accounting for over half (52.5%) of the global output.

**Figure 4:**
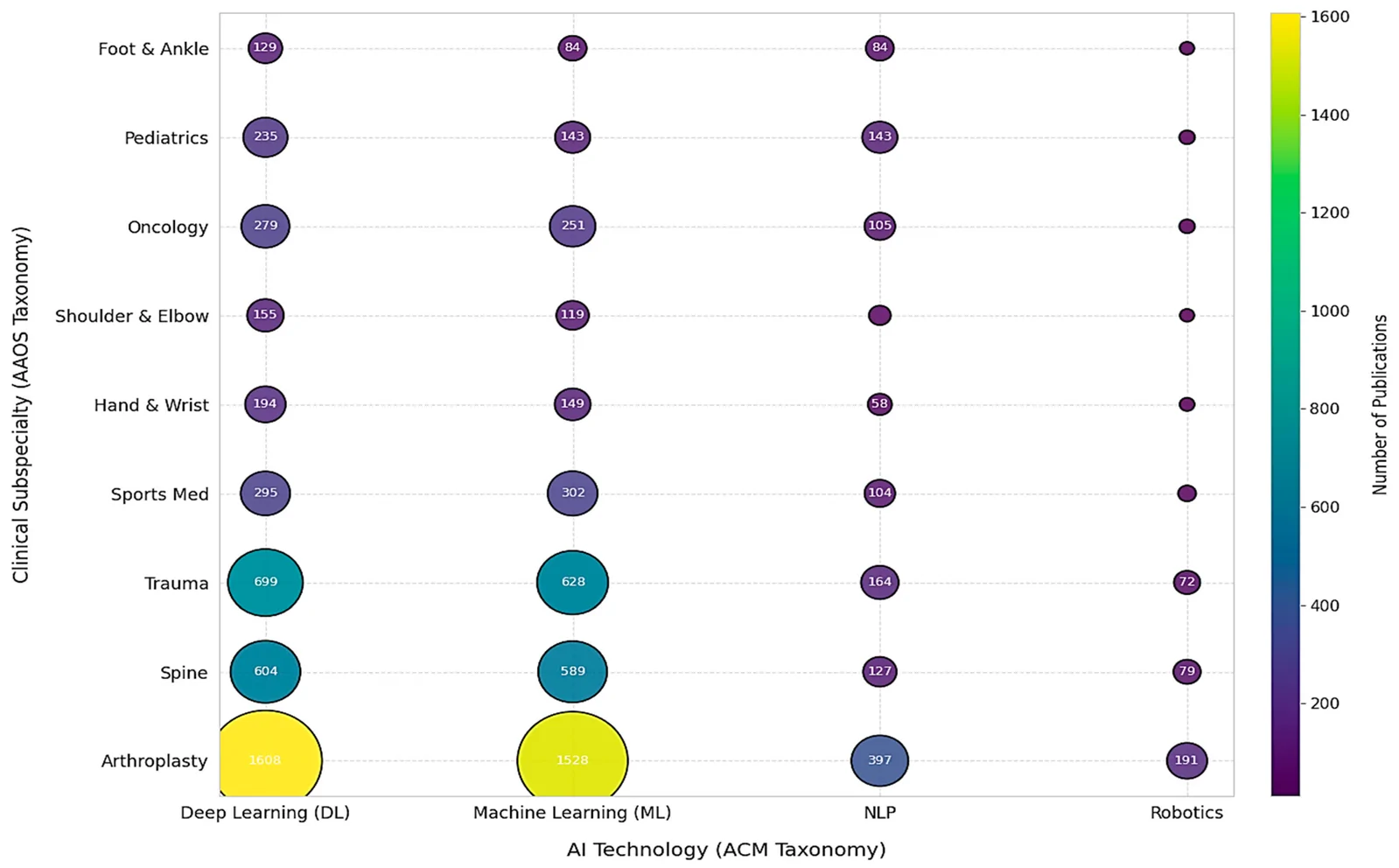
Bubble chart illustrating the intersection of AI modalities and clinical sub-specialties. Bubble size and color intensity correspond to the number of publications. Deep Learning applications in Arthroplasty and Spine surgery represent the most densely researched clusters. (ACM: Association for Computing Machinery; AAOS: American Academy of Orthopaedic Surgeons). The “Al-Clinical” Heatmap (Intersection of Modalities and Subspecialties).

**Figure 5:**
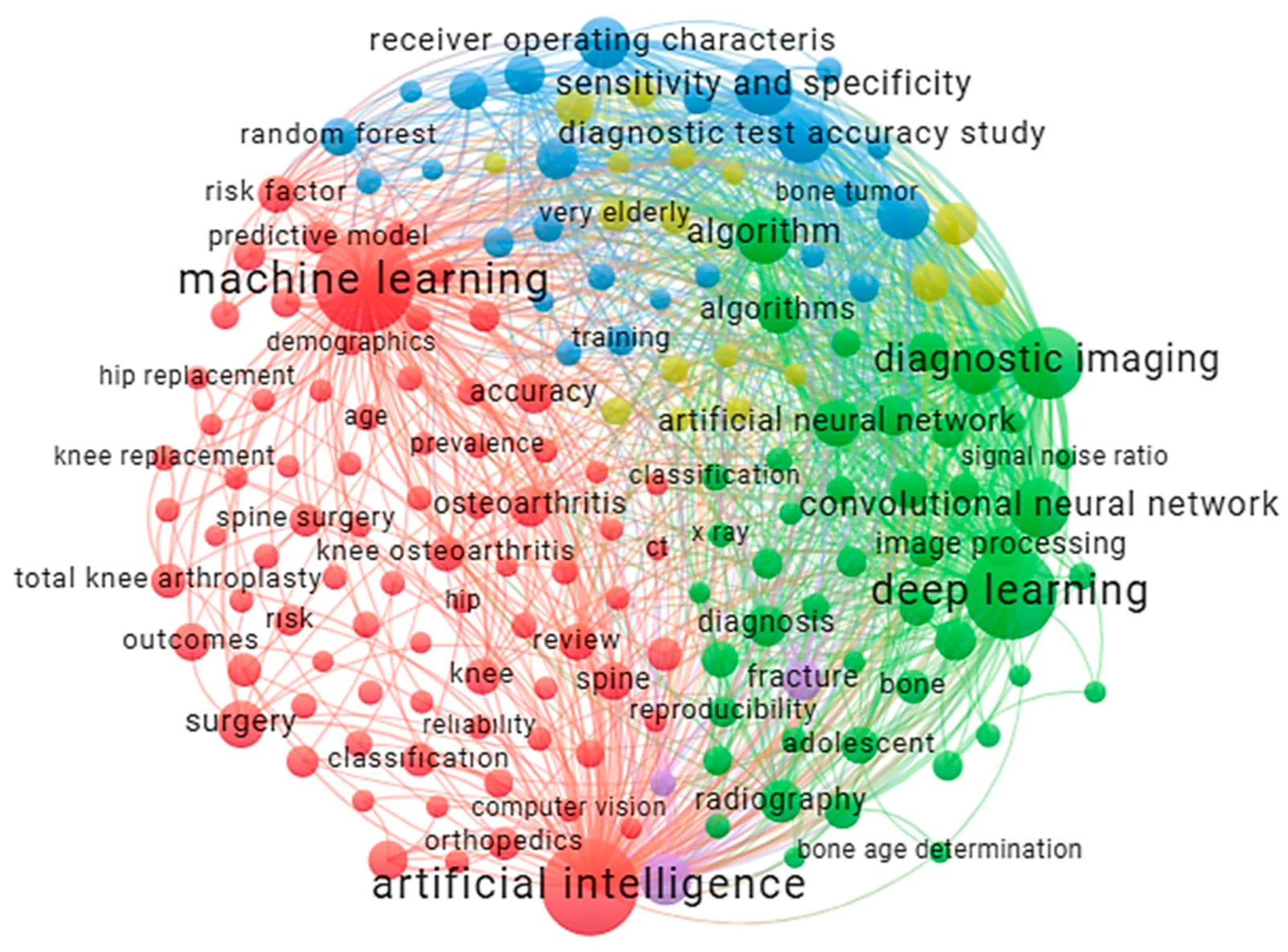
Keyword co-occurrence network analysis visualizing the thematic clusters and interconnected domains in orthopaedic artificial intelligence research. Nodes of the same color represent items belonging to the same thematic cluster.

**Figure 6:**
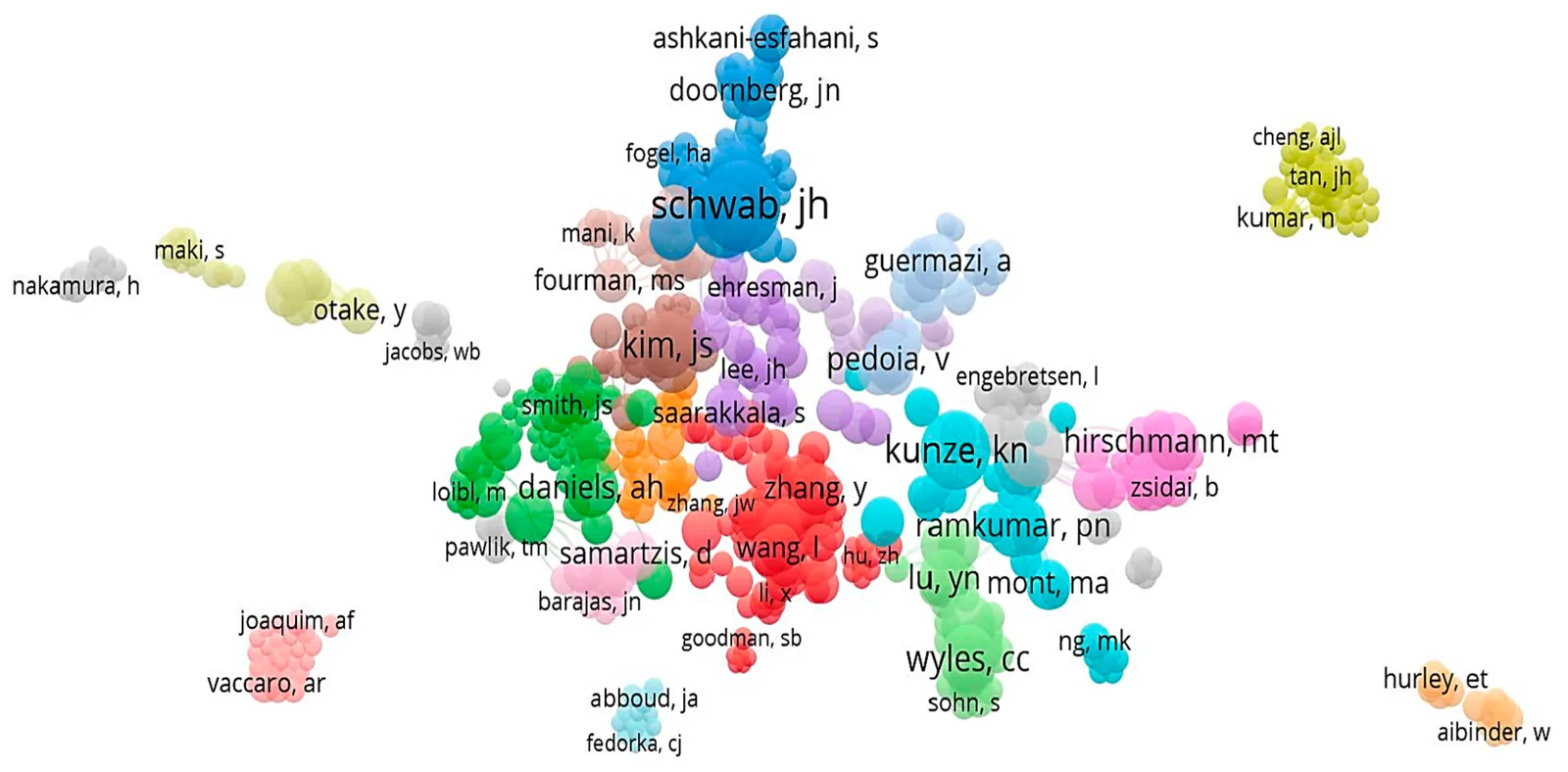
Co-authorship network analysis highlighting prominent research groups and international collaborations. Nodes represent individual authors, with node size proportional to publication volume. The connecting lines indicate co-authorship links, and colors denote distinct, tightly knit collaborative clusters anchoring the orthopaedic AI research landscape.

**Table 1:** Annual distribution of publications (2016–2025).

Year	Count	% of Total
2016	21	0.40%
2017	30	0.60%
2018	66	1.30%
2019	168	3.40%
2020	257	5.20%
2021	390	7.80%
2022	609	12.30%
2023	835	16.80%
2024	1092	22.00%
2025	1502	30.20%
Total	4970	100%

**Table 2:** Top 10 Most Productive Countries.

Rank	Country	Count	% of Global Output
1	USA	1606	32.30%
2	China	1006	20.20%
3	Germany	222	4.50%
4	South Korea	202	4.10%
5	Canada	161	3.20%
6	Japan	146	2.90%
7	United Kingdom	142	2.90%
8	France	106	2.10%
9	Italy	106	2.10%
10	Netherlands	105	2.10%
-	Other Countries (*n* = 76)	1168	23.50%
Total	Global Output	4970	100%

**Table 3:** Top 10 Most Productive Journals.

Rank	Journal Name	Count
1	European Spine Journal	107
2	Int. J. Computer Assisted Radiology and Surgery	104
3	Osteoarthritis and Cartilage	101
4	Skeletal Radiology	98
5	BMC Musculoskeletal Disorders	90
6	Journal of Arthroplasty	87
7	Spine Journal	69
8	Journal of Orthopaedic Surgery and Research	67
9	Knee Surgery Sports Traumatology Arthroscopy	67
10	Journal of Orthopaedic Research	62
-	Remaining Unique Journals (*n* = 984)	4118
Total	All Journals Combined	4970

Note: *n* = 984 represents the number of additional unique journals that published at least one relevant article outside the top 10.

**Table 4:** Distribution by Clinical Subspecialties (AAOS) and AI Modalities (ACM).

Classification System	Category	Count (n)
AAOSs	Arthroplasty (Adult Reconstruction)	3491
	Spine Surgery	2292
	Trauma	1495
	Sports Medicine	831
	Hand & Wrist	412
	Shoulder & Elbow	331
	Musculoskeletal Oncology	545
	Pediatric Orthopaedics	519
	Foot & Ankle	250
AI Taxonomy (ACM)	Deep Learning (DL)	2286
	Machine Learning (ML)	2110
	Natural Language Processing (NLP)	672
	Robotics & Navigation	259

Note: Counts are not mutually exclusive as articles can be classified into multiple subspecialties based on overlapping keyword criteria.

## Data Availability

Due to commercial licensing restrictions of Clarivate Analytics (Web of Science) and Elsevier (Scopus), raw proprietary bibliographic exports cannot be redistributed publicly. However, to ensure full transparency and reproducibility, all persistent identifiers (DOIs), derived study-level classifications, keyword dictionaries, the pilot validation cohort, and Python analysis codes are provided in the Supplementary Materials (Tables S1–S4 and File S1) accompanying this article.

## Supplementary Materials

The following supporting information can be downloaded at: https://www.mdpi.com/article/10.3390/healthcare14193183/s1, Table S1: Detailed Search Strategy and Boolean Syntax; Table S2: Classification Dictionaries with Reference Standards; Table S3: Category-Specific Validation Metrics and Confusion Matrix (50-Article Random Sample); Table S4: Dataset extract detailing study-level classifications, persistent identifiers (DOIs), clinical subspecialties (AAOS taxonomy), artificial intelligence modalities (ACM taxonomy), and primary clinical objectives for the included articles; File S1: Python source code utilized for automated bibliographic parsing, multi-dimensional classification, and data processing.
