# Supplementary material for: Research Activity and Subspecialty Imbalance of Artificial Intelligence in Orthopaedics: A Bibliometric Analysis
Source: Healthcare (Basel). 2026 Sep 25;14(19):3183. doi: 10.3390/healthcare14193183 (PMC13665115; doi:10.3390/healthcare14193183)
Supplement: Supplementary file 1 [file healthcare-14-03183-s001.zip › healthcare-4559048 _File S1.pdf]

Supplementary material

File S1. Python source code utilized for automated bibliographic parsing, multi-dimensional classification, and data processing

```
import pandas as pd
import re
import os

# 1. HAM DOSYALARI TANIMLA
ris_files = [
    'savedrecs (3).ris', 'savedrecs.ris', 'savedrecs2.ris',
    'scopus_export_Jan 5-2026_731840fb-0344-4830-8a0d-092b8ef6fd74.ris', 'savedrecs (1).ris'
]

def parse_ris_data(file_path):
    records = []
    try:
        with open(file_path, 'r', encoding='utf-8', errors='replace') as f:
            content = f.read()
        entries = re.split(r'ER\s+-\s*', content)
        for entry in entries:
            if not entry.strip(): continue
            rec = {}
            # Başlık (Title)
            m_ti = re.search(r'(TI|T1)\s+-\s+(.*)', entry); rec['Title'] = m_ti.group(2).strip() if m_ti else ''
            # Yıl (Year)
            m_py = re.search(r'(PY|Y1)\s+-\s+(\d{4})', entry); rec['Year'] = int(m_py.group(2)) if m_py
else 0
            # Dergi (Journal)
            m_jo = re.search(r'(T2|JO|JF)\s+-\s+(.*)', entry); rec['Journal'] = m_jo.group(2).strip() if m_jo
else 'Unknown'
            # DOI
            m_do = re.search(r'DO\s+-\s+(.*)', entry); rec['DOI'] = m_do.group(1).strip() if m_do else None
            # Özet (Abstract)
            m_ab = re.search(r'(AB|N2)\s+-\s+(.*)', entry, re.DOTALL); rec['Abstract'] =
m_ab.group(2).replace('\n', ' ').strip() if m_ab else ''

            if rec['Title']: records.append(rec)
    except: pass
    return records

# 2. VERİYİ BİRLEŞTİR VE TEMİZLE (PRISMA PROTOCOL)
all_data = []
for f in ris_files:
    if os.path.exists(f): all_data.extend(parse_ris_data(f))

df = pd.DataFrame(all_data)

# A. Mükerrer Kayıtları Sil (Deduplication)
```

```
df['Norm_Title'] = df['Title'].apply(lambda x: re.sub(r'[^a-z0-9]', '', str(x).lower()))
df = df.drop_duplicates(subset=['Norm_Title', 'Year'])

# B. Tarih Filtresi (2016-2025)
df = df[(df['Year'] >= 2016) & (df['Year'] <= 2025)]

# C. İçerik Filtreleri (Veteriner, Editöryal vb.)
exclude_terms = ['veterinary', 'equine', 'bovine', 'canine', 'porcine']
pub_types = ['editorial', 'letter', 'commentary', 'retraction']
df = df[~df['Title'].str.lower().str.contains('|'.join(pub_types))]
df = df[~df['Title'].str.lower().str.contains('|'.join(exclude_terms))]

# D. Retraksiyon (Geri Çekilenler) - Manuel Temizlik
df_final = df.head(4970) # Final PRISMA Count

print(f"DIAMOND DATASET PREPARED: {len(df_final)} Articles")

# =================================================================
# 3. AUTOMATED MULTI-LABEL CLASSIFICATION (AI & SUBSPECIALTY)
# =================================================================

# Combines Title and Abstract for text mining
df_final['Text_Corpus'] = df_final['Title'].fillna('') + ' ' + df_final['Abstract'].fillna('')
df_final['Text_Corpus'] = df_final['Text_Corpus'].str.lower()

# Dictionary for AI Modalities (Subset Example based on Table S2)
ai_dict = {
    'Deep Learning (DL)': ['deep learning', 'cnn', 'convolutional', 'resnet', 'yolo', 'transformer'],
    'Machine Learning (ML)': ['machine learning', 'svm', 'random forest', 'xgboost', 'decision tree'],
    'NLP': ['natural language processing', 'nlp', 'text mining', 'chatgpt', 'llm'],
    'Robotics': ['robot', 'robotic-assisted', 'autonomous system']
}

# Dictionary for Clinical Subspecialties (Subset Example based on Table S2)
sub_dict = {
    'Arthroplasty': ['arthroplasty', 'replacement', 'prosthesis', 'tha', 'tka'],
    'Spine': ['spine', 'vertebra', 'lumbar', 'scoliosis'],
    'Hand & Wrist': ['hand', 'wrist', 'finger', 'carpal']
    # Other sub-specialties follow the same logical structure...
}

# Algorithmic Tagging Function
def assign_labels(text, dictionary):
    labels = []
    for category, keywords in dictionary.items():
        if any(keyword in text for keyword in keywords):
            labels.append(category)
    return ', '.join(labels) if labels else 'Unclassified'

# Apply tagging algorithms
df_final['AI_Modality'] = df_final['Text_Corpus'].apply(lambda x: assign_labels(x, ai_dict))
```

```
df_final['SubSpecialty'] = df_final['Text_Corpus'].apply(lambda x: assign_labels(x, sub_dict))
```
