# Supplementary material for: Research Activity and Subspecialty Imbalance of Artificial Intelligence in Orthopaedics: A Bibliometric Analysis
Source: Healthcare (Basel). 2026 Sep 25;14(19):3183. doi: 10.3390/healthcare14193183 (PMC13665115; doi:10.3390/healthcare14193183)
Supplement: Supplementary file 1 [file healthcare-14-03183-s001.zip › healthcare-4559048_Tables S1–S3.pdf]

Supplementary materials

Table S1. Detailed Search Strategy and Boolean Syntax.

Constructed in accordance with PRESS 2015 Guidelines. Asterisks (*) denote wildcard truncation.

Database	Search Query String (Syntax)	
**Web of Science (WoS)**	TS=(("Artificial Intelligence" OR "Machine Learning" OR "Deep Learning" OR "Neural Network*" OR "Computer Vision" OR "Natural Language Processing" OR "Generative AI" OR "Large Language Model*" OR "ChatGPT" OR "Transformer Model*" OR "Reinforcement Learning") AND ("Orthop*" OR "Bone" OR "Joint" OR "Fracture" OR "Spine" OR "Arthroplasty" OR "Musculoskeletal" OR "Osteoarthritis" OR "Scoliosis" OR "ACL" OR "Meniscus")) AND PY=(2016-2025) AND DT=(Article)	Number of Results: 3369<br>Language: English<br>Searching Date: 04.01.2026
**Scopus**	TITLE ("Artificial Intelligence" OR "Machine Learning" OR "Deep Learning" OR "Neural Network*" OR "Computer Vision" OR "Natural Language Processing" OR "Generative AI" OR "Large Language Model*" OR "ChatGPT" OR "Transformer Model*") AND TITLE ("Orthop*" OR "Bone" OR "Joint" OR "Fracture" OR "Spine" OR "Arthroplasty" OR "Musculoskeletal" OR "Osteoarthritis" OR "Scoliosis") AND PUBYEAR > 2015 AND PUBYEAR < 2026 AND SUBJAREA (MEDI) AND (LIMIT-TO (DOCTYPE , "ar") OR LIMIT-TO (DOCTYPE , "re")) AND (LIMIT-TO (LANGUAGE , "English"))	Number of Results: 2839<br>Language: English<br>Searching Date: 04.01.2026

Table S2. Classification Dictionaries with Reference Standards.

Validated via pilot testing on 50 randomly selected articles.

Category / Dimension	Sub-Category	Validation Standard	Inclusion Keywords (Search Strings)
AAOS Sub-Specialty	Adult Reconstruction	MeSH: D019643	arthroplasty, replacement, prosthesis, tha, tka, hemiarthroplasty, periprosthetic
	Spine	MeSH: D013131	spine, vertebra, lumbar, cervical, thoracic, scoliosis, kyphosis, disc herniation
	Trauma	MeSH: D014193	fracture, trauma, dislocation, nonunion, malunion, intramedullary nail, plate fixation
	Sports Medicine	MeSH: D013178	arthroscopy, sport, acl, meniscus, ligament, athlete, rotator cuff (sports context)
	Hand & Wrist	MeSH: D006225	hand, wrist, finger, carpal, metacarpal, scaphoid, flexor tendon, replantation
	Shoulder & Elbow	MeSH: D012782	shoulder, elbow, clavicle, scapula, humerus, glenoid, rotator cuff, labrum
	Foot & Ankle	MeSH: D005528	foot, ankle, calcaneus, talus, metatarsal, hallux, achilles tendon, plantar fasciitis
	Musculoskeletal Oncology	MeSH: D009369	tumor, cancer, oncology, sarcoma, metastasis, osteosarcoma, ewing
	Pediatric Orthopedics	MeSH: D010372	pediatric, child, adolescent, juvenile, congenital, cerebral palsy, dysplasia
AI Technology	Deep Learning	ACM CCS: 10010147	deep learning, cnn, convolutional, rnn, u-net, resnet, transformer, gan, yolo
	Machine Learning	ACM CCS: 10010147	machine learning, svm, random forest, decision tree, xgboost, gradient boosting, regression
	NLP	ACM CCS: 10002951	natural language processing, nlp, text mining, sentiment analysis, bert, gpt, llm, chatgpt
	Robotics	ACM CCS: 10010520	robot, robotic-assisted, haptic, autonomous system, navigation

Table S3. Category-Specific Validation Metrics and Confusion Matrix (50-Article Random Sample)

This table presents the detailed performance metrics derived from a purely random pilot sample of 50 articles. The manually adjudicated gold standard was established by two independent reviewers who were blinded to the automated algorithm's output. The metrics demonstrate the algorithm's multi-label classification accuracy across both clinical subspecialties and artificial intelligence modalities. The Cohen’s Kappa column represents the inter-rater agreement between the two independent human reviewers for each respective category.

Category / Label	TP	TN	FP	FN	Sensitivity (%)	Specificity (%)	Cohen’s Kappa
AAOS Sub-Specialties							
Adult Reconstruction	16	31	2	1	94.1	93.9	0.88
Spine Surgery	11	37	1	1	91.7	97.4	0.89
Trauma	8	40	1	1	88.9	97.6	0.85
Sports Medicine	5	43	1	1	83.3	97.7	0.81
Hand & Wrist	3	47	0	0	100.0	100.0	1.00
Shoulder & Elbow	2	47	1	0	100.0	97.9	0.79
Musculoskeletal Oncology	3	46	1	0	100.0	97.9	0.84
Pediatric Orthopaedics	2	47	0	1	66.7	100.0	0.79
Foot & Ankle	1	48	1	0	100.0	98.0	0.66
AI Technologies (ACM)							
Deep Learning (DL)	22	25	2	1	95.7	92.6	0.88
Machine Learning (ML)	19	28	1	2	90.5	96.6	0.87
Natural Language Processing	5	45	0	0	100.0	100.0	1.00
Robotics & Navigation	2	48	0	0	100.0	100.0	1.00

TP: True Positive, TN: True Negative, FP: False Positive, FN: False Negative. All metrics evaluated against the manually adjudicated dual-reviewer gold standard.
