# Supplementary material for: Research Activity and Subspecialty Imbalance of Artificial Intelligence in Orthopaedics: A Bibliometric Analysis
Source: Healthcare (Basel). 2026 Sep 25;14(19):3183. doi: 10.3390/healthcare14193183 (PMC13665115; doi:10.3390/healthcare14193183)
Supplement: Supplementary file 1 [file healthcare-14-03183-s001.zip › healthcare-4559048_Table S4.pdf]

Table S4. Dataset extract detailing study-level classifications, persistent identifiers (DOIs), clinical subspecialties (AAOS taxonomy), artificial intelligence modalities (ACM taxonomy), and primary clinical objectives for the included articles.

Article ID	Persistent Identifier (DOI)	Publication Year	AAOS Subspecialty (Multi-label)	ACM AI Modality (Multi-label)	Clinical Objective
1	10.1016/j.spinee.2020.04.001	2021	Sports Medicine; Trauma; Spine Surgery; Pediatric Orthopaedics; Arthroplasty	Natural Language Processing (NLP); Machine Learning (ML); Robotics; Deep Learning (DL)	Prognosis & Prediction
2	10.1186/s42836-025-00302-5	2025	Arthroplasty	Machine Learning (ML); Robotics	Prognosis & Prediction
3	10.1016/j.arthro.2020.09.012	2021	Sports Medicine	Unclassified	Prognosis & Prediction
4	N/A	2024	Sports Medicine; Trauma; Spine Surgery; Pediatric Orthopaedics; Arthroplasty	Natural Language Processing (NLP); Machine Learning (ML); Robotics; Deep Learning (DL)	Prognosis & Prediction
5	10.1016/j.knee.2025.05.018	2025	Sports Medicine	Natural Language Processing (NLP)	Unclassified
6	10.1007/s00264-021-05175-2	2021	Arthroplasty	Machine Learning (ML); Robotics	Unclassified
7	10.1177/21925682231164935	2024	Sports Medicine; Trauma; Spine Surgery; Pediatric Orthopaedics; Arthroplasty	Natural Language Processing (NLP); Machine Learning (ML); Robotics; Deep Learning (DL)	Prognosis & Prediction
8	10.1177/10225536251350411	2025	Arthroplasty	Natural Language Processing (NLP)	Prognosis & Prediction
9	10.1016/j.spinee.2022.10.011	2023	Sports Medicine; Trauma; Spine Surgery; Pediatric Orthopaedics; Arthroplasty	Natural Language Processing (NLP); Machine Learning (ML); Robotics; Deep Learning (DL)	Prognosis & Prediction
10	10.1007/s12178-020-09600-8	2020	Unclassified	Machine Learning (ML); Deep Learning (DL)	Unclassified
11	10.1007/s00167-023-07338-7	2023	Sports Medicine; Trauma; Spine Surgery; Pediatric Orthopaedics; Arthroplasty	Natural Language Processing (NLP); Machine Learning (ML); Robotics; Deep Learning (DL)	Prognosis & Prediction
12	10.1177/17531934241312896	2025	Trauma	Machine Learning (ML)	Prognosis & Prediction
13	10.1186/s12891-024-07434-w	2024	Sports Medicine; Trauma; Arthroplasty	Machine Learning (ML)	Prognosis & Prediction
14	10.1177/19476035221074009	2022	Sports Medicine; Arthroplasty	Machine Learning (ML); Deep Learning (DL)	Diagnosis & Detection

15	10.5435/JAAOS-D-23-00133	2023	Arthroplasty	Natural Language Processing (NLP); Machine Learning (ML)	Prognosis & Prediction
16	10.1016/j.spinee.2025.02.010	2025	Spine Surgery	Natural Language Processing (NLP)	Diagnosis & Detection
17	10.1016/j.artd.2025.101672	2025	Arthroplasty	Machine Learning (ML)	Prognosis & Prediction
18	10.1302/0301-620X.105B12	2023	Trauma; Arthroplasty	Unclassified	Prognosis & Prediction
19	10.1007/s00586-025-09166-4	2025	Sports Medicine; Trauma; Spine Surgery; Pediatric Orthopaedics; Arthroplasty	Natural Language Processing (NLP); Machine Learning (ML); Robotics; Deep Learning (DL)	Prognosis & Prediction
20	10.1002/ksa.12241	2025	Sports Medicine; Trauma	Machine Learning (ML); Deep Learning (DL)	Prognosis & Prediction
21	10.1016/j.jor.2023.08.002	2023	Trauma; Spine Surgery	Robotics	Unclassified
22	10.1055/s-0045-1811667	2025	Spine Surgery	Robotics; Deep Learning (DL)	Surgical Planning
23	10.1177/03635465251360240	2025	Sports Medicine; Trauma; Arthroplasty	Machine Learning (ML)	Prognosis & Prediction
24	10.1007/s00167-022-07117-w	2023	Sports Medicine; Trauma	Machine Learning (ML)	Prognosis & Prediction
25	10.1016/j.jseint.2024.07.002	2025	Sports Medicine; Trauma; Spine Surgery; Pediatric Orthopaedics; Arthroplasty	Natural Language Processing (NLP); Machine Learning (ML); Robotics; Deep Learning (DL)	Prognosis & Prediction
26	10.3171/2025.4.FOCUS25234	2025	Spine Surgery	Machine Learning (ML); Deep Learning (DL)	Diagnosis & Detection
27	10.1177/2192568219899944	2020	Spine Surgery	Deep Learning (DL)	Unclassified
28	10.14444/8501	2023	Spine Surgery	Machine Learning (ML)	Prognosis & Prediction
29	10.1016/j.arth.2025.05.093	2025	Sports Medicine; Trauma; Spine Surgery; Pediatric Orthopaedics; Arthroplasty	Natural Language Processing (NLP); Machine Learning (ML); Robotics; Deep Learning (DL)	Prognosis & Prediction
30	10.22038/ABJS.2022.58485.2897	2023	Arthroplasty	Machine Learning (ML); Deep Learning (DL)	Prognosis & Prediction
31	10.1016/j.jseint.2025.05.008	2025	Sports Medicine; Arthroplasty	Natural Language Processing (NLP)	Prognosis & Prediction
32	10.1007/s00264-021-05191-2	2021	Arthroplasty	Machine Learning (ML)	Unclassified
33	10.1227/neu.0000000000003360_423	2025	Spine Surgery	Machine Learning (ML)	Unclassified
34	10.1007/s12178-025-09982-7	2025	Unclassified	Natural Language Processing (NLP); Machine Learning (ML); Robotics	Prognosis & Prediction

35	10.1016/j.hansur.2025.102122	2025	Sports Medicine; Trauma; Spine Surgery; Pediatric Orthopaedics; Arthroplasty	Natural Language Processing (NLP); Machine Learning (ML); Robotics; Deep Learning (DL)	Prognosis & Prediction
36	10.1007/s00586-025-08742-y	2025	Spine Surgery	Machine Learning (ML)	Prognosis & Prediction
37	10.1186/s12891-025-08296-6	2025	Arthroplasty	Machine Learning (ML)	Prognosis & Prediction
38	10.1007/s43390-025-01087-y	2025	Sports Medicine; Trauma; Spine Surgery; Pediatric Orthopaedics; Arthroplasty	Natural Language Processing (NLP); Machine Learning (ML); Robotics; Deep Learning (DL)	Prognosis & Prediction
39	10.1007/s00256-022-04081-x	2022	Sports Medicine; Trauma	Deep Learning (DL)	Prognosis & Prediction
40	10.1097/BRS.0000000000004889	2024	Trauma; Spine Surgery	Deep Learning (DL)	Prognosis & Prediction
41	10.1016/j.jor.2024.01.016	2024	Arthroplasty	Machine Learning (ML)	Prognosis & Prediction
42	10.1002/jor.70067	2025	Sports Medicine; Trauma	Machine Learning (ML)	Prognosis & Prediction
43	10.20517/ais.2024.45	2025	Spine Surgery	Machine Learning (ML)	Segmentation & Measurement
44	10.1186/s42836-024-00256-0	2024	Arthroplasty	Machine Learning (ML)	Prognosis & Prediction
45	10.1186/s12891-025-09315-2	2025	Pediatric Orthopaedics	Natural Language Processing (NLP); Machine Learning (ML)	Diagnosis & Detection
46	10.1016/j.arth.2020.10.024	2021	Arthroplasty	Natural Language Processing (NLP); Machine Learning (ML)	Unclassified
47	10.1007/s00264-024-06369-0	2025	Arthroplasty	Machine Learning (ML)	Prognosis & Prediction
48	10.1055/a-2689-8280	2025	Pediatric Orthopaedics; Arthroplasty	Natural Language Processing (NLP); Machine Learning (ML)	Prognosis & Prediction
49	10.1016/j.nec.2023.11.009	2024	Trauma; Spine Surgery	Machine Learning (ML)	Prognosis & Prediction
50	10.1055/a-2693-0756	2025	Arthroplasty	Natural Language Processing (NLP)	Prognosis & Prediction
51	10.20517/ais.2024.73	2025	Spine Surgery	Machine Learning (ML); Robotics	Prognosis & Prediction
52	10.1177/15563316251340074	2025	Unclassified	Machine Learning (ML)	Prognosis & Prediction
53	10.1007/s00402-023-04827-9	2023	Arthroplasty	Machine Learning (ML); Deep Learning (DL)	Prognosis & Prediction
54	10.1016/j.jisako.2025.100843	2025	Sports Medicine; Trauma	Natural Language Processing (NLP)	Unclassified

55	10.1002/jor.70064	2025	Arthroplasty	Machine Learning (ML)	Prognosis & Prediction
56	10.1227/neu.0000000000002660	2024	Spine Surgery	Natural Language Processing (NLP); Machine Learning (ML); Robotics	Prognosis & Prediction
57	10.1016/j.joca.2024.03.089	2024	Arthroplasty	Natural Language Processing (NLP)	Unclassified
58	10.1186/s13037-024-00393-0	2024	Spine Surgery	Machine Learning (ML)	Prognosis & Prediction
59	10.1093/neuros/nyab227	2021	Spine Surgery	Machine Learning (ML)	Prognosis & Prediction
60	10.1302/2046-3758.142.BJR-2024-0313.R1	2025	Sports Medicine; Arthroplasty	Unclassified	Prognosis & Prediction
61	10.1177/10711007221093574	2022	Arthroplasty	Machine Learning (ML); Deep Learning (DL)	Diagnosis & Detection
62	10.3171/2025.4.SPINE241099	2025	Spine Surgery	Natural Language Processing (NLP)	Prognosis & Prediction
63	10.1016/j.arthro.2024.12.009	2025	Unclassified	Deep Learning (DL)	Unclassified
64	10.1007/s00402-024-05298-2	2024	Trauma	Natural Language Processing (NLP)	Diagnosis & Detection
65	10.1016/j.ocl.2024.07.003	2025	Arthroplasty	Robotics	Unclassified
66	10.1007/s00590-025-04198-4	2025	Sports Medicine; Pediatric Orthopaedics	Natural Language Processing (NLP)	Unclassified
67	10.1007/s00590-021-03059-0	2022	Arthroplasty	Natural Language Processing (NLP); Machine Learning (ML); Robotics	Prognosis & Prediction
68	10.4055/cios22181	2023	Sports Medicine; Trauma; Spine Surgery; Pediatric Orthopaedics; Arthroplasty	Natural Language Processing (NLP); Machine Learning (ML); Robotics; Deep Learning (DL)	Prognosis & Prediction
69	10.14245/ns.2448388.194	2024	Trauma; Spine Surgery	Machine Learning (ML); Robotics; Deep Learning (DL)	Prognosis & Prediction
70	10.4103/jllr.jllr_11_24	2024	Unclassified	Natural Language Processing (NLP)	Unclassified
71	10.1016/j.joca.2024.03.036	2024	Arthroplasty	Natural Language Processing (NLP)	Unclassified
72	10.14245/ns.2142412.206	2021	Spine Surgery; Trauma; Arthroplasty	Machine Learning (ML); Robotics	Prognosis & Prediction
73	10.1177/17531934241295493	2024	Trauma	Natural Language Processing (NLP)	Prognosis & Prediction
74	10.1002/jeo2.70548	2025	Spine Surgery; Trauma; Arthroplasty	Natural Language Processing (NLP); Machine Learning (ML)	Prognosis & Prediction
75	10.1016/j.jpra.2024.09.014	2024	Trauma; Pediatric Orthopaedics	Natural Language Processing (NLP)	Prognosis & Prediction
76	10.1016/j.cpm.2024.04.011	2024	Arthroplasty	Deep Learning (DL)	Segmentation & Measurement

77	10.1016/j.xnsj.2025.100621	2025	Trauma; Spine Surgery	Deep Learning (DL)	Prognosis & Prediction
78	10.1177/15589447241265519	2025	Sports Medicine; Trauma; Arthroplasty	Natural Language Processing (NLP)	Prognosis & Prediction
79	10.1002/jeo2.70138	2024	Trauma; Arthroplasty	Natural Language Processing (NLP)	Prognosis & Prediction
80	10.1177/21925682251344248	2026	Spine Surgery	Natural Language Processing (NLP)	Unclassified
81	10.1016/j.artd.2025.101730	2025	Arthroplasty	Natural Language Processing (NLP)	Prognosis & Prediction
82	10.1016/j.arth.2020.09.055	2021	Trauma; Arthroplasty	Natural Language Processing (NLP); Machine Learning (ML)	Prognosis & Prediction
83	10.1002/ksa.70227	2025	Arthroplasty	Deep Learning (DL)	Diagnosis & Detection
84	10.1002/ksa.12618	2025	Sports Medicine; Trauma	Deep Learning (DL)	Diagnosis & Detection
85	10.1007/s00132-023-04426-x	2023	Pediatric Orthopaedics; Arthroplasty	Machine Learning (ML); Robotics; Deep Learning (DL)	Prognosis & Prediction
86	10.1016/j.arthro.2024.01.017	2024	Sports Medicine; Trauma	Natural Language Processing (NLP)	Prognosis & Prediction
87	10.1016/j.spinee.2020.02.021	2020	Spine Surgery	Natural Language Processing (NLP); Machine Learning (ML)	Prognosis & Prediction
88	10.1016/j.arth.2023.04.007	2023	Arthroplasty	Natural Language Processing (NLP); Machine Learning (ML)	Prognosis & Prediction
89	10.1097/BRS.0000000000005381	2025	Pediatric Orthopaedics; Spine Surgery	Machine Learning (ML); Deep Learning (DL)	Surgical Planning
90	10.1302/0301-620X.104B12	2022	Trauma; Arthroplasty	Unclassified	Prognosis & Prediction
91	10.1002/ksa.12389	2024	Arthroplasty	Machine Learning (ML)	Prognosis & Prediction
92	10.20517/ais.2024.39	2024	Spine Surgery	Machine Learning (ML)	Prognosis & Prediction
93	10.1177/18632521231191687	2023	Pediatric Orthopaedics	Natural Language Processing (NLP)	Unclassified
94	10.1186/s42836-023-00189-0	2023	Arthroplasty	Machine Learning (ML); Deep Learning (DL)	Prognosis & Prediction
95	10.1007/s12178-020-09663-7	2020	Sports Medicine; Arthroplasty	Deep Learning (DL)	Prognosis & Prediction
96	10.1007/s00264-022-05411-3	2023	Arthroplasty	Machine Learning (ML); Robotics	Prognosis & Prediction
97	10.1097/JS9.0000000000002223	2025	Arthroplasty	Natural Language Processing (NLP)	Prognosis & Prediction
98	10.1002/ksa.12369	2025	Sports Medicine; Trauma	Deep Learning (DL)	Diagnosis & Detection
99	10.1097/ANA.0000000000000949	2024	Spine Surgery	Natural Language Processing (NLP)	Unclassified

100	10.1302/0301-620X.106B4.BJJ-2023-1228.R1$2.00	2024	Arthroplasty	Unclassified	Prognosis & Prediction
101	10.2106/JBJS.21.01387	2022	Trauma; Arthroplasty	Deep Learning (DL)	Unclassified
102	10.1016/j.arth.2019.08.017	2019	Arthroplasty	Unclassified	Unclassified
103	10.1007/s00132-025-04687-8	2025	Arthroplasty	Machine Learning (ML)	Prognosis & Prediction
104	10.1016/j.arth.2022.03.033	2022	Sports Medicine; Trauma; Spine Surgery; Pediatric Orthopaedics; Arthroplasty	Natural Language Processing (NLP); Machine Learning (ML); Deep Learning (DL)	Prognosis & Prediction
105	10.1007/s00264-022-05628-2	2023	Arthroplasty	Machine Learning (ML)	Diagnosis & Detection
106	10.1177/15563316251341229	2025	Arthroplasty	Unclassified	Unclassified
107	10.1016/j.wneu.2023.08.042	2023	Spine Surgery	Natural Language Processing (NLP); Machine Learning (ML)	Prognosis & Prediction
108	10.1097/BCO.0000000000001276	2024	Arthroplasty	Natural Language Processing (NLP)	Unclassified
109	10.1007/s43390-021-00360-0	2021	Spine Surgery	Machine Learning (ML)	Prognosis & Prediction
110	10.1186/s42836-022-00119-6	2022	Sports Medicine; Arthroplasty	Machine Learning (ML); Robotics	Prognosis & Prediction
111	10.1002/jor.26109	2025	Sports Medicine; Trauma	Robotics; Deep Learning (DL)	Prognosis & Prediction
112	10.1007/s00167-021-06828-w	2022	Sports Medicine; Trauma	Machine Learning (ML)	Prognosis & Prediction
113	10.1007/s00132-025-04619-6	2025	Arthroplasty	Natural Language Processing (NLP); Machine Learning (ML); Robotics; Deep Learning (DL)	Prognosis & Prediction
114	10.1177/21925682231154543	2024	Pediatric Orthopaedics; Spine Surgery	Deep Learning (DL)	Surgical Planning
115	10.1016/j.arthro.2020.12.234	2021	Arthroplasty	Machine Learning (ML)	Prognosis & Prediction
116	10.1016/j.spinee.2021.03.018	2021	Spine Surgery	Natural Language Processing (NLP); Machine Learning (ML); Robotics; Deep Learning (DL)	Surgical Planning
117	10.1007/s00142-023-00657-4	2024	Spine Surgery	Natural Language Processing (NLP); Machine Learning (ML); Deep Learning (DL)	Prognosis & Prediction

118	10.1007/s00256-023-04340-5	2023	Unclassified	Natural Language Processing (NLP); Machine Learning (ML)	Unclassified
119	10.22603/ssrr.2021-0251	2022	Sports Medicine; Trauma; Spine Surgery; Pediatric Orthopaedics; Arthroplasty	Natural Language Processing (NLP); Machine Learning (ML); Robotics; Deep Learning (DL)	Prognosis & Prediction
120	10.2106/JBJS.24.01530	2025	Sports Medicine; Trauma	Machine Learning (ML); Deep Learning (DL)	Prognosis & Prediction
121	10.1016/j.jpedsurg.2019.03.007	2019	Pediatric Orthopaedics; Trauma; Spine Surgery	Machine Learning (ML)	Prognosis & Prediction
122	10.1177/03635465251322803	2025	Sports Medicine; Trauma; Arthroplasty	Machine Learning (ML)	Prognosis & Prediction
123	10.1007/s11548-023-02880-8	2023	Trauma; Spine Surgery	Machine Learning (ML); Deep Learning (DL)	Prognosis & Prediction
124	10.1016/j.spinee.2021.08.002	2022	Spine Surgery	Natural Language Processing (NLP); Machine Learning (ML)	Prognosis & Prediction
125	10.5312/wjo.v15.i12.1112	2024	Arthroplasty	Machine Learning (ML)	Prognosis & Prediction
126	10.1007/s43390-025-01169-x	2025	Unclassified	Natural Language Processing (NLP)	Prognosis & Prediction
127	10.1097/BSD.0000000000001600	2024	Spine Surgery	Natural Language Processing (NLP)	Unclassified
128	10.1007/s12178-022-09775-2	2022	Sports Medicine; Trauma	Unclassified	Prognosis & Prediction
129	10.1177/21925682251335880	2025	Spine Surgery; Trauma; Arthroplasty	Machine Learning (ML)	Prognosis & Prediction
130	10.1302/2633-1462.310.BJO-2022-0082.R1	2022	Arthroplasty	Machine Learning (ML); Robotics; Deep Learning (DL)	Surgical Planning
131	10.1016/j.spinee.2019.12.006	2020	Spine Surgery	Natural Language Processing (NLP); Machine Learning (ML)	Prognosis & Prediction
132	10.1007/s00167-021-06848-6	2022	Arthroplasty	Machine Learning (ML)	Prognosis & Prediction
133	10.1016/j.jot.2022.07.004	2022	Arthroplasty	Machine Learning (ML); Deep Learning (DL)	Prognosis & Prediction
134	10.1002/jeo2.70459	2025	Sports Medicine; Trauma; Arthroplasty	Unclassified	Prognosis & Prediction
135	10.1177/2192568219878133	2020	Spine Surgery	Machine Learning (ML)	Prognosis & Prediction
136	10.1007/s00132-022-04293-y	2022	Trauma; Spine Surgery	Machine Learning (ML)	Diagnosis & Detection

137	10.1016/j.jhsa.2024.09.008	2025	Trauma	Natural Language Processing (NLP); Machine Learning (ML); Deep Learning (DL)	Prognosis & Prediction
138	10.1007/s11657-025-01542-3	2025	Spine Surgery; Trauma; Arthroplasty	Machine Learning (ML)	Prognosis & Prediction
139	10.2106/JBJS.24.00938	2025	Trauma; Arthroplasty	Unclassified	Unclassified
140	10.1177/19476035251395177	2025	Sports Medicine; Trauma; Arthroplasty	Machine Learning (ML)	Prognosis & Prediction
141	10.1007/s00402-025-06085-3	2025	Arthroplasty	Natural Language Processing (NLP); Robotics	Prognosis & Prediction
142	10.1007/s00586-021-07108-4	2022	Spine Surgery	Natural Language Processing (NLP); Machine Learning (ML)	Prognosis & Prediction
143	10.1097/CORR.00000000000002385	2023	Trauma	Deep Learning (DL)	Diagnosis & Detection
144	10.1016/j.arth.2023.02.055	2023	Arthroplasty	Deep Learning (DL)	Surgical Planning
145	10.1016/j.arth.2021.12.009	2022	Arthroplasty	Deep Learning (DL)	Surgical Planning
146	10.1007/s11701-025-02561-5	2025	Sports Medicine; Trauma; Arthroplasty	Natural Language Processing (NLP); Machine Learning (ML); Robotics; Deep Learning (DL)	Prognosis & Prediction
147	10.1016/j.arthro.2023.07.042	2024	Arthroplasty	Robotics; Deep Learning (DL)	Diagnosis & Detection
148	N/A	2025	Arthroplasty	Deep Learning (DL)	Unclassified
149	10.1080/03008207.2025.2490097	2025	Sports Medicine; Trauma; Arthroplasty	Machine Learning (ML)	Prognosis & Prediction
150	10.14245/ns.1938414.207	2019	Spine Surgery	Machine Learning (ML)	Prognosis & Prediction
151	N/A	2023	Arthroplasty	Deep Learning (DL)	Prognosis & Prediction
152	10.1016/j.arthro.2021.12.030	2022	Sports Medicine; Trauma; Spine Surgery; Pediatric Orthopaedics; Arthroplasty	Machine Learning (ML); Deep Learning (DL)	Prognosis & Prediction
153	10.1016/j.arth.2023.05.056	2023	Arthroplasty	Natural Language Processing (NLP)	Unclassified
154	10.25259/JMSR_209_2025	2025	Sports Medicine; Trauma; Arthroplasty	Deep Learning (DL)	Diagnosis & Detection
155	10.1097/BPO.0000000000002781	2025	Pediatric Orthopaedics; Spine Surgery	Natural Language Processing (NLP)	Prognosis & Prediction
156	10.1007/s00256-021-03802-y	2022	Sports Medicine; Trauma	Deep Learning (DL)	Prognosis & Prediction
157	10.1016/j.xnsj.2025.100599	2025	Spine Surgery	Natural Language Processing (NLP)	Diagnosis & Detection
158	10.1002/jor.24614	2020	Arthroplasty	Unclassified	Prognosis & Prediction

159	10.22603/ssrr.2020-0011	2020	Spine Surgery	Machine Learning (ML); Robotics	Unclassified
160	10.1016/j.xnsj.2023.100236	2023	Trauma; Spine Surgery	Machine Learning (ML); Deep Learning (DL)	Prognosis & Prediction
161	10.1007/s00586-020-06613-2	2021	Spine Surgery	Machine Learning (ML); Deep Learning (DL)	Prognosis & Prediction
162	10.1002/jor.25982	2025	Arthroplasty	Machine Learning (ML)	Prognosis & Prediction
163	10.1007/s00586-025-09548-8	2025	Pediatric Orthopaedics	Machine Learning (ML)	Prognosis & Prediction
164	10.1016/j.spinee.2025.03.009	2025	Spine Surgery	Natural Language Processing (NLP); Deep Learning (DL)	Prognosis & Prediction
165	10.1053/j.jfas.2024.06.009	2024	Trauma	Natural Language Processing (NLP); Machine Learning (ML)	Diagnosis & Detection
166	10.1016/j.wneu.2024.05.103	2024	Trauma; Spine Surgery	Machine Learning (ML); Deep Learning (DL)	Prognosis & Prediction
167	10.1016/j.joca.2019.02.796	2019	Trauma; Arthroplasty	Natural Language Processing (NLP); Machine Learning (ML)	Prognosis & Prediction
168	10.1097/JS9.0000000000002130	2024	Unclassified	Natural Language Processing (NLP)	Unclassified
169	10.1177/18632521251325122	2025	Pediatric Orthopaedics	Deep Learning (DL)	Prognosis & Prediction
170	10.1111/ans.18250	2023	Arthroplasty	Machine Learning (ML)	Prognosis & Prediction
171	10.1016/j.arth.2023.05.058	2023	Arthroplasty	Natural Language Processing (NLP)	Unclassified
172	10.1007/s11548-023-02831-3	2023	Unclassified	Machine Learning (ML); Deep Learning (DL)	Unclassified
173	10.1302/0301-620X.102B7.BJJ-2019-1628.R1	2020	Arthroplasty	Machine Learning (ML)	Prognosis & Prediction
174	10.1002/jor.26066	2025	Sports Medicine; Trauma; Arthroplasty	Machine Learning (ML)	Prognosis & Prediction
175	10.1016/j.knee.2025.04.008	2025	Unclassified	Natural Language Processing (NLP)	Prognosis & Prediction
176	10.1177/21925682241248110	2025	Pediatric Orthopaedics; Spine Surgery	Natural Language Processing (NLP); Machine Learning (ML)	Unclassified
177	10.1007/s11548-021-02552-5	2022	Trauma; Arthroplasty	Deep Learning (DL)	Surgical Planning
178	10.3171/2022.4.SPINE22133	2022	Unclassified	Deep Learning (DL)	Prognosis & Prediction
179	10.1007/s00132-024-04515-5	2024	Spine Surgery; Arthroplasty	Unclassified	Prognosis & Prediction

180	10.1016/j.arth.2019.07.024	2019	Trauma; Arthroplasty	Machine Learning (ML)	Prognosis & Prediction
181	10.1016/j.jse.2023.03.028	2023	Arthroplasty	Deep Learning (DL)	Prognosis & Prediction
182	10.1016/j.knee.2025.06.018	2025	Sports Medicine	Natural Language Processing (NLP)	Unclassified
183	10.1016/j.joca.2021.11.012	2022	Sports Medicine	Unclassified	Prognosis & Prediction
184	10.1097/BCO.0000000000001249	2024	Arthroplasty	Natural Language Processing (NLP); Machine Learning (ML)	Unclassified
185	10.1016/j.arth.2024.10.100	2025	Arthroplasty	Natural Language Processing (NLP); Machine Learning (ML)	Prognosis & Prediction
186	10.1007/s00586-023-07706-4	2023	Spine Surgery	Deep Learning (DL)	Prognosis & Prediction
187	10.1016/j.arth.2023.05.059	2023	Arthroplasty	Natural Language Processing (NLP)	Unclassified
188	10.1016/j.arth.2023.05.057	2023	Arthroplasty	Natural Language Processing (NLP)	Unclassified
189	10.1016/j.knee.2024.08.014	2024	Sports Medicine	Natural Language Processing (NLP)	Unclassified
190	10.1007/s00256-024-04853-7	2025	Pediatric Orthopaedics; Spine Surgery	Deep Learning (DL)	Prognosis & Prediction
191	10.1007/s00590-024-03990-y	2024	Trauma	Natural Language Processing (NLP)	Prognosis & Prediction
192	10.1302/0301-620X.104B9.BJJ-2022-00026	2022	Trauma	Unclassified	Unclassified
193	10.1302/0301-620X.101B12.BJJ-2019-0850.R1	2019	Unclassified	Machine Learning (ML)	Prognosis & Prediction
194	10.1186/s42836-022-00112-z	2022	Arthroplasty	Machine Learning (ML); Robotics; Deep Learning (DL)	Prognosis & Prediction
195	10.1002/ksa.12711	2025	Sports Medicine; Trauma	Natural Language Processing (NLP)	Prognosis & Prediction
196	10.1007/s00586-025-09347-1	2025	Spine Surgery	Natural Language Processing (NLP)	Unclassified
197	10.1007/s11657-020-00859-5	2021	Trauma	Natural Language Processing (NLP)	Prognosis & Prediction
198	10.1097/JS9.0000000000002481	2025	Spine Surgery; Arthroplasty	Natural Language Processing (NLP); Machine Learning (ML); Robotics	Prognosis & Prediction
199	10.1097/BCO.0000000000001266	2024	Trauma; Arthroplasty	Natural Language Processing (NLP); Machine Learning (ML)	Unclassified
200	10.1007/s00264-024-06088-6	2024	Arthroplasty	Machine Learning (ML)	Prognosis & Prediction
201	10.1016/j.joca.2021.12.012	2022	Sports Medicine; Arthroplasty	Machine Learning (ML)	Prognosis & Prediction

202	10.1080/17453674.2021.1918389	2021	Unclassified	Natural Language Processing (NLP); Machine Learning (ML); Deep Learning (DL)	Prognosis & Prediction
203	10.1016/j.jse.2024.12.048	2025	Arthroplasty	Natural Language Processing (NLP)	Prognosis & Prediction
204	10.1016/j.joca.2025.08.002	2025	Sports Medicine; Arthroplasty	Machine Learning (ML)	Prognosis & Prediction
205	10.1177/10225536241243166	2024	Arthroplasty	Robotics; Deep Learning (DL)	Surgical Planning
206	10.1007/s11701-025-02603-y	2025	Sports Medicine; Spine Surgery; Trauma; Arthroplasty	Robotics	Surgical Planning
207	10.1016/j.hansur.2024.101688	2024	Unclassified	Natural Language Processing (NLP)	Unclassified
208	10.1007/s43390-024-00952-6	2025	Pediatric Orthopaedics; Spine Surgery	Machine Learning (ML)	Segmentation & Measurement
209	10.1097/BRS.0000000000003704	2021	Spine Surgery	Machine Learning (ML)	Prognosis & Prediction
210	10.1007/s00167-021-06741-2	2022	Sports Medicine	Machine Learning (ML)	Prognosis & Prediction
211	10.1016/j.jseint.2023.07.018	2023	Unclassified	Natural Language Processing (NLP); Deep Learning (DL)	Diagnosis & Detection
212	10.1016/j.arth.2023.09.025	2024	Arthroplasty	Deep Learning (DL)	Prognosis & Prediction
213	10.1016/j.knee.2024.03.008	2024	Arthroplasty	Deep Learning (DL)	Surgical Planning
214	10.1007/s00264-022-05475-1	2023	Pediatric Orthopaedics; Arthroplasty	Machine Learning (ML)	Prognosis & Prediction
215	10.1186/s13018-022-03408-7	2022	Trauma	Machine Learning (ML); Deep Learning (DL)	Diagnosis & Detection
216	10.1016/j.arthro.2023.12.009	2024	Sports Medicine	Natural Language Processing (NLP)	Unclassified
217	10.1007/s00586-022-07238-3	2022	Spine Surgery; Arthroplasty	Machine Learning (ML)	Prognosis & Prediction
218	N/A	2024	Spine Surgery	Deep Learning (DL)	Unclassified
219	10.1186/s42836-023-00218-y	2023	Arthroplasty	Unclassified	Prognosis & Prediction
220	10.1093/jhps/hnae041	2025	Spine Surgery; Arthroplasty	Machine Learning (ML); Deep Learning (DL)	Prognosis & Prediction
221	10.3389/fsurg.2025.1591756	2025	Trauma; Arthroplasty	Robotics	Prognosis & Prediction

222	10.1016/j.wnsx.2023.100182	2023	Spine Surgery	Natural Language Processing (NLP); Machine Learning (ML); Robotics	Prognosis & Prediction
223	10.2106/JBJS.RVW.23.00232	2024	Trauma; Arthroplasty	Natural Language Processing (NLP); Machine Learning (ML); Deep Learning (DL)	Prognosis & Prediction
224	10.1097/GOX.0000000000006674	2025	Arthroplasty	Unclassified	Segmentation & Measurement
225	10.1097/BCO.0000000000000992	2021	Spine Surgery	Machine Learning (ML)	Diagnosis & Detection
226	10.1007/s00590-025-04238-z	2025	Trauma; Arthroplasty	Unclassified	Prognosis & Prediction
227	10.5435/JAAOSGlobal-D-24-00182	2024	Arthroplasty	Machine Learning (ML)	Prognosis & Prediction
228	N/A	2019	Unclassified	Deep Learning (DL)	Unclassified
229	10.1016/j.jseint.2025.04.024	2025	Arthroplasty	Machine Learning (ML)	Prognosis & Prediction
230	N/A	2021	Arthroplasty	Machine Learning (ML)	Unclassified
231	N/A	2024	Arthroplasty	Machine Learning (ML)	Unclassified
232	10.1097/GOX.0000000000004999	2023	Unclassified	Natural Language Processing (NLP)	Unclassified
233	10.2106/JBJS.OA.23.00056	2023	Unclassified	Natural Language Processing (NLP)	Unclassified
234	10.1186/s13018-024-04602-5	2024	Sports Medicine; Arthroplasty	Machine Learning (ML)	Prognosis & Prediction
235	10.1002/jor.24578	2020	Sports Medicine; Spine Surgery; Pediatric Orthopaedics; Arthroplasty	Machine Learning (ML)	Prognosis & Prediction
236	10.1007/s00586-025-09352-4	2025	Spine Surgery	Natural Language Processing (NLP)	Unclassified
237	10.1055/a-1717-0955	2022	Trauma; Spine Surgery	Natural Language Processing (NLP)	Prognosis & Prediction
238	10.52312/jdrs.2026.2368	2025	Arthroplasty	Natural Language Processing (NLP)	Prognosis & Prediction
239	10.20517/ais.2025.19	2025	Unclassified	Natural Language Processing (NLP); Machine Learning (ML)	Unclassified
240	10.1007/s00132-018-3603-y	2018	Sports Medicine; Trauma	Deep Learning (DL)	Diagnosis & Detection
241	10.1055/a-2194-0970	2024	Sports Medicine; Arthroplasty	Machine Learning (ML); Robotics	Prognosis & Prediction
242	10.1007/978-3-031-55315-8_18	2024	Pediatric Orthopaedics; Spine Surgery	Unclassified	Prognosis & Prediction
243	10.1007/s00590-024-04032-3	2024	Trauma	Natural Language Processing (NLP); Deep Learning (DL)	Prognosis & Prediction

244	10.1016/j.arth.2023.10.009	2024	Arthroplasty	Machine Learning (ML)	Prognosis & Prediction
245	10.1007/s43390-024-00889-w	2024	Pediatric Orthopaedics; Spine Surgery	Machine Learning (ML)	Prognosis & Prediction
246	10.1016/j.wneu.2023.12.124	2024	Spine Surgery	Machine Learning (ML); Robotics	Prognosis & Prediction
247	10.14245/ns.2449186.593	2025	Spine Surgery	Machine Learning (ML)	Prognosis & Prediction
248	10.1016/j.jor.2023.05.012	2023	Arthroplasty	Machine Learning (ML)	Prognosis & Prediction
249	10.1227/neu.0000000000003330	2025	Spine Surgery	Machine Learning (ML)	Prognosis & Prediction
250	10.1007/s00586-023-07718-0	2023	Spine Surgery	Machine Learning (ML); Deep Learning (DL)	Prognosis & Prediction
251	10.1016/j.arth.2019.07.025	2019	Trauma; Arthroplasty	Natural Language Processing (NLP); Machine Learning (ML)	Prognosis & Prediction
252	10.1016/j.artd.2024.101398	2024	Arthroplasty	Deep Learning (DL)	Prognosis & Prediction
253	10.1007/s00264-024-06182-9	2024	Trauma	Natural Language Processing (NLP)	Unclassified
254	10.1016/j.artd.2024.101394	2024	Arthroplasty	Deep Learning (DL)	Prognosis & Prediction
255	10.1016/j.jisako.2023.10.005	2023	Trauma; Arthroplasty	Robotics	Prognosis & Prediction
256	10.1097/CORR.0000000000002741	2023	Unclassified	Natural Language Processing (NLP)	Unclassified
257	10.1097/CORR.0000000000000787	2019	Arthroplasty	Machine Learning (ML)	Prognosis & Prediction
258	N/A	2024	Unclassified	Natural Language Processing (NLP)	Unclassified
259	10.3171/2019.2.SPINE18751	2019	Spine Surgery	Machine Learning (ML)	Prognosis & Prediction
260	10.25259/JMSR_260_2023	2024	Trauma; Pediatric Orthopaedics	Machine Learning (ML); Deep Learning (DL)	Diagnosis & Detection
261	10.1016/j.joca.2018.02.844	2018	Sports Medicine	Unclassified	Diagnosis & Detection
262	10.1016/j.xnsj.2024.100333	2024	Spine Surgery	Natural Language Processing (NLP)	Diagnosis & Detection
263	10.1186/s13018-024-04774-0	2024	Arthroplasty	Natural Language Processing (NLP); Machine Learning (ML)	Prognosis & Prediction
264	10.1016/j.arth.2023.10.008	2024	Arthroplasty	Machine Learning (ML)	Prognosis & Prediction
265	N/A	2024	Arthroplasty	Robotics	Unclassified
266	10.1007/s00256-025-04951-0	2025	Trauma	Unclassified	Prognosis & Prediction

267	10.1302/0301-620X.103B12.BJJ-2021-0851.R1	2021	Trauma	Machine Learning (ML)	Prognosis & Prediction
268	10.1186/s12891-025-09137-2	2025	Sports Medicine; Arthroplasty	Deep Learning (DL)	Diagnosis & Detection
269	10.1097/CORR.0000000000001227	2020	Arthroplasty	Machine Learning (ML)	Prognosis & Prediction
270	10.1302/2046-3758.128.BJR-2023-0199	2023	Arthroplasty	Natural Language Processing (NLP); Machine Learning (ML); Robotics	Surgical Planning
271	10.1097/CORR.0000000000001255	2020	Arthroplasty	Machine Learning (ML)	Prognosis & Prediction
272	10.20517/ais.2024.24	2024	Spine Surgery	Natural Language Processing (NLP)	Prognosis & Prediction
273	10.1186/s12891-022-05742-7	2022	Pediatric Orthopaedics; Spine Surgery	Unclassified	Diagnosis & Detection
274	10.1007/s00586-025-08995-7	2025	Spine Surgery	Deep Learning (DL)	Prognosis & Prediction
275	10.1097/JSM.0000000000001327	2025	Unclassified	Natural Language Processing (NLP)	Prognosis & Prediction
276	10.1007/s00167-021-06838-8	2022	Unclassified	Deep Learning (DL)	Unclassified
277	10.1016/j.joca.2024.10.009	2025	Arthroplasty	Natural Language Processing (NLP); Deep Learning (DL)	Prognosis & Prediction
278	10.1016/j.arth.2022.10.031	2023	Trauma; Arthroplasty	Natural Language Processing (NLP)	Diagnosis & Detection
279	10.1016/j.xnsj.2025.100827	2026	Spine Surgery	Natural Language Processing (NLP); Machine Learning (ML)	Prognosis & Prediction
280	10.1177/03635465211008648	2022	Sports Medicine; Trauma	Machine Learning (ML); Deep Learning (DL)	Prognosis & Prediction
281	10.1016/j.arthro.2023.10.035	2024	Arthroplasty	Natural Language Processing (NLP); Machine Learning (ML); Deep Learning (DL)	Prognosis & Prediction
282	10.31616/asj.2024.0367	2024	Trauma; Pediatric Orthopaedics	Machine Learning (ML); Robotics	Unclassified
283	10.21037/jss-24-156	2024	Spine Surgery	Unclassified	Diagnosis & Detection
284	10.1007/s10103-025-04643-0	2025	Pediatric Orthopaedics	Deep Learning (DL)	Unclassified
285	10.3928/01477447-20230922-05	2024	Unclassified	Natural Language Processing (NLP); Machine Learning (ML)	Segmentation & Measurement
286	10.1016/j.wneu.2020.04.135	2020	Spine Surgery	Machine Learning (ML)	Prognosis & Prediction

287	10.1007/s43465-025-01469-4	2025	Pediatric Orthopaedics	Machine Learning (ML); Robotics	Prognosis & Prediction
288	10.1007/s43390-021-00419-y	2022	Unclassified	Natural Language Processing (NLP); Machine Learning (ML)	Unclassified
289	10.3171/2023.3.FOCUS2386	2023	Spine Surgery	Machine Learning (ML)	Prognosis & Prediction
290	10.2106/JBJS.RVW.21.00142	2022	Unclassified	Machine Learning (ML)	Prognosis & Prediction
291	10.5152/j.aott.2025.24090	2025	Trauma	Natural Language Processing (NLP)	Segmentation & Measurement
292	10.1016/j.artd.2024.101567	2024	Arthroplasty	Machine Learning (ML)	Prognosis & Prediction
293	10.1007/s00586-022-07245-4	2022	Spine Surgery	Machine Learning (ML); Deep Learning (DL)	Segmentation & Measurement
294	10.1302/2633-1462.31.BJO-2021-0123.R1	2022	Unclassified	Unclassified	Prognosis & Prediction
295	10.1007/s43465-023-00998-0	2023	Trauma; Spine Surgery	Unclassified	Prognosis & Prediction
296	10.1530/EOR-24-0016	2024	Unclassified	Deep Learning (DL)	Diagnosis & Detection
297	10.3389/fsurg.2024.1424716	2025	Spine Surgery	Deep Learning (DL)	Diagnosis & Detection
298	10.1177/23259671251324519	2025	Sports Medicine; Trauma	Machine Learning (ML)	Prognosis & Prediction
299	10.1007/s00586-025-08672-9	2025	Spine Surgery	Machine Learning (ML); Deep Learning (DL)	Prognosis & Prediction
300	N/A	2021	Unclassified	Unclassified	Unclassified
301	10.1007/978-3-319-74113-0_10	2018	Trauma; Spine Surgery	Deep Learning (DL)	Segmentation & Measurement
302	10.1186/s12891-024-07777-4	2024	Spine Surgery	Machine Learning (ML); Deep Learning (DL)	Surgical Planning
303	10.1097/CORR.0000000000001226	2020	Arthroplasty	Machine Learning (ML)	Prognosis & Prediction
304	10.1007/s00256-022-04010-y	2022	Trauma; Pediatric Orthopaedics	Machine Learning (ML); Deep Learning (DL)	Unclassified
305	10.2106/JBJS.24.00945	2024	Unclassified	Unclassified	Prognosis & Prediction
306	10.1186/s12891-022-05123-0	2022	Arthroplasty	Machine Learning (ML)	Prognosis & Prediction
307	10.1007/s00132-025-04695-8	2025	Arthroplasty	Robotics	Prognosis & Prediction

308	10.1177/15563316251340983	2025	Sports Medicine; Spine Surgery; Arthroplasty	Natural Language Processing (NLP); Robotics	Unclassified
309	10.1002/ksa.12196	2024	Sports Medicine; Pediatric Orthopaedics	Machine Learning (ML)	Prognosis & Prediction
310	N/A	2024	Unclassified	Natural Language Processing (NLP)	Prognosis & Prediction
311	10.1177/03635465231225215	2024	Sports Medicine; Trauma	Machine Learning (ML)	Prognosis & Prediction
312	10.1007/s13304-025-02106-3	2025	Pediatric Orthopaedics; Spine Surgery	Natural Language Processing (NLP); Machine Learning (ML)	Unclassified
313	10.1016/j.arth.2025.01.032	2025	Arthroplasty	Machine Learning (ML); Deep Learning (DL)	Prognosis & Prediction
314	10.2106/JBJS.ER2.19.00071	2020	Arthroplasty	Natural Language Processing (NLP)	Unclassified
315	10.1007/s11548-019-01933-1	2019	Trauma; Spine Surgery	Machine Learning (ML)	Diagnosis & Detection
316	10.2106/JBJS.ER.19.00071	2020	Arthroplasty	Natural Language Processing (NLP)	Unclassified
317	10.1007/s00586-024-08463-8	2024	Spine Surgery	Machine Learning (ML); Deep Learning (DL)	Diagnosis & Detection
318	10.1007/s00586-024-08375-7	2025	Sports Medicine; Pediatric Orthopaedics; Spine Surgery	Unclassified	Segmentation & Measurement
319	10.1177/21925682251349999	2026	Pediatric Orthopaedics; Spine Surgery	Deep Learning (DL)	Prognosis & Prediction
320	10.1007/s00256-023-04422-4	2023	Sports Medicine; Arthroplasty	Natural Language Processing (NLP)	Prognosis & Prediction
321	10.1007/s11548-024-03237-5	2024	Pediatric Orthopaedics; Spine Surgery	Machine Learning (ML)	Prognosis & Prediction
322	10.23736/S2784-8469.24.04454-7	2024	Arthroplasty	Robotics	Prognosis & Prediction
323	10.1097/CORR.0000000000002777	2023	Arthroplasty	Machine Learning (ML)	Prognosis & Prediction
324	10.1007/s00264-022-05533-8	2023	Trauma; Spine Surgery	Machine Learning (ML)	Diagnosis & Detection
325	10.1097/BRS.0000000000004734	2023	Pediatric Orthopaedics	Machine Learning (ML)	Prognosis & Prediction
326	10.1177/15563316251345479	2025	Trauma	Robotics	Prognosis & Prediction
327	10.1055/s-0040-1713778	2020	Sports Medicine; Arthroplasty	Machine Learning (ML)	Prognosis & Prediction
328	10.1007/978-3-031-55315-8_17	2024	Spine Surgery	Deep Learning (DL)	Diagnosis & Detection

329	10.1007/s00586-025-08906-w	2025	Trauma; Spine Surgery	Machine Learning (ML)	Prognosis & Prediction
330	10.2106/JBJS.RVW.24.00087	2024	Arthroplasty	Machine Learning (ML); Deep Learning (DL)	Prognosis & Prediction
331	10.14444/8644	2024	Spine Surgery	Robotics	Prognosis & Prediction
332	10.1007/s00586-025-09052-z	2025	Spine Surgery	Natural Language Processing (NLP)	Unclassified
333	10.1097/BPO.0000000000002428	2023	Pediatric Orthopaedics; Arthroplasty	Deep Learning (DL)	Prognosis & Prediction
334	10.52312/jdrs.2023.1312	2023	Trauma	Deep Learning (DL)	Diagnosis & Detection
335	10.1177/23259671211014206	2021	Sports Medicine; Trauma	Machine Learning (ML)	Prognosis & Prediction
336	10.1177/17585732241259754	2024	Sports Medicine	Natural Language Processing (NLP)	Unclassified
337	10.1016/j.joca.2023.03.005	2023	Arthroplasty	Natural Language Processing (NLP); Deep Learning (DL)	Prognosis & Prediction
338	10.1016/j.arth.2024.07.033	2025	Arthroplasty	Machine Learning (ML)	Prognosis & Prediction
339	10.1016/j.wneu.2020.05.071	2020	Trauma; Spine Surgery	Machine Learning (ML); Robotics	Prognosis & Prediction
340	10.1002/jor.25010	2021	Trauma	Machine Learning (ML)	Prognosis & Prediction
341	10.1016/j.jisako.2021.12.005	2022	Sports Medicine; Trauma	Machine Learning (ML)	Prognosis & Prediction
342	10.1016/j.otsr.2021.102837	2021	Trauma	Deep Learning (DL)	Prognosis & Prediction
343	10.1002/jor.25325	2023	Arthroplasty	Deep Learning (DL)	Unclassified
344	10.1007/s00256-025-05058-2	2025	Sports Medicine	Deep Learning (DL)	Diagnosis & Detection
345	10.1007/978-3-030-99333-7_16	2022	Spine Surgery	Machine Learning (ML)	Prognosis & Prediction
346	10.1016/j.arth.2022.06.020	2023	Arthroplasty	Machine Learning (ML)	Prognosis & Prediction
347	10.1016/j.arthro.2023.01.093	2023	Unclassified	Machine Learning (ML)	Prognosis & Prediction
348	10.1080/03008207.2025.2490797	2025	Sports Medicine; Trauma; Arthroplasty	Unclassified	Prognosis & Prediction
349	10.3233/BMR-235003	2023	Unclassified	Unclassified	Unclassified
350	10.15366/rimcafd2024.95.024	2024	Sports Medicine	Unclassified	Diagnosis & Detection
351	10.1097/BSD.0000000000001520	2024	Spine Surgery	Machine Learning (ML)	Prognosis & Prediction

352	10.1002/jor.26082	2025	Arthroplasty	Unclassified	Prognosis & Prediction
353	10.1302/2633-1462.43.BJO-2022-0162.R1	2023	Trauma; Arthroplasty	Machine Learning (ML)	Prognosis & Prediction
354	10.5606/ehc.2020.75300	2020	Trauma; Arthroplasty	Robotics; Deep Learning (DL)	Surgical Planning
355	10.14245/ns.1938402.201	2019	Spine Surgery	Machine Learning (ML)	Prognosis & Prediction
356	10.1007/s11548-025-03321-4	2025	Arthroplasty	Deep Learning (DL)	Surgical Planning
357	10.1002/jor.26071	2025	Sports Medicine; Arthroplasty	Deep Learning (DL)	Prognosis & Prediction
358	10.1002/ksa.12627	2025	Arthroplasty	Machine Learning (ML)	Prognosis & Prediction
359	10.14245/ns.2347178.589	2024	Spine Surgery	Machine Learning (ML); Deep Learning (DL)	Surgical Planning
360	10.1007/s00586-016-4426-3	2016	Pediatric Orthopaedics; Spine Surgery	Machine Learning (ML)	Unclassified
361	10.1177/23259671251331067	2025	Sports Medicine; Arthroplasty	Deep Learning (DL)	Prognosis & Prediction
362	10.3389/fsurg.2023.1220181	2023	Spine Surgery	Robotics	Unclassified
363	10.3389/fsurg.2025.1568587	2025	Arthroplasty	Robotics	Unclassified
364	10.1016/j.jot.2023.10.003	2024	Sports Medicine; Arthroplasty	Machine Learning (ML)	Prognosis & Prediction
365	10.1007/s00167-022-07124-x	2023	Sports Medicine; Arthroplasty	Deep Learning (DL)	Diagnosis & Detection
366	10.1007/s00586-021-06866-5	2021	Spine Surgery	Machine Learning (ML)	Prognosis & Prediction
367	10.1007/s00256-023-04355-y	2023	Sports Medicine; Arthroplasty	Unclassified	Diagnosis & Detection
368	10.1007/s40279-022-01698-9	2022	Sports Medicine; Trauma	Machine Learning (ML); Deep Learning (DL)	Prognosis & Prediction
369	10.1530/EOR-2024-0093	2025	Sports Medicine; Trauma	Deep Learning (DL)	Prognosis & Prediction
370	10.1227/neu.0000000000002474	2023	Spine Surgery	Machine Learning (ML)	Prognosis & Prediction
371	10.1007/s00264-023-05875-x	2023	Sports Medicine; Trauma; Arthroplasty	Deep Learning (DL)	Prognosis & Prediction
372	10.1186/s12891-025-08601-3	2025	Unclassified	Natural Language Processing (NLP)	Unclassified
373	10.1055/a-2673-6204	2025	Trauma; Spine Surgery	Unclassified	Prognosis & Prediction
374	10.1016/j.artd.2024.101439	2024	Arthroplasty	Unclassified	Segmentation & Measurement

375	10.1016/j.arthro.2023.08.010	2024	Sports Medicine; Trauma; Arthroplasty	Deep Learning (DL)	Diagnosis & Detection
376	10.1002/jsp2.70120	2025	Pediatric Orthopaedics; Spine Surgery	Deep Learning (DL)	Unclassified
377	10.1002/ksa.12018	2024	Arthroplasty	Unclassified	Prognosis & Prediction
378	10.1007/s00256-023-04343-2	2023	Sports Medicine; Arthroplasty	Unclassified	Unclassified
379	10.1016/j.jor.2024.03.002	2024	Trauma	Machine Learning (ML)	Prognosis & Prediction
380	10.1177/15589447241247246	2025	Unclassified	Natural Language Processing (NLP)	Diagnosis & Detection
381	10.1016/j.jse.2024.10.028	2025	Trauma; Arthroplasty	Natural Language Processing (NLP); Machine Learning (ML)	Prognosis & Prediction
382	N/A	2022	Unclassified	Unclassified	Prognosis & Prediction
383	10.5435/JAAOS-D-23-00586	2023	Arthroplasty	Machine Learning (ML)	Prognosis & Prediction
384	10.1016/j.amjsurg.2023.07.028	2023	Unclassified	Unclassified	Unclassified
385	10.1186/s12891-023-06187-2	2023	Sports Medicine; Arthroplasty	Machine Learning (ML)	Unclassified
386	10.1097/CORR.0000000000001360	2020	Unclassified	Machine Learning (ML)	Prognosis & Prediction
387	10.22038/ABJS.2023.70790.3313	2023	Trauma; Arthroplasty	Unclassified	Prognosis & Prediction
388	10.1016/j.arthro.2024.08.023	2025	Unclassified	Natural Language Processing (NLP); Deep Learning (DL)	Segmentation & Measurement
389	10.1007/s00256-021-03922-5	2022	Unclassified	Unclassified	Unclassified
390	10.1007/s00167-022-06896-6	2022	Sports Medicine	Machine Learning (ML)	Unclassified
391	10.1007/s11657-023-01292-0	2023	Trauma; Spine Surgery	Machine Learning (ML)	Prognosis & Prediction
392	10.1007/s00586-022-07155-5	2022	Spine Surgery	Deep Learning (DL)	Prognosis & Prediction
393	10.1007/s43390-025-01100-4	2025	Unclassified	Natural Language Processing (NLP)	Unclassified
394	10.1002/jso.27874	2025	Unclassified	Natural Language Processing (NLP)	Prognosis & Prediction
395	10.1177/2325967120910447	2020	Sports Medicine; Trauma	Machine Learning (ML)	Prognosis & Prediction
396	10.1016/j.arth.2022.12.007	2023	Arthroplasty	Robotics	Prognosis & Prediction

397	10.1016/j.arthro.2022.12.025	2023	Sports Medicine	Machine Learning (ML)	Prognosis & Prediction
398	10.1007/s11548-024-03203-1	2024	Spine Surgery	Machine Learning (ML)	Prognosis & Prediction
399	10.3389/fspor.2022.994221	2022	Sports Medicine; Trauma	Deep Learning (DL)	Prognosis & Prediction
400	10.1016/j.fcl.2023.04.006	2023	Arthroplasty	Deep Learning (DL)	Segmentation & Measurement
401	10.23736/S2784-8469.24.04517-6	2024	Unclassified	Unclassified	Unclassified
402	N/A	2024	Unclassified	Unclassified	Unclassified
403	10.1002/jeo2.70281	2025	Unclassified	Natural Language Processing (NLP)	Unclassified
404	10.1016/j.joca.2023.11.014	2024	Arthroplasty	Unclassified	Diagnosis & Detection
405	10.1007/s00167-022-07155-4	2022	Unclassified	Machine Learning (ML)	Unclassified
406	10.3171/2025.4.FOCUS25245	2025	Spine Surgery	Machine Learning (ML)	Prognosis & Prediction
407	10.1007/s43390-025-01109-9	2025	Unclassified	Natural Language Processing (NLP)	Unclassified
408	10.2106/JBJS.21.00550	2022	Trauma; Arthroplasty	Machine Learning (ML)	Prognosis & Prediction
409	10.1016/j.wneu.2024.04.117	2024	Spine Surgery	Natural Language Processing (NLP); Machine Learning (ML)	Prognosis & Prediction
410	10.1016/j.arth.2024.04.062	2024	Sports Medicine; Arthroplasty	Machine Learning (ML); Deep Learning (DL)	Prognosis & Prediction
411	10.1016/j.clinbiomech.2025.106673	2025	Sports Medicine; Trauma; Arthroplasty	Machine Learning (ML)	Prognosis & Prediction
412	10.1016/j.jisako.2025.100841	2025	Trauma	Natural Language Processing (NLP)	Unclassified
413	10.1007/s00586-024-08396-2	2024	Spine Surgery; Arthroplasty	Machine Learning (ML)	Prognosis & Prediction
414	10.1016/j.injury.2023.111254	2024	Trauma	Machine Learning (ML)	Prognosis & Prediction
415	10.3171/2025.4.FOCUS25157	2025	Spine Surgery	Machine Learning (ML); Robotics	Prognosis & Prediction
416	10.1177/15563316251341314	2025	Unclassified	Unclassified	Unclassified
417	10.1002/rcs.2468	2023	Spine Surgery	Robotics; Deep Learning (DL)	Unclassified
418	10.1111/sms.14319	2023	Arthroplasty	Deep Learning (DL)	Unclassified

419	10.1097/SCS.0000000000010686	2025	Arthroplasty	Natural Language Processing (NLP)	Prognosis & Prediction
420	10.3389/fspor.2020.576655	2020	Sports Medicine; Trauma; Arthroplasty	Machine Learning (ML)	Prognosis & Prediction
421	10.1007/s43390-025-01179-9	2025	Unclassified	Natural Language Processing (NLP)	Unclassified
422	10.1111/os.70182	2025	Spine Surgery	Machine Learning (ML); Deep Learning (DL)	Diagnosis & Detection
423	10.1186/s13018-023-04446-5	2023	Trauma	Unclassified	Prognosis & Prediction
424	10.1302/2633-1462.63.BJO-2024-0081.R1	2025	Spine Surgery; Trauma; Arthroplasty	Natural Language Processing (NLP)	Prognosis & Prediction
425	N/A	2022	Unclassified	Unclassified	Unclassified
426	N/A	2025	Unclassified	Unclassified	Unclassified
427	10.1016/j.hansur.2024.101757	2024	Unclassified	Natural Language Processing (NLP)	Prognosis & Prediction
428	10.1007/s00586-025-08948-0	2025	Trauma; Spine Surgery	Machine Learning (ML); Deep Learning (DL)	Prognosis & Prediction
429	10.1016/j.jse.2024.04.021	2025	Unclassified	Natural Language Processing (NLP)	Diagnosis & Detection
430	10.1002/jeo2.70181	2025	Sports Medicine	Machine Learning (ML); Deep Learning (DL)	Prognosis & Prediction
431	10.1016/j.jse.2023.04.022	2023	Arthroplasty	Machine Learning (ML); Robotics	Prognosis & Prediction
432	10.1007/s43390-021-00433-0	2022	Unclassified	Natural Language Processing (NLP)	Prognosis & Prediction
433	10.1016/j.spinee.2021.06.012	2021	Spine Surgery	Unclassified	Unclassified
434	10.1007/s11548-025-03459-1	2025	Arthroplasty	Deep Learning (DL)	Prognosis & Prediction
435	N/A	2022	Unclassified	Unclassified	Unclassified
436	10.1177/21925682211008424	2022	Spine Surgery	Machine Learning (ML)	Prognosis & Prediction
437	10.1302/0301-620X.106B1.BJJ-2023-1319$2.00	2024	Arthroplasty	Unclassified	Prognosis & Prediction
438	10.3340/jkns.2019.0084	2020	Spine Surgery	Deep Learning (DL)	Unclassified
439	10.1007/s00586-023-07552-4	2023	Spine Surgery	Natural Language Processing (NLP); Machine Learning (ML)	Prognosis & Prediction

440	10.1007/s00142-024-00667-w	2024	Arthroplasty	Machine Learning (ML)	Prognosis & Prediction
441	10.1186/s40798-024-00786-6	2024	Trauma	Machine Learning (ML)	Prognosis & Prediction
442	10.1007/s00167-022-06866-y	2022	Arthroplasty	Machine Learning (ML)	Prognosis & Prediction
443	10.1002/jor.25797	2024	Spine Surgery; Arthroplasty	Machine Learning (ML)	Segmentation & Measurement
444	10.1016/j.oto.2024.101153	2024	Arthroplasty	Machine Learning (ML); Robotics	Prognosis & Prediction
445	10.1186/s13018-022-03247-6	2022	Sports Medicine; Arthroplasty	Machine Learning (ML)	Diagnosis & Detection
446	10.5435/JAAOS-D-23-00474	2024	Unclassified	Natural Language Processing (NLP)	Unclassified
447	10.1186/s13018-023-03718-4	2023	Unclassified	Natural Language Processing (NLP); Machine Learning (ML)	Surgical Planning
448	10.1016/j.asjsur.2024.01.133	2024	Arthroplasty	Unclassified	Prognosis & Prediction
449	10.14245/ns.1938410.205	2019	Spine Surgery	Unclassified	Unclassified
450	10.1227/neu.0000000000002568	2023	Spine Surgery	Natural Language Processing (NLP)	Prognosis & Prediction
451	10.1177/17585732241259165	2024	Arthroplasty	Robotics	Prognosis & Prediction
452	10.5435/JAAOS-D-24-01057	2025	Spine Surgery	Natural Language Processing (NLP); Deep Learning (DL)	Unclassified
453	N/A	2021	Unclassified	Unclassified	Unclassified
454	10.1097/BPO.0000000000002890	2025	Trauma; Pediatric Orthopaedics	Natural Language Processing (NLP)	Unclassified
455	10.1016/j.jor.2023.11.063	2024	Unclassified	Natural Language Processing (NLP)	Surgical Planning
456	10.1016/j.arth.2020.12.043	2021	Arthroplasty	Unclassified	Unclassified
457	10.1177/21925682251360655	2025	Spine Surgery	Natural Language Processing (NLP)	Surgical Planning
458	10.1007/s00264-023-05722-z	2023	Arthroplasty	Unclassified	Diagnosis & Detection
459	10.1016/j.arth.2019.08.001	2019	Arthroplasty	Machine Learning (ML)	Prognosis & Prediction
460	10.1007/s43390-025-01182-0	2025	Unclassified	Natural Language Processing (NLP)	Unclassified

461	10.2106/JBJS.OA.24.00100	2024	Arthroplasty	Machine Learning (ML); Deep Learning (DL)	Prognosis & Prediction
462	10.1093/ptj/pzab041	2021	Spine Surgery	Unclassified	Prognosis & Prediction
463	10.1016/j.arth.2023.03.031	2023	Trauma; Arthroplasty	Natural Language Processing (NLP)	Prognosis & Prediction
464	10.1007/s00256-024-04626-2	2024	Trauma; Spine Surgery	Unclassified	Diagnosis & Detection
465	10.1007/s00167-023-07459-z	2023	Arthroplasty	Unclassified	Prognosis & Prediction
466	10.1186/s13018-023-04367-3	2023	Sports Medicine; Trauma	Unclassified	Prognosis & Prediction
467	10.1097/JS9.0000000000003076	2025	Arthroplasty	Machine Learning (ML)	Prognosis & Prediction
468	N/A	2024	Unclassified	Unclassified	Unclassified
469	10.1097/BRS.0000000000004598	2023	Trauma; Spine Surgery	Natural Language Processing (NLP); Machine Learning (ML)	Prognosis & Prediction
470	10.1177/21925682251357015	2025	Spine Surgery	Unclassified	Unclassified
471	10.1016/j.arth.2020.12.046	2021	Arthroplasty	Unclassified	Unclassified
472	10.1177/23259671241261695	2024	Sports Medicine; Trauma	Machine Learning (ML)	Prognosis & Prediction
473	10.1016/j.spinee.2023.06.399	2023	Spine Surgery	Natural Language Processing (NLP); Deep Learning (DL)	Prognosis & Prediction
474	10.3171/2025.1.SPINE24900	2025	Sports Medicine; Spine Surgery	Unclassified	Prognosis & Prediction
475	10.1016/j.jisako.2024.100348	2025	Sports Medicine; Arthroplasty	Unclassified	Prognosis & Prediction
476	10.1002/jeo2.70220	2025	Arthroplasty	Robotics; Deep Learning (DL)	Surgical Planning
477	10.1016/j.arthro.2023.12.027	2024	Unclassified	Machine Learning (ML)	Prognosis & Prediction
478	10.1016/j.arth.2024.09.001	2025	Arthroplasty	Natural Language Processing (NLP); Robotics	Prognosis & Prediction
479	10.1055/a-2621-3626	2025	Trauma	Machine Learning (ML)	Prognosis & Prediction
480	10.2519/jospt.2022.10486	2022	Unclassified	Natural Language Processing (NLP)	Unclassified
481	N/A	2019	Spine Surgery	Unclassified	Unclassified
482	10.4103/isj.isj_50_23	2023	Spine Surgery	Robotics	Unclassified
483	10.1016/j.arthro.2022.05.010	2022	Unclassified	Machine Learning (ML)	Prognosis & Prediction

484	N/A	2023	Unclassified	Unclassified	Unclassified
485	10.1177/15533506241299886	2025	Unclassified	Deep Learning (DL)	Prognosis & Prediction
486	10.1093/bjs/znac242.098	2022	Arthroplasty	Unclassified	Unclassified
487	10.14245/ns.2550308.154	2025	Spine Surgery	Unclassified	Unclassified
488	10.1016/j.artd.2021.12.004	2022	Arthroplasty	Machine Learning (ML)	Prognosis & Prediction
489	10.1002/ksa.70168	2025	Trauma; Arthroplasty	Robotics	Prognosis & Prediction
490	10.1177/21925682221085535	2023	Spine Surgery	Machine Learning (ML)	Prognosis & Prediction
491	10.1007/s00586-022-07316-6	2022	Spine Surgery	Unclassified	Unclassified
492	10.1302/0301-620X.106B11	2024	Arthroplasty	Unclassified	Prognosis & Prediction
493	10.2106/JBJS.23.00043	2023	Spine Surgery	Machine Learning (ML)	Unclassified
494	10.1016/j.spinee.2023.09.011	2024	Pediatric Orthopaedics; Spine Surgery	Natural Language Processing (NLP); Deep Learning (DL)	Prognosis & Prediction
495	10.1016/j.joca.2018.12.009	2019	Sports Medicine; Arthroplasty	Unclassified	Unclassified
496	N/A	2025	Unclassified	Unclassified	Prognosis & Prediction
497	N/A	2024	Pediatric Orthopaedics; Trauma; Spine Surgery	Unclassified	Unclassified
498	10.1007/s00586-024-08315-5	2024	Spine Surgery	Natural Language Processing (NLP); Machine Learning (ML)	Prognosis & Prediction
499	10.1007/s11701-025-02772-w	2025	Spine Surgery	Robotics	Prognosis & Prediction
500	10.1007/s43390-020-00051-2	2020	Sports Medicine; Pediatric Orthopaedics; Spine Surgery	Machine Learning (ML)	Surgical Planning
501	10.1016/j.xnsj.2025.100825	2025	Spine Surgery	Robotics; Deep Learning (DL)	Prognosis & Prediction
502	10.1016/j.arth.2023.03.006	2023	Arthroplasty	Deep Learning (DL)	Surgical Planning
503	10.52312/jdrs.2025.1961	2025	Sports Medicine	Natural Language Processing (NLP)	Unclassified
504	10.1016/j.joca.2019.09.005	2020	Arthroplasty	Unclassified	Prognosis & Prediction
505	10.1016/j.injury.2024.111607	2024	Trauma	Machine Learning (ML)	Prognosis & Prediction
506	10.1186/s42836-020-00053-5	2020	Arthroplasty	Unclassified	Diagnosis & Detection
507	10.1186/s13741-025-00531-x	2025	Unclassified	Deep Learning (DL)	Prognosis & Prediction

508	10.1016/j.spinee.2023.07.015	2023	Spine Surgery	Natural Language Processing (NLP)	Prognosis & Prediction
509	10.1007/s00167-022-06957-w	2023	Arthroplasty	Natural Language Processing (NLP); Machine Learning (ML)	Prognosis & Prediction
510	10.1186/s13037-024-00404-0	2024	Trauma	Machine Learning (ML)	Prognosis & Prediction
511	10.14245/ns.2347158.579	2024	Trauma; Spine Surgery	Machine Learning (ML); Deep Learning (DL)	Surgical Planning
512	10.1097/CORR.0000000000001415	2020	Unclassified	Unclassified	Prognosis & Prediction
513	10.1007/s00132-024-04516-4	2024	Arthroplasty	Unclassified	Surgical Planning
514	10.1002/jor.25213	2022	Pediatric Orthopaedics; Spine Surgery	Deep Learning (DL)	Surgical Planning
515	10.1016/j.otsr.2024.103944	2024	Spine Surgery; Arthroplasty	Machine Learning (ML)	Prognosis & Prediction
516	10.1177/23259671241282316	2024	Sports Medicine; Trauma	Machine Learning (ML)	Prognosis & Prediction
517	10.1080/03008207.2025.2538562	2025	Arthroplasty	Unclassified	Prognosis & Prediction
518	10.3389/fspor.2021.809898	2022	Sports Medicine; Trauma; Arthroplasty	Machine Learning (ML)	Prognosis & Prediction
519	10.3171/2021.8.FOCUS21386	2021	Spine Surgery	Machine Learning (ML)	Unclassified
520	10.1007/s00256-024-04796-z	2025	Sports Medicine; Spine Surgery	Deep Learning (DL)	Segmentation & Measurement
521	10.1080/24699322.2023.2276055	2024	Arthroplasty	Machine Learning (ML)	Segmentation & Measurement
522	10.1016/j.gaitpost.2024.09.001	2024	Arthroplasty	Machine Learning (ML)	Prognosis & Prediction
523	10.2106/JBJS.21.00113	2022	Sports Medicine; Trauma	Machine Learning (ML)	Prognosis & Prediction
524	10.1097/GOX.0000000000005822	2024	Arthroplasty	Natural Language Processing (NLP)	Prognosis & Prediction
525	10.1007/s00256-024-04651-1	2024	Unclassified	Natural Language Processing (NLP)	Unclassified
526	10.1080/14763141.2025.2528885	2025	Sports Medicine; Arthroplasty	Machine Learning (ML)	Prognosis & Prediction
527	10.14245/ns.1938438.219	2019	Spine Surgery	Unclassified	Unclassified
528	10.3171/2024.1.SPINE231252	2024	Sports Medicine; Spine Surgery	Unclassified	Prognosis & Prediction

529	10.1016/j.spinee.2024.03.019	2024	Pediatric Orthopaedics; Spine Surgery	Natural Language Processing (NLP); Deep Learning (DL)	Prognosis & Prediction
530	10.1177/23259671251319512	2025	Sports Medicine; Trauma	Machine Learning (ML)	Prognosis & Prediction
531	10.1016/j.knee.2025.02.009	2025	Sports Medicine; Trauma	Deep Learning (DL)	Segmentation & Measurement
532	10.1186/s43019-024-00246-1	2024	Sports Medicine	Deep Learning (DL)	Segmentation & Measurement
533	10.21037/jss-25-47	2025	Spine Surgery; Arthroplasty	Natural Language Processing (NLP)	Unclassified
534	10.1177/15563316231164138	2024	Spine Surgery	Machine Learning (ML)	Prognosis & Prediction
535	10.1186/s13018-025-05900-2	2025	Pediatric Orthopaedics	Machine Learning (ML)	Prognosis & Prediction
536	10.1016/j.wneu.2024.04.091	2024	Sports Medicine; Spine Surgery	Deep Learning (DL)	Diagnosis & Detection
537	10.1177/21925682241272348	2024	Spine Surgery	Unclassified	Unclassified
538	10.1186/s12957-025-03989-7	2025	Arthroplasty	Machine Learning (ML); Deep Learning (DL)	Prognosis & Prediction
539	10.1016/j.arth.2022.12.013	2023	Arthroplasty	Deep Learning (DL)	Prognosis & Prediction
540	10.1016/j.jsurg.2024.08.002	2024	Unclassified	Natural Language Processing (NLP)	Segmentation & Measurement
541	10.1007/s43465-025-01504-4	2025	Trauma; Spine Surgery	Unclassified	Prognosis & Prediction
542	10.1016/j.jseint.2023.09.009	2024	Unclassified	Natural Language Processing (NLP)	Prognosis & Prediction
543	N/A	2024	Unclassified	Unclassified	Unclassified
544	10.1097/PRS.0000000000011340	2024	Arthroplasty	Unclassified	Prognosis & Prediction
545	10.1055/s-0041-1725962	2021	Trauma	Machine Learning (ML); Deep Learning (DL)	Prognosis & Prediction
546	10.1007/s00256-025-04922-5	2025	Sports Medicine	Natural Language Processing (NLP)	Diagnosis & Detection
547	10.1055/a-2743-4868	2025	Trauma; Pediatric Orthopaedics	Deep Learning (DL)	Prognosis & Prediction
548	10.1097/CORR.0000000000000848	2019	Trauma	Unclassified	Prognosis & Prediction
549	10.1016/j.joca.2022.10.001	2023	Unclassified	Machine Learning (ML); Deep Learning (DL)	Prognosis & Prediction

550	10.1007/s00402-021-04041-5	2021	Arthroplasty	Machine Learning (ML)	Prognosis & Prediction
551	10.1186/s12891-025-09345-w	2025	Pediatric Orthopaedics	Machine Learning (ML)	Prognosis & Prediction
552	10.1080/17453674.2021.1884408	2021	Unclassified	Machine Learning (ML)	Prognosis & Prediction
553	10.1007/s00381-023-06019-7	2023	Pediatric Orthopaedics; Spine Surgery	Natural Language Processing (NLP)	Unclassified
554	10.1016/j.jse.2025.02.025	2025	Unclassified	Natural Language Processing (NLP)	Unclassified
555	10.1007/s00167-023-07564-z	2023	Arthroplasty	Unclassified	Unclassified
556	10.1302/0301-620X.105B3.BJJ-2023-0145	2023	Arthroplasty	Unclassified	Prognosis & Prediction
557	10.1007/s00256-024-04633-3	2024	Unclassified	Natural Language Processing (NLP)	Unclassified
558	10.1002/jor.25789	2024	Trauma; Spine Surgery	Machine Learning (ML)	Prognosis & Prediction
559	10.1007/s00586-023-08109-1	2024	Spine Surgery	Deep Learning (DL)	Surgical Planning
560	10.1007/s00264-022-05311-6	2022	Arthroplasty	Unclassified	Unclassified
561	10.1016/j.arth.2023.08.045	2023	Unclassified	Machine Learning (ML)	Prognosis & Prediction
562	10.1097/BRS.0000000000004909	2024	Spine Surgery; Arthroplasty	Deep Learning (DL)	Prognosis & Prediction
563	10.1186/s12891-021-04403-5	2021	Trauma; Spine Surgery	Machine Learning (ML)	Prognosis & Prediction
564	10.1080/03008207.2025.2541291	2025	Sports Medicine; Arthroplasty	Unclassified	Unclassified
565	N/A	2020	Sports Medicine	Machine Learning (ML)	Prognosis & Prediction
566	10.1177/21925682241296481	2025	Spine Surgery	Machine Learning (ML)	Prognosis & Prediction
567	10.3389/fsurg.2024.1533526	2024	Spine Surgery	Unclassified	Unclassified
568	10.1097/BRS.0000000000005388	2025	Spine Surgery	Machine Learning (ML)	Prognosis & Prediction
569	10.1007/s00586-023-07923-x	2023	Spine Surgery	Machine Learning (ML)	Prognosis & Prediction
570	10.1007/s00256-025-04878-6	2025	Arthroplasty	Natural Language Processing (NLP); Deep Learning (DL)	Prognosis & Prediction

571	10.1053/j.jfas.2024.05.005	2024	Trauma; Arthroplasty	Natural Language Processing (NLP); Machine Learning (ML)	Prognosis & Prediction
572	10.1016/j.jor.2025.08.038	2026	Trauma; Arthroplasty	Machine Learning (ML)	Prognosis & Prediction
573	10.1055/a-2302-6912	2024	Trauma	Natural Language Processing (NLP)	Prognosis & Prediction
574	10.2106/JBJS.25.00703	2025	Sports Medicine	Unclassified	Prognosis & Prediction
575	10.1186/s12891-024-07441-x	2024	Trauma; Arthroplasty	Machine Learning (ML)	Prognosis & Prediction
576	10.1186/s13018-024-04666-3	2024	Trauma	Robotics	Unclassified
577	10.1177/23259671231206180	2023	Spine Surgery	Machine Learning (ML)	Prognosis & Prediction
578	10.3389/fsurg.2021.606038	2021	Arthroplasty	Machine Learning (ML)	Prognosis & Prediction
579	10.1186/s42836-021-00109-0	2022	Arthroplasty	Robotics	Unclassified
580	10.1097/BRS.0000000000002974	2019	Unclassified	Unclassified	Prognosis & Prediction
581	10.1097/BSD.0000000000001372	2023	Spine Surgery	Natural Language Processing (NLP); Machine Learning (ML)	Prognosis & Prediction
582	10.1093/neuros/nyy469	2019	Spine Surgery	Machine Learning (ML)	Prognosis & Prediction
583	10.1097/CORR.0000000000003494	2025	Arthroplasty	Unclassified	Prognosis & Prediction
584	10.1016/j.spinee.2025.05.026	2025	Spine Surgery	Natural Language Processing (NLP)	Prognosis & Prediction
585	10.5152/j.aott.2024.23215	2024	Spine Surgery	Natural Language Processing (NLP)	Prognosis & Prediction
586	10.1002/jeo2.70383	2025	Sports Medicine; Arthroplasty	Deep Learning (DL)	Surgical Planning
587	10.1016/j.arth.2023.02.048	2023	Arthroplasty	Unclassified	Prognosis & Prediction
588	10.1016/j.surge.2023.12.005	2024	Unclassified	Natural Language Processing (NLP)	Unclassified
589	10.1007/s00256-024-04636-0	2024	Unclassified	Natural Language Processing (NLP)	Unclassified
590	10.1016/j.jcms.2025.10.010	2025	Sports Medicine; Trauma	Unclassified	Prognosis & Prediction
591	10.1007/s00256-025-05033-x	2026	Trauma	Deep Learning (DL)	Prognosis & Prediction
592	10.1007/s00167-022-07225-7	2023	Trauma; Arthroplasty	Machine Learning (ML)	Prognosis & Prediction
593	10.1016/j.ocarto.2025.100566	2025	Arthroplasty	Machine Learning (ML)	Prognosis & Prediction

594	10.1002/ohn.472	2023	Unclassified	Machine Learning (ML)	Prognosis & Prediction
595	10.1016/j.joca.2019.11.009	2020	Sports Medicine	Unclassified	Unclassified
596	10.1016/j.jhsa.2022.02.023	2022	Trauma	Machine Learning (ML)	Prognosis & Prediction
597	10.1002/ksa.70039	2025	Arthroplasty	Unclassified	Prognosis & Prediction
598	10.1097/BSD.0000000000001709	2024	Trauma; Spine Surgery	Natural Language Processing (NLP); Machine Learning (ML); Robotics	Prognosis & Prediction
599	10.1016/j.asjsur.2023.05.107	2023	Arthroplasty	Machine Learning (ML); Robotics	Prognosis & Prediction
600	10.1016/j.joca.2019.04.005	2019	Unclassified	Unclassified	Unclassified
601	N/A	2021	Unclassified	Machine Learning (ML)	Prognosis & Prediction
602	10.1016/j.joca.2023.11.015	2024	Sports Medicine; Arthroplasty	Machine Learning (ML)	Unclassified
603	10.1007/s43465-021-00529-9	2021	Arthroplasty	Deep Learning (DL)	Prognosis & Prediction
604	10.1016/j.ocarto.2023.100344	2023	Arthroplasty	Natural Language Processing (NLP); Machine Learning (ML)	Prognosis & Prediction
605	10.1007/s00256-021-03782-z	2022	Unclassified	Unclassified	Prognosis & Prediction
606	10.1007/s00586-025-08708-0	2025	Trauma; Spine Surgery	Deep Learning (DL)	Prognosis & Prediction
607	10.1016/j.jse.2023.11.005	2024	Arthroplasty	Machine Learning (ML)	Prognosis & Prediction
608	10.1097/SCS.0000000000009597	2023	Trauma	Robotics; Deep Learning (DL)	Prognosis & Prediction
609	N/A	2023	Sports Medicine	Deep Learning (DL)	Unclassified
610	10.1177/23259671251357595	2025	Sports Medicine	Deep Learning (DL)	Segmentation & Measurement
611	10.1002/jor.25334	2023	Trauma	Machine Learning (ML)	Prognosis & Prediction
612	10.1016/j.ocarto.2024.100458	2024	Arthroplasty	Natural Language Processing (NLP)	Prognosis & Prediction
613	10.1016/j.ijsu.2021.106183	2021	Arthroplasty	Machine Learning (ML)	Prognosis & Prediction
614	10.1016/j.jseint.2025.04.013	2025	Unclassified	Natural Language Processing (NLP)	Prognosis & Prediction
615	10.1002/hed.27519	2023	Arthroplasty	Machine Learning (ML)	Prognosis & Prediction
616	N/A	2022	Unclassified	Machine Learning (ML)	Prognosis & Prediction
617	10.1016/j.jsams.2021.03.016	2021	Sports Medicine; Trauma; Pediatric Orthopaedics	Machine Learning (ML)	Prognosis & Prediction
618	10.1016/j.spinee.2024.04.028	2024	Sports Medicine; Trauma; Spine Surgery	Natural Language Processing (NLP); Machine Learning (ML)	Prognosis & Prediction

619	10.1152/japplphysiol.00502.2024	2025	Spine Surgery	Unclassified	Segmentation & Measurement
620	10.1302/2633-1462.311.BJO-2022-0125.R1	2022	Unclassified	Deep Learning (DL)	Segmentation & Measurement
621	10.1007/s00586-023-07535-5	2023	Spine Surgery	Unclassified	Surgical Planning
622	10.1007/s00256-025-05106-x	2025	Sports Medicine; Trauma	Deep Learning (DL)	Diagnosis & Detection
623	10.1007/s00256-022-04268-2	2023	Spine Surgery	Deep Learning (DL)	Unclassified
624	N/A	2022	Unclassified	Machine Learning (ML)	Prognosis & Prediction
625	10.1016/j.arthro.2025.03.042	2025	Arthroplasty	Natural Language Processing (NLP)	Unclassified
626	10.1186/s42836-023-00213-3	2023	Spine Surgery; Arthroplasty	Robotics	Prognosis & Prediction
627	10.31616/asj.2018.0243	2019	Spine Surgery	Machine Learning (ML)	Prognosis & Prediction
628	10.1016/j.artd.2025.101794	2025	Arthroplasty	Natural Language Processing (NLP)	Unclassified
629	10.1097/CORR.0000000000000687	2019	Arthroplasty	Machine Learning (ML)	Prognosis & Prediction
630	10.1002/jor.23519	2017	Sports Medicine	Machine Learning (ML)	Prognosis & Prediction
631	10.1016/j.ocarto.2025.100702	2026	Sports Medicine	Deep Learning (DL)	Segmentation & Measurement
632	10.1002/jor.25707	2024	Arthroplasty	Deep Learning (DL)	Unclassified
633	10.1016/j.xnsj.2023.100214	2023	Spine Surgery	Natural Language Processing (NLP); Machine Learning (ML)	Prognosis & Prediction
634	N/A	2019	Arthroplasty	Machine Learning (ML)	Prognosis & Prediction
635	10.1016/j.clinbiomech.2021.105452	2021	Arthroplasty	Machine Learning (ML)	Prognosis & Prediction
636	10.1177/21925682231204159	2025	Trauma; Spine Surgery	Machine Learning (ML); Deep Learning (DL)	Prognosis & Prediction
637	N/A	2018	Unclassified	Machine Learning (ML)	Prognosis & Prediction
638	10.1016/j.afos.2025.10.003	2025	Trauma	Deep Learning (DL)	Prognosis & Prediction
639	10.1016/j.joca.2020.08.005	2020	Sports Medicine; Arthroplasty	Machine Learning (ML)	Segmentation & Measurement
640	N/A	2022	Arthroplasty	Machine Learning (ML); Deep Learning (DL)	Unclassified

641	10.1227/neu.0000000000001989	2022	Spine Surgery	Machine Learning (ML)	Prognosis & Prediction
642	N/A	2024	Unclassified	Machine Learning (ML)	Prognosis & Prediction
643	10.1016/j.wneu.2024.01.043	2024	Spine Surgery	Natural Language Processing (NLP)	Unclassified
644	10.1016/j.ocarto.2024.100551	2025	Unclassified	Deep Learning (DL)	Prognosis & Prediction
645	10.1016/j.ocl.2025.02.002	2025	Arthroplasty	Unclassified	Prognosis & Prediction
646	10.1016/j.joca.2022.10.015	2023	Sports Medicine	Unclassified	Prognosis & Prediction
647	10.1186/s43019-025-00287-0	2025	Sports Medicine; Arthroplasty	Robotics	Prognosis & Prediction
648	N/A	2024	Sports Medicine; Arthroplasty	Machine Learning (ML)	Prognosis & Prediction
649	10.1007/s00586-022-07309-5	2022	Spine Surgery	Deep Learning (DL)	Surgical Planning
650	10.1007/s00256-025-04969-4	2025	Sports Medicine; Pediatric Orthopaedics	Deep Learning (DL)	Diagnosis & Detection
651	10.1016/j.knee.2021.08.029	2021	Sports Medicine; Trauma; Arthroplasty	Natural Language Processing (NLP); Machine Learning (ML)	Prognosis & Prediction
652	10.1016/j.joca.2019.02.398	2019	Unclassified	Deep Learning (DL)	Prognosis & Prediction
653	10.1016/j.knee.2025.02.013	2025	Arthroplasty	Machine Learning (ML)	Surgical Planning
654	10.1097/CORR.0000000000001263	2020	Trauma; Arthroplasty	Machine Learning (ML)	Prognosis & Prediction
655	10.1016/j.spinee.2024.04.016	2024	Trauma; Spine Surgery	Machine Learning (ML)	Prognosis & Prediction
656	10.20517/ais.2024.43	2025	Spine Surgery	Machine Learning (ML)	Prognosis & Prediction
657	10.1016/j.arth.2019.05.061	2019	Arthroplasty	Machine Learning (ML)	Prognosis & Prediction
658	10.1016/j.surge.2023.07.001	2023	Unclassified	Natural Language Processing (NLP)	Prognosis & Prediction
659	N/A	2025	Unclassified	Machine Learning (ML)	Unclassified
660	10.3389/fsurg.2023.1271775	2023	Spine Surgery	Natural Language Processing (NLP)	Prognosis & Prediction
661	10.1097/JS9.0000000000002390	2025	Arthroplasty	Natural Language Processing (NLP)	Unclassified
662	10.1016/j.joca.2024.01.001	2024	Sports Medicine; Arthroplasty	Unclassified	Diagnosis & Detection
663	10.1007/s11548-024-03284-y	2025	Arthroplasty	Unclassified	Prognosis & Prediction
664	10.1007/s11548-021-02444-8	2021	Unclassified	Robotics; Deep Learning (DL)	Unclassified

665	10.1016/j.joca.2019.02.396	2019	Unclassified	Deep Learning (DL)	Prognosis & Prediction
666	10.1016/j.jse.2022.07.013	2022	Trauma; Arthroplasty	Machine Learning (ML)	Prognosis & Prediction
667	10.1007/s00586-025-09106-2	2025	Sports Medicine; Pediatric Orthopaedics; Spine Surgery	Deep Learning (DL)	Prognosis & Prediction
668	10.1177/10225536251368569	2025	Arthroplasty	Natural Language Processing (NLP)	Unclassified
669	10.1016/j.jht.2025.04.009	2025	Trauma; Arthroplasty	Natural Language Processing (NLP); Robotics	Prognosis & Prediction
670	10.1016/j.xnsj.2025.100777	2025	Trauma; Spine Surgery	Machine Learning (ML)	Prognosis & Prediction
671	10.1016/j.joca.2018.02.651	2018	Unclassified	Machine Learning (ML)	Prognosis & Prediction
672	10.1186/s13018-025-05855-4	2025	Arthroplasty	Machine Learning (ML)	Prognosis & Prediction
673	10.14245/ns.2346610.305	2023	Spine Surgery	Robotics	Surgical Planning
674	N/A	2021	Unclassified	Deep Learning (DL)	Prognosis & Prediction
675	10.1016/j.arth.2020.07.026	2021	Trauma; Arthroplasty	Machine Learning (ML)	Prognosis & Prediction
676	10.62713/aic.3625	2024	Trauma	Machine Learning (ML)	Prognosis & Prediction
677	N/A	2020	Unclassified	Deep Learning (DL)	Prognosis & Prediction
678	N/A	2023	Unclassified	Machine Learning (ML)	Unclassified
679	10.1007/s00586-024-08172-2	2024	Spine Surgery	Machine Learning (ML)	Prognosis & Prediction
680	10.1007/s00586-025-08847-4	2025	Spine Surgery; Arthroplasty	Unclassified	Prognosis & Prediction
681	10.1002/jor.24660	2020	Trauma	Machine Learning (ML)	Prognosis & Prediction
682	10.1177/03635465231205556	2024	Sports Medicine; Trauma; Pediatric Orthopaedics	Natural Language Processing (NLP); Machine Learning (ML)	Prognosis & Prediction
683	10.1177/23259671251329355	2025	Sports Medicine; Trauma; Pediatric Orthopaedics	Machine Learning (ML)	Prognosis & Prediction
684	10.1186/s43019-025-00261-w	2025	Arthroplasty	Unclassified	Unclassified
685	10.1097/CORR.0000000000003046	2024	Arthroplasty	Machine Learning (ML)	Prognosis & Prediction
686	10.3340/jkns.2021.0213	2023	Spine Surgery	Machine Learning (ML); Robotics	Prognosis & Prediction
687	10.1007/s43465-023-00889-4	2023	Arthroplasty	Deep Learning (DL)	Unclassified
688	N/A	2024	Unclassified	Deep Learning (DL)	Prognosis & Prediction
689	N/A	2020	Unclassified	Machine Learning (ML)	Prognosis & Prediction

690	10.1002/jeo2.70581	2025	Unclassified	Machine Learning (ML)	Surgical Planning
691	10.1177/03635465221112095	2022	Sports Medicine; Trauma	Machine Learning (ML)	Prognosis & Prediction
692	10.1016/j.artd.2024.101491	2024	Arthroplasty	Machine Learning (ML)	Prognosis & Prediction
693	10.1016/j.joca.2019.02.397	2019	Unclassified	Deep Learning (DL)	Unclassified
694	N/A	2024	Unclassified	Machine Learning (ML)	Prognosis & Prediction
695	N/A	2021	Unclassified	Machine Learning (ML)	Unclassified
696	10.1016/j.joca.2018.02.558	2018	Unclassified	Machine Learning (ML)	Prognosis & Prediction
697	10.2106/JBJS.OA.24.00099	2024	Trauma	Natural Language Processing (NLP)	Unclassified
698	10.1093/neuros/nyab085	2021	Spine Surgery	Machine Learning (ML)	Prognosis & Prediction
699	10.1007/s00256-025-04917-2	2025	Spine Surgery	Deep Learning (DL)	Prognosis & Prediction
700	10.1016/j.arth.2020.05.077	2020	Sports Medicine; Spine Surgery; Arthroplasty	Machine Learning (ML)	Prognosis & Prediction
701	N/A	2025	Unclassified	Machine Learning (ML)	Prognosis & Prediction
702	10.1016/j.jot.2023.11.002	2024	Arthroplasty	Natural Language Processing (NLP)	Diagnosis & Detection
703	10.1007/s00586-025-09053-y	2025	Spine Surgery	Machine Learning (ML); Deep Learning (DL)	Prognosis & Prediction
704	10.1097/CORR.0000000000000601	2019	Trauma; Arthroplasty	Machine Learning (ML)	Prognosis & Prediction
705	10.24310/riccafd.13.3.2024.20429	2024	Unclassified	Natural Language Processing (NLP)	Prognosis & Prediction
706	10.1016/j.jor.2019.12.013	2020	Arthroplasty	Machine Learning (ML); Robotics	Prognosis & Prediction
707	10.1007/s00423-025-03844-z	2025	Trauma; Arthroplasty	Natural Language Processing (NLP)	Unclassified
708	N/A	2022	Unclassified	Deep Learning (DL)	Prognosis & Prediction
709	10.1007/s00586-022-07354-0	2022	Spine Surgery	Unclassified	Prognosis & Prediction
710	N/A	2021	Trauma	Machine Learning (ML)	Prognosis & Prediction
711	N/A	2023	Sports Medicine; Trauma	Machine Learning (ML)	Prognosis & Prediction
712	10.1227/neu.0000000000003610	2025	Arthroplasty	Machine Learning (ML)	Prognosis & Prediction
713	10.1186/s13018-025-05518-4	2025	Arthroplasty	Machine Learning (ML); Robotics	Prognosis & Prediction
714	10.1016/j.artd.2025.101795	2025	Arthroplasty	Natural Language Processing (NLP)	Unclassified

715	10.1097/JS9.0000000000000027 06	2025	Unclassified	Natural Language Processing (NLP)	Unclassified
716	N/A	2025	Trauma	Machine Learning (ML)	Diagnosis & Detection
717	10.1097/CORR.00000000000 01402	2020	Arthroplasty	Unclassified	Prognosis & Prediction
718	N/A	2023	Trauma	Machine Learning (ML)	Prognosis & Prediction
719	10.1007/s00256-025-04991-6	2026	Arthroplasty	Deep Learning (DL)	Prognosis & Prediction
720	10.1055/s-0040-1710573	2022	Arthroplasty	Machine Learning (ML)	Prognosis & Prediction
721	10.1080/14763141.2021.1959 947	2021	Sports Medicine; Trauma; Arthroplasty	Unclassified	Prognosis & Prediction
722	N/A	2023	Unclassified	Machine Learning (ML)	Diagnosis & Detection
723	10.3171/2024.4.SPINE231148	2024	Spine Surgery	Natural Language Processing (NLP)	Diagnosis & Detection
724	10.1016/j.jot.2025.07.010	2025	Unclassified	Unclassified	Prognosis & Prediction
725	N/A	2022	Arthroplasty	Machine Learning (ML)	Prognosis & Prediction
726	10.1111/os.14076	2024	Sports Medicine; Arthroplasty	Machine Learning (ML)	Prognosis & Prediction
727	10.14245/ns.2142816.408	2021	Trauma	Unclassified	Prognosis & Prediction
728	10.1007/s00586-022-07311-x	2022	Spine Surgery	Machine Learning (ML); Deep Learning (DL)	Diagnosis & Detection
729	10.1016/j.clinbiomech.2025.1 06625	2025	Trauma; Spine Surgery	Unclassified	Prognosis & Prediction
730	10.1016/j.jhsa.2023.08.003	2023	Trauma	Natural Language Processing (NLP)	Unclassified
731	10.1177/23259671241291920	2024	Sports Medicine	Natural Language Processing (NLP); Machine Learning (ML)	Prognosis & Prediction
732	N/A	2021	Unclassified	Machine Learning (ML)	Unclassified
733	10.1590/1413-785220233102e263885	2023	Trauma; Arthroplasty	Unclassified	Unclassified
734	N/A	2024	Unclassified	Machine Learning (ML)	Unclassified
735	10.1016/j.wneu.2024.03.001	2024	Spine Surgery	Natural Language Processing (NLP)	Unclassified
736	10.1016/j.clinbiomech.2021.1 05447	2021	Arthroplasty	Machine Learning (ML)	Prognosis & Prediction
737	10.5435/JAAOS-D-21-00241	2022	Spine Surgery	Machine Learning (ML)	Prognosis & Prediction
738	10.1016/j.arth.2020.07.035	2021	Arthroplasty	Machine Learning (ML)	Prognosis & Prediction
739	10.1055/a-1534-3346	2021	Unclassified	Unclassified	Unclassified

740	10.1016/j.wneu.2024.05.073	2024	Spine Surgery	Natural Language Processing (NLP)	Unclassified
741	N/A	2022	Unclassified	Deep Learning (DL)	Prognosis & Prediction
742	N/A	2024	Arthroplasty	Machine Learning (ML)	Prognosis & Prediction
743	10.1016/j.bjoms.2025.01.013	2025	Arthroplasty	Unclassified	Diagnosis & Detection
744	N/A	2020	Unclassified	Deep Learning (DL)	Prognosis & Prediction
745	N/A	2023	Arthroplasty	Machine Learning (ML)	Prognosis & Prediction
746	10.1016/j.clinbiomech.2024.106285	2024	Unclassified	Machine Learning (ML)	Diagnosis & Detection
747	N/A	2022	Unclassified	Machine Learning (ML)	Unclassified
748	10.1016/j.wneu.2024.03.057	2024	Spine Surgery	Natural Language Processing (NLP)	Unclassified
749	N/A	2019	Sports Medicine	Deep Learning (DL)	Unclassified
750	10.1007/s00256-025-05097-9	2025	Unclassified	Unclassified	Unclassified
751	N/A	2024	Unclassified	Unclassified	Unclassified
752	N/A	2025	Unclassified	Machine Learning (ML)	Unclassified
753	10.1302/0301-620X.106B11.BJJ-2024-0373	2024	Arthroplasty	Unclassified	Prognosis & Prediction
754	10.1007/s43465-025-01423-4	2025	Trauma; Arthroplasty	Unclassified	Prognosis & Prediction
755	10.1007/s43390-024-00954-4	2025	Spine Surgery	Deep Learning (DL)	Prognosis & Prediction
756	10.1177/10225536251368567	2025	Arthroplasty	Natural Language Processing (NLP)	Unclassified
757	10.1002/jso.28052	2025	Unclassified	Unclassified	Unclassified
758	10.1097/CORR.0000000000003293	2024	Arthroplasty	Natural Language Processing (NLP)	Unclassified
759	10.20517/ais.2024.60	2025	Spine Surgery	Unclassified	Prognosis & Prediction
760	10.1016/j.otsr.2024.103985	2025	Arthroplasty	Machine Learning (ML)	Prognosis & Prediction
761	10.1002/ksa.70073	2025	Arthroplasty	Robotics	Prognosis & Prediction
762	10.1097/CORR.0000000000001344	2020	Arthroplasty	Machine Learning (ML)	Prognosis & Prediction
763	10.1186/s12891-023-06309-w	2023	Unclassified	Unclassified	Prognosis & Prediction
764	10.1016/j.arth.2019.01.036	2019	Arthroplasty	Machine Learning (ML)	Unclassified

765	N/A	2023	Unclassified	Unclassified	Diagnosis & Detection
766	10.5435/JAAOS-D-25-00260	2025	Unclassified	Natural Language Processing (NLP)	Unclassified
767	10.1097/CORR.0000000000001995	2022	Unclassified	Unclassified	Unclassified
768	N/A	2024	Arthroplasty	Deep Learning (DL)	Prognosis & Prediction
769	10.1002/jso.27514	2024	Unclassified	Machine Learning (ML)	Prognosis & Prediction
770	10.1016/j.arth.2022.02.104	2022	Arthroplasty	Machine Learning (ML)	Unclassified
771	10.1016/j.wnsx.2025.100437	2025	Spine Surgery	Natural Language Processing (NLP)	Prognosis & Prediction
772	10.1016/j.wneu.2025.124236	2025	Sports Medicine; Spine Surgery	Deep Learning (DL)	Diagnosis & Detection
773	10.1016/j.hansur.2024.101723	2024	Unclassified	Natural Language Processing (NLP)	Unclassified
774	10.4055/cios24148	2025	Trauma; Arthroplasty	Unclassified	Prognosis & Prediction
775	10.1016/j.spinee.2025.03.021	2025	Spine Surgery	Natural Language Processing (NLP); Machine Learning (ML)	Prognosis & Prediction
776	10.1016/j.joca.2019.02.393	2019	Unclassified	Machine Learning (ML)	Unclassified
777	N/A	2024	Unclassified	Unclassified	Prognosis & Prediction
778	10.14245/ns.1938434.217	2019	Spine Surgery	Machine Learning (ML)	Unclassified
779	10.14245/ns.1938390.195	2019	Trauma; Spine Surgery	Machine Learning (ML)	Prognosis & Prediction
780	10.17816/2311-2905-17754	2025	Arthroplasty	Machine Learning (ML)	Prognosis & Prediction
781	10.1227/neu.0000000000003360_1216	2025	Spine Surgery	Machine Learning (ML)	Segmentation & Measurement
782	10.1007/s00586-022-07357-x	2022	Spine Surgery	Unclassified	Prognosis & Prediction
783	10.1177/17585732251392203	2025	Unclassified	Deep Learning (DL)	Segmentation & Measurement
784	10.1016/j.arth.2019.01.034	2019	Arthroplasty	Machine Learning (ML)	Unclassified
785	10.1097/BRS.0000000000004125	2021	Sports Medicine; Spine Surgery	Machine Learning (ML)	Prognosis & Prediction
786	10.1177/03635465231177905	2023	Sports Medicine	Machine Learning (ML)	Prognosis & Prediction
787	10.1016/j.arth.2024.06.059	2025	Arthroplasty	Natural Language Processing (NLP); Deep Learning (DL)	Segmentation & Measurement

788	10.1007/s00586-025-08835-8	2025	Spine Surgery; Trauma; Arthroplasty	Unclassified	Prognosis & Prediction
789	10.1016/j.joca.2018.02.110	2018	Sports Medicine	Deep Learning (DL)	Segmentation & Measurement
790	10.14444/8502	2023	Unclassified	Machine Learning (ML)	Prognosis & Prediction
791	N/A	2023	Unclassified	Unclassified	Unclassified
792	N/A	2025	Arthroplasty	Machine Learning (ML)	Prognosis & Prediction
793	10.1016/j.wneu.2024.05.088	2024	Spine Surgery	Natural Language Processing (NLP)	Unclassified
794	10.1016/j.wneu.2024.03.010	2024	Spine Surgery	Natural Language Processing (NLP)	Unclassified
795	10.1186/s13018-025-05793-1	2025	Arthroplasty	Machine Learning (ML)	Surgical Planning
796	10.1097/CORR.0000000000002139	2022	Unclassified	Machine Learning (ML)	Prognosis & Prediction
797	N/A	2024	Pediatric Orthopaedics; Arthroplasty	Unclassified	Unclassified
798	10.1016/j.arth.2024.02.006	2024	Sports Medicine; Trauma; Arthroplasty	Machine Learning (ML)	Prognosis & Prediction
799	10.1016/j.wneu.2024.03.113	2024	Spine Surgery	Natural Language Processing (NLP)	Unclassified
800	N/A	2020	Spine Surgery	Unclassified	Prognosis & Prediction
801	10.1016/j.gaitpost.2018.08.026	2018	Arthroplasty	Unclassified	Prognosis & Prediction
802	10.1002/jeo2.70521	2025	Sports Medicine	Natural Language Processing (NLP)	Unclassified
803	10.1097/BRS.0000000000004436	2022	Spine Surgery; Arthroplasty	Machine Learning (ML)	Prognosis & Prediction
804	10.1016/j.arth.2025.01.003	2025	Trauma; Arthroplasty	Natural Language Processing (NLP); Machine Learning (ML)	Prognosis & Prediction
805	N/A	2021	Unclassified	Machine Learning (ML)	Unclassified
806	10.1016/j.joca.2024.03.009	2024	Unclassified	Deep Learning (DL)	Unclassified
807	N/A	2022	Unclassified	Machine Learning (ML)	Unclassified
808	10.1097/BRS.0000000000005088	2024	Sports Medicine; Spine Surgery	Machine Learning (ML); Deep Learning (DL)	Prognosis & Prediction
809	10.1016/j.asjsur.2024.09.128	2025	Spine Surgery	Deep Learning (DL)	Segmentation & Measurement
810	10.1097/BPO.0000000000002824	2025	Pediatric Orthopaedics	Natural Language Processing (NLP)	Unclassified
811	10.1093/neuros/nyz001.2	2019	Spine Surgery	Machine Learning (ML)	Prognosis & Prediction
812	10.1007/s00132-025-04708-6	2025	Unclassified	Unclassified	Prognosis & Prediction

813	N/A	2020	Sports Medicine	Machine Learning (ML)	Unclassified
814	10.14444/8611	2024	Spine Surgery; Arthroplasty	Natural Language Processing (NLP); Machine Learning (ML); Robotics	Unclassified
815	N/A	2023	Unclassified	Deep Learning (DL)	Unclassified
816	N/A	2024	Unclassified	Deep Learning (DL)	Diagnosis & Detection
817	10.1186/s12891-023-06330-z	2023	Sports Medicine; Spine Surgery; Arthroplasty	Machine Learning (ML)	Prognosis & Prediction
818	10.1016/j.wneu.2023.10.073	2024	Trauma; Spine Surgery	Machine Learning (ML)	Prognosis & Prediction
819	10.1136/tsaco-2023-001300	2024	Trauma	Deep Learning (DL)	Prognosis & Prediction
820	10.14444/8500	2023	Unclassified	Machine Learning (ML)	Prognosis & Prediction
821	10.31616/asj.2024.0337	2024	Trauma; Spine Surgery	Machine Learning (ML); Robotics	Prognosis & Prediction
822	10.1007/s00142-025-00776-0	2025	Sports Medicine	Natural Language Processing (NLP)	Unclassified
823	10.1007/s11548-022-02740-x	2022	Pediatric Orthopaedics; Spine Surgery	Deep Learning (DL)	Surgical Planning
824	10.1016/j.joca.2020.05.002	2020	Sports Medicine; Arthroplasty	Natural Language Processing (NLP); Machine Learning (ML)	Diagnosis & Detection
825	10.1186/s13018-025-06206-z	2025	Arthroplasty	Machine Learning (ML)	Prognosis & Prediction
826	10.1053/j.jfas.2025.05.010	2025	Arthroplasty	Natural Language Processing (NLP); Machine Learning (ML)	Unclassified
827	10.1016/j.arth.2023.10.035	2024	Pediatric Orthopaedics; Spine Surgery; Arthroplasty	Unclassified	Prognosis & Prediction
828	N/A	2020	Spine Surgery	Machine Learning (ML)	Prognosis & Prediction
829	10.3928/01477447-20250123-03	2025	Trauma; Arthroplasty	Natural Language Processing (NLP)	Unclassified
830	N/A	2024	Spine Surgery	Natural Language Processing (NLP)	Unclassified
831	10.1186/s12891-025-09234-2	2025	Sports Medicine	Machine Learning (ML)	Prognosis & Prediction
832	N/A	2020	Spine Surgery	Machine Learning (ML)	Prognosis & Prediction
833	10.3171/2020.10.FOCUS20792	2021	Spine Surgery	Robotics	Unclassified
834	10.1007/s00256-024-04624-4	2024	Arthroplasty	Deep Learning (DL)	Unclassified
835	N/A	2021	Unclassified	Machine Learning (ML)	Unclassified
836	10.1177/1947603519888793	2021	Sports Medicine; Arthroplasty	Unclassified	Diagnosis & Detection

837	10.5435/JAAOS-D-20-00288	2021	Unclassified	Natural Language Processing (NLP); Machine Learning (ML)	Unclassified
838	10.1186/s13018-021-02437-y	2021	Sports Medicine; Arthroplasty	Natural Language Processing (NLP); Machine Learning (ML)	Unclassified
839	10.3233/BMR-240059	2024	Unclassified	Machine Learning (ML)	Prognosis & Prediction
840	10.2106/JBJS.19.01122	2019	Unclassified	Natural Language Processing (NLP)	Unclassified
841	10.1016/j.clinbiomech.2025.106663	2025	Sports Medicine; Trauma; Arthroplasty	Deep Learning (DL)	Prognosis & Prediction
842	10.1186/s12893-023-01959-y	2023	Trauma; Spine Surgery	Machine Learning (ML)	Prognosis & Prediction
843	10.1186/s12891-022-05376-9	2022	Unclassified	Machine Learning (ML)	Prognosis & Prediction
844	10.1016/j.jseint.2025.06.014	2025	Arthroplasty	Machine Learning (ML)	Prognosis & Prediction
845	10.1186/s12891-024-07355-8	2024	Trauma	Machine Learning (ML); Deep Learning (DL)	Diagnosis & Detection
846	N/A	2025	Unclassified	Machine Learning (ML)	Unclassified
847	10.1227/neuprac.0000000000000099	2024	Spine Surgery	Machine Learning (ML)	Prognosis & Prediction
848	10.1007/s43465-024-01281-6	2024	Sports Medicine; Trauma; Arthroplasty	Robotics	Diagnosis & Detection
849	10.1007/s00586-025-09460-1	2025	Trauma; Spine Surgery	Machine Learning (ML)	Prognosis & Prediction
850	10.1016/j.hcl.2025.08.003	2026	Unclassified	Machine Learning (ML)	Prognosis & Prediction
851	N/A	2021	Sports Medicine	Deep Learning (DL)	Segmentation & Measurement
852	N/A	2020	Unclassified	Deep Learning (DL)	Unclassified
853	10.1097/CORR.0000000000002397	2022	Unclassified	Machine Learning (ML)	Prognosis & Prediction
854	10.1016/j.spinee.2023.09.019	2024	Trauma; Spine Surgery	Natural Language Processing (NLP); Machine Learning (ML); Deep Learning (DL)	Prognosis & Prediction
855	10.1177/17531934241258868	2024	Sports Medicine; Arthroplasty	Unclassified	Segmentation & Measurement
856	10.1016/j.wneu.2025.124514	2025	Spine Surgery	Robotics	Prognosis & Prediction
857	10.1186/s43019-025-00268-3	2025	Unclassified	Deep Learning (DL)	Unclassified

858	N/A	2020	Sports Medicine; Trauma	Deep Learning (DL)	Segmentation & Measurement
859	10.1111/os.13952	2024	Arthroplasty	Unclassified	Prognosis & Prediction
860	10.1302/2046-3758.99.BJR-2019-0192.R1	2020	Arthroplasty	Machine Learning (ML)	Prognosis & Prediction
861	10.1016/j.bjoms.2025.03.008	2025	Arthroplasty	Unclassified	Diagnosis & Detection
862	10.1016/j.wneu.2022.08.149	2022	Spine Surgery	Machine Learning (ML)	Prognosis & Prediction
863	10.1177/00031348251358446	2026	Trauma	Machine Learning (ML)	Prognosis & Prediction
864	10.1177/21925682241290226	2025	Spine Surgery	Unclassified	Unclassified
865	10.1007/s00256-025-04927-0	2025	Trauma; Pediatric Orthopaedics	Deep Learning (DL)	Diagnosis & Detection
866	10.1016/j.arthro.2022.07.012	2023	Sports Medicine	Machine Learning (ML)	Prognosis & Prediction
867	N/A	2024	Unclassified	Deep Learning (DL)	Diagnosis & Detection
868	10.1016/j.joca.2019.07.014	2019	Unclassified	Machine Learning (ML)	Unclassified
869	10.1007/s00167-020-06321-w	2021	Sports Medicine	Machine Learning (ML)	Prognosis & Prediction
870	10.1007/s00586-018-5497-0	2018	Spine Surgery	Machine Learning (ML)	Prognosis & Prediction
871	10.2106/JBJS.ER.20.00875	2023	Spine Surgery	Machine Learning (ML)	Unclassified
872	10.1002/jeo2.70457	2025	Unclassified	Natural Language Processing (NLP)	Prognosis & Prediction
873	10.1007/s00586-024-08376-6	2025	Sports Medicine; Pediatric Orthopaedics	Machine Learning (ML)	Prognosis & Prediction
874	10.3340/jkns.2020.0258	2021	Pediatric Orthopaedics; Trauma; Spine Surgery	Unclassified	Prognosis & Prediction
875	10.5435/JAAOS-D-19-00395	2020	Arthroplasty	Machine Learning (ML)	Prognosis & Prediction
876	10.14444/8778	2025	Spine Surgery	Natural Language Processing (NLP)	Unclassified
877	10.1016/j.joca.2019.02.385	2019	Unclassified	Deep Learning (DL)	Unclassified
878	10.1186/s13018-023-03863-w	2023	Unclassified	Machine Learning (ML)	Prognosis & Prediction
879	10.5435/JAAOS-D-24-01234	2025	Sports Medicine; Trauma; Spine Surgery	Unclassified	Prognosis & Prediction
880	10.1007/s00402-024-05554-5	2024	Trauma	Natural Language Processing (NLP)	Diagnosis & Detection

881	10.1016/j.arthro.2021.02.032	2021	Unclassified	Natural Language Processing (NLP); Machine Learning (ML)	Prognosis & Prediction
882	10.1097/CORR.0000000000002398	2022	Unclassified	Machine Learning (ML)	Prognosis & Prediction
883	10.1007/s00256-022-04070-0	2022	Trauma; Pediatric Orthopaedics	Deep Learning (DL)	Diagnosis & Detection
884	10.1007/s00586-019-06115-w	2019	Sports Medicine; Spine Surgery	Deep Learning (DL)	Diagnosis & Detection
885	N/A	2020	Trauma	Machine Learning (ML)	Prognosis & Prediction
886	10.1111/os.14362	2025	Pediatric Orthopaedics	Unclassified	Surgical Planning
887	10.1016/j.jse.2020.07.042	2021	Trauma; Arthroplasty	Machine Learning (ML)	Prognosis & Prediction
888	10.1016/j.jse.2019.10.031	2020	Arthroplasty	Machine Learning (ML)	Prognosis & Prediction
889	10.1016/j.arth.2020.06.063	2020	Trauma; Arthroplasty	Machine Learning (ML)	Prognosis & Prediction
890	10.2106/JBJS.24.01601	2025	Trauma; Arthroplasty	Deep Learning (DL)	Prognosis & Prediction
891	10.1097/BRS.0000000000005435	2025	Sports Medicine; Spine Surgery	Natural Language Processing (NLP); Deep Learning (DL)	Segmentation & Measurement
892	10.31616/asj.2024.0314	2025	Trauma; Spine Surgery	Unclassified	Prognosis & Prediction
893	10.3389/fsurg.2020.575318	2020	Spine Surgery	Robotics	Prognosis & Prediction
894	10.1097/SCS.0000000000007863	2022	Unclassified	Machine Learning (ML)	Diagnosis & Detection
895	10.1016/j.arth.2025.05.048	2025	Arthroplasty	Natural Language Processing (NLP); Machine Learning (ML)	Prognosis & Prediction
896	10.1016/j.ijsu.2022.106851	2022	Unclassified	Deep Learning (DL)	Prognosis & Prediction
897	10.1186/s13018-023-04128-2	2023	Arthroplasty	Machine Learning (ML)	Diagnosis & Detection
898	10.1186/s13018-023-03837-y	2023	Sports Medicine; Arthroplasty	Machine Learning (ML)	Surgical Planning
899	10.1177/17585732231187124	2024	Arthroplasty	Machine Learning (ML)	Prognosis & Prediction
900	10.1002/jsp2.70013	2024	Spine Surgery	Deep Learning (DL)	Unclassified
901	10.1302/0301-620X.102B7.BJJ-2019-1574.R1	2020	Arthroplasty	Natural Language Processing (NLP)	Prognosis & Prediction
902	10.1227/neu.0000000000003074	2025	Spine Surgery	Machine Learning (ML)	Prognosis & Prediction

903	10.1136/bjsports-2022-106142	2023	Sports Medicine	Machine Learning (ML)	Prognosis & Prediction
904	10.1111/den.14187	2022	Arthroplasty	Deep Learning (DL)	Prognosis & Prediction
905	10.1002/ksa.12362	2025	Arthroplasty	Deep Learning (DL)	Segmentation & Measurement
906	10.1016/j.artd.2024.101484	2024	Arthroplasty	Natural Language Processing (NLP)	Prognosis & Prediction
907	10.1016/j.arth.2020.12.019	2021	Arthroplasty	Machine Learning (ML)	Prognosis & Prediction
908	N/A	2021	Spine Surgery	Machine Learning (ML)	Prognosis & Prediction
909	10.1007/s00256-018-3118-y	2019	Trauma; Spine Surgery	Natural Language Processing (NLP)	Diagnosis & Detection
910	10.1177/23259671241312681	2025	Sports Medicine; Trauma; Arthroplasty	Machine Learning (ML)	Prognosis & Prediction
911	10.1002/ksa.12462	2025	Sports Medicine; Trauma; Pediatric Orthopaedics	Machine Learning (ML)	Prognosis & Prediction
912	10.2106/JBJS.18.00695	2019	Arthroplasty	Machine Learning (ML)	Prognosis & Prediction
913	10.2106/JBJS.24.00427	2025	Sports Medicine; Trauma	Unclassified	Prognosis & Prediction
914	10.1016/j.arth.2025.05.052	2025	Sports Medicine; Trauma; Arthroplasty	Natural Language Processing (NLP); Robotics; Deep Learning (DL)	Surgical Planning
915	10.1007/s00586-025-08931-9	2025	Spine Surgery	Natural Language Processing (NLP)	Unclassified
916	10.1007/s11657-025-01613-5	2025	Trauma	Natural Language Processing (NLP); Machine Learning (ML)	Prognosis & Prediction
917	10.1016/j.otsr.2024.103842	2024	Arthroplasty	Machine Learning (ML)	Prognosis & Prediction
918	10.1007/s00586-024-08253-2	2024	Spine Surgery	Natural Language Processing (NLP)	Unclassified
919	10.1097/SHK.0000000000002549	2025	Trauma	Machine Learning (ML)	Prognosis & Prediction
920	10.1002/jor.25800	2024	Sports Medicine	Deep Learning (DL)	Diagnosis & Detection
921	10.1007/s00586-024-08272-z	2024	Spine Surgery	Natural Language Processing (NLP)	Unclassified
922	10.1016/j.artd.2023.101181	2023	Arthroplasty	Machine Learning (ML)	Prognosis & Prediction
923	10.23736/S0022-4707.25.17137-5	2025	Sports Medicine; Trauma	Natural Language Processing (NLP)	Prognosis & Prediction
924	10.1007/s00256-025-04936-z	2025	Sports Medicine; Arthroplasty	Unclassified	Prognosis & Prediction
925	10.1016/j.clinbiomech.2024.106192	2024	Trauma	Robotics; Deep Learning (DL)	Prognosis & Prediction

926	10.1177/21514593231179316	2023	Arthroplasty	Machine Learning (ML)	Prognosis & Prediction
927	10.1186/s13018-025-06139-7	2025	Arthroplasty	Machine Learning (ML)	Prognosis & Prediction
928	10.1007/s40279-025-02196-4	2025	Trauma; Pediatric Orthopaedics	Machine Learning (ML)	Prognosis & Prediction
929	10.1007/s00402-022-04452-y	2023	Sports Medicine; Trauma; Arthroplasty	Machine Learning (ML)	Prognosis & Prediction
930	10.1093/neuros/nyaa209	2020	Spine Surgery	Machine Learning (ML)	Unclassified
931	10.5435/JAAOS-D-23-01144	2025	Trauma	Machine Learning (ML)	Prognosis & Prediction
932	10.1016/j.spinee.2019.01.009	2019	Spine Surgery	Natural Language Processing (NLP); Machine Learning (ML)	Prognosis & Prediction
933	10.1007/s11548-024-03290-0	2025	Arthroplasty	Machine Learning (ML); Deep Learning (DL)	Prognosis & Prediction
934	10.1186/s13018-024-04588-0	2024	Arthroplasty	Unclassified	Prognosis & Prediction
935	10.1016/j.arth.2024.03.026	2024	Sports Medicine; Arthroplasty	Natural Language Processing (NLP)	Prognosis & Prediction
936	10.1007/s00402-023-05069-5	2024	Arthroplasty	Machine Learning (ML)	Prognosis & Prediction
937	10.1186/s13018-024-05002-5	2024	Spine Surgery	Deep Learning (DL)	Diagnosis & Detection
938	10.1007/s00256-025-05085-z	2025	Sports Medicine; Trauma	Machine Learning (ML)	Prognosis & Prediction
939	10.1016/j.arthro.2022.01.026	2022	Unclassified	Natural Language Processing (NLP); Machine Learning (ML)	Prognosis & Prediction
940	10.1016/j.joca.2017.09.001	2017	Unclassified	Machine Learning (ML)	Prognosis & Prediction
941	10.1177/21925682241274371	2025	Spine Surgery; Trauma; Arthroplasty	Machine Learning (ML)	Prognosis & Prediction
942	10.1186/s13018-022-03314-y	2022	Sports Medicine	Natural Language Processing (NLP); Machine Learning (ML)	Prognosis & Prediction
943	10.1016/j.humov.2025.103366	2025	Arthroplasty	Unclassified	Prognosis & Prediction
944	10.1016/j.joca.2023.03.008	2023	Trauma; Arthroplasty	Machine Learning (ML)	Prognosis & Prediction
945	10.1007/s00586-025-08653-y	2025	Spine Surgery	Machine Learning (ML)	Prognosis & Prediction
946	10.1007/s11548-025-03485-z	2025	Trauma; Pediatric Orthopaedics	Machine Learning (ML)	Prognosis & Prediction

947	10.1007/s00590-022-03446-1	2023	Arthroplasty	Machine Learning (ML)	Prognosis & Prediction
948	10.1007/s11701-024-02001-w	2024	Spine Surgery	Robotics	Prognosis & Prediction
949	10.1007/s00256-023-04502-5	2024	Sports Medicine; Arthroplasty	Deep Learning (DL)	Segmentation & Measurement
950	10.1016/j.arth.2020.05.061	2020	Arthroplasty	Machine Learning (ML)	Prognosis & Prediction
951	10.1016/j.jisako.2024.100306	2024	Sports Medicine; Trauma	Natural Language Processing (NLP)	Prognosis & Prediction
952	10.1016/j.arth.2021.02.063	2021	Sports Medicine; Arthroplasty	Machine Learning (ML); Robotics	Prognosis & Prediction
953	10.1007/s00590-023-03805-6	2024	Trauma; Arthroplasty	Machine Learning (ML)	Prognosis & Prediction
954	10.1016/j.xnsj.2024.100562	2024	Spine Surgery	Natural Language Processing (NLP)	Prognosis & Prediction
955	10.1007/s00586-025-08939-1	2025	Spine Surgery	Natural Language Processing (NLP)	Unclassified
956	10.1007/s00264-021-05176-1	2021	Arthroplasty	Machine Learning (ML)	Prognosis & Prediction
957	10.1002/jeo2.70084	2024	Unclassified	Deep Learning (DL)	Prognosis & Prediction
958	10.1016/j.xnsj.2024.100564	2024	Spine Surgery	Deep Learning (DL)	Prognosis & Prediction
959	N/A	2022	Arthroplasty	Machine Learning (ML)	Segmentation & Measurement
960	10.1302/2046-3758.129.BJR-2023-0115.R1	2023	Unclassified	Unclassified	Diagnosis & Detection
961	10.2106/JBJS.RVW.22.00086	2022	Spine Surgery	Robotics	Unclassified
962	10.1249/JES.0000000000000121	2017	Spine Surgery	Unclassified	Prognosis & Prediction
963	10.1016/j.spinee.2024.10.010	2025	Spine Surgery	Machine Learning (ML)	Prognosis & Prediction
964	10.1007/s00256-025-04995-2	2026	Trauma; Spine Surgery	Deep Learning (DL)	Prognosis & Prediction
965	10.1007/s00402-021-04149-8	2023	Sports Medicine; Arthroplasty	Unclassified	Prognosis & Prediction
966	10.1186/s10195-025-00841-2	2025	Arthroplasty	Unclassified	Prognosis & Prediction
967	10.1080/17453674.2020.1723292	2020	Trauma	Deep Learning (DL)	Diagnosis & Detection

968	10.1007/s00586-024-08257-y	2024	Spine Surgery	Natural Language Processing (NLP)	Unclassified
969	10.1007/s00586-021-06916-y	2021	Spine Surgery	Unclassified	Unclassified
970	10.1007/s00586-020-06447-y	2020	Spine Surgery	Unclassified	Prognosis & Prediction
971	10.1007/s00586-025-09369-9	2025	Trauma; Spine Surgery	Machine Learning (ML)	Prognosis & Prediction
972	10.1007/s12178-025-09986-3	2025	Pediatric Orthopaedics	Unclassified	Prognosis & Prediction
973	10.1007/s00167-023-07497-7	2023	Sports Medicine; Trauma	Machine Learning (ML)	Prognosis & Prediction
974	10.1097/BRS.0000000000004510	2023	Trauma; Spine Surgery	Machine Learning (ML)	Prognosis & Prediction
975	10.1055/s-0042-1756503	2023	Arthroplasty	Robotics	Prognosis & Prediction
976	10.5435/JAAOS-D-23-00087	2023	Sports Medicine; Pediatric Orthopaedics	Machine Learning (ML)	Prognosis & Prediction
977	10.1177/03635465251359618	2025	Unclassified	Deep Learning (DL)	Prognosis & Prediction
978	10.14245/ns.2448202.101	2024	Trauma; Spine Surgery	Deep Learning (DL)	Segmentation & Measurement
979	10.1007/s11657-020-00827-z	2020	Trauma	Unclassified	Prognosis & Prediction
980	10.1016/j.clinbiomech.2025.106648	2025	Arthroplasty	Unclassified	Prognosis & Prediction
981	10.1002/jor.25693	2024	Unclassified	Machine Learning (ML)	Prognosis & Prediction
982	10.3928/01477447-20220225-02	2022	Arthroplasty	Machine Learning (ML)	Prognosis & Prediction
983	10.1007/s00586-025-08781-5	2025	Spine Surgery	Machine Learning (ML)	Prognosis & Prediction
984	10.1186/s12891-025-08733-6	2025	Sports Medicine; Trauma	Machine Learning (ML); Deep Learning (DL)	Diagnosis & Detection
985	10.1016/j.arth.2023.09.027	2024	Arthroplasty	Machine Learning (ML)	Prognosis & Prediction
986	10.1007/s00586-024-08623-w	2025	Trauma; Spine Surgery	Deep Learning (DL)	Diagnosis & Detection
987	10.1007/s00590-023-03766-w	2024	Trauma	Natural Language Processing (NLP)	Unclassified
988	10.1016/j.jseint.2023.04.003	2024	Trauma; Arthroplasty	Machine Learning (ML)	Prognosis & Prediction

989	10.1227/neu.0000000000003335	2025	Trauma; Spine Surgery	Robotics	Prognosis & Prediction
990	10.1016/j.otsm.2025.151189	2025	Sports Medicine; Arthroplasty	Natural Language Processing (NLP); Machine Learning (ML); Robotics	Prognosis & Prediction
991	10.1097/JS9.0000000000003030	2025	Unclassified	Unclassified	Prognosis & Prediction
992	10.1016/j.knee.2024.02.006	2024	Trauma; Arthroplasty	Machine Learning (ML)	Prognosis & Prediction
993	10.1002/jsp2.1326	2024	Spine Surgery	Machine Learning (ML); Deep Learning (DL)	Unclassified
994	10.5435/JAAOS-D-24-01274	2025	Pediatric Orthopaedics	Natural Language Processing (NLP)	Unclassified
995	10.1007/s00132-024-04567-7	2024	Arthroplasty	Unclassified	Prognosis & Prediction
996	10.1016/j.ocarto.2023.100334	2023	Sports Medicine; Arthroplasty	Unclassified	Prognosis & Prediction
997	10.1186/s13102-021-00243-x	2021	Sports Medicine	Unclassified	Diagnosis & Detection
998	10.1186/s42836-023-00219-x	2023	Arthroplasty	Machine Learning (ML)	Prognosis & Prediction
999	10.1007/s00132-023-04456-5	2023	Unclassified	Machine Learning (ML)	Unclassified
1000	10.1016/j.arthro.2021.07.001	2021	Trauma; Arthroplasty	Unclassified	Unclassified
1001	10.3171/2020.2.SPINE20127	2020	Spine Surgery	Natural Language Processing (NLP)	Prognosis & Prediction
1002	10.23736/S0022-4707.23.15417-X	2024	Trauma	Machine Learning (ML)	Prognosis & Prediction
1003	10.1080/02640414.2025.2524283	2025	Arthroplasty	Deep Learning (DL)	Unclassified
1004	10.1007/s00586-025-09446-z	2025	Spine Surgery	Machine Learning (ML)	Prognosis & Prediction
1005	10.1016/j.artd.2024.101553	2024	Arthroplasty	Unclassified	Diagnosis & Detection
1006	10.1097/CORR.0000000000002668	2023	Spine Surgery; Arthroplasty	Natural Language Processing (NLP)	Unclassified
1007	10.3171/2020.3.SPINE191541	2020	Pediatric Orthopaedics; Spine Surgery	Machine Learning (ML)	Prognosis & Prediction
1008	10.1302/2633-1462.66.BJO-2024-0257.R1	2025	Sports Medicine; Arthroplasty	Machine Learning (ML)	Prognosis & Prediction

1009	N/A	2024	Arthroplasty	Machine Learning (ML)	Unclassified
1010	10.1007/s00590-019-02596-z	2020	Pediatric Orthopaedics; Spine Surgery	Machine Learning (ML)	Prognosis & Prediction
1011	10.1055/a-1365-9655	2022	Arthroplasty	Unclassified	Unclassified
1012	10.1249/01.mss.0000518712.49164.ed	2017	Unclassified	Machine Learning (ML)	Unclassified
1013	N/A	2022	Unclassified	Machine Learning (ML)	Unclassified
1014	10.1177/17543371241230731	2024	Sports Medicine; Arthroplasty	Unclassified	Diagnosis & Detection
1015	10.1007/s00586-025-09481-w	2025	Spine Surgery	Machine Learning (ML)	Prognosis & Prediction
1016	10.1016/j.jhsa.2025.02.013	2025	Trauma	Machine Learning (ML)	Unclassified
1017	10.1016/j.jhsa.2022.10.001	2022	Trauma	Machine Learning (ML)	Unclassified
1018	10.1016/j.joca.2022.10.014	2023	Sports Medicine	Natural Language Processing (NLP); Machine Learning (ML)	Prognosis & Prediction
1019	10.1186/s12891-025-08829-z	2025	Trauma	Machine Learning (ML)	Prognosis & Prediction
1020	10.1016/j.wneu.2025.124399	2025	Trauma; Arthroplasty	Machine Learning (ML)	Prognosis & Prediction
1021	10.1007/s10143-024-02803-4	2024	Spine Surgery	Deep Learning (DL)	Prognosis & Prediction
1022	10.1016/j.artd.2021.06.020	2021	Arthroplasty	Machine Learning (ML)	Prognosis & Prediction
1023	10.1186/s12891-024-08052-2	2024	Spine Surgery	Machine Learning (ML); Robotics	Unclassified
1024	10.1007/s00586-025-09474-9	2025	Spine Surgery	Machine Learning (ML)	Prognosis & Prediction
1025	10.1016/j.joca.2024.08.001	2024	Arthroplasty	Unclassified	Prognosis & Prediction
1026	10.1007/s11548-024-03087-1	2024	Arthroplasty	Deep Learning (DL)	Prognosis & Prediction
1027	10.1016/j.jse.2022.04.008	2022	Unclassified	Machine Learning (ML)	Prognosis & Prediction
1028	10.1177/2192568220956978	2022	Spine Surgery	Machine Learning (ML)	Prognosis & Prediction
1029	10.5435/JAAOS-D-23-00784	2024	Arthroplasty	Machine Learning (ML)	Prognosis & Prediction
1030	10.1007/s00586-025-09115-1	2025	Spine Surgery	Machine Learning (ML)	Prognosis & Prediction
1031	10.1016/j.asjsur.2023.05.111	2023	Arthroplasty	Deep Learning (DL)	Unclassified

1032	10.1007/s12178-025-09964-9	2025	Arthroplasty	Unclassified	Prognosis & Prediction
1033	10.1016/j.gaitpost.2022.11.041	2023	Arthroplasty	Deep Learning (DL)	Prognosis & Prediction
1034	10.1007/s11701-025-02770-y	2025	Trauma; Spine Surgery	Robotics	Prognosis & Prediction
1035	10.1016/j.nec.2023.11.006	2024	Spine Surgery	Machine Learning (ML); Robotics	Prognosis & Prediction
1036	10.1016/j.arthro.2023.07.048	2024	Trauma	Natural Language Processing (NLP)	Unclassified
1037	10.1007/s11548-025-03385-2	2025	Spine Surgery	Robotics	Unclassified
1038	10.1007/s00264-015-2839-y	2016	Sports Medicine; Trauma	Machine Learning (ML)	Diagnosis & Detection
1039	N/A	2025	Arthroplasty	Natural Language Processing (NLP)	Prognosis & Prediction
1040	10.1016/j.jor.2020.03.052	2020	Arthroplasty	Machine Learning (ML)	Prognosis & Prediction
1041	10.1016/j.otsr.2023.103694	2023	Trauma	Natural Language Processing (NLP)	Prognosis & Prediction
1042	10.1002/jeo2.70070	2024	Arthroplasty	Deep Learning (DL)	Unclassified
1043	10.1007/s00402-023-05113-4	2024	Unclassified	Natural Language Processing (NLP)	Unclassified
1044	10.1186/s42836-023-00186-3	2023	Arthroplasty	Machine Learning (ML)	Prognosis & Prediction
1045	10.1249/MSS.0000000000003610	2025	Sports Medicine; Trauma	Machine Learning (ML)	Prognosis & Prediction
1046	N/A	2021	Unclassified	Machine Learning (ML)	Prognosis & Prediction
1047	10.1097/TP.0000000000004980	2024	Trauma; Arthroplasty	Machine Learning (ML)	Unclassified
1048	10.1007/s11678-025-00852-9	2025	Arthroplasty	Unclassified	Surgical Planning
1049	10.1007/s00586-025-09018-1	2025	Trauma; Spine Surgery	Machine Learning (ML)	Prognosis & Prediction
1050	10.1007/s43465-023-00936-0	2023	Sports Medicine	Machine Learning (ML)	Prognosis & Prediction
1051	10.1016/j.wneu.2024.07.067	2024	Spine Surgery	Robotics	Prognosis & Prediction
1052	10.1097/CORR.0000000000003514	2025	Trauma	Machine Learning (ML)	Prognosis & Prediction
1053	10.1007/s00586-025-09447-y	2025	Spine Surgery	Machine Learning (ML)	Prognosis & Prediction

1054	10.1007/s00586-025-09017-2	2025	Trauma; Spine Surgery	Machine Learning (ML)	Prognosis & Prediction
1055	10.1016/j.jot.2025.08.011	2025	Trauma; Spine Surgery	Machine Learning (ML)	Prognosis & Prediction
1056	10.1186/s13018-023-04049-0	2023	Trauma	Machine Learning (ML)	Prognosis & Prediction
1057	10.1007/s00586-025-08782-4	2025	Spine Surgery	Machine Learning (ML)	Prognosis & Prediction
1058	10.1016/j.spinee.2024.02.013	2024	Spine Surgery	Natural Language Processing (NLP)	Prognosis & Prediction
1059	10.2106/JBJS.24.01466	2025	Arthroplasty	Deep Learning (DL)	Surgical Planning
1060	10.1016/j.artd.2021.03.001	2021	Arthroplasty	Machine Learning (ML)	Prognosis & Prediction
1061	10.1302/2633-1462.51.BJO-2023-0095.R1	2024	Trauma	Unclassified	Prognosis & Prediction
1062	10.1007/s11548-021-02555-2	2022	Sports Medicine; Arthroplasty	Deep Learning (DL)	Segmentation & Measurement
1063	10.1016/j.arth.2022.12.032	2023	Arthroplasty	Machine Learning (ML)	Prognosis & Prediction
1064	N/A	2025	Unclassified	Deep Learning (DL)	Segmentation & Measurement
1065	10.1007/s00586-024-08538-6	2024	Sports Medicine; Pediatric Orthopaedics; Spine Surgery	Unclassified	Prognosis & Prediction
1066	10.1245/s10434-022-11996-1	2022	Arthroplasty	Robotics	Unclassified
1067	N/A	2025	Arthroplasty	Machine Learning (ML)	Prognosis & Prediction
1068	10.1007/s11657-024-01372-9	2024	Spine Surgery	Deep Learning (DL)	Prognosis & Prediction
1069	10.1002/jeo2.70445	2025	Sports Medicine	Natural Language Processing (NLP)	Prognosis & Prediction
1070	10.3171/2025.2.SPINE24872	2025	Trauma; Spine Surgery	Machine Learning (ML)	Prognosis & Prediction
1071	10.1007/s00256-024-04830-0	2025	Sports Medicine	Machine Learning (ML); Deep Learning (DL)	Prognosis & Prediction
1072	10.1016/j.knee.2025.05.011	2025	Arthroplasty	Machine Learning (ML)	Prognosis & Prediction
1073	10.3171/2022.1.SPINE211284	2022	Spine Surgery	Machine Learning (ML); Deep Learning (DL)	Prognosis & Prediction
1074	10.1177/03635465211024964	2021	Unclassified	Machine Learning (ML)	Prognosis & Prediction
1075	10.3390/biomechanics5020022	2025	Trauma	Natural Language Processing (NLP); Machine Learning (ML)	Prognosis & Prediction

1076	10.1097/JSA.0000000000000426	2025	Sports Medicine	Unclassified	Prognosis & Prediction
1077	10.1016/j.spinee.2019.02.002	2019	Spine Surgery	Natural Language Processing (NLP); Machine Learning (ML)	Unclassified
1078	10.1177/23259671251333607	2025	Sports Medicine; Arthroplasty	Deep Learning (DL)	Segmentation & Measurement
1079	10.1007/s00167-020-06421-7	2022	Arthroplasty	Machine Learning (ML)	Prognosis & Prediction
1080	10.3171/2023.8.PEDS2352	2024	Pediatric Orthopaedics; Spine Surgery	Machine Learning (ML)	Prognosis & Prediction
1081	10.1145/3606038.3616172	2023	Sports Medicine; Arthroplasty	Deep Learning (DL)	Unclassified
1082	10.31616/asj.2018.12.4.611	2018	Trauma; Spine Surgery	Machine Learning (ML)	Unclassified
1083	10.1016/j.joca.2019.02.388	2019	Unclassified	Deep Learning (DL)	Unclassified
1084	10.1302/2046-3758.141.BJR-2024-0134.R1	2025	Trauma; Arthroplasty	Unclassified	Prognosis & Prediction
1085	10.1016/j.gaitpost.2025.02.022	2025	Unclassified	Unclassified	Unclassified
1086	10.1016/j.arthro.2025.01.064	2025	Sports Medicine; Arthroplasty	Robotics	Prognosis & Prediction
1087	10.1177/15563316251344945	2025	Unclassified	Unclassified	Diagnosis & Detection
1088	10.1097/JS9.0000000000002910	2025	Trauma; Spine Surgery	Robotics	Prognosis & Prediction
1089	10.1097/BRS.0000000000005173	2025	Spine Surgery	Machine Learning (ML)	Prognosis & Prediction
1090	N/A	2025	Arthroplasty	Deep Learning (DL)	Segmentation & Measurement
1091	10.1007/s11548-023-02856-8	2023	Arthroplasty	Machine Learning (ML)	Unclassified
1092	10.1055/a-2568-9610	2025	Trauma	Unclassified	Prognosis & Prediction
1093	10.1007/s00586-025-09476-7	2025	Spine Surgery	Machine Learning (ML)	Prognosis & Prediction
1094	10.1007/s00586-025-09547-9	2025	Spine Surgery	Deep Learning (DL)	Diagnosis & Detection
1095	10.1016/j.ocarto.2025.100571	2025	Unclassified	Machine Learning (ML)	Prognosis & Prediction
1096	10.1186/s12891-025-08726-5	2025	Trauma	Machine Learning (ML)	Prognosis & Prediction

1097	10.1007/s00586-025-09118-y	2025	Spine Surgery	Machine Learning (ML)	Prognosis & Prediction
1098	10.1007/978-981-97-2898-5_8	2024	Sports Medicine; Arthroplasty	Unclassified	Unclassified
1099	10.1016/j.artd.2024.101410	2024	Arthroplasty	Robotics	Prognosis & Prediction
1100	10.1016/j.otsr.2023.103564	2023	Trauma	Unclassified	Diagnosis & Detection
1101	10.1080/02640414.2022.2117474	2022	Sports Medicine; Trauma; Arthroplasty	Deep Learning (DL)	Prognosis & Prediction
1102	10.1007/s11548-025-03361-w	2025	Trauma; Pediatric Orthopaedics	Deep Learning (DL)	Diagnosis & Detection
1103	10.1016/j.joca.2021.12.007	2022	Arthroplasty	Machine Learning (ML)	Prognosis & Prediction
1104	10.1007/s11548-020-02150-x	2020	Trauma	Deep Learning (DL)	Prognosis & Prediction
1105	10.1016/j.joca.2025.01.001	2025	Sports Medicine	Deep Learning (DL)	Prognosis & Prediction
1106	10.1007/s11657-024-01455-7	2024	Trauma; Spine Surgery	Deep Learning (DL)	Prognosis & Prediction
1107	10.1007/s11657-023-01216-y	2023	Spine Surgery	Deep Learning (DL)	Diagnosis & Detection
1108	10.5152/j.aott.2024.23065	2024	Trauma	Unclassified	Diagnosis & Detection
1109	10.21182/jmisst.2025.02376	2025	Spine Surgery	Robotics	Unclassified
1110	10.1016/j.jos.2024.03.012	2025	Spine Surgery	Unclassified	Prognosis & Prediction
1111	10.1177/00031348231198112	2024	Unclassified	Machine Learning (ML)	Prognosis & Prediction
1112	10.1002/jor.24973	2021	Spine Surgery	Natural Language Processing (NLP); Machine Learning (ML)	Prognosis & Prediction
1113	10.1016/j.gaitpost.2021.10.028	2022	Pediatric Orthopaedics	Machine Learning (ML)	Diagnosis & Detection
1114	10.1177/21925682231202782	2025	Spine Surgery	Machine Learning (ML)	Prognosis & Prediction
1115	10.1302/2633-1462.610.BJO-2025-0075.R2	2025	Arthroplasty	Machine Learning (ML)	Prognosis & Prediction
1116	N/A	2020	Spine Surgery	Robotics	Unclassified
1117	10.14245/ns.2346776.388	2023	Trauma; Spine Surgery	Machine Learning (ML)	Prognosis & Prediction
1118	10.1016/j.arth.2020.03.019	2020	Arthroplasty	Natural Language Processing (NLP); Machine Learning (ML)	Prognosis & Prediction
1119	10.1002/jeo2.70518	2025	Unclassified	Unclassified	Unclassified

1120	10.1007/s10143-024-03003-w	2024	Trauma	Unclassified	Prognosis & Prediction
1121	10.1177/00031348251358429	2026	Trauma	Robotics	Prognosis & Prediction
1122	10.1007/978-3-031-55315-8_15	2024	Trauma	Machine Learning (ML)	Prognosis & Prediction
1123	10.1007/s00586-025-09021-6	2025	Trauma; Spine Surgery	Machine Learning (ML)	Prognosis & Prediction
1124	10.2196/39650	2023	Spine Surgery	Machine Learning (ML)	Prognosis & Prediction
1125	10.1007/s10143-022-01908-y	2022	Pediatric Orthopaedics; Spine Surgery	Natural Language Processing (NLP); Machine Learning (ML); Robotics	Unclassified
1126	10.1007/s00586-025-09489-2	2025	Spine Surgery	Machine Learning (ML)	Prognosis & Prediction
1127	10.1007/s00256-025-05034-w	2026	Sports Medicine	Deep Learning (DL)	Segmentation & Measurement
1128	10.20517/ais.2024.42	2025	Spine Surgery	Machine Learning (ML)	Prognosis & Prediction
1129	10.1093/neuros/nyaa477	2021	Spine Surgery	Machine Learning (ML)	Prognosis & Prediction
1130	10.1016/j.jot.2023.08.004	2023	Spine Surgery; Trauma; Arthroplasty	Unclassified	Unclassified
1131	10.5435/JAAOS-D-23-00839	2024	Unclassified	Natural Language Processing (NLP)	Prognosis & Prediction
1132	10.1016/j.spinee.2023.08.009	2023	Trauma; Spine Surgery	Natural Language Processing (NLP); Machine Learning (ML)	Prognosis & Prediction
1133	10.1016/j.joca.2018.02.885	2018	Sports Medicine	Deep Learning (DL)	Segmentation & Measurement
1134	10.1016/j.jor.2025.08.046	2026	Arthroplasty	Robotics	Prognosis & Prediction
1135	10.1177/2192568220959037	2021	Spine Surgery	Unclassified	Prognosis & Prediction
1136	10.1302/0301-620X.98B10.BJJ.2016.0473.R1	2016	Arthroplasty	Unclassified	Unclassified
1137	10.1007/s00256-022-04192-5	2023	Spine Surgery	Deep Learning (DL)	Unclassified
1138	10.15366/rimcafd2024.96.003	2024	Sports Medicine; Trauma	Deep Learning (DL)	Prognosis & Prediction
1139	10.1097/PXR.0000000000000233	2023	Unclassified	Machine Learning (ML)	Unclassified
1140	10.1016/j.arthro.2022.04.016	2023	Unclassified	Machine Learning (ML)	Prognosis & Prediction

1141	10.1016/j.jseint.2024.02.005	2024	Trauma; Arthroplasty	Machine Learning (ML)	Prognosis & Prediction
1142	10.1016/j.ejso.2024.108671	2024	Unclassified	Deep Learning (DL)	Unclassified
1143	10.1016/j.csm.2024.08.004	2025	Sports Medicine; Trauma	Deep Learning (DL)	Diagnosis & Detection
1144	10.1186/s12891-022-05126-x	2022	Unclassified	Unclassified	Prognosis & Prediction
1145	10.1097/CORR.0000000000002200	2022	Spine Surgery	Natural Language Processing (NLP)	Prognosis & Prediction
1146	10.1002/ksa.12031	2024	Sports Medicine; Trauma	Machine Learning (ML)	Prognosis & Prediction
1147	10.1007/s00586-025-08763-7	2025	Spine Surgery	Machine Learning (ML)	Prognosis & Prediction
1148	10.5435/JAAOS-D-22-00234	2023	Arthroplasty	Machine Learning (ML)	Prognosis & Prediction
1149	10.1186/s13018-023-04105-9	2023	Spine Surgery	Machine Learning (ML); Deep Learning (DL)	Prognosis & Prediction
1150	10.1007/s11701-025-02913-1	2025	Unclassified	Deep Learning (DL)	Surgical Planning
1151	10.1016/j.injury.2023.03.020	2023	Sports Medicine	Machine Learning (ML)	Unclassified
1152	10.1007/s00113-025-01549-y	2025	Unclassified	Unclassified	Unclassified
1153	10.3171/2022.5.SPINE22109	2022	Spine Surgery	Unclassified	Segmentation & Measurement
1154	10.1111/os.14172	2024	Unclassified	Machine Learning (ML)	Diagnosis & Detection
1155	10.2340/17453674.2024.35749	2024	Arthroplasty	Unclassified	Prognosis & Prediction
1156	10.1007/s00586-025-09304-y	2025	Spine Surgery	Machine Learning (ML)	Prognosis & Prediction
1157	10.1016/j.ejso.2025.110509	2025	Pediatric Orthopaedics	Machine Learning (ML)	Prognosis & Prediction
1158	10.1002/ksa.70141	2025	Sports Medicine; Arthroplasty	Unclassified	Prognosis & Prediction
1159	10.1016/j.eats.2022.06.009	2022	Sports Medicine	Unclassified	Surgical Planning
1160	10.1177/03635465221124258	2022	Sports Medicine; Trauma	Machine Learning (ML)	Prognosis & Prediction
1161	10.1177/10711007211015177	2021	Unclassified	Machine Learning (ML)	Diagnosis & Detection
1162	10.1007/s11548-019-02096-9	2020	Arthroplasty	Deep Learning (DL)	Diagnosis & Detection

1163	10.1007/s11548-022-02607-1	2022	Trauma	Deep Learning (DL)	Diagnosis & Detection
1164	10.1007/s00256-023-04473-7	2024	Unclassified	Deep Learning (DL)	Prognosis & Prediction
1165	10.1007/s11548-019-02105-x	2020	Unclassified	Deep Learning (DL)	Diagnosis & Detection
1166	10.1016/j.ejso.2024.108670	2025	Unclassified	Deep Learning (DL)	Unclassified
1167	10.1002/jor.25675	2024	Trauma	Machine Learning (ML)	Prognosis & Prediction
1168	10.1007/s00586-025-09151-x	2025	Trauma; Spine Surgery	Robotics; Deep Learning (DL)	Surgical Planning
1169	10.1007/s00586-023-08123-3	2024	Spine Surgery	Robotics; Deep Learning (DL)	Surgical Planning
1170	10.1002/jor.70047	2025	Arthroplasty	Deep Learning (DL)	Unclassified
1171	10.3389/fsurg.2021.663377	2021	Trauma	Machine Learning (ML)	Prognosis & Prediction
1172	10.1055/a-2717-5826	2025	Trauma	Unclassified	Prognosis & Prediction
1173	10.1007/s43465-025-01615-y	2025	Sports Medicine; Trauma	Machine Learning (ML)	Prognosis & Prediction
1174	10.1016/j.spinee.2019.10.007	2020	Spine Surgery	Natural Language Processing (NLP); Machine Learning (ML)	Prognosis & Prediction
1175	10.1007/s00586-025-09690-3	2025	Pediatric Orthopaedics; Spine Surgery	Deep Learning (DL)	Diagnosis & Detection
1176	10.1016/j.injury.2025.112893	2025	Unclassified	Machine Learning (ML)	Prognosis & Prediction
1177	10.1186/s13037-025-00440-4	2025	Spine Surgery	Natural Language Processing (NLP); Robotics	Prognosis & Prediction
1178	10.1007/s00167-021-06742-1	2022	Arthroplasty	Machine Learning (ML)	Prognosis & Prediction
1179	10.1519/JSC.0000000000003982	2021	Trauma; Pediatric Orthopaedics	Machine Learning (ML)	Prognosis & Prediction
1180	10.1007/s00586-025-09546-w	2025	Pediatric Orthopaedics	Machine Learning (ML)	Prognosis & Prediction
1181	10.2106/JBJS.21.00718	2022	Arthroplasty	Machine Learning (ML)	Prognosis & Prediction
1182	10.1016/j.otsr.2023.103734	2024	Arthroplasty	Machine Learning (ML)	Prognosis & Prediction
1183	10.3390/sports13120439	2025	Sports Medicine; Trauma	Machine Learning (ML)	Prognosis & Prediction
1184	10.1016/j.wnsx.2024.100335	2024	Spine Surgery	Robotics	Prognosis & Prediction
1185	10.1016/j.wneu.2024.10.038	2025	Spine Surgery	Machine Learning (ML)	Prognosis & Prediction

1186	10.1177/03635465241313235	2025	Sports Medicine; Trauma; Arthroplasty	Machine Learning (ML)	Prognosis & Prediction
1187	10.1007/s11701-025-02737-z	2025	Unclassified	Robotics	Unclassified
1188	10.1016/j.jse.2025.03.038	2026	Arthroplasty	Machine Learning (ML)	Surgical Planning
1189	10.1097/BRS.0000000000001804	2016	Trauma; Spine Surgery	Unclassified	Unclassified
1190	10.1016/j.spinee.2021.10.020	2022	Pediatric Orthopaedics; Trauma; Spine Surgery	Natural Language Processing (NLP); Deep Learning (DL)	Prognosis & Prediction
1191	10.1177/10556656251393077	2025	Unclassified	Unclassified	Prognosis & Prediction
1192	10.1302/2633-1462	2025	Arthroplasty	Machine Learning (ML)	Prognosis & Prediction
1193	10.1016/j.ocarto.2024.100440	2024	Arthroplasty	Machine Learning (ML)	Prognosis & Prediction
1194	10.1016/j.ocarto.2025.100603	2025	Unclassified	Machine Learning (ML)	Prognosis & Prediction
1195	10.1016/j.arth.2019.07.022	2019	Sports Medicine; Arthroplasty	Machine Learning (ML)	Segmentation & Measurement
1196	10.3390/biomechanics5040094	2025	Sports Medicine	Machine Learning (ML)	Prognosis & Prediction
1197	10.1111/sms.14693	2024	Sports Medicine; Arthroplasty	Unclassified	Surgical Planning
1198	10.1016/j.arthro.2024.11.087	2025	Sports Medicine	Unclassified	Prognosis & Prediction
1199	10.1227/neu.0000000000002180	2023	Spine Surgery	Machine Learning (ML)	Prognosis & Prediction
1200	10.1016/j.wnsx.2024.100416	2025	Spine Surgery	Robotics	Prognosis & Prediction
1201	10.1016/j.arth.2022.09.007	2023	Arthroplasty	Natural Language Processing (NLP); Machine Learning (ML)	Prognosis & Prediction
1202	10.1016/j.knee.2025.01.002	2025	Arthroplasty	Natural Language Processing (NLP); Machine Learning (ML); Robotics	Diagnosis & Detection
1203	10.1007/s00256-020-03429-5	2020	Sports Medicine; Pediatric Orthopaedics	Machine Learning (ML)	Unclassified
1204	10.1097/BRS.0000000000004416	2023	Spine Surgery	Machine Learning (ML)	Prognosis & Prediction
1205	10.1227/neu.0000000000002902	2024	Unclassified	Machine Learning (ML)	Prognosis & Prediction

1206	10.3389/fsurg.2022.1037978	2023	Spine Surgery	Natural Language Processing (NLP); Machine Learning (ML)	Unclassified
1207	10.2106/JBJS.21.01229	2022	Arthroplasty	Deep Learning (DL)	Segmentation & Measurement
1208	10.1002/jso.28109	2025	Unclassified	Natural Language Processing (NLP)	Prognosis & Prediction
1209	10.1055/s-0040-1713658	2022	Sports Medicine; Trauma	Machine Learning (ML)	Prognosis & Prediction
1210	10.1177/21925682251323277	2025	Spine Surgery	Robotics	Prognosis & Prediction
1211	10.15366/rimcafd2022.85.016	2022	Sports Medicine; Arthroplasty	Deep Learning (DL)	Unclassified
1212	10.5152/j.aott.2024.23115	2024	Spine Surgery; Trauma; Arthroplasty	Unclassified	Prognosis & Prediction
1213	10.1007/s00586-024-08579-x	2025	Trauma; Spine Surgery	Machine Learning (ML)	Prognosis & Prediction
1214	10.1097/BOT.0000000000002887	2024	Trauma	Machine Learning (ML)	Unclassified
1215	10.31616/asj.2025.0198	2025	Spine Surgery	Robotics	Prognosis & Prediction
1216	10.1186/s13018-023-04280-9	2023	Sports Medicine; Trauma	Robotics	Unclassified
1217	10.1016/j.spinee.2020.02.003	2021	Spine Surgery	Machine Learning (ML)	Prognosis & Prediction
1218	10.1152/japplphysiol.00366.2017	2018	Sports Medicine; Trauma; Arthroplasty	Natural Language Processing (NLP)	Prognosis & Prediction
1219	10.1007/s00132-023-04402-5	2023	Arthroplasty	Unclassified	Unclassified
1220	10.1302/0301-620X.104B8.BJJ-2022-0120.R2	2022	Arthroplasty	Unclassified	Diagnosis & Detection
1221	10.3171/2021.2.FOCUS201113	2021	Spine Surgery	Natural Language Processing (NLP); Machine Learning (ML)	Prognosis & Prediction
1222	10.1177/10225536251331664	2025	Unclassified	Machine Learning (ML)	Prognosis & Prediction
1223	10.1007/s00586-023-08084-7	2024	Spine Surgery	Machine Learning (ML)	Prognosis & Prediction
1224	10.1016/j.spinee.2020.10.006	2021	Spine Surgery	Machine Learning (ML)	Prognosis & Prediction
1225	10.62713/aic.4036	2025	Trauma	Unclassified	Prognosis & Prediction

1226	10.1002/jor.25314	2022	Sports Medicine; Arthroplasty	Natural Language Processing (NLP); Deep Learning (DL)	Surgical Planning
1227	10.3389/fsurg.2025.1631651	2025	Trauma; Spine Surgery	Machine Learning (ML)	Prognosis & Prediction
1228	10.1016/j.spinee.2023.01.023	2023	Spine Surgery	Natural Language Processing (NLP); Machine Learning (ML)	Prognosis & Prediction
1229	10.1002/ohn.1365	2025	Unclassified	Robotics; Deep Learning (DL)	Prognosis & Prediction
1230	10.1089/sur.2023.274	2024	Unclassified	Machine Learning (ML)	Prognosis & Prediction
1231	10.1016/j.wneu.2020.11.037	2021	Spine Surgery	Machine Learning (ML)	Prognosis & Prediction
1232	10.14245/ns.2346296.148	2023	Unclassified	Machine Learning (ML)	Prognosis & Prediction
1233	10.1111/os.70112	2025	Spine Surgery; Arthroplasty	Robotics	Surgical Planning
1234	10.1186/s12891-024-08215-1	2024	Trauma; Arthroplasty	Machine Learning (ML)	Prognosis & Prediction
1235	10.1097/BSD.0000000000001295	2022	Sports Medicine; Spine Surgery	Machine Learning (ML)	Prognosis & Prediction
1236	10.1007/s00586-024-08409-0	2024	Sports Medicine; Spine Surgery; Arthroplasty	Deep Learning (DL)	Diagnosis & Detection
1237	10.1186/s13018-024-05062-7	2024	Trauma; Spine Surgery	Machine Learning (ML)	Prognosis & Prediction
1238	10.1186/s13018-021-02294-9	2021	Arthroplasty	Unclassified	Prognosis & Prediction
1239	10.14245/ns.2342434.217	2023	Trauma; Spine Surgery	Machine Learning (ML)	Prognosis & Prediction
1240	10.52312/jdrs.2023.1059	2023	Arthroplasty	Unclassified	Prognosis & Prediction
1241	10.1245/s10434-025-18448-6	2026	Arthroplasty	Machine Learning (ML)	Prognosis & Prediction
1242	10.1007/s00264-022-05641-5	2023	Arthroplasty	Machine Learning (ML)	Prognosis & Prediction
1243	10.1097/SCS.0000000000007256	2021	Unclassified	Unclassified	Unclassified
1244	10.15621/ijphy/2025/v12i1s/1607	2025	Unclassified	Unclassified	Prognosis & Prediction
1245	10.1016/j.arth.2023.02.076	2023	Arthroplasty	Robotics	Prognosis & Prediction
1246	10.1007/s00586-025-09340-8	2025	Pediatric Orthopaedics; Spine Surgery; Arthroplasty	Deep Learning (DL)	Prognosis & Prediction
1247	10.1016/j.gaitpost.2022.05.015	2022	Spine Surgery	Machine Learning (ML)	Segmentation & Measurement
1248	10.20517/ais.2023.12	2023	Trauma	Unclassified	Diagnosis & Detection

1249	10.1007/s00276-022-03076-6	2023	Spine Surgery	Machine Learning (ML)	Prognosis & Prediction
1250	10.1007/s00586-021-07036-3	2022	Spine Surgery	Natural Language Processing (NLP); Machine Learning (ML)	Diagnosis & Detection
1251	10.1177/21925682251336714	2026	Spine Surgery	Machine Learning (ML)	Prognosis & Prediction
1252	10.1016/j.wneu.2025.124513	2025	Spine Surgery	Robotics	Prognosis & Prediction
1253	10.3389/fsurg.2025.1591671	2025	Trauma	Machine Learning (ML)	Prognosis & Prediction
1254	10.1016/j.spinee.2020.09.007	2021	Spine Surgery	Natural Language Processing (NLP); Robotics	Unclassified
1255	10.1007/s43390-022-00618-1	2023	Pediatric Orthopaedics; Spine Surgery	Machine Learning (ML)	Prognosis & Prediction
1256	10.1016/j.wneu.2020.11.122	2021	Sports Medicine; Trauma	Unclassified	Unclassified
1257	10.1002/jor.25319	2022	Sports Medicine; Trauma; Arthroplasty	Machine Learning (ML)	Unclassified
1258	10.1080/14763141.2021.1947358	2023	Sports Medicine; Trauma	Machine Learning (ML)	Prognosis & Prediction
1259	10.1007/s00586-022-07431-4	2023	Trauma; Spine Surgery	Machine Learning (ML)	Prognosis & Prediction
1260	10.1186/s13018-023-03522-0	2023	Arthroplasty	Machine Learning (ML)	Prognosis & Prediction
1261	10.1002/hed.27797	2024	Unclassified	Unclassified	Prognosis & Prediction
1262	10.1007/s11657-025-01524-5	2025	Trauma; Spine Surgery	Unclassified	Diagnosis & Detection
1263	10.1136/bmjsem-2023-001859	2024	Trauma	Machine Learning (ML)	Prognosis & Prediction
1264	10.1007/s11548-025-03330-3	2025	Unclassified	Deep Learning (DL)	Prognosis & Prediction
1265	10.1016/j.jse.2024.04.008	2025	Trauma; Arthroplasty	Natural Language Processing (NLP); Machine Learning (ML)	Prognosis & Prediction
1266	10.3389/fspor.2025.1607212	2025	Arthroplasty	Machine Learning (ML)	Prognosis & Prediction
1267	10.2340/17453674.2024.40949	2024	Trauma	Machine Learning (ML); Deep Learning (DL)	Diagnosis & Detection
1268	10.1002/jso.26708	2022	Unclassified	Machine Learning (ML)	Prognosis & Prediction
1269	10.1186/s13018-021-02818-3	2021	Trauma	Machine Learning (ML)	Unclassified

1270	10.1007/s00586-023-07887-y	2023	Trauma; Spine Surgery	Machine Learning (ML)	Prognosis & Prediction
1271	10.15621/ijphy/2025/v12i3/1861	2025	Spine Surgery	Unclassified	Diagnosis & Detection
1272	10.1016/j.gaitpost.2024.06.007	2024	Arthroplasty	Natural Language Processing (NLP)	Diagnosis & Detection
1273	10.1002/rcs.2184	2021	Unclassified	Machine Learning (ML); Robotics	Diagnosis & Detection
1274	10.1016/j.knee.2024.04.002	2024	Arthroplasty	Unclassified	Prognosis & Prediction
1275	10.1007/978-3-319-60822-8_9	2018	Arthroplasty	Machine Learning (ML)	Unclassified
1276	10.5435/JAAOSGlobal-D-24-00053	2024	Spine Surgery; Trauma; Arthroplasty	Machine Learning (ML)	Unclassified
1277	10.1097/TA.0000000000004624	2025	Trauma	Machine Learning (ML)	Prognosis & Prediction
1278	10.1097/CORR.0000000000002857	2024	Trauma; Arthroplasty	Machine Learning (ML)	Prognosis & Prediction
1279	10.1016/j.jse.2021.10.032	2022	Arthroplasty	Machine Learning (ML)	Prognosis & Prediction
1280	10.1097/BRS.0000000000005278	2025	Spine Surgery	Machine Learning (ML)	Prognosis & Prediction
1281	10.1007/s12178-023-09880-w	2024	Arthroplasty	Machine Learning (ML)	Prognosis & Prediction
1282	10.1007/s00586-020-06577-3	2021	Spine Surgery	Deep Learning (DL)	Diagnosis & Detection
1283	10.1016/j.joca.2024.05.013	2024	Sports Medicine; Arthroplasty	Unclassified	Prognosis & Prediction
1284	10.1177/23259671251358390	2025	Arthroplasty	Machine Learning (ML)	Prognosis & Prediction
1285	10.1016/j.arth.2024.03.042	2024	Arthroplasty	Robotics	Prognosis & Prediction
1286	10.2106/JBJS.16.00167	2017	Arthroplasty	Unclassified	Prognosis & Prediction
1287	10.1097/BSD.0000000000001677	2024	Sports Medicine; Trauma; Spine Surgery	Unclassified	Prognosis & Prediction
1288	10.1186/s12891-024-07528-5	2024	Spine Surgery	Machine Learning (ML)	Prognosis & Prediction
1289	10.1007/s00586-019-06054-6	2020	Trauma; Spine Surgery	Robotics	Prognosis & Prediction
1290	10.3171/2022.1.FOCUS21721	2022	Trauma	Unclassified	Surgical Planning

1291	10.1016/j.injury.2024.111902	2024	Trauma	Machine Learning (ML)	Prognosis & Prediction
1292	10.1111/ajt.15850	2020	Arthroplasty	Machine Learning (ML)	Diagnosis & Detection
1293	10.3928/1081597X-20230831-03	2023	Sports Medicine	Unclassified	Prognosis & Prediction
1294	10.1016/j.ocarto.2022.100236	2022	Unclassified	Machine Learning (ML)	Prognosis & Prediction
1295	10.5312/wjo.v14.i1.1	2023	Unclassified	Unclassified	Diagnosis & Detection
1296	10.1016/j.arth.2025.05.075	2025	Trauma; Arthroplasty	Natural Language Processing (NLP)	Prognosis & Prediction
1297	10.1097/TA.0000000000004675	2025	Trauma; Arthroplasty	Machine Learning (ML)	Prognosis & Prediction
1298	10.1007/s11657-025-01579-4	2025	Spine Surgery; Trauma; Arthroplasty	Unclassified	Prognosis & Prediction
1299	10.1249/MSS.0000000000001974	2019	Trauma	Machine Learning (ML)	Prognosis & Prediction
1300	10.1007/s00586-024-08284-9	2024	Spine Surgery	Machine Learning (ML)	Prognosis & Prediction
1301	10.3171/2023.3.FOCUS2345	2023	Spine Surgery	Machine Learning (ML); Robotics	Unclassified
1302	10.1177/10225536251357767	2025	Unclassified	Unclassified	Unclassified
1303	10.1080/02640414.2024.2435729	2025	Sports Medicine; Trauma	Machine Learning (ML)	Prognosis & Prediction
1304	10.1097/BRS.0000000000005087	2024	Sports Medicine; Spine Surgery; Arthroplasty	Machine Learning (ML)	Prognosis & Prediction
1305	10.1002/jeo2.12103	2024	Unclassified	Unclassified	Unclassified
1306	10.1007/s00586-025-09540-2	2025	Spine Surgery	Deep Learning (DL)	Diagnosis & Detection
1307	10.1016/j.injury.2022.08.049	2022	Trauma	Robotics	Unclassified
1308	10.2106/JBJS.20.00903	2021	Trauma; Pediatric Orthopaedics	Machine Learning (ML)	Prognosis & Prediction
1309	10.1007/s00167-023-07644-0	2023	Sports Medicine; Arthroplasty	Deep Learning (DL)	Surgical Planning
1310	10.2106/JBJS.OA.25.00145	2025	Unclassified	Natural Language Processing (NLP)	Diagnosis & Detection
1311	10.1097/GOX.0000000000006650	2025	Unclassified	Unclassified	Segmentation & Measurement

1312	10.1177/21925682211069933	2023	Spine Surgery	Machine Learning (ML)	Prognosis & Prediction
1313	10.1186/s13018-025-06208-x	2025	Arthroplasty	Unclassified	Prognosis & Prediction
1314	10.1177/23259671211053326	2021	Sports Medicine	Machine Learning (ML)	Prognosis & Prediction
1315	10.1097/BSD.0000000000001575	2024	Spine Surgery	Machine Learning (ML)	Prognosis & Prediction
1316	10.1016/j.spinee.2019.12.019	2020	Spine Surgery	Unclassified	Prognosis & Prediction
1317	10.1007/s00586-024-08449-6	2024	Spine Surgery	Machine Learning (ML)	Prognosis & Prediction
1318	10.1302/0301-620X.105B5.BJJ-2022-1123	2023	Arthroplasty	Unclassified	Prognosis & Prediction
1319	10.1002/jsp2.70136	2025	Spine Surgery	Robotics	Prognosis & Prediction
1320	10.1002/ohn.70061	2025	Unclassified	Natural Language Processing (NLP)	Unclassified
1321	10.1007/s00256-024-04690-8	2024	Sports Medicine; Spine Surgery; Trauma; Arthroplasty	Deep Learning (DL)	Diagnosis & Detection
1322	10.1007/s00586-020-06406-7	2020	Spine Surgery	Deep Learning (DL)	Segmentation & Measurement
1323	10.1007/s00256-024-04679-3	2024	Pediatric Orthopaedics	Deep Learning (DL)	Diagnosis & Detection
1324	10.1177/10225536251357645	2025	Unclassified	Unclassified	Surgical Planning
1325	10.1177/21925682251340033	2026	Trauma; Spine Surgery	Natural Language Processing (NLP)	Unclassified
1326	10.3390/sports13020046	2025	Sports Medicine; Trauma	Natural Language Processing (NLP); Machine Learning (ML)	Prognosis & Prediction
1327	10.1186/s12891-023-06218-y	2023	Trauma	Machine Learning (ML)	Prognosis & Prediction
1328	10.1002/rcs.2464	2023	Arthroplasty	Robotics; Deep Learning (DL)	Prognosis & Prediction
1329	10.1302/0301-620X.105B.BJJ-2023-0272	2023	Unclassified	Unclassified	Unclassified
1330	10.1186/s12891-024-07758-7	2024	Unclassified	Machine Learning (ML)	Unclassified
1331	10.15366/rimcafd2025.99.023	2025	Sports Medicine; Arthroplasty	Deep Learning (DL)	Prognosis & Prediction

1332	10.1097/BRS.0000000000005342	2025	Spine Surgery; Arthroplasty	Machine Learning (ML)	Prognosis & Prediction
1333	10.1002/jor.25566	2023	Unclassified	Unclassified	Unclassified
1334	10.1097/BTF.0000000000000434	2025	Trauma	Unclassified	Prognosis & Prediction
1335	10.1016/j.joca.2020.11.003	2021	Sports Medicine	Unclassified	Unclassified
1336	10.1097/BRS.0000000000004490	2023	Spine Surgery	Machine Learning (ML)	Prognosis & Prediction
1337	10.2106/JBJS.RVW.23.00158	2023	Arthroplasty	Robotics	Prognosis & Prediction
1338	10.1007/s11548-020-02194-z	2020	Arthroplasty	Robotics	Surgical Planning
1339	10.3171/2021.3.SPINE201534	2022	Trauma; Spine Surgery	Machine Learning (ML)	Prognosis & Prediction
1340	10.1007/s00256-024-04786-1	2025	Sports Medicine; Arthroplasty	Deep Learning (DL)	Segmentation & Measurement
1341	10.2106/JBJS.23.00293	2023	Unclassified	Unclassified	Unclassified
1342	10.1097/CORR.0000000000002658	2023	Unclassified	Unclassified	Unclassified
1343	10.1177/23259671211048188	2021	Sports Medicine; Trauma; Arthroplasty	Unclassified	Prognosis & Prediction
1344	10.1097/CORR.0000000000003235	2024	Trauma; Arthroplasty	Unclassified	Prognosis & Prediction
1345	10.1177/21925682241256949	2025	Sports Medicine; Spine Surgery	Natural Language Processing (NLP)	Prognosis & Prediction
1346	10.1016/j.jcms.2023.09.003	2023	Trauma	Deep Learning (DL)	Segmentation & Measurement
1347	10.1007/s00586-023-07562-2	2023	Sports Medicine; Spine Surgery	Machine Learning (ML); Deep Learning (DL)	Prognosis & Prediction
1348	10.1007/s00264-023-05830-w	2023	Unclassified	Unclassified	Unclassified
1349	10.20517/ais.2024.50	2024	Trauma	Natural Language Processing (NLP)	Prognosis & Prediction
1350	10.5152/j.aott.2025.25418	2025	Unclassified	Unclassified	Unclassified
1351	N/A	2018	Unclassified	Unclassified	Unclassified
1352	10.1007/s43465-024-01295-0	2025	Trauma	Unclassified	Prognosis & Prediction
1353	10.1016/j.arth.2023.04.040	2023	Arthroplasty	Machine Learning (ML); Robotics	Prognosis & Prediction

1354	10.1016/j.jor.2021.03.001	2021	Arthroplasty	Machine Learning (ML)	Prognosis & Prediction
1355	10.22038/ABJS.2025.90701.4108	2025	Unclassified	Unclassified	Unclassified
1356	10.1503/cjs.010423	2025	Arthroplasty	Natural Language Processing (NLP); Robotics	Unclassified
1357	10.1177/21925682241241241	2024	Spine Surgery	Natural Language Processing (NLP)	Unclassified
1358	10.1186/s41984-025-00466-x	2025	Spine Surgery	Unclassified	Prognosis & Prediction
1359	10.14245/ns.2143080.540	2022	Spine Surgery	Natural Language Processing (NLP)	Diagnosis & Detection
1360	10.1007/s00586-022-07307-7	2022	Spine Surgery	Machine Learning (ML)	Prognosis & Prediction
1361	10.1007/s00264-022-05454-6	2022	Unclassified	Unclassified	Unclassified
1362	10.1186/s12891-025-08631-x	2025	Trauma	Machine Learning (ML); Deep Learning (DL)	Prognosis & Prediction
1363	10.1016/j.otsr.2025.104338	2025	Trauma; Arthroplasty	Unclassified	Prognosis & Prediction
1364	10.1007/s00586-019-06158-z	2019	Spine Surgery	Machine Learning (ML)	Diagnosis & Detection
1365	10.2106/JBJS.24.00416	2024	Unclassified	Unclassified	Unclassified
1366	10.2106/JBJS.24.00633	2025	Unclassified	Unclassified	Unclassified
1367	10.5435/JAAOSGlobal-D-24-00023	2024	Arthroplasty	Machine Learning (ML)	Prognosis & Prediction
1368	10.1177/10711007241232288	2024	Unclassified	Unclassified	Unclassified
1369	10.1302/2046-3758.139.BJR-2024-0135.R1	2024	Trauma	Unclassified	Unclassified
1370	10.1016/j.spinee.2022.07.089	2022	Spine Surgery	Natural Language Processing (NLP); Machine Learning (ML)	Prognosis & Prediction
1371	10.1097/CORR.0000000000003084	2024	Unclassified	Unclassified	Unclassified
1372	10.3171/2018.12.SPINE181397	2019	Spine Surgery	Machine Learning (ML); Robotics	Segmentation & Measurement
1373	10.1007/s43390-025-01086-z	2025	Sports Medicine; Trauma	Machine Learning (ML); Robotics	Prognosis & Prediction
1374	10.1097/CORR.0000000000002725	2023	Unclassified	Unclassified	Unclassified

1375	10.1007/s00264-023-05936-1	2023	Unclassified	Unclassified	Unclassified
1376	10.1002/jor.24567	2020	Sports Medicine; Trauma; Arthroplasty	Unclassified	Unclassified
1377	10.1016/j.arth.2021.09.005	2022	Arthroplasty	Machine Learning (ML)	Prognosis & Prediction
1378	10.1186/s13018-025-06024-3	2025	Pediatric Orthopaedics; Spine Surgery; Trauma; Arthroplasty	Unclassified	Prognosis & Prediction
1379	10.1002/jor.70020	2025	Pediatric Orthopaedics; Arthroplasty	Deep Learning (DL)	Diagnosis & Detection
1380	10.1007/s00464-025-12304-0	2025	Unclassified	Robotics	Prognosis & Prediction
1381	10.1007/s00586-024-08235-4	2024	Trauma; Spine Surgery	Machine Learning (ML); Deep Learning (DL)	Prognosis & Prediction
1382	10.1097/BCO.0000000000000846	2020	Unclassified	Unclassified	Prognosis & Prediction
1383	10.1016/j.injury.2025.112467	2025	Unclassified	Unclassified	Unclassified
1384	10.1016/j.asjsur.2023.07.025	2023	Unclassified	Unclassified	Unclassified
1385	10.1227/neu.0000000000002924	2024	Spine Surgery; Arthroplasty	Machine Learning (ML)	Prognosis & Prediction
1386	10.1007/s00586-019-05936-z	2019	Spine Surgery	Machine Learning (ML)	Prognosis & Prediction
1387	10.1010/j.wneu.2025.124302	2025	Spine Surgery; Arthroplasty	Robotics	Surgical Planning
1388	10.3389/fsurg.2022.952539	2022	Unclassified	Natural Language Processing (NLP); Machine Learning (ML); Robotics	Diagnosis & Detection
1389	10.1007/s11548-024-03156-5	2024	Sports Medicine; Trauma	Unclassified	Prognosis & Prediction
1390	10.1111/ans.18044	2022	Trauma	Natural Language Processing (NLP); Machine Learning (ML)	Prognosis & Prediction
1391	10.1186/s42836-023-00220-4	2023	Unclassified	Robotics	Prognosis & Prediction
1392	10.1016/j.joca.2023.09.001	2023	Trauma; Arthroplasty	Machine Learning (ML)	Prognosis & Prediction
1393	10.1016/j.gaitpost.2020.10.026	2021	Arthroplasty	Deep Learning (DL)	Segmentation & Measurement
1394	10.1016/j.jor.2022.08.018	2022	Unclassified	Robotics	Unclassified
1395	10.1093/bjsopen/zrab070	2021	Unclassified	Natural Language Processing (NLP); Machine Learning (ML)	Unclassified

1396	10.1302/2046-3758.1410.BJR-2024-0353.R2	2025	Sports Medicine	Machine Learning (ML)	Segmentation & Measurement
1397	10.62713/aic.3367	2025	Spine Surgery	Unclassified	Prognosis & Prediction
1398	10.3389/fsurg.2023.1247527	2023	Spine Surgery	Deep Learning (DL)	Surgical Planning
1399	10.1186/s12893-025-03285-x	2025	Unclassified	Unclassified	Prognosis & Prediction
1400	10.1007/s00113-022-01270-0	2023	Trauma; Arthroplasty	Natural Language Processing (NLP); Robotics	Unclassified
1401	10.1007/s00256-024-04726-z	2025	Sports Medicine	Unclassified	Prognosis & Prediction
1402	10.3390/jfmk10030286	2025	Sports Medicine; Trauma; Arthroplasty	Machine Learning (ML)	Prognosis & Prediction
1403	10.1001/jamasurg.2019.2979	2019	Arthroplasty	Machine Learning (ML)	Prognosis & Prediction
1404	10.2106/JBJS.OA.23.00103	2023	Unclassified	Natural Language Processing (NLP)	Unclassified
1405	10.1016/j.jcms.2023.08.001	2023	Unclassified	Unclassified	Unclassified
1406	10.1186/s43019-023-00209-y	2024	Sports Medicine; Arthroplasty	Deep Learning (DL)	Segmentation & Measurement
1407	10.1007/s00132-024-04568-6	2024	Trauma; Arthroplasty	Machine Learning (ML)	Prognosis & Prediction
1408	10.1016/j.WNEU.2023.01.079	2023	Spine Surgery	Unclassified	Prognosis & Prediction
1409	10.1007/s00256-024-04769-2	2025	Pediatric Orthopaedics	Deep Learning (DL)	Prognosis & Prediction
1410	10.1097/CORR.0000000000003442	2025	Trauma; Arthroplasty	Machine Learning (ML)	Prognosis & Prediction
1411	10.62713/aic.4244	2025	Trauma; Arthroplasty	Machine Learning (ML)	Prognosis & Prediction
1412	10.1055/a-2389-5207	2025	Trauma; Spine Surgery	Unclassified	Prognosis & Prediction
1413	10.1186/s12891-022-05927-0	2022	Spine Surgery	Deep Learning (DL)	Diagnosis & Detection
1414	10.1007/s11548-019-02043-8	2019	Trauma; Arthroplasty	Deep Learning (DL)	Prognosis & Prediction
1415	10.1016/j.spinee.2019.09.003	2020	Spine Surgery	Natural Language Processing (NLP); Machine Learning (ML)	Prognosis & Prediction
1416	10.1097/CORR.0000000000002820	2023	Trauma	Unclassified	Prognosis & Prediction

1417	10.1007/s11548-022-02561-y	2022	Spine Surgery	Robotics	Prognosis & Prediction
1418	10.1227/NEU.0000000000001749	2022	Unclassified	Machine Learning (ML)	Prognosis & Prediction
1419	10.1016/j.jse.2023.08.005	2024	Arthroplasty	Machine Learning (ML)	Prognosis & Prediction
1420	10.31616/asj.2022.0376	2022	Spine Surgery	Machine Learning (ML); Robotics	Prognosis & Prediction
1421	10.1186/s12891-023-06446-2	2023	Spine Surgery	Machine Learning (ML)	Prognosis & Prediction
1422	10.1002/jeo2.70477	2025	Arthroplasty	Machine Learning (ML)	Prognosis & Prediction
1423	10.1097/CORR.0000000000003470	2025	Unclassified	Unclassified	Unclassified
1424	10.1016/j.jot.2025.06.011	2025	Unclassified	Unclassified	Diagnosis & Detection
1425	10.1123/jab.2024-0263	2025	Sports Medicine; Arthroplasty	Machine Learning (ML)	Prognosis & Prediction
1426	10.1097/BOT.0000000000002070	2021	Trauma	Machine Learning (ML)	Prognosis & Prediction
1427	10.1007/s00402-025-06057-7	2025	Arthroplasty	Unclassified	Prognosis & Prediction
1428	10.17816/2311-2905-1731	2022	Spine Surgery	Unclassified	Segmentation & Measurement
1429	10.1016/j.gaitpost.2025.04.016	2025	Arthroplasty	Unclassified	Diagnosis & Detection
1430	10.1007/s00586-025-08741-z	2025	Spine Surgery	Machine Learning (ML)	Prognosis & Prediction
1431	10.1097/CORR.0000000000003559	2025	Unclassified	Unclassified	Unclassified
1432	10.1007/s11548-021-02395-0	2021	Spine Surgery	Natural Language Processing (NLP); Robotics; Deep Learning (DL)	Surgical Planning
1433	10.1080/14763141.2025.2526702	2025	Arthroplasty	Deep Learning (DL)	Unclassified
1434	10.1186/s12891-025-09348-7	2025	Arthroplasty	Unclassified	Prognosis & Prediction
1435	10.22603/ssrr.2024-0119	2025	Spine Surgery	Machine Learning (ML)	Segmentation & Measurement
1436	10.1007/s43465-024-01152-0	2024	Unclassified	Machine Learning (ML)	Prognosis & Prediction
1437	10.1227/neu.0000000000003249	2025	Pediatric Orthopaedics; Spine Surgery	Natural Language Processing (NLP)	Diagnosis & Detection

1438	10.1016/j.wneu.2023.02.064	2023	Spine Surgery	Machine Learning (ML)	Prognosis & Prediction
1439	10.1111/os.70162	2025	Spine Surgery	Unclassified	Prognosis & Prediction
1440	10.1002/jor.25127	2022	Sports Medicine; Arthroplasty	Deep Learning (DL)	Segmentation & Measurement
1441	10.1097/CORR.0000000000003527	2025	Unclassified	Unclassified	Unclassified
1442	10.1097/JS9.0000000000001282	2024	Spine Surgery	Unclassified	Unclassified
1443	10.1016/j.gaitpost.2023.10.023	2024	Trauma	Machine Learning (ML)	Prognosis & Prediction
1444	10.1177/23259671241236496	2024	Sports Medicine; Pediatric Orthopaedics	Natural Language Processing (NLP); Machine Learning (ML)	Prognosis & Prediction
1445	10.1177/17531934251326153	2025	Trauma; Arthroplasty	Robotics	Unclassified
1446	10.1002/ksa.12306	2024	Arthroplasty	Robotics	Prognosis & Prediction
1447	10.14245/ns.2347340.670	2024	Spine Surgery	Machine Learning (ML)	Prognosis & Prediction
1448	10.1016/j.otsr.2025.104225	2025	Unclassified	Unclassified	Prognosis & Prediction
1449	10.2340/17453674.2024.41911	2024	Pediatric Orthopaedics	Machine Learning (ML)	Prognosis & Prediction
1450	10.1097/BRS.0000000000004664	2023	Spine Surgery	Machine Learning (ML)	Prognosis & Prediction
1451	10.1177/23259671241257507	2024	Arthroplasty	Machine Learning (ML)	Prognosis & Prediction
1452	10.1007/s43390-024-00825-y	2024	Spine Surgery	Unclassified	Surgical Planning
1453	10.3171/2020.10.SPINE201354	2021	Spine Surgery	Machine Learning (ML)	Prognosis & Prediction
1454	10.1097/BRS.0000000000004159	2022	Sports Medicine; Spine Surgery; Arthroplasty	Machine Learning (ML)	Prognosis & Prediction
1455	10.1002/jeo2.70097	2024	Arthroplasty	Deep Learning (DL)	Prognosis & Prediction
1456	10.1249/MSS.0000000000001881	2019	Sports Medicine; Trauma	Machine Learning (ML)	Prognosis & Prediction
1457	10.1007/s11548-024-03254-4	2025	Pediatric Orthopaedics	Machine Learning (ML); Deep Learning (DL)	Prognosis & Prediction
1458	10.1007/s43465-025-01622-z	2025	Trauma	Unclassified	Prognosis & Prediction

1459	10.1007/s11548-023-02959-2	2023	Arthroplasty	Robotics; Deep Learning (DL)	Unclassified
1460	10.2106/JBJS.24.00316	2025	Arthroplasty	Deep Learning (DL)	Segmentation & Measurement
1461	10.31616/asj.2025.0042	2025	Spine Surgery	Machine Learning (ML); Robotics	Prognosis & Prediction
1462	10.3389/fsurg.2022.1060691	2023	Trauma	Machine Learning (ML)	Prognosis & Prediction
1463	10.5435/JAAOSGlobal-D-24-00289	2025	Unclassified	Natural Language Processing (NLP)	Prognosis & Prediction
1464	10.1007/s00586-025-09549-7	2025	Trauma; Spine Surgery	Machine Learning (ML)	Prognosis & Prediction
1465	10.1016/j.ocarto.2024.100556	2025	Sports Medicine	Deep Learning (DL)	Prognosis & Prediction
1466	10.1007/s00256-023-04310-x	2023	Sports Medicine	Unclassified	Prognosis & Prediction
1467	10.3389/fsurg.2023.1047558	2023	Arthroplasty	Machine Learning (ML)	Prognosis & Prediction
1468	10.3171/2020.8.FOCUS20610	2020	Spine Surgery	Machine Learning (ML)	Prognosis & Prediction
1469	10.1080/24699322.2023.2211728	2023	Unclassified	Robotics; Deep Learning (DL)	Surgical Planning
1470	10.1177/21925682211019361	2023	Spine Surgery	Machine Learning (ML)	Prognosis & Prediction
1471	10.1016/j.ijsu.2020.08.036	2020	Spine Surgery	Deep Learning (DL)	Prognosis & Prediction
1472	10.1002/ksa.70191	2025	Unclassified	Deep Learning (DL)	Diagnosis & Detection
1473	10.1016/j.joca.2019.04.008	2019	Sports Medicine; Arthroplasty	Machine Learning (ML)	Unclassified
1474	10.3171/2023.3.FOCUS2352	2023	Unclassified	Machine Learning (ML)	Prognosis & Prediction
1475	10.1016/j.injury.2022.11.031	2023	Trauma	Machine Learning (ML)	Prognosis & Prediction
1476	10.1007/s00264-024-06102-x	2024	Unclassified	Unclassified	Unclassified
1477	10.2340/17453674.2025.44248	2025	Trauma	Machine Learning (ML)	Prognosis & Prediction
1478	10.1016/j.spinee.2023.11.010	2024	Spine Surgery	Natural Language Processing (NLP); Machine Learning (ML)	Prognosis & Prediction
1479	10.1177/00031348211047505	2023	Unclassified	Unclassified	Prognosis & Prediction

1480	10.1002/jor.25390	2023	Sports Medicine	Deep Learning (DL)	Diagnosis & Detection
1481	10.5435/JAAOS-D-24-00438	2025	Unclassified	Natural Language Processing (NLP)	Prognosis & Prediction
1482	10.3389/fsurg.2023.1114922	2023	Sports Medicine; Arthroplasty	Machine Learning (ML)	Prognosis & Prediction
1483	10.1007/s11548-024-03110-5	2024	Unclassified	Deep Learning (DL)	Surgical Planning
1484	10.1016/j.jot.2025.08.005	2025	Trauma	Machine Learning (ML)	Prognosis & Prediction
1485	10.1186/s12893-024-02427-x	2024	Trauma; Spine Surgery	Machine Learning (ML)	Prognosis & Prediction
1486	10.1002/jso.27854	2024	Unclassified	Machine Learning (ML)	Prognosis & Prediction
1487	10.1016/j.jhsa.2024.01.003	2024	Arthroplasty	Machine Learning (ML)	Prognosis & Prediction
1488	10.22038/ABJS.2025.81228.3708	2025	Trauma	Machine Learning (ML)	Prognosis & Prediction
1489	10.2106/JBJS.24.01420	2025	Unclassified	Unclassified	Prognosis & Prediction
1490	10.1002/jeo2.70418	2025	Sports Medicine	Unclassified	Diagnosis & Detection
1491	10.1007/s00402-021-04038-0	2021	Arthroplasty	Robotics	Prognosis & Prediction
1492	10.1016/j.knee.2025.02.006	2025	Unclassified	Natural Language Processing (NLP); Machine Learning (ML)	Prognosis & Prediction
1493	10.1007/s12178-020-09630-2	2020	Spine Surgery	Machine Learning (ML)	Prognosis & Prediction
1494	10.1016/j.joca.2021.04.002	2021	Unclassified	Machine Learning (ML)	Prognosis & Prediction
1495	10.3389/fsurg.2024.1505155	2025	Spine Surgery	Machine Learning (ML)	Unclassified
1496	10.1016/j.arth.2023.01.026	2023	Arthroplasty	Machine Learning (ML)	Prognosis & Prediction
1497	10.1097/BRS.0000000000004243	2022	Spine Surgery	Machine Learning (ML)	Prognosis & Prediction
1498	10.1002/jeo2.70247	2025	Unclassified	Unclassified	Prognosis & Prediction
1499	10.1097/BRS.0000000000003749	2021	Trauma; Spine Surgery	Deep Learning (DL)	Diagnosis & Detection
1500	10.3171/2018.8.SPINE1869	2019	Spine Surgery	Machine Learning (ML)	Prognosis & Prediction
1501	10.1055/a-1476-8931	2022	Unclassified	Unclassified	Diagnosis & Detection
1502	10.5312/wjo.v14.i8.598	2023	Unclassified	Unclassified	Unclassified
1503	10.1177/21925682231210183	2024	Trauma; Spine Surgery	Machine Learning (ML)	Unclassified

1504	10.1016/j.jamcollsurg.2020.09.014	2021	Trauma	Machine Learning (ML)	Prognosis & Prediction
1505	10.1007/s00402-021-04048-y	2021	Sports Medicine; Arthroplasty	Machine Learning (ML); Robotics	Prognosis & Prediction
1506	10.1007/s11548-020-02157-4	2020	Unclassified	Deep Learning (DL)	Unclassified
1507	10.14245/ns.2448060.030	2024	Spine Surgery	Unclassified	Diagnosis & Detection
1508	10.1302/2633-1462.67.BJO-2025-0056.R1	2025	Unclassified	Natural Language Processing (NLP); Machine Learning (ML)	Prognosis & Prediction
1509	10.1007/s11548-023-02907-0	2023	Trauma	Deep Learning (DL)	Diagnosis & Detection
1510	10.1530/EOR-23-0081	2023	Trauma; Arthroplasty	Robotics	Unclassified
1511	10.1097/BRS.0000000000005441	2025	Spine Surgery	Machine Learning (ML)	Prognosis & Prediction
1512	10.1016/j.pcorm.2024.100432	2024	Pediatric Orthopaedics	Machine Learning (ML)	Prognosis & Prediction
1513	10.1007/s11548-024-03065-7	2024	Unclassified	Deep Learning (DL)	Segmentation & Measurement
1514	10.1177/15589447251389661	2025	Unclassified	Natural Language Processing (NLP)	Prognosis & Prediction
1515	10.52312/jdrs.2026.2374	2025	Trauma	Machine Learning (ML)	Prognosis & Prediction
1516	10.1002/jor.24811	2021	Sports Medicine; Arthroplasty	Deep Learning (DL)	Prognosis & Prediction
1517	10.22603/ssrr.2023-0255	2024	Trauma; Spine Surgery	Machine Learning (ML)	Prognosis & Prediction
1518	10.1016/j.spinee.2025.04.004	2025	Spine Surgery	Natural Language Processing (NLP); Machine Learning (ML)	Prognosis & Prediction
1519	10.1002/jeo2.70283	2025	Arthroplasty	Robotics	Prognosis & Prediction
1520	10.1016/j.arthro.2023.06.018	2024	Unclassified	Unclassified	Diagnosis & Detection
1521	10.1016/j.spinee.2024.02.010	2024	Spine Surgery	Natural Language Processing (NLP); Machine Learning (ML)	Prognosis & Prediction
1522	10.1016/j.amjsurg.2020.01.043	2020	Unclassified	Machine Learning (ML)	Prognosis & Prediction
1523	10.1002/ksa.12691	2025	Trauma; Arthroplasty	Unclassified	Prognosis & Prediction
1524	10.1007/s00264-023-05909-4	2023	Unclassified	Natural Language Processing (NLP)	Unclassified
1525	10.1186/s13018-024-04654-7	2024	Sports Medicine; Spine Surgery	Deep Learning (DL)	Diagnosis & Detection

1526	10.1007/s11657-025-01602-8	2025	Unclassified	Machine Learning (ML)	Prognosis & Prediction
1527	10.1007/s12283-024-00460-w	2024	Sports Medicine; Arthroplasty	Natural Language Processing (NLP)	Segmentation & Measurement
1528	10.21037/aoj-25-7	2025	Unclassified	Unclassified	Unclassified
1529	10.1007/s11548-024-03299-5	2025	Unclassified	Robotics; Deep Learning (DL)	Segmentation & Measurement
1530	10.1016/j.arthro.2024.08.036	2025	Sports Medicine	Natural Language Processing (NLP)	Unclassified
1531	10.52628/90.3.12636	2024	Unclassified	Machine Learning (ML)	Prognosis & Prediction
1532	10.1055/a-2525-4622	2025	Arthroplasty	Machine Learning (ML)	Prognosis & Prediction
1533	10.1302/2046-3758.132	2024	Sports Medicine	Unclassified	Unclassified
1534	10.1007/s00586-019-06079-x	2019	Unclassified	Machine Learning (ML)	Prognosis & Prediction
1535	10.1177/21925682251375072	2025	Spine Surgery	Machine Learning (ML)	Prognosis & Prediction
1536	10.1010/j.wneu.2025.124018	2025	Trauma	Machine Learning (ML)	Prognosis & Prediction
1537	10.1002/jor.25962	2025	Trauma	Natural Language Processing (NLP); Machine Learning (ML)	Prognosis & Prediction
1538	10.1001/jamaoto.2024.1201	2024	Arthroplasty	Unclassified	Prognosis & Prediction
1539	10.1016/j.joca.2020.12.019	2021	Sports Medicine	Deep Learning (DL)	Segmentation & Measurement
1540	10.1186/s13017-025-00583-w	2025	Trauma; Spine Surgery	Machine Learning (ML)	Prognosis & Prediction
1541	10.1016/j.xnsj.2025.100750	2025	Trauma; Spine Surgery	Machine Learning (ML)	Prognosis & Prediction
1542	10.1007/s00586-024-08223-8	2024	Unclassified	Machine Learning (ML)	Prognosis & Prediction
1543	10.1016/j.hansur.2024.102073	2025	Unclassified	Natural Language Processing (NLP)	Unclassified
1544	10.5137/1019-5149.JTN.36068-21.3	2023	Spine Surgery	Machine Learning (ML)	Prognosis & Prediction
1545	10.1007/s43465-021-00511-5	2021	Pediatric Orthopaedics	Unclassified	Prognosis & Prediction
1546	10.1123/jab.2024-0308	2025	Sports Medicine	Natural Language Processing (NLP)	Prognosis & Prediction
1547	10.1007/s11548-024-03192-1	2025	Arthroplasty	Machine Learning (ML); Robotics	Prognosis & Prediction

1548	10.1016/j.ocarto.2025.100582	2025	Sports Medicine; Arthroplasty	Machine Learning (ML)	Prognosis & Prediction
1549	10.1007/s40279-024-02030-3	2024	Arthroplasty	Unclassified	Prognosis & Prediction
1550	10.1186/s13018-024-05279-6	2024	Arthroplasty	Unclassified	Prognosis & Prediction
1551	10.1016/j.arthro.2024.05.009	2025	Unclassified	Natural Language Processing (NLP)	Unclassified
1552	10.1111/os.14064	2024	Unclassified	Machine Learning (ML)	Unclassified
1553	10.3389/fsurg.2025.1594849	2025	Arthroplasty	Machine Learning (ML); Deep Learning (DL)	Unclassified
1554	10.1016/j.jss.2025.09.007	2025	Arthroplasty	Natural Language Processing (NLP); Machine Learning (ML)	Prognosis & Prediction
1555	10.1002/ksa.12657	2025	Unclassified	Unclassified	Prognosis & Prediction
1556	10.1097/JS9.0000000000002187	2025	Sports Medicine; Spine Surgery; Trauma; Arthroplasty	Deep Learning (DL)	Prognosis & Prediction
1557	10.3171/2023.3.FOCUS22653	2023	Spine Surgery	Machine Learning (ML)	Prognosis & Prediction
1558	10.1016/j.ocarto.2025.100691	2025	Unclassified	Machine Learning (ML)	Prognosis & Prediction
1559	10.1227/neu.0000000000003131	2024	Spine Surgery	Natural Language Processing (NLP); Machine Learning (ML)	Prognosis & Prediction
1560	10.1093/bjs/znae163.699	2024	Unclassified	Natural Language Processing (NLP)	Diagnosis & Detection
1561	10.1016/j.asjsur.2024.02.053	2024	Unclassified	Natural Language Processing (NLP)	Unclassified
1562	10.21037/jss-24-1	2024	Pediatric Orthopaedics; Spine Surgery	Unclassified	Prognosis & Prediction
1563	10.1007/s00264-023-05916-5	2023	Unclassified	Natural Language Processing (NLP)	Unclassified
1564	10.1007/s00586-025-09033-2	2025	Spine Surgery	Unclassified	Prognosis & Prediction
1565	10.1016/j.injury.2025.112577	2025	Trauma	Machine Learning (ML)	Prognosis & Prediction
1566	10.55860/cn2vdj44	2025	Sports Medicine	Machine Learning (ML)	Prognosis & Prediction
1567	10.1007/s00586-025-09653-8	2025	Spine Surgery	Machine Learning (ML); Deep Learning (DL)	Surgical Planning
1568	10.3171/2024.2.FOCUS23927	2023	Spine Surgery	Machine Learning (ML)	Prognosis & Prediction

1569	10.2106/JBJS.OA.25.00174	2025	Spine Surgery	Natural Language Processing (NLP)	Unclassified
1570	10.1007/s00590-023-03796-4	2024	Arthroplasty	Machine Learning (ML)	Prognosis & Prediction
1571	10.1007/s00132-018-3642-4	2018	Trauma; Arthroplasty	Robotics	Diagnosis & Detection
1572	10.1016/j.gaitpost.2024.04.029	2024	Arthroplasty	Machine Learning (ML)	Prognosis & Prediction
1573	10.1302/2046-3758.1310.BJR-2023-0366.R1	2024	Arthroplasty	Machine Learning (ML)	Unclassified
1574	10.1007/s00256-023-04321-8	2023	Unclassified	Deep Learning (DL)	Diagnosis & Detection
1575	10.1016/j.jseint.2024.04.015	2024	Trauma; Arthroplasty	Machine Learning (ML)	Prognosis & Prediction
1576	10.1097/CORR.0000000000002619	2023	Unclassified	Natural Language Processing (NLP)	Unclassified
1577	10.1177/21925682211004250	2022	Spine Surgery	Unclassified	Prognosis & Prediction
1578	10.1097/BSD.0000000000001512	2023	Spine Surgery	Machine Learning (ML)	Prognosis & Prediction
1579	10.1097/TA.0000000000004390	2025	Trauma; Spine Surgery	Machine Learning (ML)	Prognosis & Prediction
1580	10.1080/14763141.2024.2329066	2025	Arthroplasty	Machine Learning (ML); Robotics	Unclassified
1581	10.1007/s00264-022-05507-w	2023	Arthroplasty	Robotics	Prognosis & Prediction
1582	10.1097/PRS.0000000000011041	2024	Spine Surgery	Unclassified	Prognosis & Prediction
1583	10.3171/2020.6.SPINE20861	2021	Unclassified	Unclassified	Prognosis & Prediction
1584	10.1186/s12891-019-2768-9	2019	Unclassified	Machine Learning (ML)	Prognosis & Prediction
1585	10.3171/2023.1.SPINE221095	2023	Spine Surgery	Unclassified	Prognosis & Prediction
1586	10.1016/j.amjsurg.2022.11.025	2023	Arthroplasty	Machine Learning (ML)	Prognosis & Prediction
1587	10.1007/s00167-023-07372-5	2023	Unclassified	Natural Language Processing (NLP)	Unclassified
1588	10.2106/JBJS.23.00269	2023	Unclassified	Natural Language Processing (NLP)	Unclassified
1589	10.1002/jeo2.70361	2025	Sports Medicine; Arthroplasty	Deep Learning (DL)	Surgical Planning

1590	10.1016/j.spinee.2021.06.006	2022	Spine Surgery	Natural Language Processing (NLP); Machine Learning (ML)	Prognosis & Prediction
1591	10.1016/j.xnsj.2025.100816	2025	Trauma	Natural Language Processing (NLP); Machine Learning (ML)	Prognosis & Prediction
1592	10.1097/SCS.0000000000008745	2022	Unclassified	Machine Learning (ML)	Prognosis & Prediction
1593	10.1016/j.surge.2023.08.005	2023	Unclassified	Natural Language Processing (NLP)	Unclassified
1594	10.1007/s00104-019-01091-9	2020	Trauma	Natural Language Processing (NLP); Robotics	Unclassified
1595	10.1007/s11548-024-03166-3	2024	Arthroplasty	Deep Learning (DL)	Unclassified
1596	10.1016/j.knee.2025.06.005	2025	Unclassified	Natural Language Processing (NLP)	Unclassified
1597	10.1007/s00402-023-05151-y	2024	Arthroplasty	Unclassified	Segmentation & Measurement
1598	10.1007/s00586-025-09032-3	2025	Spine Surgery	Machine Learning (ML)	Prognosis & Prediction
1599	10.1123/jsr.2024-0182	2025	Sports Medicine; Arthroplasty	Machine Learning (ML)	Prognosis & Prediction
1600	10.1097/CORR.0000000000002246	2022	Trauma	Unclassified	Prognosis & Prediction
1601	10.1016/j.wneu.2022.07.082	2022	Spine Surgery	Machine Learning (ML)	Prognosis & Prediction
1602	10.1016/j.wneu.2018.12.091	2019	Spine Surgery	Machine Learning (ML)	Prognosis & Prediction
1603	10.1080/14763141.2025.2465793	2025	Arthroplasty	Machine Learning (ML)	Segmentation & Measurement
1604	10.1002/jsp2.1248	2023	Unclassified	Natural Language Processing (NLP); Machine Learning (ML)	Prognosis & Prediction
1605	10.1186/s13018-024-04576-4	2024	Spine Surgery	Machine Learning (ML)	Prognosis & Prediction
1606	10.3389/fsurg.2022.955761	2023	Spine Surgery	Machine Learning (ML)	Prognosis & Prediction
1607	10.1016/j.jse.2018.01.013	2018	Arthroplasty	Machine Learning (ML)	Unclassified
1608	10.1186/s12891-025-08921-4	2025	Trauma	Deep Learning (DL)	Unclassified
1609	10.1089/fpsam.2021.0107	2022	Trauma	Machine Learning (ML)	Prognosis & Prediction
1610	10.1186/s13102-022-00426-0	2022	Sports Medicine; Trauma	Machine Learning (ML)	Prognosis & Prediction
1611	10.1002/jor.26024	2025	Sports Medicine; Trauma; Arthroplasty	Machine Learning (ML); Deep Learning (DL)	Diagnosis & Detection

1612	N/A	2018	Spine Surgery	Machine Learning (ML); Robotics	Segmentation & Measurement
1613	10.1503/cjs.009623	2024	Unclassified	Unclassified	Unclassified
1614	10.1016/j.spinee.2019.04.022	2019	Unclassified	Natural Language Processing (NLP); Machine Learning (ML)	Prognosis & Prediction
1615	10.3389/fsurg.2023.1166734	2023	Unclassified	Machine Learning (ML)	Prognosis & Prediction
1616	10.1016/j.otsr.2024.103859	2024	Trauma	Unclassified	Unclassified
1617	10.1016/j.otsr.2024.103864	2024	Trauma	Unclassified	Unclassified
1618	10.1007/s00264-022-05651-3	2023	Arthroplasty	Machine Learning (ML); Robotics	Prognosis & Prediction
1619	10.1007/s00586-025-08785-1	2025	Trauma; Spine Surgery	Unclassified	Prognosis & Prediction
1620	N/A	2025	Unclassified	Unclassified	Diagnosis & Detection
1621	10.3171/2023.4.SPINE23135	2023	Spine Surgery	Unclassified	Prognosis & Prediction
1622	10.1002/ksa.12653	2025	Unclassified	Natural Language Processing (NLP)	Unclassified
1623	10.1055/a-1231-5304	2021	Sports Medicine; Trauma; Arthroplasty	Machine Learning (ML)	Prognosis & Prediction
1624	10.1007/s11548-025-03426-w	2025	Unclassified	Deep Learning (DL)	Segmentation & Measurement
1625	10.1007/s00586-025-09550-0	2025	Unclassified	Natural Language Processing (NLP)	Prognosis & Prediction
1626	10.1016/j.spinee.2019.06.018	2019	Spine Surgery	Machine Learning (ML)	Prognosis & Prediction
1627	10.1080/17453674.2017.1387732	2017	Unclassified	Machine Learning (ML)	Unclassified
1628	10.1007/s00586-025-08958-y	2025	Sports Medicine; Spine Surgery; Arthroplasty	Deep Learning (DL)	Diagnosis & Detection
1629	10.1016/j.jsurg.2025.103748	2026	Spine Surgery; Trauma; Arthroplasty	Natural Language Processing (NLP); Deep Learning (DL)	Surgical Planning
1630	10.2147/ORR.S519541	2025	Pediatric Orthopaedics; Spine Surgery	Unclassified	Unclassified
1631	10.1177/10556656251387396	2025	Pediatric Orthopaedics	Natural Language Processing (NLP)	Unclassified
1632	10.1016/j.knee.2025.06.003	2025	Unclassified	Natural Language Processing (NLP)	Unclassified
1633	10.1097/BRS.0000000000004572	2023	Spine Surgery	Deep Learning (DL)	Prognosis & Prediction

1634	10.1016/j.injury.2022.12.016	2023	Trauma	Machine Learning (ML)	Prognosis & Prediction
1635	10.21182/jmisst.2024.01172	2024	Trauma; Spine Surgery	Robotics	Prognosis & Prediction
1636	10.1016/j.injury.2024.111334	2024	Trauma	Unclassified	Prognosis & Prediction
1637	10.1016/j.surg.2016.09.044	2017	Unclassified	Machine Learning (ML)	Prognosis & Prediction
1638	10.1016/j.spinee.2025.07.014	2026	Spine Surgery	Natural Language Processing (NLP); Robotics	Prognosis & Prediction
1639	10.1097/BRS.0000000000005303	2025	Spine Surgery	Unclassified	Prognosis & Prediction
1640	10.3171/2023.3.FOCUS22651	2023	Spine Surgery	Machine Learning (ML)	Prognosis & Prediction
1641	10.1001/jamaoto.2025.3225	2025	Arthroplasty	Deep Learning (DL)	Prognosis & Prediction
1642	10.1186/s12891-023-06433-7	2023	Sports Medicine	Unclassified	Prognosis & Prediction
1643	10.1002/jor.25518	2023	Arthroplasty	Machine Learning (ML)	Segmentation & Measurement
1644	10.1007/s00132-024-04496-5	2024	Trauma	Unclassified	Unclassified
1645	10.1007/s00264-023-05887-7	2023	Unclassified	Natural Language Processing (NLP)	Unclassified
1646	10.1177/10806032241283380	2025	Spine Surgery	Unclassified	Prognosis & Prediction
1647	10.1097/PRS.0000000000011099	2024	Trauma	Machine Learning (ML)	Prognosis & Prediction
1648	10.1016/j.joca.2020.02.831	2020	Unclassified	Machine Learning (ML)	Prognosis & Prediction
1649	10.1016/j.ijsu.2022.106889	2022	Arthroplasty	Unclassified	Prognosis & Prediction
1650	10.1186/s13018-025-05799-9	2025	Sports Medicine	Machine Learning (ML)	Prognosis & Prediction
1651	10.3389/fsurg.2021.745220	2021	Arthroplasty	Machine Learning (ML)	Prognosis & Prediction
1652	10.1016/j.cjtee.2024.04.002	2025	Trauma	Unclassified	Prognosis & Prediction
1653	10.1177/10225536231165362	2023	Unclassified	Machine Learning (ML)	Unclassified
1654	10.1016/j.arth.2025.01.050	2025	Arthroplasty	Natural Language Processing (NLP)	Prognosis & Prediction
1655	10.1002/jeo2.70081	2024	Trauma	Natural Language Processing (NLP); Machine Learning (ML)	Prognosis & Prediction
1656	10.1111/iwj.70515	2025	Unclassified	Machine Learning (ML)	Prognosis & Prediction

1657	10.1097/SCS.0000000000011328	2025	Trauma	Machine Learning (ML)	Prognosis & Prediction
1658	10.5435/JAAOS-D-24-00595	2025	Trauma	Natural Language Processing (NLP)	Diagnosis & Detection
1659	10.1177/23259671251389129	2025	Trauma	Machine Learning (ML)	Prognosis & Prediction
1660	10.1186/s12891-024-07555-2	2024	Trauma	Machine Learning (ML)	Prognosis & Prediction
1661	10.1002/jor.24849	2021	Sports Medicine	Deep Learning (DL)	Surgical Planning
1662	10.1001/jamaoto.2022.0900	2022	Pediatric Orthopaedics	Deep Learning (DL)	Prognosis & Prediction
1663	10.1186/s12891-025-09164-z	2025	Arthroplasty	Machine Learning (ML)	Prognosis & Prediction
1664	10.1016/j.arth.2024.03.043	2024	Unclassified	Machine Learning (ML)	Unclassified
1665	10.1097/GOX.0000000000006699	2025	Trauma	Unclassified	Unclassified
1666	10.1016/j.wneu.2024.08.008	2024	Sports Medicine; Spine Surgery	Robotics	Unclassified
1667	10.1007/s00276-025-03769-8	2025	Unclassified	Unclassified	Unclassified
1668	10.1097/CORR.0000000000003417	2025	Unclassified	Natural Language Processing (NLP)	Unclassified
1669	10.1097/CORR.0000000000002705	2023	Arthroplasty	Machine Learning (ML)	Prognosis & Prediction
1670	10.1007/s10103-025-04572-y	2025	Unclassified	Natural Language Processing (NLP); Machine Learning (ML)	Prognosis & Prediction
1671	10.1002/tsm2.181	2020	Trauma	Machine Learning (ML)	Prognosis & Prediction
1672	10.1177/03635465241265692	2024	Unclassified	Natural Language Processing (NLP)	Unclassified
1673	10.1177/23259671241265705	2024	Unclassified	Natural Language Processing (NLP)	Unclassified
1674	10.14245/ns.2448236.118	2024	Spine Surgery	Natural Language Processing (NLP)	Unclassified
1675	10.2106/JBJS.23.00473	2023	Arthroplasty	Natural Language Processing (NLP); Machine Learning (ML)	Unclassified
1676	10.1002/jeo2.70273	2025	Unclassified	Natural Language Processing (NLP)	Unclassified
1677	10.1016/j.knee.2025.03.008	2025	Arthroplasty	Natural Language Processing (NLP)	Prognosis & Prediction
1678	10.1016/j.jos.2025.03.005	2025	Unclassified	Natural Language Processing (NLP)	Unclassified
1679	10.1097/CORR.0000000000003304	2025	Unclassified	Natural Language Processing (NLP)	Unclassified

1680	10.1097/CORR.0000000000003269	2025	Unclassified	Natural Language Processing (NLP)	Unclassified
1681	10.1097/CORR.0000000000003331	2025	Unclassified	Natural Language Processing (NLP)	Unclassified
1682	10.1227/neu.0000000000003360_427	2025	Spine Surgery	Natural Language Processing (NLP)	Unclassified
1683	10.1186/s40634-023-00700-1	2023	Spine Surgery	Natural Language Processing (NLP); Machine Learning (ML)	Prognosis & Prediction
1684	10.1186/s40634-023-00662-4	2023	Sports Medicine	Natural Language Processing (NLP)	Unclassified
1685	10.1177/21925682241230084	2024	Spine Surgery	Natural Language Processing (NLP)	Unclassified
1686	10.1016/j.arthro.2025.06.020	2025	Unclassified	Natural Language Processing (NLP)	Unclassified
1687	10.2519/jospt.2023.12000	2023	Unclassified	Natural Language Processing (NLP)	Unclassified
1688	10.4174/astr.2025.109.5.318	2025	Arthroplasty	Natural Language Processing (NLP)	Unclassified
1689	10.1016/j.wneu.2024.11.114	2025	Trauma; Spine Surgery	Natural Language Processing (NLP)	Unclassified
1690	10.1007/s00586-023-08038-z	2024	Spine Surgery	Natural Language Processing (NLP)	Unclassified
1691	10.1177/15563316251335334	2025	Unclassified	Natural Language Processing (NLP)	Surgical Planning
1692	10.1016/j.arthro.2024.10.010	2025	Unclassified	Natural Language Processing (NLP)	Unclassified
1693	10.1007/s00586-025-09443-2	2025	Spine Surgery	Machine Learning (ML); Deep Learning (DL)	Surgical Planning
1694	10.1016/j.arthro.2024.09.047	2025	Unclassified	Natural Language Processing (NLP)	Prognosis & Prediction
1695	10.1007/s00586-025-08994-8	2025	Spine Surgery	Natural Language Processing (NLP)	Unclassified
1696	10.14245/ns.2448098.049	2024	Spine Surgery	Natural Language Processing (NLP)	Unclassified
1697	10.1016/j.knee.2025.02.007	2025	Trauma; Arthroplasty	Natural Language Processing (NLP)	Prognosis & Prediction
1698	10.1177/15589447241267766	2025	Unclassified	Natural Language Processing (NLP)	Unclassified
1699	10.1007/s00276-025-03723-8	2025	Unclassified	Natural Language Processing (NLP)	Unclassified
1700	10.52312/jdrs.2026.2436	2025	Unclassified	Natural Language Processing (NLP)	Unclassified

1701	10.1177/15589447241257643	2025	Unclassified	Natural Language Processing (NLP); Machine Learning (ML)	Diagnosis & Detection
1702	10.1016/j.jshs.2024.101016	2025	Unclassified	Natural Language Processing (NLP)	Prognosis & Prediction
1703	10.1016/j.jor.2024.12.039	2025	Unclassified	Natural Language Processing (NLP); Machine Learning (ML)	Prognosis & Prediction
1704	10.1016/j.wneu.2025.124423	2025	Spine Surgery	Natural Language Processing (NLP)	Unclassified
1705	10.1016/j.jposna.2025.100294	2026	Sports Medicine; Pediatric Orthopaedics	Natural Language Processing (NLP)	Prognosis & Prediction
1706	10.1016/j.injury.2025.112400	2025	Trauma	Natural Language Processing (NLP); Machine Learning (ML)	Unclassified
1707	10.1002/ksa.70222	2025	Unclassified	Natural Language Processing (NLP); Machine Learning (ML)	Prognosis & Prediction
1708	10.1177/17585732241283971	2025	Unclassified	Natural Language Processing (NLP); Machine Learning (ML)	Unclassified
1709	10.1007/s00586-025-08825-w	2025	Spine Surgery	Natural Language Processing (NLP)	Surgical Planning
1710	10.1227/neu.0000000000003354	2025	Spine Surgery	Natural Language Processing (NLP); Machine Learning (ML); Robotics	Unclassified
1711	10.1016/j.spinee.2025.03.011	2025	Spine Surgery	Natural Language Processing (NLP); Deep Learning (DL)	Prognosis & Prediction
1712	10.1177/17585732251365178	2025	Unclassified	Natural Language Processing (NLP)	Unclassified
1713	10.1016/j.jham.2025.100326	2025	Unclassified	Natural Language Processing (NLP); Deep Learning (DL)	Unclassified
1714	10.1186/s13018-024-04996-2	2024	Unclassified	Natural Language Processing (NLP)	Prognosis & Prediction
1715	10.1177/15589447251371089	2025	Trauma; Arthroplasty	Natural Language Processing (NLP)	Unclassified
1716	10.1186/s12891-025-09227-1	2025	Sports Medicine	Natural Language Processing (NLP)	Unclassified
1717	10.1016/j.jposna.2025.100196	2025	Pediatric Orthopaedics; Spine Surgery	Natural Language Processing (NLP)	Unclassified
1718	10.1016/j.jseint.2024.11.012	2025	Unclassified	Natural Language Processing (NLP)	Unclassified
1719	10.1007/s00256-024-04638-y	2024	Arthroplasty	Natural Language Processing (NLP)	Unclassified
1720	10.1177/23259671251332596	2025	Sports Medicine	Natural Language Processing (NLP)	Prognosis & Prediction

1721	10.1016/j.wneu.2024.05.052	2024	Spine Surgery	Natural Language Processing (NLP)	Diagnosis & Detection
1722	10.14245/ns.2448248.124	2024	Spine Surgery	Natural Language Processing (NLP)	Unclassified
1723	10.1016/j.fas.2024.01.008	2024	Unclassified	Natural Language Processing (NLP)	Unclassified
1724	10.1016/j.ocarto.2025.100687	2025	Unclassified	Natural Language Processing (NLP); Deep Learning (DL)	Prognosis & Prediction
1725	10.1177/15589447241279460	2026	Unclassified	Natural Language Processing (NLP)	Unclassified
1726	10.1177/23259671251385127	2025	Unclassified	Natural Language Processing (NLP)	Prognosis & Prediction
1727	10.1007/s11548-024-03120-3	2024	Trauma	Natural Language Processing (NLP); Machine Learning (ML); Robotics	Prognosis & Prediction
1728	10.1016/j.jss.2025.06.026	2025	Spine Surgery; Arthroplasty	Natural Language Processing (NLP)	Unclassified
1729	10.1016/j.hansur.2025.102530	2025	Unclassified	Natural Language Processing (NLP)	Diagnosis & Detection
1730	10.1007/s12178-025-10001-y	2025	Unclassified	Natural Language Processing (NLP)	Prognosis & Prediction
1731	10.1177/22104917231208212	2024	Unclassified	Natural Language Processing (NLP); Machine Learning (ML)	Prognosis & Prediction
1732	10.1007/s43390-024-00955-3	2025	Pediatric Orthopaedics; Spine Surgery	Natural Language Processing (NLP)	Diagnosis & Detection
1733	10.1002/ksa.70207	2025	Sports Medicine	Natural Language Processing (NLP)	Prognosis & Prediction
1734	10.22038/ABJS.2024.80093.3669	2025	Trauma	Natural Language Processing (NLP)	Prognosis & Prediction
1735	10.1007/s10143-025-03785-7	2025	Spine Surgery	Natural Language Processing (NLP)	Diagnosis & Detection
1736	10.1007/s00256-024-04599-2	2024	Spine Surgery	Natural Language Processing (NLP)	Unclassified
1737	10.1016/j.arthro.2024.02.039	2024	Sports Medicine	Natural Language Processing (NLP)	Unclassified
1738	10.1016/j.arthro.2024.10.020	2025	Trauma	Natural Language Processing (NLP)	Prognosis & Prediction
1739	10.1016/j.wneu.2025.124519	2025	Spine Surgery	Natural Language Processing (NLP)	Prognosis & Prediction
1740	10.1002/ksa.70126	2025	Unclassified	Natural Language Processing (NLP)	Unclassified
1741	10.1002/pmrj.13386	2025	Trauma; Pediatric Orthopaedics	Natural Language Processing (NLP); Machine Learning (ML)	Prognosis & Prediction

1742	10.1177/23259671251371234	2025	Sports Medicine; Trauma	Natural Language Processing (NLP)	Unclassified
1743	10.1016/j.arthro.2024.12.001	2025	Unclassified	Natural Language Processing (NLP); Machine Learning (ML); Deep Learning (DL)	Prognosis & Prediction
1744	10.1016/j.spinee.2024.10.021	2025	Pediatric Orthopaedics; Spine Surgery	Natural Language Processing (NLP)	Prognosis & Prediction
1745	10.1007/s00132-025-04754-0	2025	Unclassified	Natural Language Processing (NLP)	Unclassified
1746	10.1186/s40634-023-00683-z	2023	Trauma	Natural Language Processing (NLP); Machine Learning (ML)	Prognosis & Prediction
1747	10.1227/neu.0000000000002632	2023	Spine Surgery	Natural Language Processing (NLP); Machine Learning (ML)	Unclassified
1748	10.2106/JBJS.23.00914	2024	Spine Surgery	Natural Language Processing (NLP)	Unclassified
1749	10.2106/JBJS.RVW.24.00122	2024	Arthroplasty	Natural Language Processing (NLP)	Prognosis & Prediction
1750	10.1007/s00132-025-04752-2	2025	Trauma; Arthroplasty	Natural Language Processing (NLP); Machine Learning (ML)	Diagnosis & Detection
1751	10.1016/j.arth.2024.03.017	2024	Arthroplasty	Natural Language Processing (NLP); Machine Learning (ML)	Prognosis & Prediction
1752	10.1016/j.arthro.2024.10.042	2025	Sports Medicine; Trauma	Natural Language Processing (NLP)	Unclassified
1753	10.1002/jsp2.1300	2023	Spine Surgery	Natural Language Processing (NLP)	Unclassified
1754	10.1007/s00276-024-03549-w	2024	Unclassified	Natural Language Processing (NLP)	Unclassified
1755	10.5435/JAAOSGlobal-D-25-00174	2025	Trauma	Natural Language Processing (NLP); Machine Learning (ML); Deep Learning (DL)	Prognosis & Prediction
1756	10.1007/s00142-024-00668-9	2024	Sports Medicine; Trauma	Natural Language Processing (NLP)	Prognosis & Prediction
1757	10.1016/j.arthro.2025.03.066	2025	Sports Medicine	Natural Language Processing (NLP)	Unclassified
1758	10.2106/JBJS.OA.24.00007	2025	Unclassified	Natural Language Processing (NLP); Machine Learning (ML)	Prognosis & Prediction
1759	10.1002/rcs.2621	2024	Arthroplasty	Natural Language Processing (NLP)	Unclassified
1760	10.1007/s00586-024-08198-6	2024	Spine Surgery	Natural Language Processing (NLP)	Prognosis & Prediction

1761	10.1177/10556656251378591	2025	Pediatric Orthopaedics	Natural Language Processing (NLP)	Unclassified
1762	10.1097/IOP.0000000000002868	2025	Trauma	Natural Language Processing (NLP)	Prognosis & Prediction
1763	10.1186/s13018-025-05981-z	2025	Pediatric Orthopaedics	Natural Language Processing (NLP)	Diagnosis & Detection
1764	10.1007/s00256-025-05070-6	2025	Unclassified	Natural Language Processing (NLP)	Diagnosis & Detection
1765	10.2106/JBJS.24.01429	2025	Pediatric Orthopaedics; Spine Surgery	Natural Language Processing (NLP)	Unclassified
1766	10.1016/j.jisako.2025.100892	2025	Arthroplasty	Natural Language Processing (NLP)	Unclassified
1767	10.1177/21925682231195783	2024	Spine Surgery	Natural Language Processing (NLP)	Diagnosis & Detection
1768	10.5435/JAAOS-D-24-01049	2025	Trauma	Natural Language Processing (NLP)	Diagnosis & Detection
1769	10.1016/j.arthro.2025.04.033	2025	Sports Medicine; Trauma	Natural Language Processing (NLP)	Prognosis & Prediction
1770	10.1016/j.jse.2024.02.029	2024	Unclassified	Natural Language Processing (NLP); Machine Learning (ML); Deep Learning (DL)	Unclassified
1771	10.1016/j.xnsj.2025.100787	2025	Spine Surgery	Natural Language Processing (NLP); Machine Learning (ML); Robotics	Prognosis & Prediction
1772	10.1007/s43465-023-00967-7	2023	Trauma	Natural Language Processing (NLP)	Unclassified
1773	10.1186/s43019-024-00218-5	2024	Arthroplasty	Natural Language Processing (NLP)	Unclassified
1774	10.1186/s12891-024-07934-9	2024	Arthroplasty	Deep Learning (DL)	Diagnosis & Detection
1775	10.1016/j.xnsj.2024.100580	2025	Spine Surgery	Natural Language Processing (NLP); Deep Learning (DL)	Prognosis & Prediction
1776	10.1097/BRS.0000000000004915	2024	Spine Surgery	Natural Language Processing (NLP)	Prognosis & Prediction
1777	10.1007/s43465-025-01430-5	2025	Unclassified	Natural Language Processing (NLP)	Unclassified
1778	10.1007/s10143-025-03783-9	2025	Spine Surgery	Natural Language Processing (NLP); Machine Learning (ML)	Prognosis & Prediction
1779	10.1016/j.sopen.2025.04.012	2025	Trauma; Arthroplasty	Natural Language Processing (NLP)	Unclassified

1780	10.1186/s13018-025-05831-y	2025	Sports Medicine	Natural Language Processing (NLP)	Unclassified
1781	10.1002/ksa.70247	2025	Sports Medicine	Natural Language Processing (NLP)	Unclassified
1782	10.1177/15563316251341321	2025	Trauma	Natural Language Processing (NLP); Machine Learning (ML); Robotics	Prognosis & Prediction
1783	10.1016/j.bjps.2025.04.033	2025	Unclassified	Natural Language Processing (NLP)	Unclassified
1784	10.1142/S2424835524300019	2024	Unclassified	Natural Language Processing (NLP)	Surgical Planning
1785	10.1016/j.knee.2025.04.004	2025	Unclassified	Natural Language Processing (NLP)	Diagnosis & Detection
1786	10.1302/0301-620X.106B7.BJJ-2024-0136$2.00	2024	Arthroplasty	Natural Language Processing (NLP)	Prognosis & Prediction
1787	10.1177/19417381251326549	2025	Unclassified	Natural Language Processing (NLP)	Diagnosis & Detection
1788	10.1016/j.jor.2023.10.026	2024	Sports Medicine	Natural Language Processing (NLP)	Prognosis & Prediction
1789	10.1002/jeo2.70393	2025	Sports Medicine; Arthroplasty	Natural Language Processing (NLP)	Unclassified
1790	10.1097/BPO.0000000000002695	2024	Pediatric Orthopaedics	Natural Language Processing (NLP)	Diagnosis & Detection
1791	10.1007/s43465-025-01474-7	2025	Arthroplasty	Natural Language Processing (NLP)	Unclassified
1792	10.1002/ksa.12571	2025	Sports Medicine; Trauma	Natural Language Processing (NLP)	Unclassified
1793	10.2106/JBJS.24.01462	2025	Sports Medicine; Arthroplasty	Natural Language Processing (NLP)	Prognosis & Prediction
1794	10.1016/j.jse.2023.11.014	2024	Arthroplasty	Natural Language Processing (NLP)	Prognosis & Prediction
1795	10.21037/aoj-24-49	2025	Unclassified	Natural Language Processing (NLP)	Unclassified
1796	10.1186/s10195-023-00740-4	2023	Arthroplasty	Natural Language Processing (NLP); Machine Learning (ML)	Diagnosis & Detection
1797	10.1097/BPO.0000000000002682	2024	Pediatric Orthopaedics	Natural Language Processing (NLP)	Prognosis & Prediction
1798	10.1016/j.hansur.2022.02.003	2022	Arthroplasty	Deep Learning (DL)	Prognosis & Prediction
1799	10.1007/s00256-021-03828-2	2022	Unclassified	Deep Learning (DL)	Unclassified
1800	N/A	2018	Unclassified	Deep Learning (DL)	Prognosis & Prediction
1801	10.1016/j.arthro.2024.06.021	2025	Sports Medicine	Natural Language Processing (NLP)	Prognosis & Prediction

1802	10.1097/CORR.00000000000003263	2025	Unclassified	Natural Language Processing (NLP)	Prognosis & Prediction
1803	10.1016/j.jse.2023.12.009	2024	Unclassified	Deep Learning (DL)	Prognosis & Prediction
1804	10.1016/j.joca.2020.12.017	2021	Unclassified	Machine Learning (ML); Deep Learning (DL)	Prognosis & Prediction
1805	10.52312/jdrs.2022.445	2022	Trauma; Spine Surgery	Deep Learning (DL)	Diagnosis & Detection
1806	10.3928/01477447-20240718-02	2024	Unclassified	Natural Language Processing (NLP); Machine Learning (ML); Deep Learning (DL)	Prognosis & Prediction
1807	10.1117/12.2545001	2019	Spine Surgery	Deep Learning (DL)	Segmentation & Measurement
1808	N/A	2020	Sports Medicine	Deep Learning (DL)	Segmentation & Measurement
1809	10.1016/j.ijom.2024.09.004	2025	Trauma; Arthroplasty	Machine Learning (ML); Deep Learning (DL)	Prognosis & Prediction
1810	10.1007/s00256-019-03284-z	2020	Unclassified	Deep Learning (DL)	Diagnosis & Detection
1811	10.14245/ns.2448898.449	2024	Trauma; Spine Surgery	Deep Learning (DL)	Diagnosis & Detection
1812	10.5435/JAAOS-D-23-00831	2024	Unclassified	Deep Learning (DL)	Unclassified
1813	10.1016/j.surg.2021.08.055	2022	Unclassified	Deep Learning (DL)	Prognosis & Prediction
1814	10.1007/s00256-020-03458-0	2020	Sports Medicine	Deep Learning (DL)	Diagnosis & Detection
1815	10.1007/s00586-021-07073-y	2022	Trauma; Spine Surgery	Machine Learning (ML); Deep Learning (DL)	Prognosis & Prediction
1816	10.1177/2292550321997012	2021	Unclassified	Deep Learning (DL)	Unclassified
1817	10.1002/rcs.2639	2024	Trauma	Machine Learning (ML); Robotics; Deep Learning (DL)	Prognosis & Prediction
1818	10.1002/jor.70000	2025	Trauma; Spine Surgery	Deep Learning (DL)	Prognosis & Prediction
1819	10.1016/j.joca.2018.02.845	2018	Unclassified	Deep Learning (DL)	Unclassified
1820	10.1007/s00586-023-08089-2	2024	Spine Surgery	Machine Learning (ML); Deep Learning (DL)	Diagnosis & Detection
1821	10.1007/s11548-023-02839-9	2023	Trauma	Deep Learning (DL)	Diagnosis & Detection
1822	10.22038/ABJS.2024.76701.3545	2024	Pediatric Orthopaedics; Spine Surgery; Arthroplasty	Machine Learning (ML); Deep Learning (DL)	Prognosis & Prediction
1823	10.1245/s10434-018-6343-7	2018	Unclassified	Deep Learning (DL)	Prognosis & Prediction

1824	10.1002/rcs.2229	2021	Unclassified	Deep Learning (DL)	Segmentation & Measurement
1825	10.5435/JAAOS-D-20-01142	2022	Trauma	Machine Learning (ML); Deep Learning (DL)	Prognosis & Prediction
1826	10.1007/s11548-018-1721-y	2018	Unclassified	Deep Learning (DL)	Surgical Planning
1827	10.1016/j.joca.2024.02.891	2024	Sports Medicine; Arthroplasty	Machine Learning (ML); Deep Learning (DL)	Prognosis & Prediction
1828	10.1007/s43390-024-00940-w	2024	Pediatric Orthopaedics; Spine Surgery	Natural Language Processing (NLP); Machine Learning (ML); Deep Learning (DL)	Prognosis & Prediction
1829	10.1155/aort/6257188	2025	Trauma	Natural Language Processing (NLP); Machine Learning (ML); Deep Learning (DL)	Prognosis & Prediction
1830	10.1186/s12893-024-02646-2	2024	Spine Surgery	Deep Learning (DL)	Prognosis & Prediction
1831	10.3389/fsurg.2023.1351643	2023	Spine Surgery	Machine Learning (ML); Deep Learning (DL)	Unclassified
1832	10.1302/0301-620X.103B9.BJJ-2021-0192.R1	2021	Spine Surgery	Machine Learning (ML); Deep Learning (DL)	Prognosis & Prediction
1833	10.1007/s00256-021-03873-x	2022	Spine Surgery	Deep Learning (DL)	Prognosis & Prediction
1834	10.1097/CORR.0000000000002828	2023	Trauma	Deep Learning (DL)	Prognosis & Prediction
1835	10.1016/j.clinbiomech.2025.106646	2025	Arthroplasty	Deep Learning (DL)	Prognosis & Prediction
1836	10.1227/ons.0000000000000173	2022	Spine Surgery	Deep Learning (DL)	Unclassified
1837	10.1007/s00586-023-08099-0	2024	Spine Surgery	Deep Learning (DL)	Diagnosis & Detection
1838	N/A	2020	Unclassified	Deep Learning (DL)	Unclassified
1839	10.1590/1517-8692202127032021_0127	2021	Sports Medicine; Arthroplasty	Deep Learning (DL)	Prognosis & Prediction
1840	N/A	2024	Unclassified	Deep Learning (DL)	Diagnosis & Detection
1841	10.14245/ns.1836248.124	2018	Spine Surgery	Machine Learning (ML)	Prognosis & Prediction
1842	10.1097/BSD.0000000000001119	2021	Spine Surgery	Machine Learning (ML); Deep Learning (DL)	Unclassified

1843	10.1016/j.xnsj.2025.100598	2025	Spine Surgery	Deep Learning (DL)	Prognosis & Prediction
1844	10.1016/j.ocl.2022.11.004	2023	Unclassified	Machine Learning (ML); Deep Learning (DL)	Prognosis & Prediction
1845	10.1177/2192568220969373	2022	Spine Surgery	Machine Learning (ML)	Prognosis & Prediction
1846	10.1177/21925682231211273	2025	Pediatric Orthopaedics	Deep Learning (DL)	Prognosis & Prediction
1847	10.1007/s10143-023-01987-5	2023	Spine Surgery	Deep Learning (DL)	Diagnosis & Detection
1848	10.5312/wjo.v12.i9.685	2021	Trauma; Spine Surgery	Machine Learning (ML); Deep Learning (DL)	Prognosis & Prediction
1849	10.1007/s11657-021-00985-8	2021	Trauma	Deep Learning (DL)	Prognosis & Prediction
1850	10.5435/JAAOS-D-20-00429	2021	Trauma	Machine Learning (ML); Deep Learning (DL)	Prognosis & Prediction
1851	10.1186/s12891-023-06153-y	2023	Sports Medicine; Arthroplasty	Deep Learning (DL)	Surgical Planning
1852	10.1007/s00586-025-08908-8	2025	Spine Surgery	Deep Learning (DL)	Prognosis & Prediction
1853	10.1007/s00701-025-06645-x	2025	Sports Medicine; Pediatric Orthopaedics; Spine Surgery	Deep Learning (DL)	Prognosis & Prediction
1854	10.1007/s11548-022-02601-7	2022	Arthroplasty	Robotics; Deep Learning (DL)	Prognosis & Prediction
1855	10.1097/BRS.0000000000004481	2022	Spine Surgery	Machine Learning (ML); Deep Learning (DL)	Prognosis & Prediction
1856	10.1016/j.jor.2021.11.013	2021	Arthroplasty	Deep Learning (DL)	Prognosis & Prediction
1857	10.1016/j.spinee.2025.01.020	2025	Sports Medicine; Spine Surgery	Natural Language Processing (NLP); Deep Learning (DL)	Prognosis & Prediction
1858	10.1016/j.wneu.2022.07.041	2022	Spine Surgery	Deep Learning (DL)	Prognosis & Prediction
1859	10.1097/BRS.0000000000005438	2025	Trauma; Spine Surgery	Deep Learning (DL)	Diagnosis & Detection
1860	10.1002/ksa.12547	2025	Trauma; Arthroplasty	Deep Learning (DL)	Prognosis & Prediction
1861	10.1007/s00113-022-01202-y	2022	Trauma; Arthroplasty	Machine Learning (ML); Deep Learning (DL)	Prognosis & Prediction
1862	10.1055/a-2674-3914	2025	Trauma	Deep Learning (DL)	Prognosis & Prediction
1863	10.1080/02688697.2018.1436691	2018	Spine Surgery	Deep Learning (DL)	Prognosis & Prediction

1864	10.1016/j.knee.2022.12.007	2023	Sports Medicine	Machine Learning (ML); Deep Learning (DL)	Prognosis & Prediction
1865	10.1007/s00256-023-04324-5	2023	Trauma	Deep Learning (DL)	Unclassified
1866	10.1177/2192568220973984	2021	Spine Surgery	Unclassified	Unclassified
1867	10.1177/2192568219868190	2020	Spine Surgery	Machine Learning (ML); Deep Learning (DL)	Segmentation & Measurement
1868	10.1097/BPB.0000000000001198	2025	Pediatric Orthopaedics; Arthroplasty	Deep Learning (DL)	Prognosis & Prediction
1869	10.1016/j.jelekin.2019.07.007	2022	Trauma; Arthroplasty	Deep Learning (DL)	Segmentation & Measurement
1870	N/A	2019	Unclassified	Robotics; Deep Learning (DL)	Surgical Planning
1871	10.1007/s11332-023-01075-7	2024	Arthroplasty	Deep Learning (DL)	Unclassified
1872	10.31616/asj.2020.0147	2020	Spine Surgery	Natural Language Processing (NLP); Deep Learning (DL)	Prognosis & Prediction
1873	10.1097/BRS.0000000000004439	2022	Spine Surgery	Machine Learning (ML); Deep Learning (DL)	Prognosis & Prediction
1874	10.3390/jfmk9030123	2024	Pediatric Orthopaedics	Deep Learning (DL)	Segmentation & Measurement
1875	10.1186/s12891-025-09011-1	2025	Unclassified	Deep Learning (DL)	Prognosis & Prediction
1876	10.1016/j.gaitpost.2025.01.014	2025	Sports Medicine; Arthroplasty	Deep Learning (DL)	Prognosis & Prediction
1877	10.1002/rcs.1989	2019	Unclassified	Robotics; Deep Learning (DL)	Diagnosis & Detection
1878	10.1016/j.spinee.2022.01.004	2022	Sports Medicine; Trauma; Spine Surgery	Natural Language Processing (NLP); Deep Learning (DL)	Prognosis & Prediction
1879	10.1007/s43465-021-00455-w	2021	Pediatric Orthopaedics	Deep Learning (DL)	Prognosis & Prediction
1880	10.1055/s-0044-1779317	2024	Spine Surgery	Deep Learning (DL)	Diagnosis & Detection
1881	10.1007/s43390-025-01125-9	2025	Pediatric Orthopaedics; Spine Surgery	Deep Learning (DL)	Prognosis & Prediction
1882	10.1002/jor.24617	2020	Arthroplasty	Deep Learning (DL)	Prognosis & Prediction
1883	10.1080/02640414.2023.2300570	2023	Sports Medicine; Trauma; Arthroplasty	Machine Learning (ML); Deep Learning (DL)	Prognosis & Prediction
1884	10.1016/j.gaitpost.2023.03.020	2023	Unclassified	Machine Learning (ML); Deep Learning (DL)	Prognosis & Prediction

1885	10.1007/s11548-021-02432-y	2021	Trauma	Deep Learning (DL)	Prognosis & Prediction
1886	10.55095/ACHOT2023/041	2023	Unclassified	Deep Learning (DL)	Diagnosis & Detection
1887	10.14444/7131	2020	Spine Surgery	Natural Language Processing (NLP); Deep Learning (DL)	Prognosis & Prediction
1888	10.1016/j.wneu.2021.02.114	2021	Spine Surgery	Machine Learning (ML); Deep Learning (DL)	Prognosis & Prediction
1889	10.1002/ksa.12123	2024	Sports Medicine; Trauma	Deep Learning (DL)	Prognosis & Prediction
1890	10.1002/jor.25201	2022	Unclassified	Deep Learning (DL)	Segmentation & Measurement
1891	10.1080/02640414.2023.2251767	2023	Arthroplasty	Machine Learning (ML); Deep Learning (DL)	Prognosis & Prediction
1892	10.1016/j.arth.2018.02.067	2018	Unclassified	Natural Language Processing (NLP); Machine Learning (ML); Deep Learning (DL)	Unclassified
1893	10.1016/j.gaitpost.2019.02.011	2019	Arthroplasty	Deep Learning (DL)	Unclassified
1894	10.1080/17461391.2022.2028013	2023	Arthroplasty	Deep Learning (DL)	Prognosis & Prediction
1895	10.1007/s11548-024-03075-5	2024	Sports Medicine; Arthroplasty	Machine Learning (ML); Deep Learning (DL)	Segmentation & Measurement
1896	10.1308/rcsann.2023.0017	2023	Trauma; Pediatric Orthopaedics	Deep Learning (DL)	Diagnosis & Detection
1897	10.1055/s-0041-1741396	2023	Arthroplasty	Machine Learning (ML); Deep Learning (DL)	Prognosis & Prediction
1898	10.1002/ksa.12481	2025	Sports Medicine; Trauma; Arthroplasty	Deep Learning (DL)	Surgical Planning
1899	10.1080/02640414.2023.2194725	2023	Arthroplasty	Machine Learning (ML); Deep Learning (DL)	Unclassified
1900	10.1007/s43465-024-01272-7	2025	Trauma; Arthroplasty	Deep Learning (DL)	Prognosis & Prediction
1901	10.1007/s00586-021-06914-0	2021	Sports Medicine; Trauma; Spine Surgery	Deep Learning (DL)	Prognosis & Prediction
1902	10.1016/j.injury.2022.04.013	2022	Trauma	Deep Learning (DL)	Prognosis & Prediction
1903	10.1080/14763141.2020.1718196	2022	Sports Medicine; Arthroplasty	Machine Learning (ML); Deep Learning (DL)	Prognosis & Prediction
1904	10.1002/rcs.2275	2021	Arthroplasty	Robotics	Unclassified

1905	10.1016/j.arth.2023.06.009	2023	Arthroplasty	Machine Learning (ML); Deep Learning (DL)	Prognosis & Prediction
1906	10.1186/s12891-022-05818-4	2022	Sports Medicine; Arthroplasty	Deep Learning (DL)	Prognosis & Prediction
1907	10.14444/8503	2023	Spine Surgery	Machine Learning (ML)	Prognosis & Prediction
1908	10.1016/j.knee.2022.11.026	2023	Unclassified	Deep Learning (DL)	Surgical Planning
1909	10.1016/j.afos.2025.09.002	2025	Trauma	Machine Learning (ML); Deep Learning (DL)	Prognosis & Prediction
1910	10.3389/fsurg.2020.00054	2020	Trauma; Spine Surgery	Natural Language Processing (NLP); Machine Learning (ML); Deep Learning (DL)	Prognosis & Prediction
1911	10.1186/s12891-022-05468-6	2022	Sports Medicine	Natural Language Processing (NLP); Machine Learning (ML); Deep Learning (DL)	Diagnosis & Detection
1912	10.1007/s00264-022-05634-4	2023	Sports Medicine; Trauma; Arthroplasty	Machine Learning (ML); Robotics; Deep Learning (DL)	Segmentation & Measurement
1913	10.1007/s00256-023-04283-x	2023	Spine Surgery	Deep Learning (DL)	Prognosis & Prediction
1914	10.1016/j.joca.2020.12.007	2021	Sports Medicine	Machine Learning (ML); Deep Learning (DL)	Unclassified
1915	10.1007/s00586-016-4654-6	2016	Spine Surgery	Deep Learning (DL)	Diagnosis & Detection
1916	10.1007/s11548-020-02162-7	2020	Unclassified	Robotics; Deep Learning (DL)	Diagnosis & Detection
1917	10.1007/s00256-020-03410-2	2020	Sports Medicine	Deep Learning (DL)	Diagnosis & Detection
1918	10.1123/jab.2024-0113	2025	Arthroplasty	Machine Learning (ML); Deep Learning (DL)	Prognosis & Prediction
1919	10.1016/j.jelekin.2017.06.001	2017	Arthroplasty	Deep Learning (DL)	Prognosis & Prediction
1920	10.1097/JSA.0000000000000371	2023	Unclassified	Machine Learning (ML); Robotics; Deep Learning (DL)	Prognosis & Prediction
1921	10.1016/j.gaitpost.2021.06.015	2021	Unclassified	Deep Learning (DL)	Prognosis & Prediction
1922	10.1007/s00586-023-07954-4	2023	Spine Surgery	Machine Learning (ML); Deep Learning (DL)	Prognosis & Prediction
1923	10.1186/s12891-025-08846-y	2025	Sports Medicine; Arthroplasty	Machine Learning (ML); Deep Learning (DL)	Diagnosis & Detection
1924	10.1016/j.arth.2021.02.028	2021	Arthroplasty	Deep Learning (DL)	Prognosis & Prediction

1925	10.1117/12.2610770	2022	Trauma; Arthroplasty	Machine Learning (ML)	Surgical Planning
1926	10.1007/s11548-022-02577-4	2022	Arthroplasty	Deep Learning (DL)	Diagnosis & Detection
1927	10.1007/s00256-022-04008-6	2022	Unclassified	Deep Learning (DL)	Diagnosis & Detection
1928	10.1097/BRS.0000000000004532	2023	Spine Surgery	Deep Learning (DL)	Diagnosis & Detection
1929	10.1302/2633-1462.210.BJO-2021-0133	2021	Trauma	Machine Learning (ML); Deep Learning (DL)	Prognosis & Prediction
1930	10.52312/jdrs.2023.1291	2023	Unclassified	Deep Learning (DL)	Segmentation & Measurement
1931	10.1007/s11548-021-02373-6	2021	Arthroplasty	Deep Learning (DL)	Prognosis & Prediction
1932	10.1016/j.jse.2024.12.033	2025	Sports Medicine	Machine Learning (ML); Deep Learning (DL)	Prognosis & Prediction
1933	10.1007/978-3-030-01201-4_31	2018	Arthroplasty	Deep Learning (DL)	Prognosis & Prediction
1934	10.1097/BSD.0000000000001547	2024	Sports Medicine; Trauma; Spine Surgery	Deep Learning (DL)	Diagnosis & Detection
1935	10.1016/j.spinee.2021.01.022	2021	Pediatric Orthopaedics	Natural Language Processing (NLP); Deep Learning (DL)	Prognosis & Prediction
1936	10.1080/14763141.2025.2481164	2025	Sports Medicine; Trauma; Arthroplasty	Machine Learning (ML); Deep Learning (DL)	Prognosis & Prediction
1937	10.1007/s00586-023-07838-7	2023	Trauma; Spine Surgery	Natural Language Processing (NLP); Deep Learning (DL)	Diagnosis & Detection
1938	10.1002/jsp2.1044	2019	Pediatric Orthopaedics; Trauma; Spine Surgery	Natural Language Processing (NLP); Machine Learning (ML); Robotics; Deep Learning (DL)	Prognosis & Prediction
1939	10.1016/j.otsr.2022.103456	2023	Trauma; Spine Surgery	Machine Learning (ML); Robotics; Deep Learning (DL)	Prognosis & Prediction
1940	10.1007/s00142-023-00606-1	2023	Sports Medicine; Trauma	Deep Learning (DL)	Prognosis & Prediction
1941	10.1016/j.artd.2025.101656	2025	Trauma; Arthroplasty	Machine Learning (ML); Deep Learning (DL)	Segmentation & Measurement
1942	10.1097/BPO.0000000000002611	2024	Pediatric Orthopaedics	Machine Learning (ML); Deep Learning (DL)	Segmentation & Measurement
1943	10.1007/s00586-022-07320-w	2022	Spine Surgery	Deep Learning (DL)	Segmentation & Measurement

1944	10.5312/wjo.v14.i6.387	2023	Unclassified	Deep Learning (DL)	Diagnosis & Detection
1945	10.48307/ATR.2023.176358	2023	Trauma	Deep Learning (DL)	Diagnosis & Detection
1946	10.1016/j.knee.2023.03.010	2023	Arthroplasty	Deep Learning (DL)	Prognosis & Prediction
1947	10.1016/j.spinee.2022.05.002	2022	Spine Surgery	Natural Language Processing (NLP); Robotics; Deep Learning (DL)	Prognosis & Prediction
1948	10.3171/2021.3.SPINE21189	2022	Spine Surgery	Deep Learning (DL)	Prognosis & Prediction
1949	10.1016/j.gaitpost.2025.05.006	2025	Pediatric Orthopaedics; Arthroplasty	Deep Learning (DL)	Diagnosis & Detection
1950	10.1055/a-1511-8595	2023	Trauma; Spine Surgery	Machine Learning (ML); Deep Learning (DL)	Diagnosis & Detection
1951	10.1097/BRS.0000000000004308	2022	Spine Surgery; Arthroplasty	Deep Learning (DL)	Diagnosis & Detection
1952	10.1016/j.humov.2020.102649	2020	Trauma; Arthroplasty	Machine Learning (ML); Deep Learning (DL)	Unclassified
1953	10.3171/2019.3.SPINE181367	2019	Spine Surgery	Machine Learning (ML); Deep Learning (DL)	Prognosis & Prediction
1954	10.1007/s11548-022-02798-7	2023	Unclassified	Natural Language Processing (NLP); Deep Learning (DL)	Diagnosis & Detection
1955	10.1080/17453674.2019.1600125	2019	Trauma	Deep Learning (DL)	Diagnosis & Detection
1956	10.1007/s11548-018-1818-3	2018	Spine Surgery	Natural Language Processing (NLP); Deep Learning (DL)	Segmentation & Measurement
1957	10.1097/SCS.0000000000007435	2021	Trauma	Deep Learning (DL)	Prognosis & Prediction
1958	10.1007/s11548-022-02830-w	2023	Unclassified	Deep Learning (DL)	Surgical Planning
1959	N/A	2016	Spine Surgery	Deep Learning (DL)	Prognosis & Prediction
1960	10.1002/rcs.2672	2024	Arthroplasty	Deep Learning (DL)	Unclassified
1961	10.1007/s11548-017-1575-8	2017	Spine Surgery	Machine Learning (ML); Deep Learning (DL)	Segmentation & Measurement
1962	10.1186/s12891-022-05524-1	2022	Sports Medicine; Trauma	Machine Learning (ML); Deep Learning (DL)	Prognosis & Prediction
1963	10.1186/s12891-025-08939-8	2025	Sports Medicine; Arthroplasty	Deep Learning (DL)	Prognosis & Prediction
1964	10.3389/fsurg.2023.1172313	2023	Spine Surgery	Deep Learning (DL)	Diagnosis & Detection

1965	10.1530/EOR-24-0019	2024	Spine Surgery	Natural Language Processing (NLP); Machine Learning (ML); Deep Learning (DL)	Prognosis & Prediction
1966	10.1007/s00256-021-03862-0	2022	Spine Surgery	Natural Language Processing (NLP)	Segmentation & Measurement
1967	10.1002/jor.70024	2025	Arthroplasty	Machine Learning (ML); Deep Learning (DL)	Diagnosis & Detection
1968	10.1007/s11548-021-02525-8	2022	Unclassified	Robotics; Deep Learning (DL)	Unclassified
1969	10.23736/S0390-5616.16.03078-2	2017	Spine Surgery	Deep Learning (DL)	Unclassified
1970	10.2340/17453674.2024.42702	2025	Trauma; Pediatric Orthopaedics	Deep Learning (DL)	Diagnosis & Detection
1971	10.1002/jsp2.70000	2024	Spine Surgery	Deep Learning (DL)	Segmentation & Measurement
1972	10.1186/s13018-025-05459-y	2025	Sports Medicine	Machine Learning (ML); Deep Learning (DL)	Prognosis & Prediction
1973	10.1186/s42836-022-00145-4	2022	Arthroplasty	Deep Learning (DL)	Diagnosis & Detection
1974	10.1080/02701367.2022.2070103	2023	Arthroplasty	Deep Learning (DL)	Segmentation & Measurement
1975	10.1016/j.fas.2022.05.005	2022	Trauma	Machine Learning (ML); Deep Learning (DL)	Prognosis & Prediction
1976	10.1097/SCS.0000000000008198	2022	Unclassified	Deep Learning (DL)	Segmentation & Measurement
1977	10.1136/tsaco-2021-000705	2021	Trauma	Deep Learning (DL)	Prognosis & Prediction
1978	10.1097/BRS.0000000000004903	2024	Spine Surgery	Deep Learning (DL)	Diagnosis & Detection
1979	10.23736/S0390-5616.17.04123-6	2021	Trauma	Deep Learning (DL)	Prognosis & Prediction
1980	10.1016/j.jposna.2025.100291	2026	Trauma; Pediatric Orthopaedics	Deep Learning (DL)	Prognosis & Prediction
1981	10.3389/fsurg.2022.977505	2022	Arthroplasty	Deep Learning (DL)	Prognosis & Prediction
1982	10.1007/978-3-319-74113-0_9	2018	Trauma; Spine Surgery	Deep Learning (DL)	Diagnosis & Detection
1983	10.1016/j.arth.2021.02.026	2021	Arthroplasty	Deep Learning (DL)	Prognosis & Prediction

1984	10.1016/j.knee.2025.10.028	2026	Sports Medicine; Arthroplasty	Natural Language Processing (NLP); Machine Learning (ML); Deep Learning (DL)	Prognosis & Prediction
1985	10.1016/j.joca.2025.02.787	2025	Sports Medicine; Arthroplasty	Natural Language Processing (NLP); Deep Learning (DL)	Prognosis & Prediction
1986	10.1007/s00256-021-03709-8	2021	Trauma	Deep Learning (DL)	Diagnosis & Detection
1987	10.1177/2192568220961353	2021	Trauma; Spine Surgery	Machine Learning (ML)	Diagnosis & Detection
1988	10.1186/s12891-025-08784-9	2025	Arthroplasty	Deep Learning (DL)	Surgical Planning
1989	10.1007/s00586-021-07025-6	2022	Spine Surgery	Deep Learning (DL)	Diagnosis & Detection
1990	10.1007/s00256-021-03844-2	2022	Sports Medicine; Pediatric Orthopaedics; Spine Surgery	Deep Learning (DL)	Prognosis & Prediction
1991	10.52312/jdrs.2023.1308	2023	Pediatric Orthopaedics	Deep Learning (DL)	Diagnosis & Detection
1992	10.1007/s11548-021-02345-w	2021	Unclassified	Deep Learning (DL)	Segmentation & Measurement
1993	10.1007/s00256-021-03830-8	2022	Sports Medicine; Trauma; Arthroplasty	Deep Learning (DL)	Prognosis & Prediction
1994	10.2106/JBJS.21.01305	2022	Spine Surgery	Machine Learning (ML); Deep Learning (DL)	Prognosis & Prediction
1995	10.1097/CORR.0000000000002995	2024	Unclassified	Natural Language Processing (NLP)	Unclassified
1996	10.1302/0301-620X.102B6.BJJ-2019-1577.R1	2020	Unclassified	Unclassified	Diagnosis & Detection
1997	10.1016/j.wneu.2022.03.096	2022	Spine Surgery	Machine Learning (ML); Deep Learning (DL)	Prognosis & Prediction
1998	10.5312/wjo.v15.i2.105	2024	Unclassified	Deep Learning (DL)	Diagnosis & Detection
1999	10.1016/j.spinee.2024.06.009	2024	Spine Surgery	Natural Language Processing (NLP); Deep Learning (DL)	Prognosis & Prediction
2000	10.1016/j.arth.2023.02.054	2023	Arthroplasty	Machine Learning (ML); Deep Learning (DL)	Prognosis & Prediction
2001	10.1007/s10143-019-01163-8	2020	Spine Surgery	Natural Language Processing (NLP); Machine Learning (ML); Deep Learning (DL)	Prognosis & Prediction
2002	10.1097/BSD.0000000000001082	2021	Trauma; Spine Surgery	Machine Learning (ML); Deep Learning (DL)	Prognosis & Prediction

2003	10.1002/jor.26086	2025	Arthroplasty	Deep Learning (DL)	Surgical Planning
2004	10.1016/j.jot.2022.05.006	2022	Sports Medicine; Trauma	Deep Learning (DL)	Diagnosis & Detection
2005	10.1007/s00264-023-05813-x	2023	Unclassified	Deep Learning (DL)	Diagnosis & Detection
2006	10.1007/s00586-025-09168-2	2025	Trauma; Spine Surgery	Machine Learning (ML); Deep Learning (DL)	Prognosis & Prediction
2007	10.1007/s12178-016-9354-7	2016	Spine Surgery	Machine Learning (ML)	Prognosis & Prediction
2008	10.1016/j.wneu.2020.10.073	2021	Spine Surgery	Machine Learning (ML); Deep Learning (DL)	Prognosis & Prediction
2009	10.1007/s11548-020-02266-0	2020	Pediatric Orthopaedics	Deep Learning (DL)	Segmentation & Measurement
2010	10.1080/13645706.2021.1980402	2022	Arthroplasty	Deep Learning (DL)	Unclassified
2011	10.1007/s00256-024-04618-2	2024	Unclassified	Deep Learning (DL)	Prognosis & Prediction
2012	10.4240/wjgs.v14.i8.833	2022	Arthroplasty	Machine Learning (ML); Deep Learning (DL)	Prognosis & Prediction
2013	10.1177/21925682221098672	2024	Pediatric Orthopaedics; Spine Surgery	Deep Learning (DL)	Segmentation & Measurement
2014	10.1016/j.spinee.2018.11.009	2019	Spine Surgery	Natural Language Processing (NLP); Machine Learning (ML); Deep Learning (DL)	Prognosis & Prediction
2015	10.1097/SCS.0000000000008620	2022	Pediatric Orthopaedics	Deep Learning (DL)	Prognosis & Prediction
2016	10.1007/s00256-021-03740-9	2022	Trauma; Arthroplasty	Deep Learning (DL)	Diagnosis & Detection
2017	10.1016/j.gaitpost.2025.01.028	2025	Sports Medicine; Arthroplasty	Machine Learning (ML); Deep Learning (DL)	Prognosis & Prediction
2018	10.1007/s00586-025-09541-1	2025	Sports Medicine; Spine Surgery	Deep Learning (DL)	Segmentation & Measurement
2019	10.1177/19476035211042406	2021	Sports Medicine	Deep Learning (DL)	Segmentation & Measurement
2020	10.1007/s11548-021-02474-2	2021	Unclassified	Deep Learning (DL)	Diagnosis & Detection
2021	10.3171/2025.4.FOCUS25170	2025	Spine Surgery	Deep Learning (DL)	Unclassified
2022	10.1016/j.injury.2020.09.010	2021	Trauma	Deep Learning (DL)	Unclassified

2023	10.1016/j.ocarto.2025.100645	2025	Sports Medicine	Deep Learning (DL)	Segmentation & Measurement
2024	10.1007/s00264-023-05788-9	2023	Unclassified	Machine Learning (ML); Deep Learning (DL)	Prognosis & Prediction
2025	10.1097/BRS.0000000000003377	2020	Spine Surgery	Deep Learning (DL)	Unclassified
2026	10.1016/j.apmr.2019.03.016	2019	Pediatric Orthopaedics	Deep Learning (DL)	Unclassified
2027	10.5397/cise.2025.00185	2025	Arthroplasty	Machine Learning (ML); Robotics; Deep Learning (DL)	Prognosis & Prediction
2028	10.1097/BSD.0000000000001748	2025	Spine Surgery	Deep Learning (DL)	Prognosis & Prediction
2029	10.1089/sur.2024.266	2025	Spine Surgery; Arthroplasty	Natural Language Processing (NLP); Machine Learning (ML); Deep Learning (DL)	Prognosis & Prediction
2030	10.1186/s12891-024-08034-4	2024	Arthroplasty	Machine Learning (ML); Deep Learning (DL)	Prognosis & Prediction
2031	10.1007/s11548-021-02436-8	2021	Unclassified	Deep Learning (DL)	Prognosis & Prediction
2032	10.1007/s00586-022-07176-0	2022	Pediatric Orthopaedics; Trauma; Spine Surgery	Natural Language Processing (NLP); Machine Learning (ML); Deep Learning (DL)	Prognosis & Prediction
2033	10.1002/ohn.317	2023	Unclassified	Machine Learning (ML); Deep Learning (DL)	Prognosis & Prediction
2034	10.20517/ais.2024.35	2025	Spine Surgery	Machine Learning (ML); Deep Learning (DL)	Unclassified
2035	10.1007/978-3-031-55315-8_20	2024	Arthroplasty	Deep Learning (DL)	Unclassified
2036	10.1016/j.jot.2025.01.007	2025	Sports Medicine	Machine Learning (ML); Deep Learning (DL)	Prognosis & Prediction
2037	10.1007/s12178-025-09992-5	2025	Spine Surgery	Machine Learning (ML); Robotics; Deep Learning (DL)	Prognosis & Prediction
2038	10.1016/j.gaitpost.2024.06.010	2024	Arthroplasty	Deep Learning (DL)	Prognosis & Prediction
2039	10.14444/7130	2020	Spine Surgery	Deep Learning (DL)	Prognosis & Prediction
2040	10.62713/aic.3666	2025	Trauma	Machine Learning (ML); Deep Learning (DL)	Prognosis & Prediction
2041	10.1016/j.jor.2023.03.001	2023	Spine Surgery	Deep Learning (DL)	Prognosis & Prediction
2042	10.1002/rcs.70008	2024	Trauma	Robotics; Deep Learning (DL)	Surgical Planning

2043	10.1016/j.joca.2018.02.907	2018	Sports Medicine	Deep Learning (DL)	Prognosis & Prediction
2044	10.1007/978-3-030-87202-1_17	2021	Pediatric Orthopaedics; Arthroplasty	Deep Learning (DL)	Unclassified
2045	10.31616/asj.2024.0197	2024	Spine Surgery	Natural Language Processing (NLP); Deep Learning (DL)	Prognosis & Prediction
2046	10.1016/j.arthro.2022.06.032	2023	Sports Medicine	Machine Learning (ML); Deep Learning (DL)	Prognosis & Prediction
2047	10.1002/rcs.2228	2021	Spine Surgery	Deep Learning (DL)	Unclassified
2048	10.3171/2023.3.FOCUS2390	2023	Sports Medicine; Spine Surgery	Machine Learning (ML); Deep Learning (DL)	Diagnosis & Detection
2049	10.1016/j.ocarto.2025.100663	2025	Sports Medicine; Arthroplasty	Deep Learning (DL)	Prognosis & Prediction
2050	10.1007/s00586-025-09167-3	2025	Spine Surgery	Machine Learning (ML); Deep Learning (DL)	Surgical Planning
2051	10.1186/s43019-024-00256-z	2025	Arthroplasty	Deep Learning (DL)	Prognosis & Prediction
2052	10.1016/j.arth.2023.02.053	2023	Arthroplasty	Deep Learning (DL)	Prognosis & Prediction
2053	10.1007/s11548-022-02783-0	2023	Sports Medicine; Spine Surgery	Deep Learning (DL)	Prognosis & Prediction
2054	10.1016/j.knee.2025.04.011	2025	Arthroplasty	Natural Language Processing (NLP); Machine Learning (ML); Robotics; Deep Learning (DL)	Prognosis & Prediction
2055	10.1097/TA.0000000000003384	2021	Trauma; Spine Surgery	Deep Learning (DL)	Prognosis & Prediction
2056	10.1016/j.jhsa.2025.04.015	2025	Trauma; Pediatric Orthopaedics	Deep Learning (DL)	Prognosis & Prediction
2057	10.1002/jsp2.1204	2022	Spine Surgery	Deep Learning (DL)	Prognosis & Prediction
2058	10.5114/jhk/163059	2023	Trauma; Arthroplasty	Machine Learning (ML); Deep Learning (DL)	Prognosis & Prediction
2059	10.1002/jor.25761	2024	Unclassified	Deep Learning (DL)	Prognosis & Prediction
2060	10.1007/s43465-024-01246-9	2024	Unclassified	Machine Learning (ML); Deep Learning (DL)	Prognosis & Prediction
2061	10.1186/s12891-020-03768-3	2020	Sports Medicine	Deep Learning (DL)	Segmentation & Measurement
2062	10.1016/j.jisako.2024.01.013	2024	Sports Medicine	Natural Language Processing (NLP); Machine Learning (ML); Deep Learning (DL)	Prognosis & Prediction

2063	10.1097/SCS.0000000000010663	2024	Arthroplasty	Machine Learning (ML); Deep Learning (DL)	Prognosis & Prediction
2064	10.1186/s13018-024-05340-4	2024	Sports Medicine	Machine Learning (ML); Deep Learning (DL)	Diagnosis & Detection
2065	10.1016/j.jse.2024.01.033	2024	Arthroplasty	Machine Learning (ML); Robotics; Deep Learning (DL)	Prognosis & Prediction
2066	10.1007/s11657-024-01443-x	2024	Trauma	Unclassified	Prognosis & Prediction
2067	10.1007/s00381-020-04671-x	2020	Trauma; Pediatric Orthopaedics	Deep Learning (DL)	Prognosis & Prediction
2068	10.3171/2021.11.SPINE211136	2022	Spine Surgery	Deep Learning (DL)	Diagnosis & Detection
2069	10.1007/s11548-024-03073-7	2024	Unclassified	Deep Learning (DL)	Segmentation & Measurement
2070	10.1007/s11548-021-02335-y	2021	Arthroplasty	Machine Learning (ML); Deep Learning (DL)	Prognosis & Prediction
2071	10.1186/s12891-024-08120-7	2024	Unclassified	Natural Language Processing (NLP); Machine Learning (ML); Deep Learning (DL)	Prognosis & Prediction
2072	10.2106/JBJS.OA.23.00039	2023	Trauma	Deep Learning (DL)	Prognosis & Prediction
2073	10.1016/j.wneu.2023.10.042	2024	Spine Surgery	Machine Learning (ML); Deep Learning (DL)	Prognosis & Prediction
2074	10.1007/s11548-025-03465-3	2025	Unclassified	Robotics; Deep Learning (DL)	Diagnosis & Detection
2075	10.1186/s12891-025-08356-x	2025	Spine Surgery	Machine Learning (ML); Deep Learning (DL)	Prognosis & Prediction
2076	10.14529/hsm180206	2018	Sports Medicine; Arthroplasty	Deep Learning (DL)	Unclassified
2077	10.20517/ais.2024.36	2025	Sports Medicine; Spine Surgery	Deep Learning (DL)	Segmentation & Measurement
2078	10.1016/j.otsr.2023.103652	2023	Unclassified	Deep Learning (DL)	Unclassified
2079	10.1097/BRS.0000000000003865	2021	Spine Surgery	Machine Learning (ML); Deep Learning (DL)	Prognosis & Prediction
2080	10.1007/s11548-022-02558-7	2022	Arthroplasty	Robotics; Deep Learning (DL)	Diagnosis & Detection
2081	10.1007/s11548-019-01934-0	2019	Unclassified	Deep Learning (DL)	Segmentation & Measurement
2082	10.1177/15563316221120582	2023	Trauma	Machine Learning (ML); Deep Learning (DL)	Prognosis & Prediction

2083	10.1097/BSD.0000000000001208	2022	Spine Surgery	Machine Learning (ML)	Prognosis & Prediction
2084	10.1186/s13018-023-04144-2	2023	Trauma; Spine Surgery	Deep Learning (DL)	Prognosis & Prediction
2085	10.3389/fsurg.2022.798761	2022	Arthroplasty	Deep Learning (DL)	Prognosis & Prediction
2086	10.1016/j.injury.2019.08.033	2020	Sports Medicine; Trauma	Machine Learning (ML); Deep Learning (DL)	Prognosis & Prediction
2087	10.1097/BOT.0000000000001663	2020	Trauma	Machine Learning (ML); Deep Learning (DL)	Prognosis & Prediction
2088	10.1097/BRS.0000000000004172	2022	Spine Surgery; Arthroplasty	Machine Learning (ML); Deep Learning (DL)	Prognosis & Prediction
2089	10.1007/s11548-025-03388-z	2025	Trauma; Spine Surgery	Deep Learning (DL)	Prognosis & Prediction
2090	10.1002/jor.25557	2023	Arthroplasty	Machine Learning (ML); Deep Learning (DL)	Diagnosis & Detection
2091	10.1007/s00256-021-03876-8	2022	Sports Medicine	Machine Learning (ML); Deep Learning (DL)	Diagnosis & Detection
2092	10.1016/j.arth.2022.12.017	2023	Arthroplasty	Deep Learning (DL)	Prognosis & Prediction
2093	10.1123/jab.2020-0222	2021	Trauma	Deep Learning (DL)	Prognosis & Prediction
2094	10.1111/iwj.14607	2024	Pediatric Orthopaedics; Spine Surgery	Machine Learning (ML); Deep Learning (DL)	Prognosis & Prediction
2095	10.14245/ns.2244272.136	2022	Trauma	Unclassified	Unclassified
2096	10.1007/s00256-022-04160-z	2023	Unclassified	Deep Learning (DL)	Diagnosis & Detection
2097	10.1016/j.jor.2022.11.004	2023	Arthroplasty	Machine Learning (ML); Deep Learning (DL)	Prognosis & Prediction
2098	10.1016/j.clinbiomech.2022.105682	2022	Arthroplasty	Natural Language Processing (NLP)	Segmentation & Measurement
2099	10.2106/JBJS.RVW.20.00247	2021	Trauma	Unclassified	Prognosis & Prediction
2100	10.1186/s13018-023-03909-z	2023	Arthroplasty	Deep Learning (DL)	Diagnosis & Detection
2101	10.1016/j.jor.2024.06.016	2024	Pediatric Orthopaedics; Arthroplasty	Machine Learning (ML); Deep Learning (DL)	Prognosis & Prediction
2102	10.1007/s00113-025-01653-z	2025	Trauma	Natural Language Processing (NLP); Deep Learning (DL)	Diagnosis & Detection
2103	10.52312/jdrs.2023.996	2023	Unclassified	Deep Learning (DL)	Diagnosis & Detection

2104	10.5312/wjo.v14.i10.741	2023	Trauma; Arthroplasty	Machine Learning (ML); Deep Learning (DL)	Prognosis & Prediction
2105	10.1007/s00256-018-3033-2	2019	Pediatric Orthopaedics	Machine Learning (ML); Deep Learning (DL)	Diagnosis & Detection
2106	10.1007/s00264-022-05517-8	2023	Trauma; Spine Surgery	Machine Learning (ML)	Prognosis & Prediction
2107	10.1007/s00590-025-04592-y	2025	Trauma	Deep Learning (DL)	Prognosis & Prediction
2108	10.1007/s00256-024-04634-2	2024	Spine Surgery; Arthroplasty	Deep Learning (DL)	Unclassified
2109	10.20517/ais.2024.41	2025	Trauma; Spine Surgery	Machine Learning (ML); Deep Learning (DL)	Prognosis & Prediction
2110	10.21037/jss-24-8	2024	Trauma; Spine Surgery	Machine Learning (ML); Deep Learning (DL)	Prognosis & Prediction
2111	10.23736/S0390-5616.25.06506-3	2025	Spine Surgery	Machine Learning (ML); Deep Learning (DL)	Unclassified
2112	10.1007/s00586-022-07188-w	2022	Unclassified	Machine Learning (ML); Deep Learning (DL)	Prognosis & Prediction
2113	10.1186/s13018-023-04509-7	2024	Unclassified	Deep Learning (DL)	Segmentation & Measurement
2114	10.1007/s43390-025-01208-7	2025	Pediatric Orthopaedics; Spine Surgery	Natural Language Processing (NLP); Machine Learning (ML); Deep Learning (DL)	Prognosis & Prediction
2115	10.1016/j.afos.2024.01.001	2024	Trauma; Spine Surgery	Unclassified	Diagnosis & Detection
2116	10.3389/fsurg.2023.1325423	2024	Trauma	Robotics	Unclassified
2117	10.1016/j.ocarto.2022.100234	2022	Sports Medicine	Deep Learning (DL)	Segmentation & Measurement
2118	10.1016/j.ijom.2025.04.005	2025	Unclassified	Machine Learning (ML); Deep Learning (DL)	Prognosis & Prediction
2119	10.1007/s00586-019-05944-z	2019	Pediatric Orthopaedics; Spine Surgery; Arthroplasty	Deep Learning (DL)	Prognosis & Prediction
2120	10.1227/NEU.0000000000001736	2022	Spine Surgery	Machine Learning (ML); Deep Learning (DL)	Prognosis & Prediction
2121	10.3389/fsurg.2024.1458569	2024	Spine Surgery	Deep Learning (DL)	Prognosis & Prediction
2122	10.20408/jti.2025.0100	2025	Trauma; Spine Surgery	Machine Learning (ML); Deep Learning (DL)	Prognosis & Prediction

2123	10.1186/s13018-025-05564-y	2025	Pediatric Orthopaedics	Deep Learning (DL)	Prognosis & Prediction
2124	10.1186/s12891-022-05821-9	2022	Unclassified	Unclassified	Prognosis & Prediction
2125	10.1007/s00256-019-03342-6	2020	Trauma; Spine Surgery	Machine Learning (ML); Deep Learning (DL)	Prognosis & Prediction
2126	10.1016/j.jot.2025.06.016	2025	Arthroplasty	Machine Learning (ML); Deep Learning (DL)	Prognosis & Prediction
2127	10.5435/JAAOS-D-21-00987	2022	Arthroplasty	Machine Learning (ML); Deep Learning (DL)	Prognosis & Prediction
2128	10.1186/s12891-025-08841-3	2025	Pediatric Orthopaedics; Spine Surgery	Machine Learning (ML); Deep Learning (DL)	Prognosis & Prediction
2129	10.1007/s43465-024-01134-2	2024	Sports Medicine; Spine Surgery	Robotics; Deep Learning (DL)	Diagnosis & Detection
2130	10.1007/s11548-020-02204-0	2020	Spine Surgery; Trauma; Arthroplasty	Deep Learning (DL)	Prognosis & Prediction
2131	10.1002/jsp2.1353	2024	Sports Medicine; Spine Surgery; Arthroplasty	Machine Learning (ML); Deep Learning (DL)	Unclassified
2132	10.1016/j.spinee.2019.06.024	2019	Spine Surgery	Natural Language Processing (NLP); Machine Learning (ML)	Prognosis & Prediction
2133	10.1097/TA.0000000000003913	2023	Trauma	Machine Learning (ML); Deep Learning (DL)	Prognosis & Prediction
2134	10.1007/s11548-022-02597-0	2022	Unclassified	Deep Learning (DL)	Prognosis & Prediction
2135	10.1186/s12891-025-09275-7	2025	Unclassified	Deep Learning (DL)	Diagnosis & Detection
2136	10.1097/JS9.0000000000002210	2025	Spine Surgery	Deep Learning (DL)	Prognosis & Prediction
2137	10.1002/jor.24984	2022	Sports Medicine	Deep Learning (DL)	Segmentation & Measurement
2138	10.1007/s40279-016-0666-y	2017	Sports Medicine; Trauma; Arthroplasty	Deep Learning (DL)	Prognosis & Prediction
2139	10.1177/15589447241308603	2025	Trauma	Machine Learning (ML); Deep Learning (DL)	Diagnosis & Detection
2140	10.1016/j.wneu.2019.03.165	2019	Trauma; Spine Surgery	Machine Learning (ML)	Prognosis & Prediction
2141	10.1007/s00402-024-05542-9	2024	Arthroplasty	Machine Learning (ML); Deep Learning (DL)	Prognosis & Prediction
2142	10.1016/j.joca.2020.12.022	2021	Sports Medicine	Natural Language Processing (NLP); Machine Learning (ML)	Prognosis & Prediction

2143	10.1007/978-3-030-87202-1_51	2021	Trauma; Spine Surgery	Deep Learning (DL)	Prognosis & Prediction
2144	10.1002/jor.70059	2025	Arthroplasty	Machine Learning (ML); Deep Learning (DL)	Prognosis & Prediction
2145	10.1097/BRS.0000000000004438	2023	Spine Surgery	Machine Learning (ML); Deep Learning (DL)	Diagnosis & Detection
2146	10.1016/j.artd.2023.101116	2023	Arthroplasty	Machine Learning (ML); Deep Learning (DL)	Prognosis & Prediction
2147	10.1007/s00586-017-4956-3	2017	Spine Surgery	Deep Learning (DL)	Diagnosis & Detection
2148	10.1016/j.wneu.2025.124468	2025	Spine Surgery	Machine Learning (ML); Deep Learning (DL)	Prognosis & Prediction
2149	10.3171/CASE23120	2023	Trauma; Spine Surgery	Robotics; Deep Learning (DL)	Unclassified
2150	10.1007/s11548-023-02941-y	2023	Trauma	Machine Learning (ML); Robotics; Deep Learning (DL)	Unclassified
2151	10.1002/jor.24991	2021	Pediatric Orthopaedics	Machine Learning (ML); Deep Learning (DL)	Prognosis & Prediction
2152	10.1530/EOR-23-0174	2024	Trauma	Unclassified	Diagnosis & Detection
2153	10.1007/s11548-021-02388-z	2021	Arthroplasty	Robotics; Deep Learning (DL)	Prognosis & Prediction
2154	10.1097/PRS.0000000000010928	2024	Trauma	Deep Learning (DL)	Diagnosis & Detection
2155	10.1016/j.ocarto.2022.100290	2022	Sports Medicine	Deep Learning (DL)	Prognosis & Prediction
2156	10.1007/s11657-024-01433-z	2024	Trauma; Spine Surgery	Machine Learning (ML); Deep Learning (DL)	Prognosis & Prediction
2157	10.1002/jor.25902	2024	Arthroplasty	Deep Learning (DL)	Prognosis & Prediction
2158	10.1002/lsm.23473	2022	Unclassified	Deep Learning (DL)	Diagnosis & Detection
2159	10.1007/s00113-025-01536-3	2025	Arthroplasty	Deep Learning (DL)	Unclassified
2160	10.1177/03635465221137875	2023	Sports Medicine; Trauma	Machine Learning (ML)	Prognosis & Prediction
2161	10.1007/s43390-024-00933-9	2024	Pediatric Orthopaedics; Spine Surgery	Deep Learning (DL)	Surgical Planning
2162	10.1186/s12891-021-04514-z	2021	Pediatric Orthopaedics; Arthroplasty	Machine Learning (ML)	Diagnosis & Detection
2163	10.1007/s11548-019-02044-7	2019	Arthroplasty	Deep Learning (DL)	Prognosis & Prediction

2164	10.1093/neuros/nyz070	2019	Spine Surgery	Machine Learning (ML)	Prognosis & Prediction
2165	10.1016/j.arth.2023.01.065	2023	Arthroplasty	Machine Learning (ML); Deep Learning (DL)	Prognosis & Prediction
2166	10.1007/978-3-030-87202-1_43	2021	Trauma; Arthroplasty	Robotics; Deep Learning (DL)	Surgical Planning
2167	10.20517/ais.2024.40	2024	Trauma; Spine Surgery	Machine Learning (ML); Deep Learning (DL)	Prognosis & Prediction
2168	10.1007/s00586-021-06799-z	2021	Spine Surgery	Deep Learning (DL)	Prognosis & Prediction
2169	10.1302/2046-3758.1310.BJR-2023-0275.R3	2024	Trauma	Deep Learning (DL)	Diagnosis & Detection
2170	10.1177/1553350621996961	2021	Unclassified	Machine Learning (ML); Deep Learning (DL)	Prognosis & Prediction
2171	10.1016/j.jor.2025.06.006	2025	Arthroplasty	Machine Learning (ML); Deep Learning (DL)	Prognosis & Prediction
2172	10.3389/fsurg.2024.1441346	2024	Trauma	Machine Learning (ML); Deep Learning (DL)	Diagnosis & Detection
2173	10.3389/fsurg.2024.1387351	2024	Sports Medicine; Trauma; Arthroplasty	Deep Learning (DL)	Prognosis & Prediction
2174	10.2106/JBJS.OA.22.00030	2022	Trauma	Deep Learning (DL)	Diagnosis & Detection
2175	10.1007/s00586-025-09415-6	2025	Trauma; Spine Surgery	Natural Language Processing (NLP); Deep Learning (DL)	Prognosis & Prediction
2176	10.1177/21925682221097650	2024	Spine Surgery; Arthroplasty	Machine Learning (ML); Deep Learning (DL)	Prognosis & Prediction
2177	10.1016/j.arth.2024.10.129	2025	Arthroplasty	Natural Language Processing (NLP); Machine Learning (ML); Deep Learning (DL)	Prognosis & Prediction
2178	10.1097/BRS.0000000000003844	2021	Spine Surgery	Deep Learning (DL)	Unclassified
2179	10.1177/21925682241270036	2025	Pediatric Orthopaedics; Spine Surgery	Deep Learning (DL)	Surgical Planning
2180	10.1007/s00256-024-04692-6	2026	Trauma	Deep Learning (DL)	Prognosis & Prediction
2181	10.1007/s10143-024-02366-4	2024	Pediatric Orthopaedics; Arthroplasty	Natural Language Processing (NLP); Deep Learning (DL)	Prognosis & Prediction
2182	10.1007/s10143-021-01624-z	2022	Spine Surgery	Machine Learning (ML); Robotics	Prognosis & Prediction

2183	10.1016/j.arth.2023.08.018	2024	Trauma	Deep Learning (DL)	Prognosis & Prediction
2184	10.1177/2192568220963060	2021	Unclassified	Unclassified	Prognosis & Prediction
2185	10.1002/jeo2.70317	2025	Arthroplasty	Machine Learning (ML); Deep Learning (DL)	Prognosis & Prediction
2186	10.3389/fsurg.2022.850342	2022	Spine Surgery	Deep Learning (DL)	Prognosis & Prediction
2187	10.1007/s00256-022-04041-5	2022	Arthroplasty	Deep Learning (DL)	Prognosis & Prediction
2188	10.1177/10556656241299890	2026	Sports Medicine; Trauma; Spine Surgery	Deep Learning (DL)	Diagnosis & Detection
2189	10.3390/sports13090298	2025	Trauma; Arthroplasty	Machine Learning (ML); Deep Learning (DL)	Prognosis & Prediction
2190	10.1002/ohn.470	2023	Sports Medicine	Deep Learning (DL)	Diagnosis & Detection
2191	10.1016/j.ocarto.2025.100657	2025	Sports Medicine; Arthroplasty	Deep Learning (DL)	Diagnosis & Detection
2192	10.1302/2046-3758.129.BJR-2023-0070.R2	2023	Trauma; Arthroplasty	Machine Learning (ML); Deep Learning (DL)	Prognosis & Prediction
2193	10.1002/lsm.70031	2025	Unclassified	Machine Learning (ML); Deep Learning (DL)	Prognosis & Prediction
2194	10.1007/s11548-020-02184-1	2020	Unclassified	Natural Language Processing (NLP); Deep Learning (DL)	Segmentation & Measurement
2195	10.1097/BRS.0000000000005100	2024	Spine Surgery	Deep Learning (DL)	Prognosis & Prediction
2196	10.1080/17453674.2018.1453714	2018	Trauma	Deep Learning (DL)	Diagnosis & Detection
2197	10.1002/ksa.70029	2025	Arthroplasty	Machine Learning (ML); Deep Learning (DL)	Prognosis & Prediction
2198	10.1007/s00586-025-09281-2	2025	Spine Surgery	Deep Learning (DL)	Diagnosis & Detection
2199	10.1016/j.clinbiomech.2024.106188	2024	Trauma; Arthroplasty	Machine Learning (ML); Deep Learning (DL)	Prognosis & Prediction
2200	10.1016/j.wneu.2023.07.103	2023	Arthroplasty	Machine Learning (ML); Deep Learning (DL)	Surgical Planning
2201	10.1007/s00264-024-06194-5	2024	Trauma	Machine Learning (ML); Deep Learning (DL)	Prognosis & Prediction
2202	10.14444/7132	2020	Spine Surgery	Deep Learning (DL)	Prognosis & Prediction
2203	10.1002/jor.25269	2022	Spine Surgery; Arthroplasty	Machine Learning (ML)	Prognosis & Prediction

2204	10.1016/j.arth.2024.02.001	2024	Arthroplasty	Machine Learning (ML); Deep Learning (DL)	Surgical Planning
2205	10.1016/j.wneu.2018.07.276	2018	Spine Surgery	Machine Learning (ML)	Prognosis & Prediction
2206	10.1117/12.2612913	2022	Trauma	Deep Learning (DL)	Segmentation & Measurement
2207	10.1227/ons.0000000000000274	2022	Spine Surgery	Machine Learning (ML); Deep Learning (DL)	Diagnosis & Detection
2208	10.1016/j.otsr.2025.104238	2025	Arthroplasty	Machine Learning (ML); Deep Learning (DL)	Prognosis & Prediction
2209	10.1016/j.joca.2021.06.011	2021	Arthroplasty	Machine Learning (ML); Deep Learning (DL)	Prognosis & Prediction
2210	10.23736/S0022-4707.24.15759-3	2024	Trauma; Arthroplasty	Machine Learning (ML)	Diagnosis & Detection
2211	10.1016/j.oto.2019.100712	2019	Spine Surgery	Machine Learning (ML); Deep Learning (DL)	Prognosis & Prediction
2212	10.1002/jeo2.70080	2024	Sports Medicine	Machine Learning (ML); Deep Learning (DL)	Prognosis & Prediction
2213	10.1111/os.70034	2025	Trauma	Deep Learning (DL)	Prognosis & Prediction
2214	10.1016/j.arth.2025.04.072	2025	Arthroplasty	Machine Learning (ML); Deep Learning (DL)	Prognosis & Prediction
2215	10.1002/jeo2.12104	2024	Arthroplasty	Machine Learning (ML); Deep Learning (DL)	Prognosis & Prediction
2216	10.1097/BRS.0000000000004595	2023	Spine Surgery	Deep Learning (DL)	Diagnosis & Detection
2217	10.1007/s11548-015-1278-y	2016	Spine Surgery	Deep Learning (DL)	Diagnosis & Detection
2218	10.1016/j.ijom.2025.02.009	2025	Sports Medicine; Arthroplasty	Deep Learning (DL)	Prognosis & Prediction
2219	10.1007/s00167-023-07565-y	2023	Arthroplasty	Machine Learning (ML); Deep Learning (DL)	Diagnosis & Detection
2220	10.1186/s12891-022-05378-7	2022	Sports Medicine; Trauma	Deep Learning (DL)	Prognosis & Prediction
2221	10.1016/j.joca.2020.01.010	2020	Sports Medicine; Arthroplasty	Natural Language Processing (NLP); Deep Learning (DL)	Prognosis & Prediction
2222	10.3171/2019.9.SPINE19860	2020	Spine Surgery	Machine Learning (ML)	Prognosis & Prediction
2223	10.1080/02640414.2025.2578584	2025	Sports Medicine; Trauma	Machine Learning (ML); Deep Learning (DL)	Unclassified
2224	10.1007/s00264-022-05675-9	2023	Unclassified	Deep Learning (DL)	Segmentation & Measurement

2225	10.22603/ssrr.2023-0269	2024	Pediatric Orthopaedics	Deep Learning (DL)	Surgical Planning
2226	10.1055/s-0035-1570364	2016	Spine Surgery	Natural Language Processing (NLP)	Diagnosis & Detection
2227	10.1007/s11548-024-03315-8	2025	Arthroplasty	Deep Learning (DL)	Diagnosis & Detection
2228	10.5312/wjo.v15.i12.1146	2024	Unclassified	Deep Learning (DL)	Prognosis & Prediction
2229	10.3171/2020.10.FOCUS20801	2021	Spine Surgery	Machine Learning (ML); Robotics; Deep Learning (DL)	Surgical Planning
2230	10.1016/j.xnsj.2024.100513	2024	Trauma; Spine Surgery	Natural Language Processing (NLP); Deep Learning (DL)	Prognosis & Prediction
2231	10.1186/s10195-025-00846-x	2025	Trauma	Deep Learning (DL)	Prognosis & Prediction
2232	10.5312/wjo.v16.i6.103832	2025	Sports Medicine	Machine Learning (ML); Deep Learning (DL)	Surgical Planning
2233	10.1007/s00421-017-3630-0	2017	Pediatric Orthopaedics	Machine Learning (ML)	Unclassified
2234	10.1007/s00256-019-03289-8	2020	Unclassified	Deep Learning (DL)	Prognosis & Prediction
2235	10.1007/s11548-021-02505-y	2022	Arthroplasty	Deep Learning (DL)	Segmentation & Measurement
2236	10.1007/s11548-018-1883-7	2019	Unclassified	Deep Learning (DL)	Surgical Planning
2237	10.1097/CORR.0000000000000755	2019	Sports Medicine; Arthroplasty	Unclassified	Prognosis & Prediction
2238	10.21037/jss-23-89	2024	Spine Surgery	Machine Learning (ML)	Prognosis & Prediction
2239	10.1007/s00402-022-04391-8	2023	Arthroplasty	Machine Learning (ML); Deep Learning (DL)	Prognosis & Prediction
2240	10.1016/j.wneu.2021.08.136	2021	Unclassified	Deep Learning (DL)	Prognosis & Prediction
2241	10.1007/s11548-020-02172-5	2020	Arthroplasty	Machine Learning (ML); Deep Learning (DL)	Prognosis & Prediction
2242	10.1002/jfa2.70081	2025	Unclassified	Deep Learning (DL)	Segmentation & Measurement
2243	10.1177/17543371251348697	2025	Arthroplasty	Deep Learning (DL)	Prognosis & Prediction
2244	10.1016/j.knee.2024.11.006	2025	Sports Medicine; Arthroplasty	Natural Language Processing (NLP); Deep Learning (DL)	Diagnosis & Detection
2245	10.1016/j.xnsj.2022.100196	2023	Spine Surgery	Machine Learning (ML)	Prognosis & Prediction
2246	10.1007/s00256-021-03773-0	2022	Sports Medicine; Arthroplasty	Deep Learning (DL)	Prognosis & Prediction

2247	10.1016/j.arthro.2023.09.023	2024	Arthroplasty	Machine Learning (ML); Deep Learning (DL)	Prognosis & Prediction
2248	10.3389/fsurg.2023.1259756	2023	Spine Surgery	Machine Learning (ML); Deep Learning (DL)	Prognosis & Prediction
2249	10.14444/8507	2023	Spine Surgery	Natural Language Processing (NLP); Machine Learning (ML); Deep Learning (DL)	Prognosis & Prediction
2250	10.1007/s11548-022-02688-y	2022	Arthroplasty	Deep Learning (DL)	Unclassified
2251	10.1007/s00586-023-07909-9	2023	Spine Surgery	Machine Learning (ML); Deep Learning (DL)	Prognosis & Prediction
2252	10.1007/s00586-025-08697-0	2025	Trauma; Spine Surgery	Deep Learning (DL)	Prognosis & Prediction
2253	10.1097/BRS.0000000000004816	2023	Spine Surgery	Machine Learning (ML); Deep Learning (DL)	Prognosis & Prediction
2254	10.1002/ksa.12492	2025	Arthroplasty	Machine Learning (ML); Deep Learning (DL)	Prognosis & Prediction
2255	10.2106/JBJS.OA.25.00257	2025	Pediatric Orthopaedics; Spine Surgery	Deep Learning (DL)	Prognosis & Prediction
2256	10.1177/23259671231221579	2024	Sports Medicine; Trauma	Deep Learning (DL)	Prognosis & Prediction
2257	10.1002/ejsc.12186	2024	Arthroplasty	Deep Learning (DL)	Diagnosis & Detection
2258	10.1007/s00402-023-05013-7	2023	Arthroplasty	Machine Learning (ML); Deep Learning (DL)	Prognosis & Prediction
2259	10.1186/s12891-024-08228-w	2025	Sports Medicine; Trauma	Machine Learning (ML); Deep Learning (DL)	Prognosis & Prediction
2260	10.2106/JBJS.23.00549	2024	Arthroplasty	Deep Learning (DL)	Prognosis & Prediction
2261	10.1007/s11548-021-02329-w	2021	Trauma; Arthroplasty	Unclassified	Segmentation & Measurement
2262	10.1016/j.clineuro.2020.105718	2020	Spine Surgery	Machine Learning (ML)	Prognosis & Prediction
2263	10.22603/ssrr.2024-0154	2024	Spine Surgery	Machine Learning (ML); Deep Learning (DL)	Prognosis & Prediction
2264	10.1016/j.arth.2022.10.003	2023	Trauma; Arthroplasty	Deep Learning (DL)	Prognosis & Prediction
2265	10.1177/23259671251334138	2025	Unclassified	Machine Learning (ML); Deep Learning (DL)	Surgical Planning
2266	10.5312/wjo.v14.i11.800	2023	Unclassified	Deep Learning (DL)	Surgical Planning

2267	10.1016/j.joca.2020.12.018	2021	Sports Medicine	Machine Learning (ML); Deep Learning (DL)	Unclassified
2268	10.3171/2018.8.FOCUS18340	2018	Spine Surgery	Machine Learning (ML)	Prognosis & Prediction
2269	10.1111/iwj.14275	2023	Trauma	Machine Learning (ML); Deep Learning (DL)	Prognosis & Prediction
2270	10.1016/j.spinee.2024.12.025	2025	Trauma; Spine Surgery	Natural Language Processing (NLP); Deep Learning (DL)	Prognosis & Prediction
2271	10.1111/os.14280	2025	Spine Surgery	Deep Learning (DL)	Diagnosis & Detection
2272	10.1016/j.wneu.2024.11.048	2025	Spine Surgery	Machine Learning (ML); Deep Learning (DL)	Prognosis & Prediction
2273	10.1016/j.spinee.2019.06.002	2019	Spine Surgery	Natural Language Processing (NLP); Machine Learning (ML)	Prognosis & Prediction
2274	10.1186/s13018-024-05010-5	2024	Arthroplasty	Deep Learning (DL)	Prognosis & Prediction
2275	10.1302/2633-1462.58.BJO-2024-0020.R1	2024	Arthroplasty	Machine Learning (ML); Robotics; Deep Learning (DL)	Prognosis & Prediction
2276	10.5435/JAAOSGlobal-D-21-00093	2021	Arthroplasty	Machine Learning (ML)	Prognosis & Prediction
2277	10.1177/03635465221142280	2023	Unclassified	Deep Learning (DL)	Prognosis & Prediction
2278	10.1016/j.spinee.2025.03.016	2025	Spine Surgery	Natural Language Processing (NLP); Machine Learning (ML); Deep Learning (DL)	Prognosis & Prediction
2279	10.1186/s43019-024-00228-3	2024	Unclassified	Deep Learning (DL)	Unclassified
2280	10.1002/jeo2.12080	2024	Arthroplasty	Machine Learning (ML)	Prognosis & Prediction
2281	10.1007/s00421-020-04575-3	2021	Arthroplasty	Deep Learning (DL)	Unclassified
2282	10.1002/jor.25538	2023	Arthroplasty	Deep Learning (DL)	Segmentation & Measurement
2283	10.1186/s12891-024-07244-0	2024	Unclassified	Deep Learning (DL)	Prognosis & Prediction
2284	10.1007/s11548-020-02221-z	2020	Unclassified	Deep Learning (DL)	Segmentation & Measurement
2285	10.1080/03008207.2022.2048827	2022	Sports Medicine; Arthroplasty	Deep Learning (DL)	Segmentation & Measurement

2286	10.1016/j.artd.2025.101717	2025	Trauma; Arthroplasty	Machine Learning (ML); Deep Learning (DL)	Prognosis & Prediction
2287	10.3390/jfmk10030262	2025	Unclassified	Machine Learning (ML); Deep Learning (DL)	Prognosis & Prediction
2288	10.1002/rcs.2494	2023	Arthroplasty	Deep Learning (DL)	Surgical Planning
2289	10.1097/BSD.0000000000001337	2022	Spine Surgery	Machine Learning (ML); Deep Learning (DL)	Prognosis & Prediction
2290	10.1186/s13018-025-05668-5	2025	Trauma	Machine Learning (ML); Deep Learning (DL)	Prognosis & Prediction
2291	10.1111/os.13507	2022	Trauma	Robotics; Deep Learning (DL)	Unclassified
2292	10.1007/s11548-021-02431-z	2021	Unclassified	Deep Learning (DL)	Prognosis & Prediction
2293	10.1007/s11548-020-02129-8	2020	Sports Medicine; Arthroplasty	Natural Language Processing (NLP); Machine Learning (ML); Deep Learning (DL)	Surgical Planning
2294	10.1007/s11657-022-01063-3	2022	Unclassified	Deep Learning (DL)	Segmentation & Measurement
2295	10.21037/gs-2025-168	2025	Arthroplasty	Machine Learning (ML); Deep Learning (DL)	Prognosis & Prediction
2296	10.1007/s00167-022-07213-x	2023	Trauma; Arthroplasty	Natural Language Processing (NLP); Deep Learning (DL)	Prognosis & Prediction
2297	10.1007/s11548-023-02961-8	2023	Unclassified	Deep Learning (DL)	Unclassified
2298	10.1007/s00264-023-05856-0	2023	Spine Surgery; Trauma; Arthroplasty	Deep Learning (DL)	Prognosis & Prediction
2299	10.1097/TA.0000000000002566	2020	Trauma	Deep Learning (DL)	Prognosis & Prediction
2300	10.1186/s13018-025-05644-z	2025	Trauma; Arthroplasty	Machine Learning (ML); Deep Learning (DL)	Prognosis & Prediction
2301	10.1016/j.jht.2020.07.002	2021	Trauma; Arthroplasty	Unclassified	Unclassified
2302	10.1016/j.jse.2019.05.017	2019	Arthroplasty	Machine Learning (ML); Deep Learning (DL)	Prognosis & Prediction
2303	10.1016/j.jseint.2022.10.004	2023	Unclassified	Machine Learning (ML); Deep Learning (DL)	Prognosis & Prediction
2304	10.1111/os.13469	2022	Unclassified	Machine Learning (ML); Deep Learning (DL)	Prognosis & Prediction
2305	10.1007/s10143-025-03766-w	2025	Spine Surgery	Machine Learning (ML); Deep Learning (DL)	Prognosis & Prediction

2306	10.1007/s00264-022-05644-2	2023	Spine Surgery; Trauma; Arthroplasty	Natural Language Processing (NLP); Machine Learning (ML); Deep Learning (DL)	Prognosis & Prediction
2307	10.26603/001c.124935	2024	Sports Medicine; Trauma	Deep Learning (DL)	Unclassified
2308	10.2196/59422	2025	Trauma	Machine Learning (ML); Deep Learning (DL)	Prognosis & Prediction
2309	10.1016/j.gaitpost.2021.10.015	2022	Arthroplasty	Deep Learning (DL)	Prognosis & Prediction
2310	10.1111/os.14144	2024	Pediatric Orthopaedics; Spine Surgery	Deep Learning (DL)	Surgical Planning
2311	10.23736/S0390-5616.21.05483-7	2022	Spine Surgery	Machine Learning (ML)	Prognosis & Prediction
2312	10.1016/j.wneu.2019.10.063	2020	Spine Surgery	Machine Learning (ML)	Prognosis & Prediction
2313	10.1016/j.arth.2023.06.002	2023	Arthroplasty	Machine Learning (ML); Deep Learning (DL)	Prognosis & Prediction
2314	10.1016/j.jvsv.2024.101943	2024	Trauma	Machine Learning (ML); Deep Learning (DL)	Prognosis & Prediction
2315	10.1007/s00264-022-05631-7	2023	Trauma; Spine Surgery	Machine Learning (ML); Deep Learning (DL)	Prognosis & Prediction
2316	10.3171/2017.7.JNS17878	2018	Trauma	Unclassified	Unclassified
2317	10.1007/s40279-019-01058-0	2019	Sports Medicine; Trauma	Natural Language Processing (NLP); Deep Learning (DL)	Prognosis & Prediction
2318	10.1007/s11657-020-00802-8	2020	Unclassified	Machine Learning (ML)	Prognosis & Prediction
2319	10.1016/j.arthro.2024.01.043	2025	Sports Medicine; Arthroplasty	Deep Learning (DL)	Prognosis & Prediction
2320	10.1007/s00586-021-07020-x	2022	Spine Surgery	Deep Learning (DL)	Prognosis & Prediction
2321	10.1016/j.spinee.2019.11.010	2020	Spine Surgery	Natural Language Processing (NLP); Deep Learning (DL)	Diagnosis & Detection
2322	10.1016/j.jos.2022.01.010	2023	Unclassified	Machine Learning (ML); Deep Learning (DL)	Prognosis & Prediction
2323	10.1097/BRS.0000000000003830	2021	Spine Surgery	Machine Learning (ML); Deep Learning (DL)	Segmentation & Measurement
2324	10.1007/s00256-020-03505-w	2021	Spine Surgery	Machine Learning (ML); Deep Learning (DL)	Prognosis & Prediction
2325	10.3171/2016.10.SPINE16197	2017	Spine Surgery	Unclassified	Prognosis & Prediction

2326	N/A	2020	Arthroplasty	Unclassified	Unclassified
2327	10.1186/s12891-018-1943-8	2018	Unclassified	Natural Language Processing (NLP)	Prognosis & Prediction
2328	10.1117/12.2611912	2022	Spine Surgery	Natural Language Processing (NLP); Deep Learning (DL)	Unclassified
2329	10.1080/2000656X.2020.1858842	2021	Arthroplasty	Unclassified	Unclassified
2330	10.1016/j.gaitpost.2021.03.003	2021	Pediatric Orthopaedics; Arthroplasty	Deep Learning (DL)	Unclassified
2331	10.1002/lsm.23290	2021	Unclassified	Machine Learning (ML); Deep Learning (DL)	Unclassified
2332	10.1177/21925682241227428	2025	Spine Surgery	Deep Learning (DL)	Prognosis & Prediction
2333	10.1186/s13018-024-04680-5	2024	Sports Medicine; Arthroplasty	Unclassified	Segmentation & Measurement
2334	10.1016/j.gaitpost.2022.03.008	2022	Arthroplasty	Deep Learning (DL)	Prognosis & Prediction
2335	10.1002/ksa.70076	2025	Sports Medicine; Arthroplasty	Deep Learning (DL)	Prognosis & Prediction
2336	10.5435/JAAOS-D-23-00063	2023	Arthroplasty	Machine Learning (ML); Robotics; Deep Learning (DL)	Prognosis & Prediction
2337	10.1186/s12891-023-06911-y	2023	Spine Surgery	Natural Language Processing (NLP); Machine Learning (ML); Deep Learning (DL)	Prognosis & Prediction
2338	10.5606/ehc.2020.72163	2020	Trauma	Deep Learning (DL)	Diagnosis & Detection
2339	10.1007/s11657-025-01609-1	2025	Trauma	Deep Learning (DL)	Prognosis & Prediction
2340	10.1097/CORR.0000000000001318	2020	Trauma	Machine Learning (ML); Deep Learning (DL)	Prognosis & Prediction
2341	10.3171/2023.5.SPINE23293	2023	Spine Surgery	Deep Learning (DL)	Segmentation & Measurement
2342	10.1186/s12891-023-06326-9	2023	Trauma; Arthroplasty	Deep Learning (DL)	Prognosis & Prediction
2343	10.1177/23259671211046575	2021	Sports Medicine; Trauma	Machine Learning (ML); Deep Learning (DL)	Prognosis & Prediction
2344	10.3171/2019.5.SPINE1987	2019	Spine Surgery	Machine Learning (ML); Deep Learning (DL)	Prognosis & Prediction
2345	10.1016/j.surg.2025.109376	2025	Arthroplasty	Natural Language Processing (NLP); Machine Learning (ML); Deep Learning (DL)	Prognosis & Prediction

2346	10.1186/s12891-024-08056-y	2024	Unclassified	Deep Learning (DL)	Segmentation & Measurement
2347	10.3171/2022.1.FOCUS21745	2022	Sports Medicine; Trauma; Spine Surgery	Machine Learning (ML); Deep Learning (DL)	Prognosis & Prediction
2348	10.1016/j.spinee.2025.05.003	2025	Trauma; Spine Surgery	Natural Language Processing (NLP); Machine Learning (ML); Deep Learning (DL)	Prognosis & Prediction
2349	10.1007/s13304-025-02296-w	2025	Spine Surgery	Machine Learning (ML); Deep Learning (DL)	Prognosis & Prediction
2350	10.1177/23259671241289096	2024	Sports Medicine; Spine Surgery; Trauma; Arthroplasty	Deep Learning (DL)	Prognosis & Prediction
2351	10.1007/s10143-023-02028-x	2023	Spine Surgery	Machine Learning (ML); Deep Learning (DL)	Prognosis & Prediction
2352	10.1097/CORR.0000000000000997	2020	Trauma	Machine Learning (ML); Deep Learning (DL)	Prognosis & Prediction
2353	10.2106/JBJS.OA.24.00213	2025	Unclassified	Machine Learning (ML); Deep Learning (DL)	Prognosis & Prediction
2354	10.1016/j.spinee.2021.02.007	2021	Trauma; Spine Surgery	Machine Learning (ML)	Prognosis & Prediction
2355	10.1016/j.jse.2020.05.013	2021	Arthroplasty	Machine Learning (ML); Deep Learning (DL)	Prognosis & Prediction
2356	10.1097/JS9.0000000000002806	2025	Spine Surgery	Machine Learning (ML); Deep Learning (DL)	Prognosis & Prediction
2357	10.1016/j.spinee.2020.05.098	2020	Spine Surgery	Natural Language Processing (NLP); Machine Learning (ML)	Prognosis & Prediction
2358	10.2106/JBJS.21.00406	2022	Arthroplasty	Deep Learning (DL)	Prognosis & Prediction
2359	10.1097/CORR.0000000000000433	2018	Unclassified	Machine Learning (ML)	Prognosis & Prediction
2360	10.1097/JS9.0000000000001599	2024	Trauma	Machine Learning (ML); Deep Learning (DL)	Prognosis & Prediction
2361	10.1097/CORR.0000000000001343	2020	Trauma; Arthroplasty	Machine Learning (ML); Deep Learning (DL)	Prognosis & Prediction
2362	10.1016/j.spinee.2024.09.015	2025	Spine Surgery	Natural Language Processing (NLP); Deep Learning (DL)	Prognosis & Prediction
2363	10.1177/15563316251339660	2025	Unclassified	Machine Learning (ML); Deep Learning (DL)	Prognosis & Prediction
2364	10.1016/j.suc.2022.11.004	2023	Spine Surgery	Machine Learning (ML); Deep Learning (DL)	Prognosis & Prediction

2365	10.1302/2046-3758.73.BJR-2017-0147.R1	2018	Arthroplasty	Machine Learning (ML); Deep Learning (DL)	Unclassified
2366	10.1177/18632521251359098	2025	Pediatric Orthopaedics; Spine Surgery	Natural Language Processing (NLP)	Unclassified
2367	10.14245/ns.2347022.511	2023	Trauma; Spine Surgery	Machine Learning (ML); Deep Learning (DL)	Prognosis & Prediction
2368	10.1016/j.wneu.2022.08.109	2022	Spine Surgery	Natural Language Processing (NLP); Machine Learning (ML)	Prognosis & Prediction
2369	10.1016/j.arth.2022.04.003	2023	Arthroplasty	Machine Learning (ML)	Prognosis & Prediction
2370	10.1177/21925682211062831	2023	Unclassified	Natural Language Processing (NLP); Machine Learning (ML)	Unclassified
2371	10.1007/s00256-021-03879-5	2022	Arthroplasty	Natural Language Processing (NLP); Deep Learning (DL)	Diagnosis & Detection
2372	10.1007/s00264-022-05346-9	2022	Trauma; Arthroplasty	Machine Learning (ML); Deep Learning (DL)	Prognosis & Prediction
2373	10.52312/jdrs.2022.57906	2022	Arthroplasty	Machine Learning (ML); Deep Learning (DL)	Unclassified
2374	10.1186/s42836-022-00118-7	2022	Arthroplasty	Machine Learning (ML)	Prognosis & Prediction
2375	10.1007/s00256-021-03824-6	2022	Trauma	Machine Learning (ML); Deep Learning (DL)	Prognosis & Prediction
2376	10.1016/j.knee.2024.08.015	2024	Arthroplasty	Natural Language Processing (NLP)	Prognosis & Prediction
2377	10.1177/21925682211026910	2023	Unclassified	Natural Language Processing (NLP); Deep Learning (DL)	Diagnosis & Detection
2378	10.1007/s00256-024-04684-6	2024	Pediatric Orthopaedics; Trauma; Arthroplasty	Deep Learning (DL)	Diagnosis & Detection
2379	10.1016/j.arth.2019.05.055	2019	Arthroplasty	Machine Learning (ML)	Prognosis & Prediction
2380	N/A	2018	Trauma; Arthroplasty	Machine Learning (ML); Robotics; Deep Learning (DL)	Unclassified
2381	10.5312/wjo.v16.i8.107064	2025	Spine Surgery	Machine Learning (ML); Robotics; Deep Learning (DL)	Prognosis & Prediction
2382	10.1007/s00132-025-04751-3	2025	Sports Medicine; Arthroplasty	Machine Learning (ML); Deep Learning (DL)	Prognosis & Prediction
2383	10.1186/s40634-023-00642-8	2023	Sports Medicine; Spine Surgery; Trauma; Arthroplasty	Natural Language Processing (NLP); Machine Learning (ML); Deep Learning (DL)	Prognosis & Prediction
2384	10.1177/23259671211027543	2021	Sports Medicine; Trauma	Machine Learning (ML)	Prognosis & Prediction

2385	10.2106/JBJS.RVW.24.00075	2024	Spine Surgery; Arthroplasty	Natural Language Processing (NLP); Machine Learning (ML)	Prognosis & Prediction
2386	10.2519/jospt.2024.12151	2024	Unclassified	Natural Language Processing (NLP); Machine Learning (ML)	Unclassified
2387	10.1002/jeo2.12057	2024	Spine Surgery; Arthroplasty	Natural Language Processing (NLP); Machine Learning (ML)	Unclassified
2388	10.1016/j.wneu.2023.12.065	2024	Sports Medicine; Trauma; Spine Surgery	Natural Language Processing (NLP); Machine Learning (ML)	Prognosis & Prediction
2389	10.1016/j.knee.2025.02.032	2025	Sports Medicine	Natural Language Processing (NLP); Machine Learning (ML)	Prognosis & Prediction
2390	10.1177/23259671231215820	2023	Sports Medicine; Trauma; Arthroplasty	Machine Learning (ML); Deep Learning (DL)	Prognosis & Prediction
2391	10.1186/s42836-023-00195-2	2023	Arthroplasty	Natural Language Processing (NLP); Machine Learning (ML); Deep Learning (DL)	Prognosis & Prediction
2392	10.1007/s00256-023-04416-2	2024	Sports Medicine; Trauma	Deep Learning (DL)	Diagnosis & Detection
2393	10.1016/j.spinee.2021.04.019	2021	Trauma; Spine Surgery	Natural Language Processing (NLP); Machine Learning (ML); Deep Learning (DL)	Prognosis & Prediction
2394	10.20517/ais.2024.34	2024	Spine Surgery	Natural Language Processing (NLP); Machine Learning (ML); Robotics; Deep Learning (DL)	Prognosis & Prediction
2395	10.1016/j.wnsx.2024.100301	2024	Spine Surgery	Machine Learning (ML); Deep Learning (DL)	Prognosis & Prediction
2396	10.2106/JBJS.ER.21.00717	2022	Arthroplasty	Machine Learning (ML)	Unclassified
2397	N/A	2019	Trauma; Spine Surgery	Unclassified	Diagnosis & Detection
2398	10.1097/BSD.0000000000001768	2025	Sports Medicine; Spine Surgery; Trauma; Arthroplasty	Natural Language Processing (NLP); Machine Learning (ML)	Unclassified
2399	10.1227/neu.0000000000003360_421	2025	Spine Surgery	Natural Language Processing (NLP)	Unclassified
2400	10.1016/j.artd.2024.101412	2024	Trauma; Arthroplasty	Natural Language Processing (NLP)	Unclassified
2401	10.1016/j.jisako.2024.100323	2024	Trauma; Arthroplasty	Natural Language Processing (NLP); Machine Learning (ML); Deep Learning (DL)	Prognosis & Prediction
2402	10.1007/s12178-025-09997-0	2025	Sports Medicine; Trauma; Pediatric Orthopaedics	Robotics; Deep Learning (DL)	Surgical Planning

2403	10.1007/s00256-021-03909-2	2022	Sports Medicine	Machine Learning (ML); Deep Learning (DL)	Prognosis & Prediction
2404	10.2106/JBJS.21.01548	2022	Arthroplasty	Machine Learning (ML)	Unclassified
2405	10.1007/s00264-024-06401-3	2025	Arthroplasty	Deep Learning (DL)	Prognosis & Prediction
2406	10.3389/fsurg.2025.1702415	2025	Unclassified	Machine Learning (ML); Deep Learning (DL)	Prognosis & Prediction
2407	10.3389/fsurg.2022.957085	2022	Spine Surgery	Natural Language Processing (NLP); Deep Learning (DL)	Unclassified
2408	10.1016/j.arth.2024.04.020	2024	Arthroplasty	Natural Language Processing (NLP); Machine Learning (ML)	Unclassified
2409	10.20517/2347-9264.2024.99	2025	Sports Medicine; Arthroplasty	Natural Language Processing (NLP)	Prognosis & Prediction
2410	10.1007/s00264-025-06531-2	2025	Sports Medicine	Machine Learning (ML); Deep Learning (DL)	Surgical Planning
2411	10.1016/j.jor.2022.08.020	2022	Spine Surgery; Trauma; Arthroplasty	Machine Learning (ML); Robotics; Deep Learning (DL)	Diagnosis & Detection
2412	10.1016/j.wneu.2024.02.101	2024	Pediatric Orthopaedics; Spine Surgery; Trauma; Arthroplasty	Natural Language Processing (NLP)	Diagnosis & Detection
2413	10.1007/s11657-025-01533-4	2025	Trauma	Natural Language Processing (NLP)	Prognosis & Prediction
2414	10.1002/jor.24151	2019	Sports Medicine; Arthroplasty	Deep Learning (DL)	Prognosis & Prediction
2415	10.1016/j.arth.2020.07.076	2021	Arthroplasty	Natural Language Processing (NLP)	Diagnosis & Detection
2416	10.1590/1413-785220243201e280947	2025	Trauma	Natural Language Processing (NLP)	Segmentation & Measurement
2417	10.1530/EOR-23-0083	2023	Unclassified	Machine Learning (ML); Deep Learning (DL)	Unclassified
2418	10.1186/s42836-021-00108-1	2022	Arthroplasty	Natural Language Processing (NLP); Machine Learning (ML); Robotics	Unclassified
2419	10.1177/15589447241298982	2026	Trauma	Natural Language Processing (NLP)	Diagnosis & Detection
2420	10.1007/s00142-024-00673-y	2024	Arthroplasty	Machine Learning (ML); Deep Learning (DL)	Prognosis & Prediction
2421	10.1177/17531934251322723	2025	Trauma	Machine Learning (ML); Deep Learning (DL)	Prognosis & Prediction
2422	10.1002/jeo2.70341	2025	Sports Medicine; Trauma; Arthroplasty	Natural Language Processing (NLP)	Unclassified
2423	10.1002/ksa.70229	2025	Unclassified	Deep Learning (DL)	Surgical Planning

2424	10.1002/jor.70046	2025	Trauma; Arthroplasty	Machine Learning (ML); Deep Learning (DL)	Diagnosis & Detection
2425	10.1007/s00142-024-00671-0	2024	Arthroplasty	Machine Learning (ML); Deep Learning (DL)	Prognosis & Prediction
2426	10.1016/j.spinee.2020.06.014	2021	Spine Surgery	Natural Language Processing (NLP); Machine Learning (ML)	Prognosis & Prediction
2427	N/A	2024	Arthroplasty	Natural Language Processing (NLP); Machine Learning (ML); Deep Learning (DL)	Prognosis & Prediction
2428	10.1177/10225536251407382	2025	Spine Surgery	Natural Language Processing (NLP)	Diagnosis & Detection
2429	10.1016/j.arth.2024.02.005	2024	Arthroplasty	Natural Language Processing (NLP)	Prognosis & Prediction
2430	10.1002/jor.25938	2024	Sports Medicine	Machine Learning (ML); Deep Learning (DL)	Diagnosis & Detection
2431	10.1007/s00256-023-04311-w	2023	Sports Medicine	Deep Learning (DL)	Segmentation & Measurement
2432	10.1227/neu.0000000000001901	2022	Spine Surgery	Machine Learning (ML)	Diagnosis & Detection
2433	10.3171/2022.1.FOCUS21561	2022	Spine Surgery	Natural Language Processing (NLP); Machine Learning (ML); Deep Learning (DL)	Unclassified
2434	10.1016/j.arth.2023.08.063	2024	Arthroplasty	Machine Learning (ML); Deep Learning (DL)	Prognosis & Prediction
2435	10.1002/jor.24943	2022	Sports Medicine; Trauma; Arthroplasty	Deep Learning (DL)	Unclassified
2436	10.3389/fsurg.2023.1284015	2023	Unclassified	Natural Language Processing (NLP)	Prognosis & Prediction
2437	10.14444/8506	2023	Spine Surgery	Machine Learning (ML)	Prognosis & Prediction
2438	10.1007/s00167-019-05792-w	2020	Sports Medicine; Trauma; Arthroplasty	Machine Learning (ML)	Prognosis & Prediction
2439	10.1016/j.jham.2025.100217	2025	Trauma	Natural Language Processing (NLP)	Prognosis & Prediction
2440	10.1051/sicotj/2024044	2024	Arthroplasty	Machine Learning (ML); Robotics; Deep Learning (DL)	Prognosis & Prediction
2441	10.1016/j.arth.2023.08.070	2023	Arthroplasty	Deep Learning (DL)	Unclassified
2442	10.1302/0301-620X.104B8	2022	Arthroplasty	Deep Learning (DL)	Unclassified
2443	10.1097/CORR.0000000000003234	2024	Sports Medicine; Trauma; Arthroplasty	Natural Language Processing (NLP)	Prognosis & Prediction

2444	10.1007/s00256-025-05001-5	2026	Unclassified	Deep Learning (DL)	Unclassified
2445	10.1007/978-3-030-87202-1_52	2021	Unclassified	Unclassified	Surgical Planning
2446	10.1016/j.wneu.2022.09.090	2022	Spine Surgery	Machine Learning (ML)	Prognosis & Prediction
2447	10.1177/21925682221138053	2024	Unclassified	Machine Learning (ML)	Prognosis & Prediction
2448	10.1530/EOR-23-0180	2024	Sports Medicine	Machine Learning (ML)	Prognosis & Prediction
2449	10.31616/asj.2024.0048	2024	Spine Surgery	Machine Learning (ML)	Prognosis & Prediction
2450	10.1186/s12891-016-1013-z	2016	Unclassified	Unclassified	Prognosis & Prediction
2451	10.1177/15533506221106070	2023	Unclassified	Unclassified	Unclassified
2452	10.1016/j.otsr.2025.104390	2025	Unclassified	Machine Learning (ML)	Unclassified
2453	10.1111/os.13617	2023	Trauma	Machine Learning (ML)	Prognosis & Prediction
2454	10.1186/s12891-024-08045-1	2024	Trauma; Spine Surgery	Machine Learning (ML)	Prognosis & Prediction
2455	10.1007/s11548-020-02214-y	2020	Arthroplasty	Deep Learning (DL)	Diagnosis & Detection
2456	10.1007/s00586-024-08604-z	2025	Spine Surgery	Machine Learning (ML)	Prognosis & Prediction
2457	10.1302/2046-3758.1410.BJR-2025-0036.R1	2025	Trauma; Arthroplasty	Unclassified	Surgical Planning
2458	10.1016/j.wneu.2025.124100	2025	Trauma	Machine Learning (ML)	Prognosis & Prediction
2459	10.1177/18632521221133812	2022	Pediatric Orthopaedics	Natural Language Processing (NLP); Machine Learning (ML)	Prognosis & Prediction
2460	10.1016/j.injury.2020.11.031	2021	Trauma	Unclassified	Prognosis & Prediction
2461	10.1097/CORR.0000000000003455	2025	Unclassified	Natural Language Processing (NLP)	Unclassified
2462	10.1016/j.jot.2022.03.001	2022	Arthroplasty	Robotics	Unclassified
2463	10.1177/23259671241270355	2024	Sports Medicine; Arthroplasty	Natural Language Processing (NLP); Machine Learning (ML)	Prognosis & Prediction
2464	10.1002/jeo2.12039	2024	Unclassified	Natural Language Processing (NLP)	Prognosis & Prediction
2465	10.1186/s12891-024-07714-5	2024	Spine Surgery	Machine Learning (ML)	Prognosis & Prediction

2466	10.1002/jor.24763	2020	Trauma	Machine Learning (ML)	Prognosis & Prediction
2467	10.1016/j.amjsurg.2021.03.058	2021	Unclassified	Machine Learning (ML)	Prognosis & Prediction
2468	10.2106/JBJS.15.00975	2016	Trauma; Spine Surgery	Unclassified	Prognosis & Prediction
2469	10.1016/j.jos.2023.05.004	2024	Unclassified	Deep Learning (DL)	Prognosis & Prediction
2470	10.1159/000525862	2022	Pediatric Orthopaedics	Machine Learning (ML)	Prognosis & Prediction
2471	10.5312/wjo.v15.i11.1023	2024	Pediatric Orthopaedics	Unclassified	Unclassified
2472	10.17816/2311-2905-17468	2024	Trauma	Natural Language Processing (NLP)	Unclassified
2473	10.1007/s43465-023-00845-2	2023	Unclassified	Unclassified	Prognosis & Prediction
2474	10.1117/12.2611783	2022	Arthroplasty	Deep Learning (DL)	Unclassified
2475	10.1007/s00256-025-05004-2	2025	Arthroplasty	Unclassified	Prognosis & Prediction
2476	10.1016/j.arthro.2025.02.038	2025	Unclassified	Unclassified	Prognosis & Prediction
2477	10.21037/jss-23-111	2024	Spine Surgery	Machine Learning (ML)	Prognosis & Prediction
2478	10.1177/23259671211050613	2021	Unclassified	Machine Learning (ML)	Prognosis & Prediction
2479	10.1302/0301-620X.107B7.BJJ-2025-0167.R1$2.00	2025	Pediatric Orthopaedics; Arthroplasty	Machine Learning (ML)	Prognosis & Prediction
2480	10.1007/s11548-019-01937-x	2019	Unclassified	Deep Learning (DL)	Prognosis & Prediction
2481	10.1519/JSC.0000000000004758	2024	Sports Medicine; Trauma	Machine Learning (ML)	Prognosis & Prediction
2482	10.1016/j.clinbiomech.2024.106265	2024	Trauma	Robotics; Deep Learning (DL)	Prognosis & Prediction
2483	10.1097/SCS.0000000000011573	2025	Unclassified	Unclassified	Prognosis & Prediction
2484	10.3390/std14030033	2025	Unclassified	Unclassified	Surgical Planning
2485	10.21037/jovs-22-40	2023	Unclassified	Unclassified	Prognosis & Prediction
2486	10.1016/j.surg.2022.08.037	2023	Arthroplasty	Machine Learning (ML)	Prognosis & Prediction
2487	10.1007/s11548-022-02650-y	2022	Unclassified	Robotics	Prognosis & Prediction
2488	10.1007/s00464-024-11485-4	2025	Spine Surgery	Deep Learning (DL)	Prognosis & Prediction

2489	10.1007/s00132-025-04749-x	2025	Trauma	Natural Language Processing (NLP)	Unclassified
2490	10.1016/j.jshs.2024.01.002	2024	Sports Medicine; Trauma; Arthroplasty	Unclassified	Diagnosis & Detection
2491	10.1109/STAR62027.2024.10636005	2024	Sports Medicine; Trauma	Machine Learning (ML)	Prognosis & Prediction
2492	10.1097/SCS.0000000000011959	2025	Sports Medicine	Machine Learning (ML)	Unclassified
2493	10.3389/fsurg.2024.1487716	2024	Trauma; Arthroplasty	Unclassified	Segmentation & Measurement
2494	10.1007/s00264-022-05676-8	2023	Sports Medicine	Unclassified	Unclassified
2495	10.1016/j.xnsj.2024.100529	2024	Trauma; Spine Surgery	Natural Language Processing (NLP)	Prognosis & Prediction
2496	10.1007/s11548-021-02488-w	2021	Unclassified	Deep Learning (DL)	Diagnosis & Detection
2497	10.1186/s13018-025-06332-8	2025	Arthroplasty	Machine Learning (ML)	Surgical Planning
2498	10.1016/j.jot.2025.05.011	2025	Sports Medicine; Trauma	Machine Learning (ML)	Prognosis & Prediction
2499	10.1177/21925682251341263	2026	Spine Surgery	Machine Learning (ML)	Prognosis & Prediction
2500	10.1007/s00586-025-09146-8	2025	Sports Medicine; Spine Surgery	Unclassified	Diagnosis & Detection
2501	10.1111/os.13431	2022	Spine Surgery	Deep Learning (DL)	Surgical Planning
2502	10.14444/8567	2024	Trauma; Spine Surgery	Machine Learning (ML)	Prognosis & Prediction
2503	10.1016/j.spinee.2025.03.018	2025	Unclassified	Natural Language Processing (NLP); Machine Learning (ML)	Prognosis & Prediction
2504	10.1186/s13018-025-06263-4	2025	Unclassified	Machine Learning (ML)	Unclassified
2505	10.1227/neu.0000000000002911	2024	Spine Surgery	Machine Learning (ML)	Prognosis & Prediction
2506	10.1186/s13018-025-06286-x	2025	Spine Surgery	Machine Learning (ML)	Prognosis & Prediction
2507	10.1016/j.jcms.2025.07.007	2025	Arthroplasty	Unclassified	Surgical Planning
2508	10.1007/s00586-023-07963-3	2024	Trauma; Spine Surgery	Machine Learning (ML)	Prognosis & Prediction
2509	10.3171/2023.11.SPINE23979	2023	Trauma; Spine Surgery	Unclassified	Prognosis & Prediction

2510	10.1007/s11548-023-02957-4	2023	Unclassified	Natural Language Processing (NLP); Robotics; Deep Learning (DL)	Surgical Planning
2511	10.1016/j.spinee.2021.11.015	2022	Spine Surgery	Natural Language Processing (NLP); Machine Learning (ML)	Prognosis & Prediction
2512	10.1016/j.wnEu.2024.04.025	2024	Spine Surgery	Deep Learning (DL)	Diagnosis & Detection
2513	10.1002/jor.26089	2025	Sports Medicine	Machine Learning (ML)	Prognosis & Prediction
2514	10.1186/s13037-023-00356-x	2023	Unclassified	Machine Learning (ML)	Prognosis & Prediction
2515	10.1007/s00104-019-01066-w	2020	Arthroplasty	Robotics	Unclassified
2516	10.3171/2024.5.SPINE231081	2024	Spine Surgery	Machine Learning (ML)	Prognosis & Prediction
2517	10.1007/s00586-023-07641-4	2023	Spine Surgery	Deep Learning (DL)	Diagnosis & Detection
2518	10.1097/SCS.0000000000010294	2025	Pediatric Orthopaedics	Unclassified	Prognosis & Prediction
2519	10.1097/JS9.0000000000002944	2025	Trauma	Unclassified	Prognosis & Prediction
2520	10.3171/2023.6.SPINE23348	2023	Spine Surgery	Machine Learning (ML)	Prognosis & Prediction
2521	10.1186/s12891-024-07589-6	2024	Unclassified	Machine Learning (ML)	Unclassified
2522	10.3389/fspor.2023.1298003	2024	Arthroplasty	Unclassified	Unclassified
2523	10.1007/s11548-019-01919-z	2019	Arthroplasty	Natural Language Processing (NLP); Machine Learning (ML)	Prognosis & Prediction
2524	10.1097/JS9.0000000000000892	2024	Trauma; Arthroplasty	Machine Learning (ML)	Prognosis & Prediction
2525	10.1097/BSD.0000000000001764	2025	Sports Medicine; Trauma; Spine Surgery	Unclassified	Unclassified
2526	N/A	2018	Spine Surgery	Natural Language Processing (NLP); Deep Learning (DL)	Segmentation & Measurement
2527	10.1007/s43390-024-00990-0	2025	Sports Medicine; Spine Surgery	Deep Learning (DL)	Prognosis & Prediction
2528	10.1002/rcs.1639	2016	Unclassified	Robotics	Segmentation & Measurement
2529	10.1002/jso.70001	2025	Arthroplasty	Machine Learning (ML)	Prognosis & Prediction

2530	10.1016/j.wneu.2022.04.008	2022	Sports Medicine; Spine Surgery	Machine Learning (ML)	Segmentation & Measurement
2531	10.1007/s00586-020-06356-0	2020	Spine Surgery	Machine Learning (ML)	Prognosis & Prediction
2532	10.3390/sports12120317	2024	Sports Medicine; Arthroplasty	Unclassified	Unclassified
2533	10.3389/fspor.2022.939980	2022	Sports Medicine; Arthroplasty	Deep Learning (DL)	Unclassified
2534	10.1016/j.jhsa.2024.10.006	2025	Arthroplasty	Machine Learning (ML)	Segmentation & Measurement
2535	10.1016/j.spinee.2023.01.002	2023	Spine Surgery	Natural Language Processing (NLP); Machine Learning (ML)	Prognosis & Prediction
2536	10.1016/j.wneu.2025.124464	2025	Spine Surgery	Machine Learning (ML)	Prognosis & Prediction
2537	10.1007/s00256-024-04818-w	2025	Trauma; Spine Surgery	Unclassified	Diagnosis & Detection
2538	10.3171/2025.4.FOCUS24614	2025	Spine Surgery	Machine Learning (ML)	Prognosis & Prediction
2539	10.1186/s12891-024-07297-1	2024	Trauma; Spine Surgery	Deep Learning (DL)	Prognosis & Prediction
2540	10.1007/s11701-025-02954-6	2025	Spine Surgery	Robotics	Surgical Planning
2541	10.1007/s12283-025-00507-6	2025	Arthroplasty	Deep Learning (DL)	Segmentation & Measurement
2542	10.1186/s13018-023-03858-7	2023	Trauma	Unclassified	Prognosis & Prediction
2543	10.1097/BRS.0000000000005267	2025	Spine Surgery	Machine Learning (ML)	Prognosis & Prediction
2544	10.2106/JBJS.24.00386	2025	Pediatric Orthopaedics; Spine Surgery	Machine Learning (ML)	Prognosis & Prediction
2545	10.1145/3606038.3616171	2023	Sports Medicine; Arthroplasty	Unclassified	Unclassified
2546	10.1097/GOX.0000000000007134	2025	Pediatric Orthopaedics	Machine Learning (ML)	Unclassified
2547	10.1016/j.wneu.2023.04.029	2023	Unclassified	Deep Learning (DL)	Prognosis & Prediction
2548	10.1097/BRS.0000000000005227	2025	Sports Medicine; Spine Surgery	Deep Learning (DL)	Prognosis & Prediction
2549	10.1016/j.avsg.2025.01.010	2025	Sports Medicine	Unclassified	Prognosis & Prediction
2550	10.1186/s40798-021-00405-8	2022	Sports Medicine; Trauma	Natural Language Processing (NLP); Machine Learning (ML)	Prognosis & Prediction

2551	10.1080/02640414.2025.2576412	2025	Arthroplasty	Unclassified	Diagnosis & Detection
2552	10.1007/s00256-022-04098-2	2023	Unclassified	Machine Learning (ML)	Prognosis & Prediction
2553	10.1177/03635465221107388	2022	Sports Medicine; Trauma	Unclassified	Prognosis & Prediction
2554	N/A	2024	Sports Medicine; Trauma	Deep Learning (DL)	Prognosis & Prediction
2555	10.1002/jor.25952	2024	Sports Medicine	Machine Learning (ML)	Prognosis & Prediction
2556	10.1007/s11548-022-02757-2	2023	Unclassified	Machine Learning (ML); Deep Learning (DL)	Prognosis & Prediction
2557	10.3390/biomechanics5040088	2025	Arthroplasty	Unclassified	Prognosis & Prediction
2558	10.14444/8163	2021	Spine Surgery	Unclassified	Prognosis & Prediction
2559	10.1177/03635465231167854	2023	Sports Medicine; Arthroplasty	Deep Learning (DL)	Prognosis & Prediction
2560	10.1007/s00256-025-05038-6	2025	Unclassified	Deep Learning (DL)	Prognosis & Prediction
2561	10.1016/j.otsr.2018.05.018	2019	Trauma; Arthroplasty	Machine Learning (ML)	Diagnosis & Detection
2562	10.1007/s11548-022-02714-z	2022	Sports Medicine; Arthroplasty	Deep Learning (DL)	Prognosis & Prediction
2563	10.1111/os.13894	2024	Spine Surgery	Deep Learning (DL)	Diagnosis & Detection
2564	10.1080/14763141.2022.2112750	2022	Arthroplasty	Machine Learning (ML)	Prognosis & Prediction
2565	10.1016/j.spinee.2025.03.022	2025	Spine Surgery	Natural Language Processing (NLP); Machine Learning (ML)	Prognosis & Prediction
2566	10.1016/j.arthro.2022.07.008	2023	Unclassified	Machine Learning (ML)	Prognosis & Prediction
2567	10.1097/TA.0000000000004568	2025	Trauma	Machine Learning (ML)	Prognosis & Prediction
2568	10.1007/s00113-025-01557-y	2025	Trauma	Unclassified	Unclassified
2569	10.2106/JBJS.24.00821	2025	Sports Medicine; Trauma	Machine Learning (ML)	Prognosis & Prediction
2570	10.1007/s43390-025-01150-8	2025	Pediatric Orthopaedics	Robotics	Surgical Planning
2571	10.1186/s40798-025-00953-3	2025	Sports Medicine; Trauma; Arthroplasty	Deep Learning (DL)	Unclassified

2572	10.3171/2024.9.SPINE24639	2024	Spine Surgery	Unclassified	Prognosis & Prediction
2573	10.1016/j.arthro.2022.01.041	2022	Sports Medicine	Machine Learning (ML)	Prognosis & Prediction
2574	10.3171/2020.6.SPINE20707	2021	Spine Surgery	Machine Learning (ML)	Prognosis & Prediction
2575	10.1002/jor.24930	2021	Sports Medicine; Spine Surgery	Natural Language Processing (NLP); Machine Learning (ML)	Unclassified
2576	10.1177/01945998211044982	2022	Unclassified	Robotics; Deep Learning (DL)	Prognosis & Prediction
2577	10.5435/JAAOS-D-20-00894	2021	Spine Surgery	Machine Learning (ML)	Prognosis & Prediction
2578	10.1186/s13018-024-05353-z	2025	Spine Surgery	Machine Learning (ML)	Prognosis & Prediction
2579	10.1016/j.wneu.2018.11.088	2019	Spine Surgery	Unclassified	Prognosis & Prediction
2580	10.2106/JBJS.19.00661	2019	Unclassified	Natural Language Processing (NLP); Machine Learning (ML)	Prognosis & Prediction
2581	10.1080/14763141.2024.2369913	2025	Trauma; Arthroplasty	Machine Learning (ML)	Unclassified
2582	10.1177/0363546520950743	2020	Unclassified	Machine Learning (ML)	Prognosis & Prediction
2583	10.1016/j.gaitpost.2019.03.008	2019	Unclassified	Unclassified	Unclassified
2584	10.1016/j.gaitpost.2021.01.021	2021	Trauma	Machine Learning (ML)	Prognosis & Prediction
2585	10.1097/CORR.0000000000003030	2024	Spine Surgery	Unclassified	Prognosis & Prediction
2586	10.1177/15589447241233762	2025	Trauma	Machine Learning (ML)	Prognosis & Prediction
2587	10.1016/j.jhsa.2020.10.021	2021	Sports Medicine	Natural Language Processing (NLP); Machine Learning (ML)	Prognosis & Prediction
2588	10.1007/s11548-022-02627-x	2022	Unclassified	Deep Learning (DL)	Unclassified
2589	10.1007/s00381-023-06041-9	2023	Pediatric Orthopaedics; Spine Surgery	Unclassified	Prognosis & Prediction
2590	10.2519/jospt.2025.12707	2025	Spine Surgery	Unclassified	Prognosis & Prediction
2591	10.1007/s11701-025-03048-z	2025	Spine Surgery	Natural Language Processing (NLP); Robotics	Unclassified

2592	10.1177/23259671241297145	2024	Sports Medicine; Pediatric Orthopaedics; Arthroplasty	Machine Learning (ML)	Prognosis & Prediction
2593	10.21037/hbsn-21-424	2023	Unclassified	Machine Learning (ML)	Prognosis & Prediction
2594	10.1007/s00113-022-01169-w	2022	Trauma	Unclassified	Unclassified
2595	10.1007/s00256-023-04447-9	2024	Arthroplasty	Deep Learning (DL)	Prognosis & Prediction
2596	10.20517/2347-9264.2024.166	2025	Unclassified	Unclassified	Prognosis & Prediction
2597	10.1016/j.fas.2024.05.010	2024	Arthroplasty	Unclassified	Diagnosis & Detection
2598	10.1016/j.jse.2024.08.034	2025	Trauma	Natural Language Processing (NLP); Machine Learning (ML)	Prognosis & Prediction
2599	10.3389/fsurg.2024.1344263	2024	Arthroplasty	Machine Learning (ML)	Prognosis & Prediction
2600	10.1097/SLA.0000000000004132	2020	Trauma	Unclassified	Prognosis & Prediction
2601	10.1007/s00256-024-04723-2	2024	Unclassified	Deep Learning (DL)	Diagnosis & Detection
2602	10.1007/s11701-025-02555-3	2025	Pediatric Orthopaedics	Robotics	Prognosis & Prediction
2603	10.1016/j.spinee.2023.03.015	2023	Spine Surgery	Natural Language Processing (NLP); Machine Learning (ML)	Prognosis & Prediction
2604	10.1007/s00256-024-04669-5	2024	Arthroplasty	Deep Learning (DL)	Prognosis & Prediction
2605	10.1186/s12891-022-05598-x	2022	Spine Surgery	Machine Learning (ML)	Prognosis & Prediction
2606	10.3171/2023.5.SPINE2367	2023	Spine Surgery	Machine Learning (ML)	Prognosis & Prediction
2607	10.1007/s00132-025-04712-w	2025	Trauma	Machine Learning (ML)	Unclassified
2608	10.1016/j.gaitpost.2024.05.029	2024	Arthroplasty	Deep Learning (DL)	Prognosis & Prediction
2609	10.1007/s11548-024-03180-5	2024	Arthroplasty	Robotics; Deep Learning (DL)	Surgical Planning
2610	10.1186/s13037-025-00456-w	2025	Unclassified	Natural Language Processing (NLP)	Prognosis & Prediction
2611	10.1016/j.ijscr.2025.111666	2025	Pediatric Orthopaedics; Spine Surgery	Unclassified	Diagnosis & Detection
2612	10.1016/j.clinbiomech.2025.106502	2025	Spine Surgery	Unclassified	Segmentation & Measurement

2613	10.1007/s00256-023-04466-6	2024	Sports Medicine; Arthroplasty	Deep Learning (DL)	Diagnosis & Detection
2614	10.3389/fsurg.2025.1546367	2025	Spine Surgery	Natural Language Processing (NLP)	Prognosis & Prediction
2615	10.1007/s43465-022-00712-6	2022	Unclassified	Natural Language Processing (NLP)	Prognosis & Prediction
2616	10.1097/BRS.0000000000004531	2023	Spine Surgery	Machine Learning (ML)	Prognosis & Prediction
2617	10.1097/SCS.0000000000005587	2020	Arthroplasty	Natural Language Processing (NLP); Robotics	Surgical Planning
2618	10.3389/fspor.2022.1094163	2023	Sports Medicine; Spine Surgery	Machine Learning (ML)	Prognosis & Prediction
2619	10.1186/s13018-023-04490-1	2024	Spine Surgery	Machine Learning (ML)	Prognosis & Prediction
2620	10.1016/j.jot.2022.09.015	2022	Unclassified	Unclassified	Unclassified
2621	10.21037/gs-2025-35	2025	Arthroplasty	Machine Learning (ML)	Prognosis & Prediction
2622	10.3171/2022.11.SPINE22763	2023	Trauma; Spine Surgery	Machine Learning (ML)	Prognosis & Prediction
2623	10.1080/02640414.2024.2308419	2023	Unclassified	Machine Learning (ML)	Unclassified
2624	10.1002/jor.24829	2021	Spine Surgery	Machine Learning (ML)	Prognosis & Prediction
2625	10.1002/jsp2.1178	2021	Spine Surgery	Machine Learning (ML)	Prognosis & Prediction
2626	10.1302/0301-620X.105B6.BJJ-2022-0998	2023	Unclassified	Machine Learning (ML)	Prognosis & Prediction
2627	10.1002/jor.25036	2022	Unclassified	Machine Learning (ML)	Prognosis & Prediction
2628	10.3389/fsurg.2022.1010420	2022	Spine Surgery	Machine Learning (ML)	Prognosis & Prediction
2629	10.1097/SCS.0000000000006943	2021	Unclassified	Machine Learning (ML)	Prognosis & Prediction
2630	10.1186/s13018-024-04569-3	2024	Sports Medicine; Trauma; Arthroplasty	Machine Learning (ML)	Diagnosis & Detection
2631	10.1016/j.gaitpost.2024.01.021	2024	Arthroplasty	Machine Learning (ML)	Segmentation & Measurement
2632	10.1016/j.jht.2025.05.019	2025	Arthroplasty	Natural Language Processing (NLP)	Diagnosis & Detection
2633	10.2106/JBJS.OA.25.00143	2025	Unclassified	Unclassified	Prognosis & Prediction
2634	10.1186/s13037-024-00403-1	2024	Trauma; Spine Surgery	Machine Learning (ML)	Prognosis & Prediction

2635	10.1007/978-3-030-87202-1_14	2021	Pediatric Orthopaedics; Arthroplasty	Deep Learning (DL)	Segmentation & Measurement
2636	10.1016/j.wneu.2020.05.131	2020	Sports Medicine; Trauma; Spine Surgery	Robotics	Unclassified
2637	10.1177/03635465221091847	2022	Unclassified	Machine Learning (ML)	Prognosis & Prediction
2638	10.3171/2020.3.SPINE20208	2020	Spine Surgery	Robotics	Surgical Planning
2639	10.1016/j.ijom.2022.01.017	2022	Arthroplasty	Robotics	Surgical Planning
2640	10.1016/j.apmr.2024.08.026	2025	Unclassified	Unclassified	Prognosis & Prediction
2641	10.1007/s11678-025-00863-6	2025	Arthroplasty	Deep Learning (DL)	Diagnosis & Detection
2642	10.1097/SCS.0000000000008868	2023	Unclassified	Machine Learning (ML)	Prognosis & Prediction
2643	10.1123/jab.2022-0041	2022	Spine Surgery	Machine Learning (ML)	Diagnosis & Detection
2644	10.3389/fsurg.2025.1555749	2025	Trauma	Unclassified	Prognosis & Prediction
2645	10.5435/JAAOS-D-25-00189	2025	Unclassified	Machine Learning (ML)	Prognosis & Prediction
2646	10.1007/s00381-023-05883-7	2023	Unclassified	Machine Learning (ML)	Diagnosis & Detection
2647	10.1097/BRS.0000000000003922	2021	Sports Medicine; Trauma; Pediatric Orthopaedics; Spine Surgery	Machine Learning (ML)	Prognosis & Prediction
2648	10.1016/j.spinee.2023.08.018	2023	Spine Surgery	Natural Language Processing (NLP); Machine Learning (ML)	Prognosis & Prediction
2649	10.1097/BRS.0000000000002515	2018	Unclassified	Machine Learning (ML)	Prognosis & Prediction
2650	10.1097/CORR.0000000000002698	2023	Unclassified	Unclassified	Prognosis & Prediction
2651	10.1007/s00132-023-04400-7	2023	Unclassified	Unclassified	Prognosis & Prediction
2652	10.1186/s12891-018-2350-x	2019	Spine Surgery	Machine Learning (ML)	Unclassified
2653	10.1097/TA.0000000000003846	2023	Trauma	Machine Learning (ML)	Prognosis & Prediction
2654	10.1016/j.spinee.2022.06.008	2022	Spine Surgery	Natural Language Processing (NLP); Machine Learning (ML)	Prognosis & Prediction
2655	10.1136/bmjsem-2022-001372	2022	Sports Medicine; Trauma	Machine Learning (ML)	Prognosis & Prediction

2656	10.1123/jmpb.2022-0018	2023	Pediatric Orthopaedics; Arthroplasty	Machine Learning (ML)	Unclassified
2657	10.1177/21925682231203491	2024	Sports Medicine; Trauma; Spine Surgery	Machine Learning (ML)	Prognosis & Prediction
2658	10.1227/neu.0000000000002733	2024	Spine Surgery	Machine Learning (ML)	Prognosis & Prediction
2659	10.1016/j.surg.2025.109263	2025	Arthroplasty	Natural Language Processing (NLP)	Prognosis & Prediction
2660	10.1016/j.spinee.2025.01.014	2025	Spine Surgery	Natural Language Processing (NLP); Machine Learning (ML)	Prognosis & Prediction
2661	10.1080/02640414.2020.1856462	2021	Sports Medicine; Arthroplasty	Machine Learning (ML)	Prognosis & Prediction
2662	10.25259/JMSR_54_2025	2025	Unclassified	Unclassified	Unclassified
2663	10.1016/j.gassur.2025.101997	2025	Arthroplasty	Natural Language Processing (NLP); Machine Learning (ML)	Prognosis & Prediction
2664	10.1097/SLA.0000000000006171	2025	Spine Surgery; Arthroplasty	Machine Learning (ML)	Prognosis & Prediction
2665	10.1186/s13018-025-05494-9	2025	Arthroplasty	Machine Learning (ML)	Prognosis & Prediction
2666	10.1016/j.gaitpost.2023.03.010	2024	Trauma; Arthroplasty	Machine Learning (ML)	Prognosis & Prediction
2667	10.1080/17461391.2021.1902570	2022	Arthroplasty	Machine Learning (ML)	Prognosis & Prediction
2668	10.1136/bmjsem-2023-001566	2023	Trauma	Machine Learning (ML)	Prognosis & Prediction
2669	10.1007/s00256-018-2919-3	2018	Spine Surgery	Natural Language Processing (NLP)	Prognosis & Prediction
2670	10.1016/j.gaitpost.2019.07.190	2019	Unclassified	Machine Learning (ML)	Unclassified
2671	10.1016/j.spinee.2023.07.021	2024	Trauma; Spine Surgery	Natural Language Processing (NLP); Machine Learning (ML)	Prognosis & Prediction
2672	10.1111/sms.14249	2023	Trauma	Machine Learning (ML)	Unclassified
2673	10.1007/s42978-021-00108-2	2021	Arthroplasty	Natural Language Processing (NLP); Robotics	Diagnosis & Detection
2674	10.1016/j.spinee.2024.09.033	2025	Trauma; Spine Surgery	Natural Language Processing (NLP); Machine Learning (ML)	Prognosis & Prediction
2675	10.1007/s11548-021-02550-7	2022	Spine Surgery	Machine Learning (ML)	Unclassified
2676	10.1007/s11548-020-02144-9	2020	Arthroplasty	Deep Learning (DL)	Diagnosis & Detection

2677	10.1097/GOX.0000000000002081	2019	Pediatric Orthopaedics	Unclassified	Unclassified
2678	N/A	2025	Unclassified	Unclassified	Prognosis & Prediction
2679	10.1177/15533506241289481	2024	Spine Surgery	Natural Language Processing (NLP)	Prognosis & Prediction
2680	10.1186/s13018-023-03933-z	2023	Sports Medicine	Machine Learning (ML)	Prognosis & Prediction
2681	10.1097/CORR.0000000000001969	2022	Unclassified	Unclassified	Prognosis & Prediction
2682	10.1016/j.spinee.2020.10.026	2021	Spine Surgery	Unclassified	Prognosis & Prediction
2683	10.1007/s11548-020-02229-5	2020	Unclassified	Unclassified	Unclassified
2684	10.1097/GOX.0000000000006413	2025	Spine Surgery	Machine Learning (ML)	Prognosis & Prediction
2685	10.1177/23259671221121410	2022	Sports Medicine; Trauma	Machine Learning (ML)	Prognosis & Prediction
2686	10.1177/0363546519892905	2020	Arthroplasty	Machine Learning (ML)	Prognosis & Prediction
2687	10.1093/ptj/pzad121	2024	Sports Medicine; Arthroplasty	Unclassified	Segmentation & Measurement
2688	10.1177/10711007211020676	2022	Arthroplasty	Unclassified	Prognosis & Prediction
2689	10.1186/s12891-023-06281-5	2023	Trauma; Spine Surgery	Natural Language Processing (NLP); Deep Learning (DL)	Prognosis & Prediction
2690	10.1186/s12891-025-09229-z	2025	Unclassified	Machine Learning (ML)	Prognosis & Prediction
2691	10.1136/jnis-2024-022275	2025	Unclassified	Machine Learning (ML)	Prognosis & Prediction
2692	10.1097/CORR.0000000000001251	2020	Sports Medicine	Machine Learning (ML)	Prognosis & Prediction
2693	10.2106/JBJS.OA.25.00148	2025	Trauma	Natural Language Processing (NLP)	Diagnosis & Detection
2694	10.1302/2633-1462.64.BJO-2025-0002	2025	Arthroplasty	Unclassified	Prognosis & Prediction
2695	10.1097/CORR.0000000000002276	2022	Arthroplasty	Machine Learning (ML)	Prognosis & Prediction
2696	10.1097/BOT.0000000000003036	2025	Trauma	Natural Language Processing (NLP)	Diagnosis & Detection
2697	10.1007/s00586-024-08132-w	2024	Spine Surgery	Machine Learning (ML)	Prognosis & Prediction

2698	10.1016/j.spinee.2020.05.003	2020	Unclassified	Natural Language Processing (NLP); Machine Learning (ML)	Prognosis & Prediction
2699	10.1080/17461391.2021.1955015	2022	Arthroplasty	Machine Learning (ML)	Prognosis & Prediction
2700	10.1016/j.surg.2018.04.010	2018	Unclassified	Unclassified	Prognosis & Prediction
2701	10.1016/j.wneu.2023.11.010	2024	Spine Surgery	Machine Learning (ML)	Prognosis & Prediction
2702	10.1177/21925682251331050	2025	Spine Surgery	Machine Learning (ML)	Prognosis & Prediction
2703	10.1002/ksa.12460	2025	Sports Medicine; Trauma	Machine Learning (ML)	Prognosis & Prediction
2704	10.1080/17453674.2021.1910448	2021	Unclassified	Machine Learning (ML)	Prognosis & Prediction
2705	10.1007/978-3-030-87202-1_39	2021	Arthroplasty	Deep Learning (DL)	Segmentation & Measurement
2706	10.1007/s11548-024-03126-x	2024	Spine Surgery; Arthroplasty	Deep Learning (DL)	Diagnosis & Detection
2707	10.1177/03635465211015179	2021	Sports Medicine; Trauma	Machine Learning (ML)	Prognosis & Prediction
2708	10.1097/FS9.0000000000000213	2025	Spine Surgery	Deep Learning (DL)	Prognosis & Prediction
2709	10.1016/j.ijscr.2025.112048	2025	Trauma	Unclassified	Prognosis & Prediction
2710	10.1097/BOT.0000000000000991	2017	Trauma	Unclassified	Prognosis & Prediction
2711	10.1097/CORR.0000000000001081	2020	Trauma	Machine Learning (ML)	Prognosis & Prediction
2712	10.1007/s11548-021-02333-0	2021	Spine Surgery	Deep Learning (DL)	Diagnosis & Detection
2713	10.1016/j.arthro.2025.02.018	2025	Unclassified	Unclassified	Prognosis & Prediction
2714	10.1186/s13102-025-01140-3	2025	Unclassified	Machine Learning (ML)	Prognosis & Prediction
2715	10.1016/j.jht.2025.01.006	2025	Unclassified	Natural Language Processing (NLP)	Unclassified
2716	10.1016/j.arth.2023.01.018	2023	Arthroplasty	Machine Learning (ML)	Prognosis & Prediction
2717	10.2106/JBJS.OA.24.00081	2024	Trauma	Natural Language Processing (NLP)	Diagnosis & Detection
2718	10.1016/j.wneu.2024.01.072	2024	Spine Surgery	Natural Language Processing (NLP); Machine Learning (ML)	Prognosis & Prediction
2719	10.1007/s43465-025-01461-y	2025	Unclassified	Machine Learning (ML); Robotics	Prognosis & Prediction

2720	10.1007/s00167-023-07580-z	2023	Sports Medicine; Arthroplasty	Machine Learning (ML)	Prognosis & Prediction
2721	10.1186/s13018-024-05004-3	2024	Trauma; Spine Surgery	Machine Learning (ML)	Prognosis & Prediction
2722	10.1016/j.gaitpost.2018.11.029	2019	Arthroplasty	Unclassified	Unclassified
2723	10.1177/1754337119874276	2020	Sports Medicine; Arthroplasty	Unclassified	Diagnosis & Detection
2724	10.1007/s00256-021-03928-z	2022	Trauma	Deep Learning (DL)	Segmentation & Measurement
2725	10.1002/jor.25865	2024	Sports Medicine	Deep Learning (DL)	Segmentation & Measurement
2726	10.1177/10556656221087071	2023	Pediatric Orthopaedics	Machine Learning (ML)	Prognosis & Prediction
2727	10.1007/s00113-024-01517-y	2025	Trauma	Natural Language Processing (NLP)	Unclassified
2728	10.1177/21925682251388420	2025	Spine Surgery	Machine Learning (ML)	Prognosis & Prediction
2729	10.14245/ns.2143244.622	2022	Spine Surgery	Machine Learning (ML)	Prognosis & Prediction
2730	10.1186/s12891-024-07903-2	2024	Spine Surgery	Machine Learning (ML)	Prognosis & Prediction
2731	10.1080/00222895.2023.2218821	2023	Arthroplasty	Machine Learning (ML)	Unclassified
2732	10.1186/s12891-023-06286-0	2023	Trauma	Unclassified	Prognosis & Prediction
2733	10.1016/j.xnsj.2025.100597	2025	Spine Surgery; Arthroplasty	Machine Learning (ML)	Prognosis & Prediction
2734	10.2106/JBJS.OA.24.00065	2025	Trauma	Machine Learning (ML)	Prognosis & Prediction
2735	10.1186/s12891-020-03679-3	2020	Spine Surgery	Deep Learning (DL)	Segmentation & Measurement
2736	10.1080/02640414.2025.2518359	2025	Sports Medicine; Trauma; Arthroplasty	Deep Learning (DL)	Prognosis & Prediction
2737	10.1186/s12891-025-08697-7	2025	Sports Medicine	Deep Learning (DL)	Surgical Planning
2738	10.3171/2022.5.SPINE22504	2022	Spine Surgery	Unclassified	Prognosis & Prediction
2739	10.1016/j.suronc.2022.101782	2022	Unclassified	Unclassified	Prognosis & Prediction
2740	10.1007/s11548-024-03123-0	2024	Unclassified	Deep Learning (DL)	Prognosis & Prediction

2741	10.1016/j.apmr.2024.12.015	2025	Spine Surgery	Machine Learning (ML)	Prognosis & Prediction
2742	10.1016/j.spinee.2025.05.025	2025	Trauma; Spine Surgery	Natural Language Processing (NLP); Machine Learning (ML)	Prognosis & Prediction
2743	10.1016/j.jos.2021.12.002	2022	Unclassified	Unclassified	Unclassified
2744	10.1016/j.bjps.2025.09.017	2025	Sports Medicine	Natural Language Processing (NLP); Robotics; Deep Learning (DL)	Prognosis & Prediction
2745	10.1007/s11548-024-03089-z	2024	Unclassified	Deep Learning (DL)	Diagnosis & Detection
2746	10.1007/s00113-020-00866-8	2021	Trauma	Unclassified	Diagnosis & Detection
2747	10.1097/BSD.0000000000001451	2023	Trauma; Spine Surgery	Machine Learning (ML)	Prognosis & Prediction
2748	10.3390/surgeries6010023	2025	Spine Surgery	Unclassified	Unclassified
2749	10.3390/jfmk10010084	2025	Arthroplasty	Machine Learning (ML)	Diagnosis & Detection
2750	10.1519/JSC.0000000000005181	2025	Arthroplasty	Machine Learning (ML)	Prognosis & Prediction
2751	10.1016/j.xnsj.2024.100313	2024	Spine Surgery	Machine Learning (ML)	Unclassified
2752	10.1007/s11548-018-1871-y	2019	Spine Surgery	Natural Language Processing (NLP); Machine Learning (ML); Robotics	Unclassified
2753	10.1002/rcs.1947	2018	Arthroplasty	Deep Learning (DL)	Unclassified
2754	10.1177/21925682241303103	2025	Spine Surgery	Machine Learning (ML)	Prognosis & Prediction
2755	10.1177/23259671251371238	2025	Arthroplasty	Machine Learning (ML)	Prognosis & Prediction
2756	10.1097/BOT.0000000000002290	2022	Trauma	Machine Learning (ML)	Prognosis & Prediction
2757	10.1097/CORR.0000000000002718	2024	Unclassified	Unclassified	Prognosis & Prediction
2758	10.2147/ORR.S518036	2025	Unclassified	Machine Learning (ML)	Prognosis & Prediction
2759	10.1007/s11548-016-1490-4	2017	Unclassified	Unclassified	Segmentation & Measurement
2760	10.1002/ksa.12190	2024	Sports Medicine; Arthroplasty	Machine Learning (ML)	Diagnosis & Detection
2761	10.1007/s00276-022-02933-8	2022	Unclassified	Deep Learning (DL)	Segmentation & Measurement
2762	10.1097/CORR.0000000000002294	2022	Trauma	Machine Learning (ML)	Prognosis & Prediction

2763	10.1177/15533506241265274	2024	Unclassified	Robotics	Prognosis & Prediction
2764	10.1302/2633-1462.411.BJO-2023-0060.R1	2023	Pediatric Orthopaedics	Unclassified	Prognosis & Prediction
2765	10.1016/j.jor.2022.06.001	2022	Trauma	Robotics	Prognosis & Prediction
2766	10.1097/SLA.0000000000005250	2023	Unclassified	Machine Learning (ML)	Prognosis & Prediction
2767	10.1097/TA.0000000000003709	2023	Pediatric Orthopaedics; Trauma; Arthroplasty	Machine Learning (ML)	Prognosis & Prediction
2768	10.3389/fsurg.2022.1049685	2022	Unclassified	Unclassified	Unclassified
2769	10.14444/7012	2020	Spine Surgery	Unclassified	Prognosis & Prediction
2770	10.1097/CORR.0000000000002706	2024	Trauma	Unclassified	Prognosis & Prediction
2771	10.1007/s00264-022-05626-4	2023	Pediatric Orthopaedics	Unclassified	Unclassified
2772	10.1016/j.surg.2019.06.005	2019	Arthroplasty	Unclassified	Prognosis & Prediction
2773	10.5312/wjo.v13.i8.693	2022	Unclassified	Natural Language Processing (NLP)	Unclassified
2774	10.14444/8153	2021	Unclassified	Unclassified	Prognosis & Prediction
2775	10.1111/os.14148	2024	Trauma; Spine Surgery	Machine Learning (ML)	Prognosis & Prediction
2776	10.1093/bjsopen/zrad141	2024	Unclassified	Unclassified	Prognosis & Prediction
2777	10.3389/fsurg.2021.776945	2022	Unclassified	Natural Language Processing (NLP); Robotics	Unclassified
2778	10.1097/CORR.0000000000000748	2019	Unclassified	Machine Learning (ML)	Prognosis & Prediction
2779	10.1007/s43465-023-00971-x	2023	Unclassified	Robotics	Unclassified
2780	10.1186/s12891-024-07548-1	2024	Pediatric Orthopaedics	Deep Learning (DL)	Prognosis & Prediction
2781	10.1155/aort/8238794	2025	Sports Medicine; Trauma	Unclassified	Prognosis & Prediction
2782	10.1007/s11548-023-02858-6	2023	Unclassified	Deep Learning (DL)	Diagnosis & Detection
2783	10.3389/fsurg.2025.1605726	2025	Arthroplasty	Machine Learning (ML)	Prognosis & Prediction
2784	10.1007/s11548-025-03348-7	2025	Trauma	Deep Learning (DL)	Surgical Planning

2785	10.1016/j.hcl.2024.12.004	2025	Sports Medicine; Trauma; Spine Surgery	Unclassified	Segmentation & Measurement
2786	10.1016/j.avsg.2025.08.001	2026	Unclassified	Unclassified	Prognosis & Prediction
2787	10.5114/biolsport.2024.144014	2024	Sports Medicine	Machine Learning (ML)	Prognosis & Prediction
2788	10.1016/j.fas.2024.03.007	2024	Trauma	Machine Learning (ML)	Prognosis & Prediction
2789	10.1097/CORR.0000000000003185	2024	Trauma	Unclassified	Prognosis & Prediction
2790	10.1097/CORR.0000000000003173	2024	Unclassified	Machine Learning (ML)	Surgical Planning
2791	10.1136/bjsports-2022-105534	2023	Unclassified	Machine Learning (ML)	Prognosis & Prediction
2792	10.1007/s44254-025-00093-7	2025	Unclassified	Machine Learning (ML)	Prognosis & Prediction
2793	10.1097/JS9.0000000000002118	2024	Trauma	Machine Learning (ML)	Prognosis & Prediction
2794	10.1016/j.jot.2025.07.005	2025	Trauma	Robotics	Diagnosis & Detection
2795	10.3171/2024.4.SPINE24107	2024	Trauma; Spine Surgery	Machine Learning (ML)	Prognosis & Prediction
2796	10.1016/j.jse.2023.04.021	2023	Arthroplasty	Machine Learning (ML)	Unclassified
2797	10.1177/0309364619883197	2019	Trauma	Unclassified	Prognosis & Prediction
2798	10.1097/DCR.0000000000003034	2024	Arthroplasty	Machine Learning (ML)	Prognosis & Prediction
2799	10.21037/jmai-24-480	2025	Spine Surgery	Natural Language Processing (NLP)	Unclassified
2800	10.1136/bmjopen-2025-104612	2025	Trauma	Natural Language Processing (NLP); Robotics	Prognosis & Prediction
2801	10.1007/s40520-025-02931-x	2025	Arthroplasty	Machine Learning (ML)	Prognosis & Prediction
2802	10.1186/s12911-025-02927-7	2025	Arthroplasty	Natural Language Processing (NLP); Machine Learning (ML)	Prognosis & Prediction
2803	10.1007/s13755-025-00343-9	2025	Spine Surgery	Machine Learning (ML)	Prognosis & Prediction
2804	10.1007/s00068-025-02793-y	2025	Trauma	Deep Learning (DL)	Diagnosis & Detection
2805	10.1007/s00068-025-02779-w	2025	Trauma; Pediatric Orthopaedics	Deep Learning (DL)	Diagnosis & Detection

2806	10.1186/s12885-025-13631-0	2025	Unclassified	Machine Learning (ML); Deep Learning (DL)	Prognosis & Prediction
2807	10.1186/s13244-025-01911-z	2025	Sports Medicine; Trauma	Deep Learning (DL)	Diagnosis & Detection
2808	10.1007/s12306-025-00894-w	2025	Unclassified	Natural Language Processing (NLP)	Unclassified
2809	10.1186/s12889-025-21658-y	2025	Unclassified	Machine Learning (ML)	Prognosis & Prediction
2810	10.1186/s41747-025-00553-6	2025	Sports Medicine; Pediatric Orthopaedics; Spine Surgery	Deep Learning (DL)	Segmentation & Measurement
2811	10.1186/s13244-025-01902-0	2025	Spine Surgery	Deep Learning (DL)	Unclassified
2812	10.1002/ar.25633	2025	Unclassified	Natural Language Processing (NLP); Deep Learning (DL)	Prognosis & Prediction
2813	10.1007/s00068-024-02731-4	2025	Trauma	Deep Learning (DL)	Prognosis & Prediction
2814	10.3390/jcm14248689	2025	Sports Medicine; Arthroplasty	Machine Learning (ML); Deep Learning (DL)	Prognosis & Prediction
2815	10.1186/s13075-025-03709-2	2025	Arthroplasty	Machine Learning (ML)	Prognosis & Prediction
2816	10.1186/s12877-025-06648-4	2025	Trauma	Machine Learning (ML)	Prognosis & Prediction
2817	10.1186/s40658-025-00812-w	2025	Unclassified	Natural Language Processing (NLP); Machine Learning (ML)	Prognosis & Prediction
2818	10.1093/sexmed/qfaf101	2025	Trauma	Machine Learning (ML); Deep Learning (DL)	Prognosis & Prediction
2819	10.1016/j.asmr.2025.101269	2025	Arthroplasty	Machine Learning (ML); Deep Learning (DL)	Prognosis & Prediction
2820	10.1016/j.jcot.2025.103250	2025	Arthroplasty	Machine Learning (ML); Deep Learning (DL)	Prognosis & Prediction
2821	10.1002/mp.70178	2025	Arthroplasty	Deep Learning (DL)	Diagnosis & Detection
2822	10.5414/CN111750	2025	Spine Surgery	Unclassified	Unclassified
2823	10.1007/s10916-025-02306-9	2025	Pediatric Orthopaedics	Deep Learning (DL)	Prognosis & Prediction
2824	10.1186/s12883-025-04510-z	2025	Arthroplasty	Machine Learning (ML)	Prognosis & Prediction
2825	10.1002/acm2.70336	2025	Spine Surgery	Deep Learning (DL)	Prognosis & Prediction
2826	10.1093/gerona/glaf200	2025	Trauma	Machine Learning (ML)	Prognosis & Prediction

2827	10.1186/s12909-025-08162-y	2025	Unclassified	Machine Learning (ML)	Prognosis & Prediction
2828	10.1016/j.mcpdig.2025.100299	2025	Spine Surgery	Deep Learning (DL)	Prognosis & Prediction
2829	10.1016/j.jocn.2025.111648	2025	Spine Surgery	Natural Language Processing (NLP)	Unclassified
2830	10.1186/s12984-025-01778-7	2025	Sports Medicine; Arthroplasty	Natural Language Processing (NLP); Deep Learning (DL)	Segmentation & Measurement
2831	10.1186/s44158-025-00291-6	2025	Trauma	Machine Learning (ML)	Prognosis & Prediction
2832	10.1007/s13755-025-00393-z	2025	Trauma	Deep Learning (DL)	Prognosis & Prediction
2833	10.1016/j.jbo.2025.100718	2025	Unclassified	Natural Language Processing (NLP); Deep Learning (DL)	Prognosis & Prediction
2834	10.1016/j.jcot.2025.103240	2025	Spine Surgery	Natural Language Processing (NLP); Robotics	Prognosis & Prediction
2835	10.1038/s41514-025-00271-8	2025	Trauma; Spine Surgery	Deep Learning (DL)	Prognosis & Prediction
2836	10.1186/s12874-025-02654-7	2025	Pediatric Orthopaedics	Machine Learning (ML); Deep Learning (DL)	Prognosis & Prediction
2837	10.1186/s12911-025-03202-5	2025	Trauma	Natural Language Processing (NLP)	Diagnosis & Detection
2838	10.1016/j.jbo.2025.100716	2025	Unclassified	Machine Learning (ML)	Prognosis & Prediction
2839	10.1186/s12911-025-03177-3	2025	Unclassified	Machine Learning (ML)	Prognosis & Prediction
2840	10.1016/j.acra.2025.09.014	2025	Pediatric Orthopaedics; Arthroplasty	Machine Learning (ML)	Prognosis & Prediction
2841	10.1186/s43055-025-01580-4	2025	Trauma	Unclassified	Prognosis & Prediction
2842	10.1186/s12883-025-04389-w	2025	Trauma	Machine Learning (ML); Deep Learning (DL)	Prognosis & Prediction
2843	10.1186/s43055-025-01575-1	2025	Trauma; Arthroplasty	Unclassified	Prognosis & Prediction
2844	10.1007/s11096-025-01989-x	2025	Pediatric Orthopaedics	Machine Learning (ML)	Prognosis & Prediction
2845	10.1016/j.ejro.2025.100679	2025	Unclassified	Deep Learning (DL)	Diagnosis & Detection
2846	10.1186/s40360-025-00990-4	2025	Unclassified	Machine Learning (ML)	Prognosis & Prediction
2847	10.1186/s13244-025-02046-x	2025	Trauma	Deep Learning (DL)	Diagnosis & Detection

2848	10.1186/s12880-025-01669-2	2025	Trauma; Pediatric Orthopaedics	Natural Language Processing (NLP); Machine Learning (ML); Deep Learning (DL)	Diagnosis & Detection
2849	10.1186/s40779-025-00633-z	2025	Unclassified	Machine Learning (ML); Deep Learning (DL)	Diagnosis & Detection
2850	10.1007/s00270-025-04126-3	2025	Sports Medicine; Trauma; Arthroplasty	Machine Learning (ML)	Prognosis & Prediction
2851	10.1007/s13246-025-01621-2	2025	Unclassified	Deep Learning (DL)	Prognosis & Prediction
2852	10.1186/s12877-025-06213-z	2025	Trauma	Machine Learning (ML)	Prognosis & Prediction
2853	10.1007/s00068-025-02931-6	2025	Trauma	Deep Learning (DL)	Diagnosis & Detection
2854	10.1038/s41746-025-01878-7	2025	Arthroplasty	Machine Learning (ML)	Prognosis & Prediction
2855	10.1111/ijlh.14527	2025	Pediatric Orthopaedics	Deep Learning (DL)	Diagnosis & Detection
2856	10.1186/s13023-025-03875-1	2025	Sports Medicine; Pediatric Orthopaedics	Unclassified	Diagnosis & Detection
2857	10.1186/s12911-025-03110-8	2025	Unclassified	Machine Learning (ML); Deep Learning (DL)	Prognosis & Prediction
2858	10.1186/s13244-025-02034-1	2025	Trauma	Deep Learning (DL)	Diagnosis & Detection
2859	10.1016/j.bspc.2025.108302	2025	Trauma	Natural Language Processing (NLP); Machine Learning (ML); Deep Learning (DL)	Diagnosis & Detection
2860	10.1016/j.bspc.2025.108257	2025	Trauma; Pediatric Orthopaedics	Deep Learning (DL)	Prognosis & Prediction
2861	10.1186/s41747-025-00572-3	2025	Trauma	Deep Learning (DL)	Prognosis & Prediction
2862	10.1007/s00330-025-11742-7	2025	Spine Surgery	Deep Learning (DL)	Diagnosis & Detection
2863	10.1007/s10278-025-01440-7	2025	Arthroplasty	Natural Language Processing (NLP); Deep Learning (DL)	Diagnosis & Detection
2864	10.1007/s10916-025-02207-x	2025	Unclassified	Natural Language Processing (NLP)	Prognosis & Prediction
2865	10.1186/s12984-025-01654-4	2025	Unclassified	Natural Language Processing (NLP); Machine Learning (ML)	Prognosis & Prediction
2866	10.1186/s12911-025-03024-5	2025	Arthroplasty	Natural Language Processing (NLP)	Unclassified

2867	10.1186/s13075-025-03576-x	2025	Unclassified	Machine Learning (ML)	Prognosis & Prediction
2868	10.1186/s13244-025-01976-w	2025	Sports Medicine	Natural Language Processing (NLP); Machine Learning (ML)	Prognosis & Prediction
2869	10.1186/s13244-025-01967-x	2025	Arthroplasty	Machine Learning (ML); Deep Learning (DL)	Prognosis & Prediction
2870	10.1007/s13721-025-00518-0	2025	Trauma; Pediatric Orthopaedics	Machine Learning (ML); Deep Learning (DL)	Diagnosis & Detection
2871	10.1186/s12877-025-05920-x	2025	Trauma	Machine Learning (ML)	Prognosis & Prediction
2872	10.1007/s12308-025-00625-x	2025	Unclassified	Deep Learning (DL)	Diagnosis & Detection
2873	10.1186/s41747-025-00574-1	2025	Unclassified	Machine Learning (ML); Deep Learning (DL)	Diagnosis & Detection
2874	10.1186/s12880-025-01641-0	2025	Trauma; Spine Surgery	Deep Learning (DL)	Diagnosis & Detection
2875	10.1038/s41698-025-00855-3	2025	Unclassified	Deep Learning (DL)	Unclassified
2876	10.1097/MD.0000000000046015	2025	Trauma; Arthroplasty	Machine Learning (ML); Robotics	Prognosis & Prediction
2877	10.21037/qims-2025-942	2025	Unclassified	Deep Learning (DL)	Diagnosis & Detection
2878	10.3390/diagnostics15222882	2025	Trauma; Pediatric Orthopaedics	Natural Language Processing (NLP)	Prognosis & Prediction
2879	10.3390/jcm14217657	2025	Arthroplasty	Machine Learning (ML); Deep Learning (DL)	Prognosis & Prediction
2880	10.1177/20552076251390556	2025	Unclassified	Machine Learning (ML); Deep Learning (DL)	Diagnosis & Detection
2881	10.4274/dir.2024.242790	2025	Sports Medicine; Pediatric Orthopaedics	Deep Learning (DL)	Segmentation & Measurement
2882	10.1002/ima.70239	2025	Unclassified	Machine Learning (ML); Deep Learning (DL)	Prognosis & Prediction
2883	10.1016/j.jcot.2025.103212	2025	Arthroplasty	Natural Language Processing (NLP); Machine Learning (ML); Deep Learning (DL)	Prognosis & Prediction
2884	10.1002/ejp.70140	2025	Trauma	Machine Learning (ML)	Prognosis & Prediction
2885	10.1016/j.xrrt.2025.07.025	2025	Trauma; Arthroplasty	Machine Learning (ML); Deep Learning (DL)	Diagnosis & Detection

2886	10.1007/s10067-025-07640-4	2025	Unclassified	Natural Language Processing (NLP)	Unclassified
2887	10.1007/s00198-025-07634-7	2025	Trauma; Spine Surgery	Deep Learning (DL)	Prognosis & Prediction
2888	10.1016/j.jhsg.2025.100822	2025	Trauma	Natural Language Processing (NLP)	Unclassified
2889	10.1016/j.xrrt.2025.06.020	2025	Sports Medicine; Arthroplasty	Natural Language Processing (NLP)	Unclassified
2890	10.1016/j.acra.2025.07.060	2025	Spine Surgery	Deep Learning (DL)	Diagnosis & Detection
2891	10.1016/j.jcot.2025.103177	2025	Arthroplasty	Natural Language Processing (NLP); Machine Learning (ML)	Prognosis & Prediction
2892	10.1016/j.acra.2025.07.058	2025	Pediatric Orthopaedics	Natural Language Processing (NLP)	Unclassified
2893	10.1016/j.mri.2025.110505	2025	Spine Surgery	Deep Learning (DL)	Diagnosis & Detection
2894	10.1007/s00198-025-07604-z	2025	Trauma; Spine Surgery	Machine Learning (ML); Deep Learning (DL)	Diagnosis & Detection
2895	10.1016/j.bone.2025.117592	2025	Unclassified	Machine Learning (ML); Robotics	Prognosis & Prediction
2896	10.1016/j.bspc.2025.108001	2025	Sports Medicine; Arthroplasty	Deep Learning (DL)	Prognosis & Prediction
2897	10.1007/s00330-025-11644-8	2025	Sports Medicine; Trauma; Spine Surgery	Deep Learning (DL)	Diagnosis & Detection
2898	10.1097/MD.0000000000045406	2025	Arthroplasty	Machine Learning (ML)	Prognosis & Prediction
2899	10.1016/j.crmeth.2025.101179	2025	Spine Surgery	Deep Learning (DL)	Diagnosis & Detection
2900	10.1097/MD.0000000000045230	2025	Arthroplasty	Machine Learning (ML)	Prognosis & Prediction
2901	N/A	2025	Unclassified	Machine Learning (ML); Deep Learning (DL)	Prognosis & Prediction
2902	10.1097/MD.0000000000044876	2025	Trauma; Spine Surgery	Robotics; Deep Learning (DL)	Prognosis & Prediction
2903	10.4274/jtss.galenos.2025.74436	2025	Spine Surgery	Natural Language Processing (NLP); Machine Learning (ML)	Unclassified
2904	10.1016/j.nec.2025.04.013	2025	Trauma; Spine Surgery	Machine Learning (ML); Deep Learning (DL)	Segmentation & Measurement
2905	10.3390/jcm14207216	2025	Pediatric Orthopaedics; Spine Surgery	Deep Learning (DL)	Prognosis & Prediction
2906	10.3390/curroncol32100533	2025	Unclassified	Machine Learning (ML); Deep Learning (DL)	Prognosis & Prediction
2907	10.3390/genes16101125	2025	Arthroplasty	Machine Learning (ML)	Prognosis & Prediction
2908	10.14744/tjtes.2025.98680	2025	Trauma; Pediatric Orthopaedics	Natural Language Processing (NLP)	Prognosis & Prediction

2909	10.3390/healthcare13192507	2025	Trauma; Arthroplasty	Machine Learning (ML)	Prognosis & Prediction
2910	10.3390/jcm14196886	2025	Arthroplasty	Machine Learning (ML)	Diagnosis & Detection
2911	10.1055/s-0045-1809941	2025	Unclassified	Natural Language Processing (NLP); Machine Learning (ML); Deep Learning (DL)	Diagnosis & Detection
2912	10.1002/mp.70010	2025	Arthroplasty	Machine Learning (ML); Deep Learning (DL)	Diagnosis & Detection
2913	10.1007/s11547-025-02028-5	2025	Trauma; Pediatric Orthopaedics	Unclassified	Diagnosis & Detection
2914	10.1016/j.compbiomed.2025.110961	2025	Spine Surgery	Deep Learning (DL)	Prognosis & Prediction
2915	10.1016/j.jcot.2025.103161	2025	Unclassified	Natural Language Processing (NLP); Machine Learning (ML)	Prognosis & Prediction
2916	10.1007/s11547-025-02051-6	2025	Trauma	Deep Learning (DL)	Prognosis & Prediction
2917	10.1007/s11547-025-02058-z	2025	Trauma; Spine Surgery	Deep Learning (DL)	Prognosis & Prediction
2918	10.1016/j.leukres.2025.107923	2025	Unclassified	Natural Language Processing (NLP); Deep Learning (DL)	Diagnosis & Detection
2919	10.1016/j.ejrad.2025.112310	2025	Sports Medicine; Spine Surgery	Deep Learning (DL)	Surgical Planning
2920	10.1016/j.jcot.2025.103116	2025	Unclassified	Natural Language Processing (NLP)	Unclassified
2921	10.1016/j.ejrad.2025.112309	2025	Trauma; Pediatric Orthopaedics	Deep Learning (DL)	Prognosis & Prediction
2922	10.1016/j.acra.2025.06.023	2025	Trauma; Spine Surgery	Machine Learning (ML)	Prognosis & Prediction
2923	10.1016/j.jcot.2025.103100	2025	Unclassified	Natural Language Processing (NLP); Machine Learning (ML); Robotics	Prognosis & Prediction
2924	10.1016/j.jcot.2025.103101	2025	Sports Medicine	Machine Learning (ML); Deep Learning (DL)	Prognosis & Prediction
2925	10.1177/14604086251345001	2025	Trauma	Natural Language Processing (NLP)	Unclassified
2926	10.1007/s10278-025-01412-x	2025	Trauma	Machine Learning (ML); Deep Learning (DL)	Prognosis & Prediction
2927	10.1007/s00330-025-11554-9	2025	Trauma; Pediatric Orthopaedics	Deep Learning (DL)	Prognosis & Prediction
2928	10.3348/jksr.2025.0013	2025	Sports Medicine; Trauma; Spine Surgery	Deep Learning (DL)	Diagnosis & Detection
2929	10.3348/jksr.2025.0015	2025	Spine Surgery; Arthroplasty	Deep Learning (DL)	Unclassified
2930	10.11152/mu-4495	2025	Arthroplasty	Deep Learning (DL)	Diagnosis & Detection

2931	10.1097/MD.0000000000043716	2025	Trauma; Arthroplasty	Natural Language Processing (NLP)	Diagnosis & Detection
2932	10.1007/s10742-025-00340-1	2025	Trauma	Machine Learning (ML); Deep Learning (DL)	Prognosis & Prediction
2933	10.1016/j.jormas.2024.102124	2025	Arthroplasty	Deep Learning (DL)	Diagnosis & Detection
2934	10.5624/isd.20250077	2025	Arthroplasty	Machine Learning (ML); Deep Learning (DL)	Prognosis & Prediction
2935	10.3390/diagnostics15182386	2025	Trauma	Deep Learning (DL)	Prognosis & Prediction
2936	10.3390/diagnostics15172162	2025	Sports Medicine; Spine Surgery	Deep Learning (DL)	Surgical Planning
2937	10.3174/ajnr.A8715	2025	Trauma; Spine Surgery	Deep Learning (DL)	Diagnosis & Detection
2938	10.1093/dmfr/twaf028	2025	Trauma	Machine Learning (ML); Deep Learning (DL)	Diagnosis & Detection
2939	10.1093/dmfr/twaf031	2025	Trauma	Deep Learning (DL)	Diagnosis & Detection
2940	10.1093/dmfr/twaf044	2025	Unclassified	Deep Learning (DL)	Prognosis & Prediction
2941	10.1016/j.bonr.2025.101870	2025	Trauma	Machine Learning (ML)	Prognosis & Prediction
2942	10.1002/msc.70177	2025	Unclassified	Natural Language Processing (NLP)	Unclassified
2943	10.1016/j.jbiomech.2025.112881	2025	Trauma; Arthroplasty	Machine Learning (ML); Deep Learning (DL)	Prognosis & Prediction
2944	10.1111/bjh.20265	2025	Unclassified	Machine Learning (ML)	Prognosis & Prediction
2945	10.1016/j.jss.2025.06.040	2025	Trauma	Machine Learning (ML)	Prognosis & Prediction
2946	10.1016/j.jbi.2025.104870	2025	Arthroplasty	Machine Learning (ML); Deep Learning (DL)	Prognosis & Prediction
2947	10.1016/j.acra.2025.06.021	2025	Unclassified	Deep Learning (DL)	Prognosis & Prediction
2948	10.1016/j.compbiomed.2025.110732	2025	Trauma; Pediatric Orthopaedics	Deep Learning (DL)	Diagnosis & Detection
2949	10.1016/j.compbiomed.2025.110683	2025	Trauma	Machine Learning (ML); Deep Learning (DL)	Prognosis & Prediction
2950	10.1016/j.acra.2025.05.059	2025	Unclassified	Machine Learning (ML)	Prognosis & Prediction
2951	10.1016/j.ejrad.2025.112230	2025	Trauma; Spine Surgery	Unclassified	Prognosis & Prediction
2952	10.1016/j.forsciint.2025.112531	2025	Unclassified	Deep Learning (DL)	Prognosis & Prediction
2953	10.1016/j.ejrad.2025.112244	2025	Trauma; Spine Surgery	Deep Learning (DL)	Prognosis & Prediction

2954	10.1016/j.cmpb.2025.108878	2025	Arthroplasty	Natural Language Processing (NLP)	Unclassified
2955	10.1097/RTI.0000000000000833	2025	Trauma	Deep Learning (DL)	Diagnosis & Detection
2956	10.1007/s00414-025-03497-z	2025	Pediatric Orthopaedics	Deep Learning (DL)	Diagnosis & Detection
2957	10.1016/j.mri.2025.110396	2025	Arthroplasty	Deep Learning (DL)	Unclassified
2958	10.1016/j.mri.2025.110405	2025	Unclassified	Deep Learning (DL)	Prognosis & Prediction
2959	10.1007/s00414-025-03469-3	2025	Pediatric Orthopaedics	Machine Learning (ML); Deep Learning (DL)	Diagnosis & Detection
2960	10.1016/j.recot.2025.02.007	2025	Trauma; Spine Surgery	Natural Language Processing (NLP); Machine Learning (ML); Deep Learning (DL)	Prognosis & Prediction
2961	10.1053/j.sart.2025.02.003	2025	Arthroplasty	Natural Language Processing (NLP)	Unclassified
2962	10.1007/s00330-025-11449-9	2025	Pediatric Orthopaedics; Trauma; Arthroplasty	Machine Learning (ML); Deep Learning (DL)	Prognosis & Prediction
2963	10.1097/MD.0000000000044136	2025	Arthroplasty	Machine Learning (ML); Deep Learning (DL)	Prognosis & Prediction
2964	10.1097/MD.0000000000043849	2025	Arthroplasty	Machine Learning (ML)	Diagnosis & Detection
2965	10.3803/EnM.2025.2518	2025	Trauma	Deep Learning (DL)	Prognosis & Prediction
2966	10.3390/biomedicines13081855	2025	Arthroplasty	Machine Learning (ML)	Prognosis & Prediction
2967	10.3390/jcm14165876	2025	Unclassified	Natural Language Processing (NLP)	Prognosis & Prediction
2968	10.1371/journal.pmed.1004665	2025	Sports Medicine; Arthroplasty	Machine Learning (ML); Deep Learning (DL)	Prognosis & Prediction
2969	10.1002/lio2.70188	2025	Unclassified	Machine Learning (ML); Robotics	Prognosis & Prediction
2970	10.1111/jep.70238	2025	Sports Medicine; Trauma	Natural Language Processing (NLP)	Prognosis & Prediction
2971	10.14744/tjtes.2025.75806	2025	Trauma	Machine Learning (ML); Deep Learning (DL)	Diagnosis & Detection
2972	10.1177/10519815251329261	2025	Unclassified	Machine Learning (ML)	Prognosis & Prediction
2973	10.1016/j.asmr.2025.101210	2025	Sports Medicine	Natural Language Processing (NLP)	Prognosis & Prediction
2974	10.1016/j.jval.2025.04.2165	2025	Trauma	Unclassified	Prognosis & Prediction
2975	10.1007/s11845-025-03971-y	2025	Arthroplasty	Natural Language Processing (NLP)	Unclassified

2976	10.1007/s10278-024-01359-5	2025	Trauma; Spine Surgery	Machine Learning (ML)	Diagnosis & Detection
2977	10.1007/s10278-024-01323-3	2025	Unclassified	Unclassified	Segmentation & Measurement
2978	10.1007/s10278-024-01317-1	2025	Unclassified	Deep Learning (DL)	Surgical Planning
2979	10.1016/j.jflm.2025.102912	2025	Pediatric Orthopaedics; Arthroplasty	Machine Learning (ML)	Diagnosis & Detection
2980	10.1016/j.compbiomed.2025.110372	2025	Spine Surgery	Machine Learning (ML); Deep Learning (DL)	Prognosis & Prediction
2981	10.1016/j.joms.2025.04.010	2025	Trauma; Pediatric Orthopaedics	Deep Learning (DL)	Prognosis & Prediction
2982	10.1002/pbc.31797	2025	Pediatric Orthopaedics	Deep Learning (DL)	Prognosis & Prediction
2983	10.1097/MD.0000000000043257	2025	Unclassified	Deep Learning (DL)	Surgical Planning
2984	10.1016/j.crmeth.2025.101107	2025	Arthroplasty	Deep Learning (DL)	Diagnosis & Detection
2985	10.1007/s11604-025-01754-0	2025	Trauma	Unclassified	Diagnosis & Detection
2986	10.1097/BPO.0000000000002923	2025	Trauma; Pediatric Orthopaedics	Natural Language Processing (NLP)	Unclassified
2987	10.1007/s00330-025-11373-y	2025	Pediatric Orthopaedics	Deep Learning (DL)	Prognosis & Prediction
2988	10.1097/RCT.0000000000001726	2025	Trauma; Spine Surgery	Deep Learning (DL)	Prognosis & Prediction
2989	10.1016/j.bspc.2025.107661	2025	Unclassified	Natural Language Processing (NLP); Deep Learning (DL)	Unclassified
2990	10.1109/EMBC58623.2025.11252672	2025	Arthroplasty	Machine Learning (ML); Deep Learning (DL)	Prognosis & Prediction
2991	10.1109/EMBC58623.2025.11254563	2025	Arthroplasty	Machine Learning (ML)	Prognosis & Prediction
2992	10.1109/EMBC58623.2025.11251671	2025	Sports Medicine; Arthroplasty	Deep Learning (DL)	Prognosis & Prediction
2993	10.1109/EMBC58623.2025.11253021	2025	Unclassified	Deep Learning (DL)	Prognosis & Prediction
2994	10.1109/EMBC58623.2025.11253133	2025	Trauma; Pediatric Orthopaedics	Deep Learning (DL)	Segmentation & Measurement
2995	10.1109/EMBC58623.2025.11253486	2025	Arthroplasty	Deep Learning (DL)	Segmentation & Measurement

2996	10.1109/EMBC58623.2025.11251672	2025	Pediatric Orthopaedics; Spine Surgery	Machine Learning (ML)	Prognosis & Prediction
2997	10.1109/EMBC58623.2025.11254420	2025	Spine Surgery	Deep Learning (DL)	Diagnosis & Detection
2998	10.1109/EMBC58623.2025.11252945	2025	Arthroplasty	Deep Learning (DL)	Diagnosis & Detection
2999	N/A	2025	Sports Medicine; Trauma; Spine Surgery	Machine Learning (ML)	Prognosis & Prediction
3000	10.1016/j.phro.2025.100823	2025	Spine Surgery	Deep Learning (DL)	Prognosis & Prediction
3001	N/A	2025	Trauma	Machine Learning (ML)	Prognosis & Prediction
3002	10.13004/kjnt.2025.21.e22	2025	Trauma; Spine Surgery	Machine Learning (ML); Deep Learning (DL)	Diagnosis & Detection
3003	10.3390/diagnostics15141834	2025	Trauma	Natural Language Processing (NLP)	Diagnosis & Detection
3004	10.3390/diagnostics15141787	2025	Trauma	Machine Learning (ML); Deep Learning (DL)	Prognosis & Prediction
3005	10.1002/mco2.70260	2025	Unclassified	Robotics; Deep Learning (DL)	Surgical Planning
3006	10.1002/mp.17949	2025	Unclassified	Deep Learning (DL)	Prognosis & Prediction
3007	10.1016/j.forsciint.2025.112511	2025	Unclassified	Deep Learning (DL)	Segmentation & Measurement
3008	10.1097/JCMA.0000000000001248	2025	Unclassified	Unclassified	Prognosis & Prediction
3009	10.1007/s10067-025-07481-1	2025	Unclassified	Natural Language Processing (NLP); Machine Learning (ML); Deep Learning (DL)	Prognosis & Prediction
3010	10.1016/j.clinimag.2025.110506	2025	Unclassified	Natural Language Processing (NLP); Machine Learning (ML); Deep Learning (DL)	Unclassified
3011	10.1016/j.clinimag.2025.110489	2025	Trauma	Natural Language Processing (NLP)	Diagnosis & Detection
3012	10.1016/j.radonc.2025.110912	2025	Trauma; Spine Surgery	Machine Learning (ML); Deep Learning (DL)	Prognosis & Prediction
3013	10.1016/j.cmpb.2025.108807	2025	Unclassified	Deep Learning (DL)	Unclassified
3014	10.1097/RLU.0000000000005898	2025	Unclassified	Machine Learning (ML); Deep Learning (DL)	Prognosis & Prediction
3015	10.1007/s00414-025-03432-2	2025	Spine Surgery	Natural Language Processing (NLP); Deep Learning (DL)	Prognosis & Prediction

3016	10.54005/geneltip.1634118	2025	Unclassified	Natural Language Processing (NLP)	Unclassified
3017	10.2174/0115734056360714250612080450	2025	Arthroplasty	Deep Learning (DL)	Diagnosis & Detection
3018	10.3324/haematol.2024.286123	2025	Unclassified	Machine Learning (ML); Deep Learning (DL)	Diagnosis & Detection
3019	10.21037/qims-24-2138	2025	Pediatric Orthopaedics; Spine Surgery	Deep Learning (DL)	Prognosis & Prediction
3020	10.1016/j.acra.2025.02.002	2025	Pediatric Orthopaedics	Deep Learning (DL)	Diagnosis & Detection
3021	10.1007/s00259-025-07091-8	2025	Unclassified	Deep Learning (DL)	Prognosis & Prediction
3022	10.1007/s13139-025-00905-5	2025	Unclassified	Machine Learning (ML); Deep Learning (DL)	Segmentation & Measurement
3023	10.1097/RHU.0000000000002198	2025	Unclassified	Natural Language Processing (NLP)	Prognosis & Prediction
3024	10.1016/j.bspc.2025.107512	2025	Unclassified	Deep Learning (DL)	Diagnosis & Detection
3025	10.1002/jmrs.850	2025	Unclassified	Unclassified	Diagnosis & Detection
3026	10.1016/j.jorep.2024.100416	2025	Pediatric Orthopaedics	Robotics	Surgical Planning
3027	10.1016/j.rcot.2024.09.002	2025	Arthroplasty	Machine Learning (ML)	Prognosis & Prediction
3028	10.61882/johe.14.3.214	2025	Unclassified	Unclassified	Prognosis & Prediction
3029	10.13104/imri.2025.0005	2025	Spine Surgery	Unclassified	Unclassified
3030	N/A	2025	Trauma	Machine Learning (ML)	Prognosis & Prediction
3031	10.3390/ime4020014	2025	Unclassified	Natural Language Processing (NLP)	Prognosis & Prediction
3032	10.3390/medicina61060954	2025	Trauma; Pediatric Orthopaedics	Machine Learning (ML); Deep Learning (DL)	Prognosis & Prediction
3033	10.1302/0301-620X.107B6.BJJ-2024-1567.R1	2025	Trauma; Arthroplasty	Deep Learning (DL)	Prognosis & Prediction
3034	10.1167/tvst.14.6.1	2025	Pediatric Orthopaedics; Arthroplasty	Deep Learning (DL)	Prognosis & Prediction
3035	10.3390/jcm14113669	2025	Spine Surgery; Arthroplasty	Machine Learning (ML); Robotics; Deep Learning (DL)	Diagnosis & Detection
3036	10.31661/jbpe.v0i0.2304-1609	2025	Pediatric Orthopaedics	Machine Learning (ML); Deep Learning (DL)	Segmentation & Measurement
3037	10.3390/diagnostics15111377	2025	Unclassified	Natural Language Processing (NLP); Machine Learning (ML); Deep Learning (DL)	Prognosis & Prediction
3038	10.3174/ajnr.A8572	2025	Arthroplasty	Deep Learning (DL)	Diagnosis & Detection

3039	10.1016/j.jhsa.2025.01.021	2025	Trauma	Machine Learning (ML); Deep Learning (DL)	Prognosis & Prediction
3040	10.1093/bjr/tqaf059	2025	Spine Surgery	Deep Learning (DL)	Diagnosis & Detection
3041	10.1002/mp.17838	2025	Spine Surgery	Deep Learning (DL)	Surgical Planning
3042	10.4103/ija.ija_1060_24	2025	Trauma	Machine Learning (ML)	Prognosis & Prediction
3043	10.1016/j.jtumed.2025.04.001	2025	Unclassified	Deep Learning (DL)	Segmentation & Measurement
3044	10.1053/j.sart.2024.11.005	2025	Arthroplasty	Natural Language Processing (NLP); Machine Learning (ML)	Unclassified
3045	10.1053/j.sart.2024.12.006	2025	Trauma; Arthroplasty	Machine Learning (ML); Deep Learning (DL)	Prognosis & Prediction
3046	10.1016/j.jbo.2025.100682	2025	Unclassified	Machine Learning (ML)	Prognosis & Prediction
3047	10.1016/j.jbo.2025.100681	2025	Trauma	Deep Learning (DL)	Prognosis & Prediction
3048	10.1002/nbm.70045	2025	Sports Medicine; Arthroplasty	Deep Learning (DL)	Prognosis & Prediction
3049	10.1007/s40477-025-01019-6	2025	Trauma; Pediatric Orthopaedics	Natural Language Processing (NLP); Deep Learning (DL)	Diagnosis & Detection
3050	10.1016/j.cmpb.2025.108779	2025	Arthroplasty	Machine Learning (ML)	Prognosis & Prediction
3051	10.1016/j.ejro.2025.100649	2025	Arthroplasty	Machine Learning (ML)	Prognosis & Prediction
3052	10.1016/j.cmpb.2025.108720	2025	Unclassified	Machine Learning (ML); Deep Learning (DL)	Prognosis & Prediction
3053	10.1097/MD.0000000000042644	2025	Pediatric Orthopaedics; Spine Surgery	Deep Learning (DL)	Segmentation & Measurement
3054	10.17305/bb.2024.11341	2025	Trauma; Pediatric Orthopaedics	Deep Learning (DL)	Diagnosis & Detection
3055	10.17305/bb.2024.10802	2025	Trauma	Machine Learning (ML); Deep Learning (DL)	Prognosis & Prediction
3056	10.1016/j.ard.2025.01.012	2025	Arthroplasty	Machine Learning (ML); Deep Learning (DL)	Prognosis & Prediction
3057	10.1016/j.acra.2025.01.023	2025	Trauma	Machine Learning (ML)	Prognosis & Prediction
3058	10.1007/s00261-024-04667-0	2025	Unclassified	Machine Learning (ML)	Prognosis & Prediction
3059	10.1111/os.14250	2025	Trauma	Deep Learning (DL)	Segmentation & Measurement
3060	10.1093/jbmr/zjaf050	2025	Trauma; Spine Surgery	Deep Learning (DL)	Prognosis & Prediction
3061	10.3390/cancers17101615	2025	Arthroplasty	Machine Learning (ML); Robotics; Deep Learning (DL)	Prognosis & Prediction

3062	10.3390/jcm14092942	2025	Arthroplasty	Natural Language Processing (NLP)	Unclassified
3063	10.1016/j.legalmed.2025.102630	2025	Pediatric Orthopaedics; Arthroplasty	Machine Learning (ML); Deep Learning (DL)	Prognosis & Prediction
3064	10.21037/qims-24-1384	2025	Arthroplasty	Machine Learning (ML); Deep Learning (DL)	Segmentation & Measurement
3065	10.1093/dmfr/twaf011	2025	Unclassified	Deep Learning (DL)	Diagnosis & Detection
3066	10.1093/dmfr/twae063	2025	Arthroplasty	Deep Learning (DL)	Diagnosis & Detection
3067	10.1016/j.mric.2025.01.001	2025	Trauma; Spine Surgery	Natural Language Processing (NLP); Machine Learning (ML); Deep Learning (DL)	Diagnosis & Detection
3068	10.1007/s00414-025-03417-1	2025	Unclassified	Machine Learning (ML); Deep Learning (DL)	Prognosis & Prediction
3069	10.1007/s00247-025-06223-4	2025	Trauma; Pediatric Orthopaedics	Deep Learning (DL)	Diagnosis & Detection
3070	10.1007/s00414-025-03412-6	2025	Sports Medicine; Trauma; Pediatric Orthopaedics; Spine Surgery	Machine Learning (ML)	Prognosis & Prediction
3071	10.1016/j.rcot.2024.07.018	2025	Trauma; Arthroplasty	Machine Learning (ML)	Prognosis & Prediction
3072	10.1016/j.jcot.2025.102971	2025	Trauma	Unclassified	Unclassified
3073	10.1016/j.ecoenv.2025.118134	2025	Arthroplasty	Deep Learning (DL)	Prognosis & Prediction
3074	10.1097/MEJ.0000000000001193	2025	Trauma	Deep Learning (DL)	Diagnosis & Detection
3075	10.1016/j.clinph.2025.01.016	2025	Unclassified	Natural Language Processing (NLP); Machine Learning (ML); Deep Learning (DL)	Diagnosis & Detection
3076	10.1002/jcsm.13728	2025	Trauma; Spine Surgery	Deep Learning (DL)	Prognosis & Prediction
3077	10.1016/j.crad.2025.106827	2025	Trauma; Spine Surgery	Deep Learning (DL)	Diagnosis & Detection
3078	10.1016/j.jbo.2024.100659	2025	Unclassified	Machine Learning (ML); Deep Learning (DL)	Prognosis & Prediction
3079	10.1016/j.bspc.2024.107267	2025	Arthroplasty	Deep Learning (DL)	Unclassified
3080	10.1016/j.acra.2024.11.004	2025	Spine Surgery	Deep Learning (DL)	Diagnosis & Detection
3081	10.1007/s11604-024-01702-4	2025	Trauma	Machine Learning (ML); Deep Learning (DL)	Diagnosis & Detection
3082	10.1007/s11357-024-01394-8	2025	Spine Surgery	Machine Learning (ML)	Prognosis & Prediction
3083	10.1007/s00330-024-11056-0	2025	Spine Surgery	Deep Learning (DL)	Segmentation & Measurement

3084	10.1007/s10278-024-01251-2	2025	Spine Surgery	Machine Learning (ML); Deep Learning (DL)	Segmentation & Measurement
3085	10.1007/s10278-024-01242-3	2025	Unclassified	Natural Language Processing (NLP); Deep Learning (DL)	Prognosis & Prediction
3086	10.4067/S0717-95022025000200675	2025	Pediatric Orthopaedics	Deep Learning (DL)	Diagnosis & Detection
3087	10.1177/03000605251333646	2025	Arthroplasty	Machine Learning (ML)	Prognosis & Prediction
3088	10.4103/singaporemedj.SMJ-2023-170	2025	Trauma	Unclassified	Prognosis & Prediction
3089	10.1148/radiol.232834	2025	Pediatric Orthopaedics	Machine Learning (ML); Deep Learning (DL)	Prognosis & Prediction
3090	10.3390/diagnostics15080993	2025	Pediatric Orthopaedics	Deep Learning (DL)	Prognosis & Prediction
3091	10.3390/medicina61040737	2025	Arthroplasty	Machine Learning (ML); Deep Learning (DL)	Surgical Planning
3092	10.3390/photonics12040324	2025	Arthroplasty	Deep Learning (DL)	Segmentation & Measurement
3093	10.1093/jbmrpl/ziaf028	2025	Unclassified	Natural Language Processing (NLP); Deep Learning (DL)	Segmentation & Measurement
3094	10.3174/ajnr.A8567	2025	Spine Surgery	Deep Learning (DL)	Diagnosis & Detection
3095	10.34172/aim.32070	2025	Pediatric Orthopaedics	Deep Learning (DL)	Prognosis & Prediction
3096	10.1016/j.legalmed.2025.102612	2025	Unclassified	Deep Learning (DL)	Diagnosis & Detection
3097	10.54079/jpmi.39.1.3492	2025	Sports Medicine; Pediatric Orthopaedics	Machine Learning (ML); Deep Learning (DL)	Unclassified
3098	10.1136/bmjopen-2024-092982	2025	Unclassified	Natural Language Processing (NLP)	Prognosis & Prediction
3099	10.1097/MD.0000000000041987	2025	Arthroplasty	Machine Learning (ML); Deep Learning (DL)	Prognosis & Prediction
3100	10.21037/qims-24-283	2025	Trauma; Pediatric Orthopaedics	Deep Learning (DL)	Prognosis & Prediction
3101	10.21037/qims-24-1745	2025	Unclassified	Deep Learning (DL)	Prognosis & Prediction
3102	10.3390/jcm14051459	2025	Pediatric Orthopaedics	Machine Learning (ML)	Prognosis & Prediction
3103	10.4274/dir.2024.242830	2025	Unclassified	Machine Learning (ML); Deep Learning (DL)	Unclassified
3104	10.14744/tjtes.2025.07724	2025	Trauma	Natural Language Processing (NLP)	Prognosis & Prediction
3105	10.1016/j.shaw.2025.01.003	2025	Arthroplasty	Machine Learning (ML)	Prognosis & Prediction

3106	10.1093/rheumatology/keae215	2025	Trauma; Arthroplasty	Deep Learning (DL)	Prognosis & Prediction
3107	10.1017/S0263574725000086	2025	Arthroplasty	Machine Learning (ML); Deep Learning (DL)	Unclassified
3108	10.1007/s00198-024-07378-w	2025	Spine Surgery	Deep Learning (DL)	Prognosis & Prediction
3109	10.1007/s00198-024-07358-0	2025	Trauma; Spine Surgery	Natural Language Processing (NLP)	Unclassified
3110	10.1016/j.compbiomed.2025.109785	2025	Arthroplasty	Deep Learning (DL)	Diagnosis & Detection
3111	10.1016/j.jbiomech.2025.112552	2025	Arthroplasty	Deep Learning (DL)	Prognosis & Prediction
3112	10.1177/02841851241307333	2025	Pediatric Orthopaedics	Machine Learning (ML); Deep Learning (DL)	Surgical Planning
3113	10.1016/j.ijmedinf.2024.105752	2025	Spine Surgery	Machine Learning (ML)	Prognosis & Prediction
3114	10.1016/j.bonr.2024.101821	2025	Unclassified	Natural Language Processing (NLP); Machine Learning (ML); Deep Learning (DL)	Prognosis & Prediction
3115	10.1016/j.fri.2024.200619	2025	Pediatric Orthopaedics; Arthroplasty	Deep Learning (DL)	Prognosis & Prediction
3116	10.1016/j.bone.2024.117321	2025	Spine Surgery	Deep Learning (DL)	Diagnosis & Detection
3117	10.1016/j.bspc.2024.107172	2025	Arthroplasty	Natural Language Processing (NLP); Deep Learning (DL)	Prognosis & Prediction
3118	10.1053/j.sart.2024.09.001	2025	Arthroplasty	Natural Language Processing (NLP); Deep Learning (DL)	Unclassified
3119	10.1002/lary.31809	2025	Unclassified	Natural Language Processing (NLP); Machine Learning (ML); Deep Learning (DL)	Surgical Planning
3120	10.1097/RLI.0000000000001118	2025	Sports Medicine; Arthroplasty	Deep Learning (DL)	Diagnosis & Detection
3121	10.1002/adsr.202400160	2025	Arthroplasty	Machine Learning (ML); Deep Learning (DL)	Diagnosis & Detection
3122	10.2214/AJR.24.32076	2025	Trauma; Spine Surgery	Machine Learning (ML)	Diagnosis & Detection
3123	10.3390/tomography11030027	2025	Arthroplasty	Deep Learning (DL)	Segmentation & Measurement
3124	10.3390/biomedinformatics5010003	2025	Spine Surgery	Machine Learning (ML); Deep Learning (DL)	Prognosis & Prediction
3125	10.3390/jcm14061983	2025	Trauma	Natural Language Processing (NLP)	Unclassified

3126	10.1148/radiol.242133	2025	Trauma; Spine Surgery	Machine Learning (ML); Deep Learning (DL)	Prognosis & Prediction
3127	10.1093/jbmr/zjaf009	2025	Spine Surgery; Arthroplasty	Machine Learning (ML)	Segmentation & Measurement
3128	10.1002/alz.14624	2025	Arthroplasty	Machine Learning (ML)	Prognosis & Prediction
3129	10.3390/metabo15020112	2025	Arthroplasty	Machine Learning (ML)	Prognosis & Prediction
3130	10.3390/diagnostics15030257	2025	Unclassified	Natural Language Processing (NLP); Machine Learning (ML); Deep Learning (DL)	Prognosis & Prediction
3131	10.3390/jcm14030690	2025	Unclassified	Unclassified	Unclassified
3132	10.1177/03000605251318195	2025	Arthroplasty	Deep Learning (DL)	Prognosis & Prediction
3133	10.1093/dmfr/twae070	2025	Unclassified	Machine Learning (ML); Deep Learning (DL)	Prognosis & Prediction
3134	10.1016/j.cca.2024.120112	2025	Arthroplasty	Deep Learning (DL)	Prognosis & Prediction
3135	10.1016/j.jbo.2024.100654	2025	Unclassified	Deep Learning (DL)	Surgical Planning
3136	10.1007/s10140-024-02300-7	2025	Trauma	Deep Learning (DL)	Diagnosis & Detection
3137	10.1007/s10140-024-02298-y	2025	Trauma	Natural Language Processing (NLP)	Diagnosis & Detection
3138	10.1016/j.asmr.2024.101027	2025	Sports Medicine; Trauma; Arthroplasty	Natural Language Processing (NLP)	Unclassified
3139	10.1016/j.archger.2024.105641	2025	Trauma	Machine Learning (ML)	Prognosis & Prediction
3140	10.1016/j.ijmedinf.2024.105692	2025	Pediatric Orthopaedics	Machine Learning (ML); Deep Learning (DL)	Prognosis & Prediction
3141	10.1016/j.neurad.2024.101231	2025	Unclassified	Machine Learning (ML); Deep Learning (DL)	Diagnosis & Detection
3142	10.1016/j.bspc.2024.106983	2025	Arthroplasty	Deep Learning (DL)	Prognosis & Prediction
3143	10.1016/j.jacr.2024.08.026	2025	Trauma; Spine Surgery	Unclassified	Diagnosis & Detection
3144	10.1016/j.bspc.2024.106791	2025	Arthroplasty	Robotics; Deep Learning (DL)	Prognosis & Prediction
3145	10.1007/s10278-024-01156-0	2025	Trauma; Pediatric Orthopaedics	Unclassified	Prognosis & Prediction
3146	10.1007/s10278-024-01218-3	2025	Unclassified	Deep Learning (DL)	Unclassified
3147	10.1007/s10278-024-01027-8	2025	Trauma	Deep Learning (DL)	Prognosis & Prediction

3148	10.52711/0974-360X.2025.00108	2025	Unclassified	Deep Learning (DL)	Diagnosis & Detection
3149	10.17305/BB.2024.10725	2025	Trauma	Machine Learning (ML)	Prognosis & Prediction
3150	10.21037/qims-24-799	2025	Sports Medicine; Trauma; Arthroplasty	Natural Language Processing (NLP); Machine Learning (ML)	Prognosis & Prediction
3151	10.1177/08953996241299998	2025	Spine Surgery	Deep Learning (DL)	Diagnosis & Detection
3152	10.3389/fimmu.2025.1550794	2025	Sports Medicine; Arthroplasty	Machine Learning (ML)	Unclassified
3153	10.3389/fnut.2025.1552047	2025	Arthroplasty	Machine Learning (ML)	Prognosis & Prediction
3154	10.3389/fonc.2025.1549851	2025	Unclassified	Machine Learning (ML)	Prognosis & Prediction
3155	10.2147/CIA.S507138	2025	Trauma; Arthroplasty	Machine Learning (ML); Deep Learning (DL)	Prognosis & Prediction
3156	10.1080/21691401.2025.2471762	2025	Arthroplasty	Machine Learning (ML)	Prognosis & Prediction
3157	10.3389/fgene.2025.1513675	2025	Arthroplasty	Machine Learning (ML)	Diagnosis & Detection
3158	10.3389/fmicb.2025.1528865	2025	Arthroplasty	Machine Learning (ML); Deep Learning (DL)	Prognosis & Prediction
3159	10.1097/CORR.0000000000003413	2025	Arthroplasty	Natural Language Processing (NLP)	Prognosis & Prediction
3160	10.3389/fimmu.2025.1517646	2025	Arthroplasty	Machine Learning (ML)	Prognosis & Prediction
3161	10.1016/j.ibmed.2025.100205	2025	Trauma; Spine Surgery	Machine Learning (ML)	Prognosis & Prediction
3162	10.46747/cfp.710110	2025	Arthroplasty	Unclassified	Unclassified
3163	10.1080/21681163.2025.2455752	2025	Pediatric Orthopaedics; Arthroplasty	Deep Learning (DL)	Surgical Planning
3164	10.1136/ip-2024-045358	2025	Trauma	Machine Learning (ML)	Prognosis & Prediction
3165	10.3390/jpm15010021	2025	Trauma; Arthroplasty	Machine Learning (ML); Robotics; Deep Learning (DL)	Prognosis & Prediction
3166	10.3390/cancers17020218	2025	Unclassified	Natural Language Processing (NLP)	Prognosis & Prediction
3167	10.1177/14604582251315587	2025	Unclassified	Natural Language Processing (NLP)	Unclassified
3168	10.2147/IDR.S482584	2025	Trauma; Spine Surgery	Deep Learning (DL)	Prognosis & Prediction
3169	10.1093/jbmr/zjae151	2025	Unclassified	Natural Language Processing (NLP); Deep Learning (DL)	Prognosis & Prediction

3170	10.1371/journal.pdig.0000529	2025	Trauma	Machine Learning (ML)	Prognosis & Prediction
3171	10.1093/dmfr/twae055	2025	Arthroplasty	Deep Learning (DL)	Diagnosis & Detection
3172	10.1093/dmfr/twae040	2025	Arthroplasty	Deep Learning (DL)	Diagnosis & Detection
3173	10.1016/j.rcot.2024.12.005	2025	Pediatric Orthopaedics; Trauma; Spine Surgery	Natural Language Processing (NLP)	Unclassified
3174	10.1159/000542396	2025	Spine Surgery	Deep Learning (DL)	Prognosis & Prediction
3175	10.1016/S2589-7500(24)00243-7	2025	Spine Surgery; Arthroplasty	Deep Learning (DL)	Surgical Planning
3176	10.1109/JBHI.2024.3501386	2025	Unclassified	Deep Learning (DL)	Diagnosis & Detection
3177	10.1016/j.artmed.2024.103027	2025	Trauma	Natural Language Processing (NLP)	Prognosis & Prediction
3178	10.1016/j.bone.2024.117317	2025	Trauma	Deep Learning (DL)	Prognosis & Prediction
3179	10.1089/cbr.2024.0162	2025	Unclassified	Machine Learning (ML)	Prognosis & Prediction
3180	10.1136/ard-2024-225862	2025	Arthroplasty	Machine Learning (ML); Deep Learning (DL)	Prognosis & Prediction
3181	10.1177/01617346241277178	2025	Arthroplasty	Deep Learning (DL)	Diagnosis & Detection
3182	10.1016/j.diii.2024.09.004	2025	Trauma	Deep Learning (DL)	Diagnosis & Detection
3183	10.1136/ard-2024-225872	2025	Unclassified	Machine Learning (ML)	Prognosis & Prediction
3184	10.1002/mrm.30275	2025	Pediatric Orthopaedics	Deep Learning (DL)	Segmentation & Measurement
3185	10.1007/s00330-024-10967-2	2025	Unclassified	Deep Learning (DL)	Diagnosis & Detection
3186	10.1007/s00330-024-10902-5	2025	Unclassified	Natural Language Processing (NLP)	Diagnosis & Detection
3187	10.1007/s00330-024-10928-9	2025	Arthroplasty	Deep Learning (DL)	Prognosis & Prediction
3188	10.1016/j.acra.2024.06.041	2025	Trauma; Spine Surgery	Machine Learning (ML)	Prognosis & Prediction
3189	10.1016/j.recot.2024.05.005	2025	Trauma	Machine Learning (ML)	Prognosis & Prediction
3190	10.1080/14740338.2024.2348569	2025	Trauma; Arthroplasty	Machine Learning (ML)	Prognosis & Prediction
3191	10.1111/ocr.70087	2025	Unclassified	Machine Learning (ML)	Prognosis & Prediction
3192	10.1155/ijbi/9065572	2025	Unclassified	Machine Learning (ML); Deep Learning (DL)	Prognosis & Prediction

3193	10.3389/fpubh.2025.1719047	2025	Unclassified	Natural Language Processing (NLP)	Prognosis & Prediction
3194	10.2196/75607	2025	Unclassified	Natural Language Processing (NLP)	Unclassified
3195	10.1016/j.oooo.2025.10.020	2025	Unclassified	Deep Learning (DL)	Diagnosis & Detection
3196	10.1016/j.acra.2025.11.023	2025	Trauma; Spine Surgery	Natural Language Processing (NLP); Deep Learning (DL)	Prognosis & Prediction
3197	10.1016/j.acra.2025.11.022	2025	Trauma; Pediatric Orthopaedics	Deep Learning (DL)	Diagnosis & Detection
3198	10.1016/j.rcl.2025.09.001	2025	Sports Medicine	Machine Learning (ML)	Unclassified
3199	10.1177/20552076251406311	2025	Trauma; Arthroplasty	Machine Learning (ML)	Prognosis & Prediction
3200	10.1007/s00247-025-06492-z	2025	Sports Medicine; Pediatric Orthopaedics; Spine Surgery	Deep Learning (DL)	Surgical Planning
3201	10.1093/bjro/tzaf029	2025	Sports Medicine; Trauma	Natural Language Processing (NLP); Deep Learning (DL)	Diagnosis & Detection
3202	10.1016/j.jse.2025.10.011	2025	Arthroplasty	Machine Learning (ML); Deep Learning (DL)	Prognosis & Prediction
3203	10.1007/s00774-025-01672-1	2025	Trauma; Spine Surgery	Deep Learning (DL)	Prognosis & Prediction
3204	10.1515/jpem-2025-0142	2025	Pediatric Orthopaedics	Unclassified	Prognosis & Prediction
3205	10.1007/s00414-025-03669-x	2025	Trauma	Deep Learning (DL)	Diagnosis & Detection
3206	10.3389/fmed.2025.1670824	2025	Sports Medicine; Trauma	Natural Language Processing (NLP)	Unclassified
3207	10.1007/s00330-025-12049-3	2025	Trauma; Spine Surgery	Deep Learning (DL)	Prognosis & Prediction
3208	10.1007/s12194-025-00987-y	2025	Arthroplasty	Deep Learning (DL)	Unclassified
3209	10.1080/00016489.2025.2588812	2025	Unclassified	Deep Learning (DL)	Surgical Planning
3210	10.1007/s11282-025-00878-3	2025	Trauma	Machine Learning (ML); Deep Learning (DL)	Prognosis & Prediction
3211	10.1007/s00247-025-06461-6	2025	Pediatric Orthopaedics; Trauma; Arthroplasty	Unclassified	Prognosis & Prediction
3212	10.1016/j.recot.2025.10.001	2025	Trauma	Natural Language Processing (NLP); Machine Learning (ML)	Diagnosis & Detection
3213	10.3389/fmed.2025.1699842	2025	Arthroplasty	Machine Learning (ML)	Prognosis & Prediction

3214	10.1016/j.recot.2025.09.005	2025	Trauma	Robotics	Prognosis & Prediction
3215	10.3389/fmed.2025.1714792	2025	Arthroplasty	Machine Learning (ML)	Prognosis & Prediction
3216	10.3389/fcvm.2025.1652467	2025	Arthroplasty	Machine Learning (ML)	Diagnosis & Detection
3217	10.1007/s00247-025-06456-3	2025	Trauma	Unclassified	Prognosis & Prediction
3218	10.1109/JBHI.2025.3631045	2025	Arthroplasty	Natural Language Processing (NLP); Deep Learning (DL)	Diagnosis & Detection
3219	10.3389/fradi.2025.1673619	2025	Sports Medicine; Trauma; Arthroplasty	Unclassified	Unclassified
3220	10.1080/07853890.2025.2584361	2025	Unclassified	Deep Learning (DL)	Surgical Planning
3221	10.1016/j.joca.2025.10.012	2025	Unclassified	Machine Learning (ML); Deep Learning (DL)	Prognosis & Prediction
3222	10.4274/balkanmedj.galenos.2025.2025-8-125	2025	Trauma	Machine Learning (ML)	Prognosis & Prediction
3223	10.3389/fradi.2025.1651798	2025	Trauma; Spine Surgery	Deep Learning (DL)	Prognosis & Prediction
3224	10.1007/s00414-025-03635-7	2025	Unclassified	Machine Learning (ML)	Prognosis & Prediction
3225	10.3389/fimmu.2025.1635818	2025	Sports Medicine; Arthroplasty	Machine Learning (ML)	Prognosis & Prediction
3226	10.1016/j.jse.2025.07.025	2025	Arthroplasty	Machine Learning (ML); Deep Learning (DL)	Prognosis & Prediction
3227	10.2196/70857	2025	Arthroplasty	Machine Learning (ML)	Prognosis & Prediction
3228	10.3389/fonc.2025.1653506	2025	Unclassified	Machine Learning (ML); Deep Learning (DL)	Prognosis & Prediction
3229	10.5114/pjr/207511	2025	Arthroplasty	Machine Learning (ML); Deep Learning (DL)	Prognosis & Prediction
3230	10.1109/TRPMS.2025.3617225	2025	Unclassified	Deep Learning (DL)	Unclassified
3231	10.1007/s00330-025-12029-7	2025	Arthroplasty	Deep Learning (DL)	Unclassified
3232	10.1016/j.recot.2025.08.004	2025	Trauma	Natural Language Processing (NLP)	Diagnosis & Detection
3233	10.3389/fmed.2025.1471746	2025	Arthroplasty	Machine Learning (ML)	Prognosis & Prediction
3234	10.2196/75665	2025	Trauma; Spine Surgery	Machine Learning (ML); Deep Learning (DL)	Prognosis & Prediction

3235	10.2147/JMDH.S536633	2025	Arthroplasty	Natural Language Processing (NLP); Machine Learning (ML); Deep Learning (DL)	Prognosis & Prediction
3236	10.3389/fmed.2025.1664219	2025	Trauma; Spine Surgery	Machine Learning (ML)	Prognosis & Prediction
3237	10.1016/j.arth.2025.08.023	2025	Arthroplasty	Machine Learning (ML); Deep Learning (DL)	Segmentation & Measurement
3238	10.1016/j.arth.2025.08.072	2025	Arthroplasty	Machine Learning (ML); Deep Learning (DL)	Prognosis & Prediction
3239	10.1007/s00247-025-06405-0	2025	Pediatric Orthopaedics	Unclassified	Unclassified
3240	10.1177/1759720X251357157	2025	Trauma; Arthroplasty	Machine Learning (ML); Deep Learning (DL)	Prognosis & Prediction
3241	10.1109/TNSRE.2025.3610281	2025	Arthroplasty	Robotics; Deep Learning (DL)	Prognosis & Prediction
3242	10.3389/fendo.2025.1528392	2025	Unclassified	Machine Learning (ML)	Prognosis & Prediction
3243	10.3389/fendo.2025.1635451	2025	Unclassified	Machine Learning (ML); Deep Learning (DL)	Prognosis & Prediction
3244	10.3389/fbioe.2025.1613417	2025	Trauma; Pediatric Orthopaedics	Deep Learning (DL)	Prognosis & Prediction
3245	10.5435/JAAOS-D-24-01501	2025	Spine Surgery	Natural Language Processing (NLP); Machine Learning (ML); Deep Learning (DL)	Prognosis & Prediction
3246	10.1097/SCS.0000000000011892	2025	Trauma	Deep Learning (DL)	Unclassified
3247	10.5435/JAAOS-D-25-00456	2025	Trauma	Natural Language Processing (NLP); Machine Learning (ML)	Prognosis & Prediction
3248	10.3389/fphys.2025.1662104	2025	Unclassified	Deep Learning (DL)	Prognosis & Prediction
3249	10.1007/s00414-025-03587-y	2025	Spine Surgery	Deep Learning (DL)	Prognosis & Prediction
3250	10.1007/s10278-025-01636-x	2025	Arthroplasty	Natural Language Processing (NLP); Machine Learning (ML); Deep Learning (DL)	Prognosis & Prediction
3251	10.1097/CORR.0000000000003660	2025	Pediatric Orthopaedics; Trauma; Arthroplasty	Deep Learning (DL)	Prognosis & Prediction
3252	10.3389/fonc.2025.1621677	2025	Unclassified	Machine Learning (ML); Deep Learning (DL)	Prognosis & Prediction

3253	10.3389/fonc.2025.1613133	2025	Unclassified	Machine Learning (ML); Deep Learning (DL)	Prognosis & Prediction
3254	10.3389/fimmu.2025.1596912	2025	Sports Medicine	Machine Learning (ML)	Prognosis & Prediction
3255	10.3389/fped.2025.1531827	2025	Pediatric Orthopaedics	Machine Learning (ML); Deep Learning (DL)	Prognosis & Prediction
3256	10.1007/s00330-025-11947-w	2025	Pediatric Orthopaedics; Trauma; Arthroplasty	Machine Learning (ML); Deep Learning (DL)	Prognosis & Prediction
3257	10.2147/JPR.S529465	2025	Spine Surgery	Machine Learning (ML); Deep Learning (DL)	Unclassified
3258	10.1227/neu.0000000000003683	2025	Spine Surgery	Natural Language Processing (NLP)	Diagnosis & Detection
3259	10.3389/fbioe.2025.1644261	2025	Trauma	Machine Learning (ML)	Prognosis & Prediction
3260	10.2147/OARRR.S541568	2025	Unclassified	Machine Learning (ML)	Prognosis & Prediction
3261	10.1016/j.jorep.2025.100760	2025	Spine Surgery	Machine Learning (ML); Deep Learning (DL)	Prognosis & Prediction
3262	10.3389/fgene.2025.1595676	2025	Unclassified	Machine Learning (ML)	Diagnosis & Detection
3263	10.2196/64536	2025	Unclassified	Natural Language Processing (NLP); Deep Learning (DL)	Prognosis & Prediction
3264	10.2463/mrms.mp.2023-0115	2025	Arthroplasty	Deep Learning (DL)	Diagnosis & Detection
3265	10.1007/s10278-025-01623-2	2025	Pediatric Orthopaedics	Deep Learning (DL)	Prognosis & Prediction
3266	10.3389/fendo.2025.1604133	2025	Pediatric Orthopaedics	Deep Learning (DL)	Diagnosis & Detection
3267	10.1504/IJMEI.2025.147586	2025	Unclassified	Deep Learning (DL)	Segmentation & Measurement
3268	10.7150/ijms.114419	2025	Trauma; Spine Surgery	Natural Language Processing (NLP); Machine Learning (ML); Deep Learning (DL)	Prognosis & Prediction
3269	10.1016/j.jorep.2025.100734	2025	Arthroplasty	Robotics	Prognosis & Prediction
3270	10.1097/BRS.0000000000005448	2025	Trauma; Spine Surgery	Deep Learning (DL)	Prognosis & Prediction
3271	10.3389/fmed.2025.1534117	2025	Trauma; Arthroplasty	Machine Learning (ML); Deep Learning (DL)	Surgical Planning
3272	10.2147/JMDH.S525183	2025	Trauma	Unclassified	Diagnosis & Detection

3273	10.3389/fmed.2025.1616923	2025	Trauma; Spine Surgery	Machine Learning (ML)	Prognosis & Prediction
3274	10.3389/fmed.2025.1553274	2025	Sports Medicine; Trauma; Arthroplasty	Machine Learning (ML)	Prognosis & Prediction
3275	10.3389/fmed.2025.1630824	2025	Arthroplasty	Unclassified	Prognosis & Prediction
3276	10.3389/fmed.2025.1574546	2025	Unclassified	Machine Learning (ML); Robotics	Prognosis & Prediction
3277	10.3389/fmed.2025.1620687	2025	Unclassified	Machine Learning (ML); Deep Learning (DL)	Prognosis & Prediction
3278	10.1007/s11282-025-00839-w	2025	Unclassified	Deep Learning (DL)	Diagnosis & Detection
3279	10.1007/s10278-025-01579-3	2025	Unclassified	Natural Language Processing (NLP); Machine Learning (ML); Deep Learning (DL)	Prognosis & Prediction
3280	10.1007/s10278-025-01603-6	2025	Trauma	Natural Language Processing (NLP)	Diagnosis & Detection
3281	10.3389/fimmu.2025.1599930	2025	Unclassified	Machine Learning (ML); Deep Learning (DL)	Prognosis & Prediction
3282	10.1590/S1808-185120252402293157	2025	Pediatric Orthopaedics; Spine Surgery	Natural Language Processing (NLP)	Surgical Planning
3283	10.3171/2025.3.FOCUS2574	2025	Pediatric Orthopaedics; Spine Surgery	Machine Learning (ML)	Prognosis & Prediction
3284	10.3389/fphar.2025.1564157	2025	Unclassified	Deep Learning (DL)	Prognosis & Prediction
3285	10.1055/a-2594-7085	2025	Unclassified	Natural Language Processing (NLP)	Diagnosis & Detection
3286	10.3389/fonc.2025.1498832	2025	Unclassified	Deep Learning (DL)	Segmentation & Measurement
3287	10.1016/j.jorep.2025.100697	2025	Unclassified	Natural Language Processing (NLP)	Prognosis & Prediction
3288	10.1097/BRS.0000000000005411	2025	Spine Surgery	Machine Learning (ML)	Prognosis & Prediction
3289	10.2147/RMHP.S503286	2025	Arthroplasty	Machine Learning (ML)	Prognosis & Prediction
3290	10.3389/fbioe.2025.1570022	2025	Pediatric Orthopaedics; Spine Surgery	Deep Learning (DL)	Prognosis & Prediction
3291	10.3389/fonc.2025.1603672	2025	Trauma; Spine Surgery	Machine Learning (ML); Deep Learning (DL)	Prognosis & Prediction
3292	10.2196/65890	2025	Unclassified	Machine Learning (ML)	Prognosis & Prediction

3293	10.1016/j.jorep.2025.100683	2025	Arthroplasty	Natural Language Processing (NLP)	Diagnosis & Detection
3294	10.2147/TCRM.S518774	2025	Trauma	Machine Learning (ML)	Prognosis & Prediction
3295	10.2196/63022	2025	Arthroplasty	Unclassified	Prognosis & Prediction
3296	10.2147/CIA.S511935	2025	Trauma	Machine Learning (ML)	Prognosis & Prediction
3297	10.18019/1028-4427-2025-31-2-237-244	2025	Trauma	Deep Learning (DL)	Prognosis & Prediction
3298	10.1007/s00428-025-04122-5	2025	Unclassified	Deep Learning (DL)	Prognosis & Prediction
3299	10.2196/67830	2025	Unclassified	Natural Language Processing (NLP)	Unclassified
3300	10.3389/fdgth.2025.1523953	2025	Trauma; Arthroplasty	Natural Language Processing (NLP); Deep Learning (DL)	Prognosis & Prediction
3301	10.3389/fmed.2025.1511487	2025	Trauma	Deep Learning (DL)	Surgical Planning
3302	10.3389/fmed.2025.1574305	2025	Trauma; Spine Surgery	Machine Learning (ML)	Prognosis & Prediction
3303	10.3389/fphar.2025.1516013	2025	Unclassified	Machine Learning (ML); Deep Learning (DL)	Prognosis & Prediction
3304	10.22141/pjs.15.1.2025.449	2025	Trauma	Unclassified	Prognosis & Prediction
3305	10.3389/fmed.2025.1534988	2025	Trauma	Machine Learning (ML)	Prognosis & Prediction
3306	10.1177/20552076251325960	2025	Unclassified	Machine Learning (ML)	Prognosis & Prediction
3307	10.3389/fmed.2025.1499670	2025	Trauma; Spine Surgery	Deep Learning (DL)	Prognosis & Prediction
3308	10.1080/17434440.2025.2484252	2025	Spine Surgery; Arthroplasty	Robotics	Prognosis & Prediction
3309	10.3389/fradi.2025.1503625	2025	Spine Surgery	Deep Learning (DL)	Prognosis & Prediction
3310	10.1016/j.cjtee.2024.10.006	2025	Trauma	Deep Learning (DL)	Surgical Planning
3311	10.3389/fmed.2025.1547588	2025	Sports Medicine; Trauma	Deep Learning (DL)	Prognosis & Prediction
3312	10.1177/24730114251322784	2025	Arthroplasty	Natural Language Processing (NLP)	Prognosis & Prediction
3313	10.3389/fcimb.2025.1461740	2025	Trauma; Spine Surgery	Machine Learning (ML)	Prognosis & Prediction
3314	10.1155/ijcp/8793065	2025	Arthroplasty	Unclassified	Prognosis & Prediction

3315	10.2196/64705	2025	Trauma; Arthroplasty	Natural Language Processing (NLP); Machine Learning (ML)	Prognosis & Prediction
3316	10.1177/20552076251326226	2025	Unclassified	Machine Learning (ML)	Prognosis & Prediction
3317	10.1097/MD.0000000000040846	2024	Pediatric Orthopaedics; Arthroplasty	Machine Learning (ML)	Prognosis & Prediction
3318	10.1136/bmjopen-2024-084448	2024	Trauma; Pediatric Orthopaedics	Unclassified	Prognosis & Prediction
3319	10.1136/bmjhci-2024-101091	2024	Trauma; Pediatric Orthopaedics	Robotics	Prognosis & Prediction
3320	10.1186/s12909-024-06592-8	2024	Unclassified	Natural Language Processing (NLP); Machine Learning (ML)	Prognosis & Prediction
3321	10.3390/clinpract14060197	2024	Sports Medicine; Trauma	Natural Language Processing (NLP); Machine Learning (ML); Deep Learning (DL)	Prognosis & Prediction
3322	10.3390/healthcare12242560	2024	Unclassified	Machine Learning (ML)	Prognosis & Prediction
3323	10.1186/s12871-024-02832-y	2024	Trauma; Arthroplasty	Machine Learning (ML)	Prognosis & Prediction
3324	10.1016/j.jocmr.2024.101123	2024	Arthroplasty	Deep Learning (DL)	Prognosis & Prediction
3325	10.1186/s13244-024-01861-y	2024	Trauma; Spine Surgery	Deep Learning (DL)	Surgical Planning
3326	10.1186/s12880-024-01489-w	2024	Spine Surgery	Deep Learning (DL)	Prognosis & Prediction
3327	10.1186/s12911-024-02740-8	2024	Spine Surgery	Natural Language Processing (NLP); Deep Learning (DL)	Surgical Planning
3328	10.1016/j.jbo.2024.100648	2024	Arthroplasty	Machine Learning (ML); Deep Learning (DL)	Prognosis & Prediction
3329	10.1186/s12984-024-01484-w	2024	Spine Surgery	Machine Learning (ML)	Diagnosis & Detection
3330	10.1016/j.compbiomed.2024.109309	2024	Arthroplasty	Robotics; Deep Learning (DL)	Unclassified
3331	10.1053/j.sart.2024.07.010	2024	Arthroplasty	Machine Learning (ML); Deep Learning (DL)	Prognosis & Prediction
3332	10.1016/j.jbo.2024.100641	2024	Spine Surgery	Deep Learning (DL)	Prognosis & Prediction
3333	10.1186/s40644-024-00784-7	2024	Pediatric Orthopaedics	Deep Learning (DL)	Diagnosis & Detection

3334	10.47197/retos.v61.109799	2024	Sports Medicine	Robotics; Deep Learning (DL)	Prognosis & Prediction
3335	10.1016/j.oooo.2024.07.010	2024	Trauma; Pediatric Orthopaedics	Deep Learning (DL)	Diagnosis & Detection
3336	10.1038/s41746-024-01265-8	2024	Arthroplasty	Machine Learning (ML); Deep Learning (DL)	Prognosis & Prediction
3337	10.1016/j.crad.2024.09.004	2024	Spine Surgery; Arthroplasty	Deep Learning (DL)	Diagnosis & Detection
3338	10.1002/ird3.99	2024	Unclassified	Machine Learning (ML); Deep Learning (DL)	Prognosis & Prediction
3339	10.1016/j.ejrad.2024.111751	2024	Arthroplasty	Machine Learning (ML)	Prognosis & Prediction
3340	10.1007/s12672-024-01327-z	2024	Unclassified	Machine Learning (ML); Deep Learning (DL)	Prognosis & Prediction
3341	10.1016/j.ijmedinf.2024.105634	2024	Arthroplasty	Machine Learning (ML)	Prognosis & Prediction
3342	10.1186/s41747-024-00504-7	2024	Sports Medicine; Arthroplasty	Deep Learning (DL)	Diagnosis & Detection
3343	10.1016/j.pacs.2024.100645	2024	Arthroplasty	Deep Learning (DL)	Prognosis & Prediction
3344	10.1016/j.ijmedinf.2024.105631	2024	Arthroplasty	Machine Learning (ML); Deep Learning (DL)	Prognosis & Prediction
3345	10.1016/j.ejrad.2024.111714	2024	Trauma; Spine Surgery	Machine Learning (ML)	Prognosis & Prediction
3346	10.1186/s12938-024-01272-6	2024	Pediatric Orthopaedics; Spine Surgery	Machine Learning (ML); Deep Learning (DL)	Prognosis & Prediction
3347	10.1186/s12905-024-03264-z	2024	Unclassified	Machine Learning (ML)	Prognosis & Prediction
3348	10.1186/s12880-024-01378-2	2024	Pediatric Orthopaedics	Deep Learning (DL)	Prognosis & Prediction
3349	10.1007/s00405-024-08862-z	2024	Unclassified	Machine Learning (ML)	Prognosis & Prediction
3350	10.1038/s41698-024-00649-z	2024	Unclassified	Machine Learning (ML); Deep Learning (DL)	Prognosis & Prediction
3351	10.1186/s40658-024-00666-8	2024	Pediatric Orthopaedics; Arthroplasty	Deep Learning (DL)	Prognosis & Prediction
3352	10.1016/j.jpi.2024.100390	2024	Unclassified	Deep Learning (DL)	Prognosis & Prediction
3353	10.1007/s12024-023-00751-x	2024	Trauma	Deep Learning (DL)	Prognosis & Prediction
3354	10.1186/s12880-024-01337-x	2024	Spine Surgery; Arthroplasty	Deep Learning (DL)	Prognosis & Prediction
3355	10.1186/s41747-024-00470-0	2024	Sports Medicine; Spine Surgery	Deep Learning (DL)	Diagnosis & Detection

3356	10.1186/s12877-024-05050-w	2024	Trauma	Machine Learning (ML)	Prognosis & Prediction
3357	10.1186/s12911-024-02540-0	2024	Unclassified	Machine Learning (ML)	Prognosis & Prediction
3358	10.1038/s41390-024-03282-5	2024	Pediatric Orthopaedics	Unclassified	Prognosis & Prediction
3359	10.1186/s12944-024-02073-5	2024	Unclassified	Machine Learning (ML)	Diagnosis & Detection
3360	10.1186/s12984-024-01337-6	2024	Unclassified	Machine Learning (ML)	Prognosis & Prediction
3361	10.1186/s13018-024-04690-3	2024	Trauma; Spine Surgery	Machine Learning (ML)	Prognosis & Prediction
3362	10.1186/s12877-024-04892-8	2024	Trauma	Machine Learning (ML)	Prognosis & Prediction
3363	10.1186/s13244-024-01659-y	2024	Arthroplasty	Machine Learning (ML); Deep Learning (DL)	Prognosis & Prediction
3364	10.1186/s41747-024-00427-3	2024	Arthroplasty	Deep Learning (DL)	Unclassified
3365	10.1186/s13048-024-01365-9	2024	Arthroplasty	Machine Learning (ML); Deep Learning (DL)	Prognosis & Prediction
3366	10.1186/s41747-023-00417-x	2024	Unclassified	Deep Learning (DL)	Prognosis & Prediction
3367	10.1186/s12911-024-02421-6	2024	Unclassified	Machine Learning (ML)	Prognosis & Prediction
3368	10.1186/s13244-024-01610-1	2024	Arthroplasty	Deep Learning (DL)	Diagnosis & Detection
3369	10.1186/s12911-024-02417-2	2024	Trauma	Machine Learning (ML); Deep Learning (DL)	Prognosis & Prediction
3370	10.1186/s13244-023-01600-9	2024	Arthroplasty	Natural Language Processing (NLP); Machine Learning (ML)	Unclassified
3371	10.1186/s41747-023-00420-2	2024	Arthroplasty	Natural Language Processing (NLP); Machine Learning (ML)	Unclassified
3372	10.1007/s10278-024-01144-4	2024	Trauma	Deep Learning (DL)	Prognosis & Prediction
3373	10.1136/bmjopen-2023-077455	2024	Sports Medicine; Trauma	Unclassified	Prognosis & Prediction
3374	10.1002/cam4.70383	2024	Arthroplasty	Machine Learning (ML); Deep Learning (DL)	Prognosis & Prediction

3375	10.3390/jcm13216515	2024	Spine Surgery; Arthroplasty	Machine Learning (ML)	Prognosis & Prediction
3376	10.1093/jamia/ocae246	2024	Arthroplasty	Deep Learning (DL)	Diagnosis & Detection
3377	10.1177/02841851241279896	2024	Pediatric Orthopaedics; Trauma; Spine Surgery	Machine Learning (ML)	Diagnosis & Detection
3378	10.1016/j.crad.2024.08.006	2024	Trauma; Arthroplasty	Deep Learning (DL)	Prognosis & Prediction
3379	10.1016/j.injury.2024.111830	2024	Trauma	Machine Learning (ML)	Prognosis & Prediction
3380	10.1002/acr2.11699	2024	Trauma; Arthroplasty	Machine Learning (ML)	Prognosis & Prediction
3381	10.1016/j.ejrad.2024.111685	2024	Trauma; Spine Surgery	Deep Learning (DL)	Prognosis & Prediction
3382	10.1016/j.jhsg.2024.06.002	2024	Trauma	Machine Learning (ML)	Prognosis & Prediction
3383	10.1016/j.bspc.2024.106670	2024	Unclassified	Machine Learning (ML); Deep Learning (DL)	Prognosis & Prediction
3384	10.1097/RCT.0000000000001637	2024	Spine Surgery	Deep Learning (DL)	Diagnosis & Detection
3385	10.1007/s00414-024-03282-4	2024	Pediatric Orthopaedics; Arthroplasty	Deep Learning (DL)	Prognosis & Prediction
3386	10.1007/s12020-024-03931-z	2024	Trauma; Spine Surgery	Machine Learning (ML); Deep Learning (DL)	Prognosis & Prediction
3387	10.1007/s00414-024-03268-2	2024	Pediatric Orthopaedics	Machine Learning (ML); Deep Learning (DL)	Prognosis & Prediction
3388	10.1177/08465371241231577	2024	Pediatric Orthopaedics; Spine Surgery	Machine Learning (ML)	Prognosis & Prediction
3389	10.1055/s-0044-1789218	2024	Unclassified	Natural Language Processing (NLP); Machine Learning (ML); Deep Learning (DL)	Prognosis & Prediction
3390	10.4274/jtss.galenos.2024.64936	2024	Trauma; Spine Surgery	Machine Learning (ML); Deep Learning (DL)	Prognosis & Prediction
3391	10.1177/14604582241295930	2024	Unclassified	Natural Language Processing (NLP)	Prognosis & Prediction
3392	10.3390/geriatrics9050129	2024	Arthroplasty	Unclassified	Unclassified
3393	10.1016/j.jcot.2024.102561	2024	Unclassified	Machine Learning (ML); Deep Learning (DL)	Prognosis & Prediction
3394	10.3390/jcm13195961	2024	Trauma; Spine Surgery	Unclassified	Prognosis & Prediction
3395	10.3390/jcm13195971	2024	Trauma	Natural Language Processing (NLP)	Unclassified
3396	10.1007/s00432-024-05969-y	2024	Unclassified	Deep Learning (DL)	Diagnosis & Detection

3397	10.1093/dmfr/twae033	2024	Arthroplasty	Machine Learning (ML); Deep Learning (DL)	Diagnosis & Detection
3398	10.1016/j.jbo.2024.100640	2024	Unclassified	Deep Learning (DL)	Prognosis & Prediction
3399	10.1016/j.jbo.2024.100638	2024	Unclassified	Natural Language Processing (NLP); Machine Learning (ML); Deep Learning (DL)	Prognosis & Prediction
3400	10.1213/ANE.0000000000006832	2024	Spine Surgery	Machine Learning (ML); Deep Learning (DL)	Prognosis & Prediction
3401	10.1016/j.radi.2024.08.012	2024	Spine Surgery	Deep Learning (DL)	Prognosis & Prediction
3402	10.1007/s11547-024-01871-2	2024	Pediatric Orthopaedics	Machine Learning (ML)	Segmentation & Measurement
3403	10.1016/j.ejrad.2024.111663	2024	Arthroplasty	Deep Learning (DL)	Diagnosis & Detection
3404	10.1016/j.ejrad.2024.111636	2024	Spine Surgery	Unclassified	Diagnosis & Detection
3405	10.1053/j.ro.2024.07.004	2024	Unclassified	Natural Language Processing (NLP); Machine Learning (ML); Deep Learning (DL)	Prognosis & Prediction
3406	10.1016/j.bone.2024.117215	2024	Trauma	Deep Learning (DL)	Prognosis & Prediction
3407	10.1002/acr2.11709	2024	Trauma; Arthroplasty	Machine Learning (ML)	Prognosis & Prediction
3408	10.1007/s11282-024-00768-0	2024	Pediatric Orthopaedics; Arthroplasty	Deep Learning (DL)	Diagnosis & Detection
3409	10.1053/j.ro.2024.06.001	2024	Unclassified	Unclassified	Prognosis & Prediction
3410	10.1016/j.jcyt.2024.05.016	2024	Unclassified	Machine Learning (ML)	Prognosis & Prediction
3411	10.1016/j.jormas.2024.101914	2024	Trauma	Deep Learning (DL)	Diagnosis & Detection
3412	10.1016/j.diii.2024.05.001	2024	Unclassified	Deep Learning (DL)	Unclassified
3413	10.1007/s00330-024-10748-x	2024	Sports Medicine; Pediatric Orthopaedics; Arthroplasty	Deep Learning (DL)	Unclassified
3414	10.1002/jmri.29211	2024	Sports Medicine; Spine Surgery; Arthroplasty	Deep Learning (DL)	Prognosis & Prediction
3415	10.1007/s10278-024-01112-y	2024	Spine Surgery	Deep Learning (DL)	Unclassified
3416	10.1007/s10278-024-01089-8	2024	Sports Medicine; Arthroplasty	Deep Learning (DL)	Prognosis & Prediction
3417	10.21037/jmai-24-58	2024	Arthroplasty	Unclassified	Segmentation & Measurement
3418	10.1136/bmjopen-2024-086061	2024	Trauma; Spine Surgery	Unclassified	Prognosis & Prediction

3419	N/A	2024	Unclassified	Machine Learning (ML); Deep Learning (DL)	Diagnosis & Detection
3420	10.4274/haseki.galenos.2024.10038	2024	Trauma	Natural Language Processing (NLP)	Unclassified
3421	10.3348/jksr.2023.0111	2024	Sports Medicine; Spine Surgery	Deep Learning (DL)	Segmentation & Measurement
3422	10.3390/medicina60091396	2024	Arthroplasty	Deep Learning (DL)	Diagnosis & Detection
3423	10.3390/tomography10090109	2024	Arthroplasty	Machine Learning (ML); Deep Learning (DL)	Prognosis & Prediction
3424	10.3390/jcm13185575	2024	Trauma	Unclassified	Diagnosis & Detection
3425	10.3390/cancers16172988	2024	Trauma; Spine Surgery	Machine Learning (ML); Deep Learning (DL)	Prognosis & Prediction
3426	10.1016/j.bonr.2024.101805	2024	Trauma	Natural Language Processing (NLP); Machine Learning (ML); Deep Learning (DL)	Prognosis & Prediction
3427	10.3390/jcm13175180	2024	Arthroplasty	Machine Learning (ML)	Prognosis & Prediction
3428	10.3174/ajnr.A8335	2024	Trauma; Pediatric Orthopaedics	Deep Learning (DL)	Unclassified
3429	10.1093/mr/road115	2024	Unclassified	Natural Language Processing (NLP)	Prognosis & Prediction
3430	10.1152/ajprenal.00050.2024	2024	Unclassified	Machine Learning (ML)	Prognosis & Prediction
3431	10.1016/j.jbiomech.2024.112271	2024	Sports Medicine	Natural Language Processing (NLP); Robotics; Deep Learning (DL)	Prognosis & Prediction
3432	10.1177/02841851241263066	2024	Spine Surgery	Deep Learning (DL)	Prognosis & Prediction
3433	10.1016/j.artmed.2024.102935	2024	Trauma; Arthroplasty	Deep Learning (DL)	Prognosis & Prediction
3434	10.1016/j.ejrad.2024.111633	2024	Spine Surgery	Deep Learning (DL)	Diagnosis & Detection
3435	10.1007/s11547-024-01856-1	2024	Unclassified	Machine Learning (ML); Deep Learning (DL)	Prognosis & Prediction
3436	10.1016/j.imbio.2024.152825	2024	Sports Medicine; Arthroplasty	Machine Learning (ML)	Diagnosis & Detection
3437	10.1053/j.sart.2024.05.002	2024	Sports Medicine; Trauma; Arthroplasty	Unclassified	Segmentation & Measurement
3438	10.1016/j.ejrad.2024.111587	2024	Spine Surgery	Deep Learning (DL)	Segmentation & Measurement
3439	10.1016/j.ejrad.2024.111593	2024	Trauma	Natural Language Processing (NLP)	Diagnosis & Detection

3440	10.1016/j.modpat.2024.100542	2024	Unclassified	Unclassified	Diagnosis & Detection
3441	10.1002/art.42915	2024	Sports Medicine; Arthroplasty	Deep Learning (DL)	Prognosis & Prediction
3442	10.1016/j.bonr.2024.101787	2024	Unclassified	Machine Learning (ML)	Prognosis & Prediction
3443	10.1016/j.medntd.2024.100318	2024	Arthroplasty	Natural Language Processing (NLP); Deep Learning (DL)	Prognosis & Prediction
3444	10.1111/bcp.16112	2024	Arthroplasty	Machine Learning (ML)	Prognosis & Prediction
3445	10.1016/j.jacr.2023.11.029	2024	Trauma; Spine Surgery	Deep Learning (DL)	Prognosis & Prediction
3446	10.1002/med4.77	2024	Unclassified	Natural Language Processing (NLP)	Unclassified
3447	10.3390/prosthesis6040066	2024	Arthroplasty	Natural Language Processing (NLP); Robotics	Unclassified
3448	10.1093/jbmr/zjae089	2024	Trauma; Spine Surgery	Machine Learning (ML)	Prognosis & Prediction
3449	10.3390/cancers16152700	2024	Spine Surgery	Machine Learning (ML); Deep Learning (DL)	Prognosis & Prediction
3450	10.1016/j.jbiomech.2024.112252	2024	Unclassified	Machine Learning (ML); Deep Learning (DL)	Segmentation & Measurement
3451	10.21037/qims-22-540	2024	Spine Surgery	Deep Learning (DL)	Prognosis & Prediction
3452	10.21037/qims-23-1743	2024	Arthroplasty	Deep Learning (DL)	Diagnosis & Detection
3453	10.5858/arpa.2022-0533-OA	2024	Unclassified	Natural Language Processing (NLP); Deep Learning (DL)	Prognosis & Prediction
3454	10.1097/MAO.0000000000004246	2024	Unclassified	Machine Learning (ML); Deep Learning (DL)	Prognosis & Prediction
3455	10.1016/S2213-8587(24)00153-0	2024	Trauma; Spine Surgery	Machine Learning (ML)	Prognosis & Prediction
3456	10.1111/ggi.14923	2024	Unclassified	Machine Learning (ML)	Prognosis & Prediction
3457	10.1016/j.compbiomed.2024.108812	2024	Arthroplasty	Machine Learning (ML); Deep Learning (DL)	Prognosis & Prediction
3458	10.1016/j.urology.2024.05.027	2024	Arthroplasty	Machine Learning (ML)	Prognosis & Prediction
3459	10.1016/j.compbiomed.2024.108791	2024	Sports Medicine; Trauma	Deep Learning (DL)	Diagnosis & Detection
3460	10.1016/j.radonc.2024.110345	2024	Arthroplasty	Machine Learning (ML); Deep Learning (DL)	Prognosis & Prediction
3461	10.1111/1754-9485.13714	2024	Pediatric Orthopaedics	Deep Learning (DL)	Unclassified
3462	10.1016/j.clinimag.2024.110207	2024	Trauma	Machine Learning (ML)	Unclassified

3463	10.1111/jop.13548	2024	Unclassified	Machine Learning (ML); Deep Learning (DL)	Prognosis & Prediction
3464	10.1016/j.bone.2024.117131	2024	Pediatric Orthopaedics	Deep Learning (DL)	Diagnosis & Detection
3465	10.1016/j.crad.2024.04.009	2024	Trauma; Pediatric Orthopaedics	Deep Learning (DL)	Diagnosis & Detection
3466	10.1002/mp.17094	2024	Spine Surgery	Deep Learning (DL)	Diagnosis & Detection
3467	10.1115/1.4064550	2024	Arthroplasty	Deep Learning (DL)	Prognosis & Prediction
3468	10.1007/s00330-023-10559-6	2024	Trauma; Spine Surgery	Deep Learning (DL)	Prognosis & Prediction
3469	10.1007/s10278-024-01039-4	2024	Spine Surgery	Deep Learning (DL)	Prognosis & Prediction
3470	10.1055/a-2344-5398	2024	Sports Medicine; Pediatric Orthopaedics	Machine Learning (ML)	Surgical Planning
3471	10.1109/EMBC53108.2024.10781821	2024	Trauma; Pediatric Orthopaedics	Machine Learning (ML)	Prognosis & Prediction
3472	10.1177/14604582241270778	2024	Unclassified	Machine Learning (ML)	Diagnosis & Detection
3473	10.3390/jcm13144013	2024	Unclassified	Natural Language Processing (NLP)	Diagnosis & Detection
3474	10.22514/jocpd.2024.093	2024	Pediatric Orthopaedics; Spine Surgery	Deep Learning (DL)	Diagnosis & Detection
3475	10.1053/j.semnuclmed.2024.06.003	2024	Unclassified	Natural Language Processing (NLP); Machine Learning (ML); Deep Learning (DL)	Prognosis & Prediction
3476	10.3390/jcm13133850	2024	Trauma	Deep Learning (DL)	Diagnosis & Detection
3477	10.1302/0301-620X.106B7.BJJ-2024-0136	2024	Arthroplasty	Natural Language Processing (NLP)	Prognosis & Prediction
3478	10.1016/j.ejrad.2024.111496	2024	Trauma; Pediatric Orthopaedics	Deep Learning (DL)	Diagnosis & Detection
3479	10.1016/j.jhsg.2024.03.014	2024	Sports Medicine; Trauma	Natural Language Processing (NLP); Machine Learning (ML)	Prognosis & Prediction
3480	10.1002/acr.25327	2024	Arthroplasty	Machine Learning (ML)	Prognosis & Prediction
3481	10.1016/j.mri.2024.03.041	2024	Spine Surgery	Machine Learning (ML); Deep Learning (DL)	Surgical Planning
3482	10.1007/s00234-024-03330-1	2024	Unclassified	Deep Learning (DL)	Diagnosis & Detection
3483	10.1016/j.wnsx.2024.100338	2024	Trauma; Spine Surgery	Machine Learning (ML)	Prognosis & Prediction
3484	10.1007/s00259-024-06668-z	2024	Unclassified	Deep Learning (DL)	Prognosis & Prediction

3485	10.1007/s00330-023-10543-0	2024	Pediatric Orthopaedics	Unclassified	Segmentation & Measurement
3486	10.1007/s00330-023-10506-5	2024	Pediatric Orthopaedics	Deep Learning (DL)	Diagnosis & Detection
3487	10.1007/s00330-023-10473-x	2024	Trauma	Machine Learning (ML); Deep Learning (DL)	Prognosis & Prediction
3488	10.1016/j.oooo.2023.01.016	2024	Arthroplasty	Deep Learning (DL)	Prognosis & Prediction
3489	10.21037/jmai-24-3	2024	Arthroplasty	Natural Language Processing (NLP)	Prognosis & Prediction
3490	10.12968/hmed.2024.0034	2024	Trauma	Machine Learning (ML)	Prognosis & Prediction
3491	10.1097/MD.0000000000038503	2024	Pediatric Orthopaedics; Trauma; Arthroplasty	Machine Learning (ML)	Prognosis & Prediction
3492	10.61186/johe.13.3.182	2024	Unclassified	Machine Learning (ML); Deep Learning (DL)	Prognosis & Prediction
3493	10.34172/jrhs.2024.158	2024	Unclassified	Machine Learning (ML)	Prognosis & Prediction
3494	10.1371/journal.pdig.0000422	2024	Arthroplasty	Deep Learning (DL)	Prognosis & Prediction
3495	10.4067/s0717-95022024000300826	2024	Unclassified	Deep Learning (DL)	Prognosis & Prediction
3496	10.3390/biomedicines12061198	2024	Trauma	Deep Learning (DL)	Prognosis & Prediction
3497	10.3390/diagnostics14121263	2024	Pediatric Orthopaedics; Spine Surgery	Deep Learning (DL)	Prognosis & Prediction
3498	10.1200/CCI.24.00027	2024	Unclassified	Machine Learning (ML)	Prognosis & Prediction
3499	10.1016/j.jcot.2024.102470	2024	Arthroplasty	Machine Learning (ML)	Prognosis & Prediction
3500	10.1093/ckj/sfae143	2024	Sports Medicine	Machine Learning (ML)	Prognosis & Prediction
3501	10.1002/mp.17072	2024	Trauma; Spine Surgery	Deep Learning (DL)	Diagnosis & Detection
3502	10.1016/j.asmr.2024.100940	2024	Sports Medicine; Trauma	Deep Learning (DL)	Prognosis & Prediction
3503	10.1016/j.ejro.2024.100567	2024	Spine Surgery	Deep Learning (DL)	Segmentation & Measurement
3504	10.1016/j.compbiomed.2024.108502	2024	Unclassified	Deep Learning (DL)	Prognosis & Prediction
3505	10.1007/s00223-024-01208-1	2024	Trauma	Machine Learning (ML)	Prognosis & Prediction
3506	10.1002/acr2.11665	2024	Sports Medicine; Arthroplasty	Machine Learning (ML); Deep Learning (DL)	Diagnosis & Detection
3507	10.1016/j.mri.2024.03.022	2024	Spine Surgery; Arthroplasty	Deep Learning (DL)	Diagnosis & Detection

3508	10.1053/j.sart.2024.01.003	2024	Trauma; Arthroplasty	Natural Language Processing (NLP); Machine Learning (ML)	Prognosis & Prediction
3509	10.1002/acr2.11662	2024	Arthroplasty	Natural Language Processing (NLP)	Unclassified
3510	10.1016/j.semarthrit.2024.152420	2024	Arthroplasty	Deep Learning (DL)	Prognosis & Prediction
3511	10.1002/mp.17003	2024	Trauma	Machine Learning (ML); Deep Learning (DL)	Prognosis & Prediction
3512	10.1016/j.diii.2024.02.001	2024	Unclassified	Deep Learning (DL)	Diagnosis & Detection
3513	10.1016/j.bspc.2024.106099	2024	Spine Surgery	Deep Learning (DL)	Segmentation & Measurement
3514	10.1016/j.acra.2024.01.009	2024	Unclassified	Deep Learning (DL)	Diagnosis & Detection
3515	10.1007/s12306-023-00811-z	2024	Arthroplasty	Machine Learning (ML)	Prognosis & Prediction
3516	10.1007/s00405-023-08403-0	2024	Unclassified	Robotics; Deep Learning (DL)	Surgical Planning
3517	10.1177/19714009231224428	2024	Spine Surgery	Deep Learning (DL)	Unclassified
3518	10.1007/s00330-023-10394-9	2024	Trauma; Spine Surgery	Machine Learning (ML); Deep Learning (DL)	Prognosis & Prediction
3519	10.1055/a-2203-2997	2024	Pediatric Orthopaedics	Unclassified	Prognosis & Prediction
3520	10.57197/JDR-2024-0055	2024	Trauma	Machine Learning (ML)	Prognosis & Prediction
3521	10.1055/a-2305-2115	2024	Arthroplasty	Machine Learning (ML); Deep Learning (DL)	Prognosis & Prediction
3522	10.1088/1361-6560/ad3797	2024	Sports Medicine; Arthroplasty	Deep Learning (DL)	Segmentation & Measurement
3523	10.1148/ryai.230240	2024	Pediatric Orthopaedics	Deep Learning (DL)	Prognosis & Prediction
3524	10.3390/jpm14050495	2024	Unclassified	Natural Language Processing (NLP)	Unclassified
3525	10.3390/jcm13092573	2024	Pediatric Orthopaedics; Spine Surgery	Deep Learning (DL)	Prognosis & Prediction
3526	10.3390/cancers16091687	2024	Unclassified	Deep Learning (DL)	Diagnosis & Detection
3527	10.1097/MNM.0000000000001826	2024	Unclassified	Natural Language Processing (NLP); Machine Learning (ML)	Diagnosis & Detection
3528	10.1016/j.ejrad.2024.111399	2024	Trauma	Deep Learning (DL)	Prognosis & Prediction
3529	10.1016/j.bspc.2024.105943	2024	Trauma; Spine Surgery	Machine Learning (ML); Deep Learning (DL)	Prognosis & Prediction
3530	10.1007/s00414-023-03148-1	2024	Pediatric Orthopaedics; Arthroplasty	Machine Learning (ML); Deep Learning (DL)	Unclassified

3531	10.1002/acr.25260	2024	Unclassified	Natural Language Processing (NLP); Deep Learning (DL)	Diagnosis & Detection
3532	10.1016/j.acra.2023.10.042	2024	Trauma; Pediatric Orthopaedics	Deep Learning (DL)	Diagnosis & Detection
3533	10.1016/j.acra.2023.10.061	2024	Trauma; Spine Surgery	Machine Learning (ML); Deep Learning (DL)	Prognosis & Prediction
3534	10.1016/j.acra.2023.10.035	2024	Unclassified	Machine Learning (ML)	Prognosis & Prediction
3535	10.1007/s00330-023-10308-9	2024	Pediatric Orthopaedics; Spine Surgery	Deep Learning (DL)	Unclassified
3536	10.1016/j.zemedi.2023.01.008	2024	Unclassified	Machine Learning (ML); Deep Learning (DL)	Diagnosis & Detection
3537	10.21037/qims-23-1412	2024	Arthroplasty	Deep Learning (DL)	Diagnosis & Detection
3538	10.4274/jtss.galenos.2024.41736	2024	Pediatric Orthopaedics; Spine Surgery	Deep Learning (DL)	Prognosis & Prediction
3539	10.6065/apem.2346050.025	2024	Pediatric Orthopaedics	Deep Learning (DL)	Unclassified
3540	10.47391/JPMA.AKU-9S-06	2024	Unclassified	Machine Learning (ML); Deep Learning (DL)	Prognosis & Prediction
3541	10.47391/JPMA.AKU-9S-14	2024	Spine Surgery; Trauma; Arthroplasty	Machine Learning (ML)	Prognosis & Prediction
3542	10.1093/jbmr/zjae025	2024	Trauma	Machine Learning (ML); Deep Learning (DL)	Prognosis & Prediction
3543	10.12996/gmj.2024.4067	2024	Trauma	Natural Language Processing (NLP)	Unclassified
3544	10.3390/cancers16071357	2024	Unclassified	Machine Learning (ML)	Prognosis & Prediction
3545	10.1093/bjr/tqae026	2024	Spine Surgery	Deep Learning (DL)	Prognosis & Prediction
3546	10.1016/j.jhin.2023.09.024	2024	Spine Surgery	Machine Learning (ML)	Prognosis & Prediction
3547	10.1016/j.forsciint.2024.111973	2024	Sports Medicine; Trauma; Spine Surgery	Machine Learning (ML); Deep Learning (DL)	Prognosis & Prediction
3548	10.1016/j.compbiomed.2024.108082	2024	Unclassified	Natural Language Processing (NLP); Deep Learning (DL)	Prognosis & Prediction
3549	10.1016/j.jbo.2024.100593	2024	Unclassified	Machine Learning (ML); Deep Learning (DL)	Prognosis & Prediction
3550	10.1016/j.compbiomed.2024.108197	2024	Unclassified	Deep Learning (DL)	Prognosis & Prediction
3551	10.1002/mp.17000	2024	Arthroplasty	Deep Learning (DL)	Surgical Planning
3552	10.1016/j.ijmedinf.2024.105383	2024	Trauma; Spine Surgery	Machine Learning (ML); Deep Learning (DL)	Prognosis & Prediction
3553	10.1007/s11547-024-01791-1	2024	Spine Surgery	Deep Learning (DL)	Diagnosis & Detection

3554	10.1016/j.mri.2023.12.009	2024	Unclassified	Machine Learning (ML)	Prognosis & Prediction
3555	10.1007/s12020-023-03630-1	2024	Pediatric Orthopaedics	Unclassified	Prognosis & Prediction
3556	10.1007/s00259-023-06558-w	2024	Unclassified	Deep Learning (DL)	Unclassified
3557	10.1002/mp.16839	2024	Unclassified	Machine Learning (ML)	Diagnosis & Detection
3558	10.1177/17531934231209876	2024	Sports Medicine; Arthroplasty	Deep Learning (DL)	Segmentation & Measurement
3559	10.1016/j.jacr.2023.05.006	2024	Spine Surgery	Deep Learning (DL)	Prognosis & Prediction
3560	10.1002/jmri.28877	2024	Spine Surgery	Natural Language Processing (NLP); Deep Learning (DL)	Diagnosis & Detection
3561	10.1007/s10278-023-00945-3	2024	Unclassified	Machine Learning (ML); Deep Learning (DL)	Prognosis & Prediction
3562	10.1007/s10278-024-00968-4	2024	Trauma; Pediatric Orthopaedics	Deep Learning (DL)	Surgical Planning
3563	10.1097/MD.0000000000037458	2024	Trauma	Natural Language Processing (NLP)	Prognosis & Prediction
3564	10.4103/jmss.jmss_13_23	2024	Arthroplasty	Deep Learning (DL)	Surgical Planning
3565	10.14245/ns.2347052.526	2024	Spine Surgery	Natural Language Processing (NLP)	Unclassified
3566	10.1148/radiol.231429	2024	Trauma; Spine Surgery	Deep Learning (DL)	Diagnosis & Detection
3567	10.1093/ageing/afae045	2024	Trauma	Machine Learning (ML)	Prognosis & Prediction
3568	10.3390/jcm13051273	2024	Arthroplasty	Machine Learning (ML)	Prognosis & Prediction
3569	10.1016/j.nuclcard.2024.101809	2024	Unclassified	Deep Learning (DL)	Diagnosis & Detection
3570	10.1002/bdr2.2316	2024	Pediatric Orthopaedics; Arthroplasty	Machine Learning (ML); Deep Learning (DL)	Prognosis & Prediction
3571	10.1016/j.jbiomech.2024.112046	2024	Unclassified	Deep Learning (DL)	Segmentation & Measurement
3572	10.1016/j.jbiomech.2024.112012	2024	Spine Surgery	Deep Learning (DL)	Prognosis & Prediction
3573	10.1093/bjr/tqae014	2024	Trauma	Deep Learning (DL)	Prognosis & Prediction
3574	10.1016/j.jbiomech.2024.111967	2024	Sports Medicine; Trauma; Spine Surgery	Machine Learning (ML)	Prognosis & Prediction
3575	10.1016/j.bonr.2024.101743	2024	Sports Medicine; Trauma	Deep Learning (DL)	Diagnosis & Detection
3576	10.1016/j.ebiom.2024.105018	2024	Sports Medicine	Natural Language Processing (NLP); Machine Learning (ML)	Segmentation & Measurement

3577	10.2214/AJR.23.29530	2024	Unclassified	Unclassified	Diagnosis & Detection
3578	10.1007/s12194-023-00776-5	2024	Unclassified	Deep Learning (DL)	Diagnosis & Detection
3579	10.1111/trf.17735	2024	Arthroplasty	Machine Learning (ML)	Prognosis & Prediction
3580	10.1002/acm2.14282	2024	Spine Surgery	Deep Learning (DL)	Diagnosis & Detection
3581	10.1016/j.legalmed.2023.102383	2024	Unclassified	Machine Learning (ML); Deep Learning (DL)	Diagnosis & Detection
3582	10.1016/j.jhsg.2023.10.013	2024	Unclassified	Natural Language Processing (NLP)	Unclassified
3583	10.1016/j.bspc.2023.105788	2024	Pediatric Orthopaedics	Deep Learning (DL)	Diagnosis & Detection
3584	10.1016/j.rcl.2023.10.005	2024	Trauma; Spine Surgery	Machine Learning (ML); Deep Learning (DL)	Prognosis & Prediction
3585	10.1007/s11604-023-01494-z	2024	Pediatric Orthopaedics	Deep Learning (DL)	Prognosis & Prediction
3586	10.1016/j.diii.2023.09.008	2024	Pediatric Orthopaedics; Trauma; Arthroplasty	Natural Language Processing (NLP)	Prognosis & Prediction
3587	10.1016/j.acra.2023.08.030	2024	Spine Surgery	Machine Learning (ML); Deep Learning (DL)	Prognosis & Prediction
3588	10.1007/s12094-023-03291-6	2024	Arthroplasty	Deep Learning (DL)	Prognosis & Prediction
3589	10.1136/bmjopen-2023-079969	2024	Pediatric Orthopaedics	Deep Learning (DL)	Prognosis & Prediction
3590	10.1097/MD.0000000000036909	2024	Arthroplasty	Machine Learning (ML)	Prognosis & Prediction
3591	10.3389/fgene.2024.1255455	2024	Unclassified	Machine Learning (ML)	Prognosis & Prediction
3592	10.21037/qims-23-878	2024	Trauma	Machine Learning (ML); Deep Learning (DL)	Diagnosis & Detection
3593	10.23812/j.biol.regul.homeost.agents.20243802.106	2024	Unclassified	Machine Learning (ML); Deep Learning (DL)	Prognosis & Prediction
3594	10.1093/jbmr/zjad007	2024	Unclassified	Natural Language Processing (NLP)	Prognosis & Prediction
3595	10.1016/j.jcot.2024.102356	2024	Trauma	Unclassified	Prognosis & Prediction
3596	10.1002/lio2.1199	2024	Arthroplasty	Deep Learning (DL)	Prognosis & Prediction
3597	10.1016/j.artmed.2024.102776	2024	Arthroplasty	Natural Language Processing (NLP); Deep Learning (DL)	Segmentation & Measurement
3598	10.1007/s00432-023-05574-5	2024	Arthroplasty	Machine Learning (ML); Deep Learning (DL)	Prognosis & Prediction
3599	10.1167/iovs.65.2.5	2024	Arthroplasty	Deep Learning (DL)	Prognosis & Prediction

3600	10.1158/0008-5472.CAN-22-2654	2024	Unclassified	Machine Learning (ML); Deep Learning (DL)	Diagnosis & Detection
3601	10.1016/j.jbo.2024.100525	2024	Unclassified	Natural Language Processing (NLP)	Diagnosis & Detection
3602	10.1016/j.bspc.2023.105711	2024	Pediatric Orthopaedics; Spine Surgery	Deep Learning (DL)	Prognosis & Prediction
3603	10.1007/s11914-023-00853-z	2024	Trauma	Natural Language Processing (NLP)	Prognosis & Prediction
3604	10.1016/j.jhsa.2023.10.019	2024	Sports Medicine; Trauma	Natural Language Processing (NLP)	Unclassified
3605	10.1097/SLA.0000000000006008	2024	Arthroplasty	Natural Language Processing (NLP)	Unclassified
3606	10.1159/000529701	2024	Unclassified	Machine Learning (ML); Deep Learning (DL)	Prognosis & Prediction
3607	10.21037/qims-23-814	2024	Trauma	Deep Learning (DL)	Prognosis & Prediction
3608	10.21037/qims-23-715	2024	Pediatric Orthopaedics	Deep Learning (DL)	Prognosis & Prediction
3609	10.1136/bmjopen-2023-076954	2024	Trauma	Deep Learning (DL)	Prognosis & Prediction
3610	10.1182/bloodadvances.2023011076	2024	Unclassified	Deep Learning (DL)	Prognosis & Prediction
3611	10.3389/fmed.2024.1506686	2024	Trauma	Deep Learning (DL)	Diagnosis & Detection
3612	10.3389/fdgth.2024.1480381	2024	Arthroplasty	Natural Language Processing (NLP)	Unclassified
3613	10.3389/fbioe.2024.1437188	2024	Arthroplasty	Deep Learning (DL)	Prognosis & Prediction
3614	10.12809/hkjr2317736	2024	Pediatric Orthopaedics	Unclassified	Unclassified
3615	10.3389/fphys.2024.1445258	2024	Arthroplasty	Deep Learning (DL)	Prognosis & Prediction
3616	10.2147/JIR.S488841	2024	Sports Medicine; Arthroplasty	Machine Learning (ML)	Diagnosis & Detection
3617	10.2147/TCRM.S483907	2024	Trauma	Machine Learning (ML); Deep Learning (DL)	Prognosis & Prediction
3618	10.2196/59607	2024	Unclassified	Natural Language Processing (NLP)	Prognosis & Prediction
3619	10.1109/JBHI.2024.3491855	2024	Spine Surgery; Arthroplasty	Deep Learning (DL)	Diagnosis & Detection
3620	10.3389/fgene.2024.1492226	2024	Pediatric Orthopaedics; Spine Surgery	Machine Learning (ML); Deep Learning (DL)	Prognosis & Prediction
3621	10.1177/23312165241286456	2024	Unclassified	Machine Learning (ML)	Prognosis & Prediction
3622	10.2174/0115734056324977240911073841	2024	Unclassified	Natural Language Processing (NLP); Deep Learning (DL)	Segmentation & Measurement

3623	10.2196/50631	2024	Sports Medicine; Pediatric Orthopaedics; Spine Surgery	Deep Learning (DL)	Segmentation & Measurement
3624	10.1177/15330338241290386	2024	Pediatric Orthopaedics	Natural Language Processing (NLP); Machine Learning (ML)	Prognosis & Prediction
3625	10.1016/j.jvoice.2024.09.029	2024	Trauma	Machine Learning (ML)	Prognosis & Prediction
3626	10.3389/fmed.2024.1479187	2024	Trauma; Spine Surgery	Machine Learning (ML); Deep Learning (DL)	Prognosis & Prediction
3627	10.5624/isd.20240038	2024	Trauma	Deep Learning (DL)	Diagnosis & Detection
3628	10.2147/JIR.S488023	2024	Sports Medicine; Trauma; Arthroplasty	Machine Learning (ML)	Prognosis & Prediction
3629	10.3389/fradi.2024.1332535	2024	Sports Medicine	Machine Learning (ML); Deep Learning (DL)	Prognosis & Prediction
3630	10.1590/1806-9282.20240381	2024	Pediatric Orthopaedics; Arthroplasty	Machine Learning (ML); Deep Learning (DL)	Prognosis & Prediction
3631	10.1109/TNSRE.2024.3459924	2024	Arthroplasty	Machine Learning (ML); Robotics; Deep Learning (DL)	Segmentation & Measurement
3632	10.1177/20552076241279238	2024	Trauma	Unclassified	Diagnosis & Detection
3633	10.1590/1806-9282.20240523	2024	Trauma; Pediatric Orthopaedics	Deep Learning (DL)	Diagnosis & Detection
3634	10.3389/fpubh.2024.1414209	2024	Unclassified	Machine Learning (ML); Deep Learning (DL)	Prognosis & Prediction
3635	10.2196/57037	2024	Unclassified	Natural Language Processing (NLP)	Segmentation & Measurement
3636	10.3389/fbioe.2024.1442606	2024	Arthroplasty	Machine Learning (ML); Robotics; Deep Learning (DL)	Prognosis & Prediction
3637	10.1177/20552076241271778	2024	Unclassified	Deep Learning (DL)	Prognosis & Prediction
3638	10.1177/20552076241272620	2024	Arthroplasty	Natural Language Processing (NLP)	Diagnosis & Detection
3639	10.2196/51706	2024	Unclassified	Machine Learning (ML); Deep Learning (DL)	Prognosis & Prediction
3640	10.3389/fnut.2024.1422617	2024	Unclassified	Machine Learning (ML)	Prognosis & Prediction
3641	10.2147/JMDH.S477219	2024	Sports Medicine; Trauma; Arthroplasty	Unclassified	Surgical Planning
3642	10.1093/bjr/tqae109	2024	Trauma	Machine Learning (ML)	Prognosis & Prediction
3643	10.3389/fimmu.2024.1414301	2024	Arthroplasty	Machine Learning (ML)	Prognosis & Prediction

3644	10.2196/48535	2024	Trauma; Spine Surgery	Machine Learning (ML); Deep Learning (DL)	Prognosis & Prediction
3645	10.3389/fneur.2024.1386802	2024	Spine Surgery	Machine Learning (ML)	Prognosis & Prediction
3646	10.3389/fdgth.2024.1387139	2024	Trauma	Natural Language Processing (NLP)	Prognosis & Prediction
3647	10.3389/fmed.2024.1402768	2024	Unclassified	Machine Learning (ML); Deep Learning (DL)	Diagnosis & Detection
3648	10.2196/52001	2024	Spine Surgery	Natural Language Processing (NLP)	Prognosis & Prediction
3649	10.3389/fonc.2024.1298516	2024	Unclassified	Machine Learning (ML); Deep Learning (DL)	Prognosis & Prediction
3650	10.1080/07853890.2024.2357225	2024	Trauma	Machine Learning (ML)	Prognosis & Prediction
3651	10.3389/fendo.2024.1296047	2024	Unclassified	Natural Language Processing (NLP); Machine Learning (ML); Deep Learning (DL)	Prognosis & Prediction
3652	10.1590/0100-3984.2023.0102	2024	Trauma; Spine Surgery	Deep Learning (DL)	Prognosis & Prediction
3653	10.2174/0115734056295090240329051158	2024	Trauma; Spine Surgery	Machine Learning (ML); Deep Learning (DL)	Prognosis & Prediction
3654	10.3389/fmed.2024.1372495	2024	Unclassified	Machine Learning (ML)	Prognosis & Prediction
3655	10.3389/fbioe.2024.1397003	2024	Trauma; Spine Surgery	Deep Learning (DL)	Prognosis & Prediction
3656	10.1177/20552076241257456	2024	Trauma	Machine Learning (ML)	Prognosis & Prediction
3657	10.2174/0115734056289963240318040945	2024	Unclassified	Deep Learning (DL)	Prognosis & Prediction
3658	10.3389/fpubh.2024.1303958	2024	Trauma	Machine Learning (ML)	Prognosis & Prediction
3659	10.4082/kjfm.23.0186	2024	Trauma; Spine Surgery	Machine Learning (ML)	Prognosis & Prediction
3660	10.1177/20552076241253531	2024	Arthroplasty	Machine Learning (ML)	Prognosis & Prediction
3661	10.1093/dmfr/twae018	2024	Trauma	Machine Learning (ML); Deep Learning (DL)	Diagnosis & Detection
3662	10.2174/0115734056278130231218073650	2024	Pediatric Orthopaedics; Spine Surgery	Machine Learning (ML); Deep Learning (DL)	Diagnosis & Detection
3663	10.3389/fpubh.2024.1347219	2024	Unclassified	Machine Learning (ML); Deep Learning (DL)	Prognosis & Prediction

3664	10.5334/jbsr.3574	2024	Trauma	Unclassified	Diagnosis & Detection
3665	10.3389/fendo.2024.1370838	2024	Trauma; Spine Surgery	Machine Learning (ML); Deep Learning (DL)	Prognosis & Prediction
3666	10.3233/CBM-230219	2024	Spine Surgery	Deep Learning (DL)	Prognosis & Prediction
3667	10.1371/journal.pdig.0000438	2024	Trauma; Spine Surgery	Machine Learning (ML); Deep Learning (DL)	Prognosis & Prediction
3668	10.3389/fbioe.2024.1372669	2024	Pediatric Orthopaedics; Arthroplasty	Machine Learning (ML); Deep Learning (DL)	Prognosis & Prediction
3669	10.3389/fmed.2024.1297088	2024	Arthroplasty	Robotics; Deep Learning (DL)	Segmentation & Measurement
3670	10.2196/50882	2024	Unclassified	Natural Language Processing (NLP)	Diagnosis & Detection
3671	10.1109/TNSRE.2024.3371474	2024	Arthroplasty	Deep Learning (DL)	Diagnosis & Detection
3672	10.21037/tcr-23-1197	2024	Arthroplasty	Machine Learning (ML); Deep Learning (DL)	Prognosis & Prediction
3673	10.1080/21691401.2024.2318210	2024	Sports Medicine	Machine Learning (ML)	Diagnosis & Detection
3674	10.3389/fphys.2024.1329145	2024	Pediatric Orthopaedics	Deep Learning (DL)	Prognosis & Prediction
3675	10.3389/fpubh.2024.1348236	2024	Arthroplasty	Unclassified	Unclassified
3676	10.1109/TNSRE.2024.3365201	2024	Arthroplasty	Machine Learning (ML); Deep Learning (DL)	Prognosis & Prediction
3677	10.2174/0115734056281578231212104108	2024	Unclassified	Machine Learning (ML); Deep Learning (DL)	Prognosis & Prediction
3678	10.1177/11769351231223806	2024	Arthroplasty	Machine Learning (ML); Deep Learning (DL)	Diagnosis & Detection
3679	10.1148/radiol.230614	2024	Trauma	Deep Learning (DL)	Prognosis & Prediction
3680	10.3390/jcm13020603	2024	Arthroplasty	Machine Learning (ML); Deep Learning (DL)	Prognosis & Prediction
3681	10.3390/biomedicines12010076	2024	Unclassified	Machine Learning (ML)	Prognosis & Prediction
3682	10.3390/jpm14010069	2024	Arthroplasty	Natural Language Processing (NLP)	Prognosis & Prediction
3683	10.1016/j.jbiomech.2024.111952	2024	Arthroplasty	Deep Learning (DL)	Prognosis & Prediction
3684	10.1177/03000605231223881	2024	Spine Surgery	Machine Learning (ML); Deep Learning (DL)	Diagnosis & Detection

3685	10.1007/s00247-023-05822-3	2024	Pediatric Orthopaedics; Trauma; Arthroplasty	Natural Language Processing (NLP)	Prognosis & Prediction
3686	10.2174/0115734056262464230922112606	2024	Arthroplasty	Machine Learning (ML); Deep Learning (DL)	Prognosis & Prediction
3687	10.1016/j.cmpb.2023.107924	2024	Unclassified	Deep Learning (DL)	Prognosis & Prediction
3688	10.1016/j.compbiomed.2023.107704	2024	Trauma	Deep Learning (DL)	Prognosis & Prediction
3689	10.1016/j.mri.2023.11.003	2024	Spine Surgery	Deep Learning (DL)	Unclassified
3690	10.1007/s00247-023-05789-1	2024	Pediatric Orthopaedics	Natural Language Processing (NLP); Deep Learning (DL)	Diagnosis & Detection
3691	10.1002/ima.22973	2024	Unclassified	Deep Learning (DL)	Prognosis & Prediction
3692	10.1016/j.bspc.2023.105447	2024	Arthroplasty	Deep Learning (DL)	Unclassified
3693	10.1080/21681163.2023.2261554	2024	Sports Medicine; Trauma; Arthroplasty	Machine Learning (ML); Deep Learning (DL)	Diagnosis & Detection
3694	10.1007/s00198-023-06913-5	2024	Trauma; Spine Surgery	Deep Learning (DL)	Prognosis & Prediction
3695	10.1007/s00198-023-06903-7	2024	Trauma; Spine Surgery	Natural Language Processing (NLP); Deep Learning (DL)	Prognosis & Prediction
3696	10.1007/s00198-023-06900-w	2024	Unclassified	Deep Learning (DL)	Prognosis & Prediction
3697	10.1016/j.diii.2023.07.001	2024	Pediatric Orthopaedics	Deep Learning (DL)	Diagnosis & Detection
3698	10.1067/j.cpradiol.2023.04.001	2024	Trauma	Natural Language Processing (NLP)	Unclassified
3699	10.1093/bjro/tzae011	2024	Trauma	Unclassified	Diagnosis & Detection
3700	10.1093/bjro/tzad005	2024	Trauma; Pediatric Orthopaedics	Machine Learning (ML)	Diagnosis & Detection
3701	10.4103/jdmimsu.jdmimsu_436_21	2023	Trauma	Deep Learning (DL)	Prognosis & Prediction
3702	10.1097/OI9.0000000000000283	2023	Trauma	Machine Learning (ML); Deep Learning (DL)	Prognosis & Prediction
3703	10.1097/OI9.0000000000000284	2023	Trauma	Machine Learning (ML); Deep Learning (DL)	Prognosis & Prediction
3704	10.1136/bmjopen-2022-071430	2023	Trauma	Machine Learning (ML)	Prognosis & Prediction
3705	N/A	2023	Unclassified	Machine Learning (ML); Deep Learning (DL)	Prognosis & Prediction
3706	10.3390/jpm13121695	2023	Pediatric Orthopaedics	Natural Language Processing (NLP)	Prognosis & Prediction

3707	10.3390/medicina59122125	2023	Trauma	Unclassified	Prognosis & Prediction
3708	10.3390/jcm12247730	2023	Trauma; Spine Surgery	Unclassified	Prognosis & Prediction
3709	10.3390/jimaging9120265	2023	Spine Surgery	Machine Learning (ML); Deep Learning (DL)	Diagnosis & Detection
3710	10.1016/j.jcot.2023.102312	2023	Arthroplasty	Machine Learning (ML); Deep Learning (DL)	Prognosis & Prediction
3711	10.3390/jcm12237382	2023	Spine Surgery	Unclassified	Prognosis & Prediction
3712	10.1186/s12871-023-02354-z	2023	Arthroplasty	Machine Learning (ML)	Prognosis & Prediction
3713	10.1186/s12880-023-01156-6	2023	Sports Medicine; Spine Surgery	Deep Learning (DL)	Diagnosis & Detection
3714	10.1016/j.rcot.2023.10.014	2023	Trauma	Natural Language Processing (NLP)	Unclassified
3715	10.1002/art.42650	2023	Arthroplasty	Machine Learning (ML); Deep Learning (DL)	Prognosis & Prediction
3716	10.1016/j.neuri.2023.100143	2023	Spine Surgery	Machine Learning (ML)	Prognosis & Prediction
3717	10.1016/j.rcot.2023.09.014	2023	Trauma	Natural Language Processing (NLP)	Unclassified
3718	10.1002/jbmr.4921	2023	Trauma; Spine Surgery	Machine Learning (ML)	Prognosis & Prediction
3719	10.1007/s00432-023-05472-w	2023	Unclassified	Machine Learning (ML)	Prognosis & Prediction
3720	10.1002/jcsm.13356	2023	Arthroplasty	Machine Learning (ML); Deep Learning (DL)	Prognosis & Prediction
3721	10.1002/jbm4.10828	2023	Trauma; Spine Surgery	Deep Learning (DL)	Prognosis & Prediction
3722	10.1186/s12891-023-06939-0	2023	Trauma; Spine Surgery	Machine Learning (ML); Deep Learning (DL)	Diagnosis & Detection
3723	10.1002/jbmr.4916	2023	Trauma; Spine Surgery	Natural Language Processing (NLP); Machine Learning (ML); Deep Learning (DL)	Diagnosis & Detection
3724	10.1186/s12920-023-01672-y	2023	Sports Medicine; Arthroplasty	Machine Learning (ML)	Diagnosis & Detection
3725	10.1007/s12149-023-01872-7	2023	Unclassified	Deep Learning (DL)	Unclassified
3726	10.1016/j.health.2023.100265	2023	Sports Medicine; Spine Surgery; Trauma; Arthroplasty	Deep Learning (DL)	Diagnosis & Detection
3727	10.1007/s00432-023-05384-9	2023	Unclassified	Deep Learning (DL)	Diagnosis & Detection

3728	10.1186/s13756-023-01294-0	2023	Pediatric Orthopaedics; Arthroplasty	Natural Language Processing (NLP); Machine Learning (ML)	Prognosis & Prediction
3729	10.1186/s12920-023-01601-z	2023	Unclassified	Machine Learning (ML)	Prognosis & Prediction
3730	10.1007/s00330-023-09918-0	2023	Spine Surgery	Deep Learning (DL)	Diagnosis & Detection
3731	10.1016/j.archger.2023.105120	2023	Trauma; Arthroplasty	Machine Learning (ML)	Prognosis & Prediction
3732	10.1016/j.jfma.2023.06.027	2023	Spine Surgery	Machine Learning (ML)	Prognosis & Prediction
3733	10.1016/j.acra.2023.04.023	2023	Trauma; Spine Surgery	Natural Language Processing (NLP); Deep Learning (DL)	Prognosis & Prediction
3734	10.1016/j.ejro.2023.100504	2023	Trauma	Deep Learning (DL)	Prognosis & Prediction
3735	10.1186/s12938-023-01132-9	2023	Trauma	Machine Learning (ML)	Prognosis & Prediction
3736	10.1002/jmri.28735	2023	Pediatric Orthopaedics; Spine Surgery	Deep Learning (DL)	Diagnosis & Detection
3737	10.1186/s12880-023-01012-7	2023	Trauma; Spine Surgery	Deep Learning (DL)	Prognosis & Prediction
3738	10.1186/s13244-023-01385-x	2023	Unclassified	Deep Learning (DL)	Prognosis & Prediction
3739	10.1186/s13075-023-03008-8	2023	Arthroplasty	Machine Learning (ML)	Unclassified
3740	10.1186/s12880-023-00975-x	2023	Trauma; Arthroplasty	Deep Learning (DL)	Diagnosis & Detection
3741	10.1186/s40644-023-00524-3	2023	Unclassified	Machine Learning (ML); Deep Learning (DL)	Prognosis & Prediction
3742	10.1007/s42399-023-01608-8	2023	Trauma; Pediatric Orthopaedics	Machine Learning (ML); Deep Learning (DL)	Prognosis & Prediction
3743	10.1088/1361-6560/ad04aa	2023	Sports Medicine; Arthroplasty	Deep Learning (DL)	Prognosis & Prediction
3744	10.1097/MD.0000000000035355	2023	Arthroplasty	Machine Learning (ML)	Prognosis & Prediction
3745	10.3390/jcm12227189	2023	Unclassified	Machine Learning (ML); Deep Learning (DL)	Diagnosis & Detection
3746	10.3348/kjr.2023.0092	2023	Pediatric Orthopaedics	Deep Learning (DL)	Prognosis & Prediction
3747	10.3349/ymj.2023.0244	2023	Pediatric Orthopaedics	Deep Learning (DL)	Prognosis & Prediction
3748	10.1007/s00774-023-01469-0	2023	Spine Surgery	Machine Learning (ML)	Prognosis & Prediction
3749	10.1016/j.cegh.2023.101438	2023	Unclassified	Machine Learning (ML)	Prognosis & Prediction

3750	10.1002/acm2.14166	2023	Spine Surgery	Deep Learning (DL)	Diagnosis & Detection
3751	10.1007/s00247-023-05754-y	2023	Trauma; Pediatric Orthopaedics	Natural Language Processing (NLP); Deep Learning (DL)	Diagnosis & Detection
3752	10.1007/s40520-023-02550-4	2023	Trauma	Machine Learning (ML)	Prognosis & Prediction
3753	10.1016/j.bone.2023.116865	2023	Trauma	Machine Learning (ML)	Prognosis & Prediction
3754	10.1109/JBHI.2023.3304639	2023	Arthroplasty	Robotics; Deep Learning (DL)	Prognosis & Prediction
3755	10.1016/j.cmpb.2023.107737	2023	Arthroplasty	Deep Learning (DL)	Prognosis & Prediction
3756	10.1007/s11926-023-01114-9	2023	Arthroplasty	Machine Learning (ML)	Prognosis & Prediction
3757	10.1007/s00414-023-03072-4	2023	Pediatric Orthopaedics	Machine Learning (ML); Deep Learning (DL)	Prognosis & Prediction
3758	10.1016/j.ajic.2023.04.165	2023	Arthroplasty	Natural Language Processing (NLP); Machine Learning (ML)	Prognosis & Prediction
3759	10.1007/s00330-023-09704-y	2023	Pediatric Orthopaedics; Arthroplasty	Deep Learning (DL)	Prognosis & Prediction
3760	10.1007/s00330-023-09726-6	2023	Unclassified	Deep Learning (DL)	Diagnosis & Detection
3761	10.1016/j.health.2023.100153	2023	Unclassified	Deep Learning (DL)	Prognosis & Prediction
3762	10.1136/bmjopen-2023-074700	2023	Trauma; Arthroplasty	Machine Learning (ML); Deep Learning (DL)	Prognosis & Prediction
3763	N/A	2023	Unclassified	Machine Learning (ML)	Unclassified
3764	10.3390/jcm12206655	2023	Arthroplasty	Natural Language Processing (NLP)	Prognosis & Prediction
3765	10.3899/jrheum.2022-1039	2023	Unclassified	Machine Learning (ML)	Prognosis & Prediction
3766	10.21037/qims-23-428	2023	Trauma; Arthroplasty	Deep Learning (DL)	Diagnosis & Detection
3767	10.1097/RLI.0000000000000986	2023	Unclassified	Machine Learning (ML); Deep Learning (DL)	Prognosis & Prediction
3768	10.1093/postmj/qgad053	2023	Trauma	Natural Language Processing (NLP)	Unclassified
3769	10.1016/j.compbiomed.2023.107391	2023	Arthroplasty	Deep Learning (DL)	Unclassified
3770	10.1002/cai2.92	2023	Pediatric Orthopaedics	Machine Learning (ML)	Prognosis & Prediction
3771	10.1016/j.rhum.2023.07.018	2023	Sports Medicine; Trauma; Spine Surgery	Machine Learning (ML); Deep Learning (DL)	Diagnosis & Detection
3772	10.1109/JBHI.2023.3305377	2023	Arthroplasty	Deep Learning (DL)	Segmentation & Measurement

3773	10.1007/s00259-023-06339-5	2023	Unclassified	Natural Language Processing (NLP); Deep Learning (DL)	Prognosis & Prediction
3774	10.1007/s10278-023-00880-3	2023	Unclassified	Deep Learning (DL)	Diagnosis & Detection
3775	10.1016/j.acra.2023.06.016	2023	Trauma	Deep Learning (DL)	Diagnosis & Detection
3776	10.1002/mrm.29710	2023	Arthroplasty	Deep Learning (DL)	Segmentation & Measurement
3777	10.1007/s10278-023-00849-2	2023	Pediatric Orthopaedics	Deep Learning (DL)	Prognosis & Prediction
3778	10.1007/s00330-023-09687-w	2023	Arthroplasty	Deep Learning (DL)	Diagnosis & Detection
3779	10.1016/j.rcot.2023.01.021	2023	Trauma	Unclassified	Unclassified
3780	10.1088/1361-6560/acf2e6	2023	Arthroplasty	Deep Learning (DL)	Diagnosis & Detection
3781	10.3390/biomedinformatics3030048	2023	Unclassified	Natural Language Processing (NLP); Machine Learning (ML)	Prognosis & Prediction
3782	10.3390/biomedinformatics3030046	2023	Arthroplasty	Deep Learning (DL)	Diagnosis & Detection
3783	10.1117/1.JMI.10.5.051810	2023	Spine Surgery; Arthroplasty	Deep Learning (DL)	Diagnosis & Detection
3784	10.5152/iao.2023.231073	2023	Arthroplasty	Deep Learning (DL)	Diagnosis & Detection
3785	10.3390/jcm12185960	2023	Pediatric Orthopaedics	Deep Learning (DL)	Prognosis & Prediction
3786	10.3390/jimaging9090187	2023	Trauma; Spine Surgery	Deep Learning (DL)	Diagnosis & Detection
3787	10.1016/j.bspc.2023.105372	2023	Trauma; Arthroplasty	Deep Learning (DL)	Prognosis & Prediction
3788	10.1016/j.compbiomed.2023.107360	2023	Sports Medicine; Trauma; Arthroplasty	Machine Learning (ML); Deep Learning (DL)	Prognosis & Prediction
3789	10.1016/j.ejmp.2023.102657	2023	Spine Surgery	Machine Learning (ML)	Diagnosis & Detection
3790	10.1002/jbmr.4879	2023	Trauma; Spine Surgery	Deep Learning (DL)	Prognosis & Prediction
3791	10.1016/j.cyto.2023.156300	2023	Sports Medicine; Arthroplasty	Machine Learning (ML)	Prognosis & Prediction
3792	10.1515/oncologie-2023-0115	2023	Unclassified	Deep Learning (DL)	Prognosis & Prediction
3793	10.1016/j.bspc.2023.105103	2023	Arthroplasty	Robotics; Deep Learning (DL)	Unclassified
3794	10.1016/j.bspc.2023.105153	2023	Trauma; Spine Surgery	Deep Learning (DL)	Segmentation & Measurement
3795	10.1016/j.compbiomed.2023.107137	2023	Arthroplasty	Machine Learning (ML); Deep Learning (DL)	Prognosis & Prediction

3796	10.1016/j.compbiomed.2023.107124	2023	Arthroplasty	Machine Learning (ML); Deep Learning (DL)	Prognosis & Prediction
3797	10.1016/j.jclinane.2023.111147	2023	Arthroplasty	Machine Learning (ML)	Prognosis & Prediction
3798	10.1007/s00330-023-09573-5	2023	Arthroplasty	Deep Learning (DL)	Prognosis & Prediction
3799	10.1016/j.xops.2023.100292	2023	Arthroplasty	Deep Learning (DL)	Prognosis & Prediction
3800	10.1001/jamanetworkopen.2023.30617	2023	Pediatric Orthopaedics; Spine Surgery	Deep Learning (DL)	Prognosis & Prediction
3801	10.1136/bmjopen-2022-071324	2023	Trauma	Natural Language Processing (NLP)	Diagnosis & Detection
3802	10.12968/hmed.2023.0258	2023	Trauma; Arthroplasty	Machine Learning (ML); Deep Learning (DL)	Prognosis & Prediction
3803	10.11005/jbm.2023.30.3.245	2023	Trauma	Machine Learning (ML)	Prognosis & Prediction
3804	10.23736/S1973-9087.23.08091-7	2023	Sports Medicine; Pediatric Orthopaedics	Unclassified	Diagnosis & Detection
3805	10.1302/2046-3758.128.BJR2023-0199	2023	Unclassified	Natural Language Processing (NLP); Machine Learning (ML); Robotics	Unclassified
3806	10.4067/S0717-95022023000401267	2023	Unclassified	Machine Learning (ML)	Prognosis & Prediction
3807	10.3390/diagnostics13162658	2023	Spine Surgery	Deep Learning (DL)	Segmentation & Measurement
3808	10.21037/qims-22-1251	2023	Sports Medicine; Arthroplasty	Machine Learning (ML); Deep Learning (DL)	Prognosis & Prediction
3809	10.3174/ajnr.A7920	2023	Trauma; Spine Surgery	Deep Learning (DL)	Unclassified
3810	10.1016/j.ebiom.2023.104676	2023	Sports Medicine; Trauma; Spine Surgery	Machine Learning (ML); Deep Learning (DL)	Prognosis & Prediction
3811	10.1093/pm/pnad035	2023	Spine Surgery	Deep Learning (DL)	Segmentation & Measurement
3812	10.1093/rheumatology/keac707	2023	Arthroplasty	Machine Learning (ML)	Diagnosis & Detection
3813	10.1093/rheumatology/keac723	2023	Arthroplasty	Unclassified	Prognosis & Prediction
3814	10.1136/bmjopen-2022-067036	2023	Sports Medicine; Arthroplasty	Machine Learning (ML)	Prognosis & Prediction
3815	10.1016/j.joms.2023.04.013	2023	Trauma	Deep Learning (DL)	Diagnosis & Detection
3816	10.1002/jbm4.10778	2023	Trauma; Spine Surgery	Machine Learning (ML)	Prognosis & Prediction

3817	10.1016/j.ejrad.2023.110899	2023	Trauma; Spine Surgery	Deep Learning (DL)	Prognosis & Prediction
3818	10.1007/s10278-023-00847-4	2023	Trauma; Spine Surgery	Deep Learning (DL)	Unclassified
3819	10.1016/j.bspc.2023.105030	2023	Arthroplasty	Deep Learning (DL)	Unclassified
3820	10.1007/s00198-023-06787-7	2023	Trauma; Spine Surgery	Machine Learning (ML)	Prognosis & Prediction
3821	10.1007/s10278-023-00830-z	2023	Sports Medicine; Spine Surgery	Deep Learning (DL)	Surgical Planning
3822	10.1016/j.jcmg.2023.01.014	2023	Unclassified	Machine Learning (ML); Deep Learning (DL)	Prognosis & Prediction
3823	10.1007/s10278-023-00829-6	2023	Trauma	Deep Learning (DL)	Prognosis & Prediction
3824	10.1007/s11604-023-01413-2	2023	Unclassified	Natural Language Processing (NLP); Deep Learning (DL)	Diagnosis & Detection
3825	10.1007/s10278-023-00793-1	2023	Trauma; Pediatric Orthopaedics	Deep Learning (DL)	Diagnosis & Detection
3826	10.1093/pm/pnac142	2023	Spine Surgery	Deep Learning (DL)	Segmentation & Measurement
3827	10.1002/jmri.28573	2023	Sports Medicine; Arthroplasty	Deep Learning (DL)	Prognosis & Prediction
3828	10.1088/1361-6560/acddc6	2023	Unclassified	Deep Learning (DL)	Diagnosis & Detection
3829	10.4103/jcvjs.jcvjs_69_23	2023	Spine Surgery	Machine Learning (ML); Deep Learning (DL)	Prognosis & Prediction
3830	10.1302/2046-3758.127.BJR2023-0111.R1	2023	Sports Medicine; Trauma; Arthroplasty	Natural Language Processing (NLP); Machine Learning (ML); Deep Learning (DL)	Prognosis & Prediction
3831	10.1016/j.eclinm.2023.102050	2023	Pediatric Orthopaedics; Spine Surgery	Deep Learning (DL)	Prognosis & Prediction
3832	10.1148/ryai.220158	2023	Sports Medicine; Pediatric Orthopaedics; Spine Surgery	Machine Learning (ML); Deep Learning (DL)	Surgical Planning
3833	10.1007/s40520-023-02428-5	2023	Arthroplasty	Machine Learning (ML); Deep Learning (DL)	Diagnosis & Detection
3834	10.1007/s00330-023-09713-x	2023	Sports Medicine; Trauma; Spine Surgery	Deep Learning (DL)	Unclassified
3835	10.1007/s00330-023-09678-x	2023	Trauma; Spine Surgery	Machine Learning (ML)	Prognosis & Prediction
3836	10.1016/j.diii.2023.04.003	2023	Pediatric Orthopaedics	Unclassified	Unclassified

3837	10.1016/j.forsciint.2023.111704	2023	Unclassified	Deep Learning (DL)	Segmentation & Measurement
3838	10.1016/j.bspc.2023.104713	2023	Arthroplasty	Machine Learning (ML); Deep Learning (DL)	Prognosis & Prediction
3839	10.1007/s00247-023-05621-w	2023	Sports Medicine; Trauma; Pediatric Orthopaedics	Deep Learning (DL)	Prognosis & Prediction
3840	10.1016/j.jhqr.2022.11.009	2023	Arthroplasty	Machine Learning (ML); Deep Learning (DL)	Prognosis & Prediction
3841	10.1136/postgradmedj-2022-141596	2023	Sports Medicine; Trauma	Natural Language Processing (NLP); Machine Learning (ML); Robotics; Deep Learning (DL)	Prognosis & Prediction
3842	10.1097/MD.0000000000034113	2023	Trauma; Arthroplasty	Unclassified	Prognosis & Prediction
3843	10.1097/MD.0000000000034071	2023	Spine Surgery; Arthroplasty	Unclassified	Diagnosis & Detection
3844	10.1088/1361-6560/acd48b	2023	Trauma; Spine Surgery	Natural Language Processing (NLP); Deep Learning (DL)	Diagnosis & Detection
3845	10.13104/imri.2022.1102	2023	Unclassified	Deep Learning (DL)	Unclassified
3846	10.21037/qims-22-1250	2023	Arthroplasty	Deep Learning (DL)	Diagnosis & Detection
3847	10.1001/jamanetworkopen.2023.20400	2023	Unclassified	Unclassified	Prognosis & Prediction
3848	10.1016/j.jbiomech.2023.111668	2023	Arthroplasty	Machine Learning (ML); Deep Learning (DL)	Prognosis & Prediction
3849	10.1259/bjr.20221006	2023	Trauma	Deep Learning (DL)	Diagnosis & Detection
3850	10.1016/j.jbo.2023.100483	2023	Spine Surgery	Deep Learning (DL)	Prognosis & Prediction
3851	10.1016/j.compbiomed.2023.106839	2023	Spine Surgery	Natural Language Processing (NLP); Deep Learning (DL)	Diagnosis & Detection
3852	10.1002/jbmr.4814	2023	Trauma; Spine Surgery	Deep Learning (DL)	Prognosis & Prediction
3853	10.1007/s13246-023-01256-1	2023	Unclassified	Natural Language Processing (NLP); Deep Learning (DL)	Prognosis & Prediction
3854	10.1007/s13246-023-01261-4	2023	Trauma; Arthroplasty	Deep Learning (DL)	Diagnosis & Detection
3855	10.1016/j.ejrad.2023.110844	2023	Arthroplasty	Deep Learning (DL)	Segmentation & Measurement
3856	10.1002/acm2.13996	2023	Spine Surgery	Deep Learning (DL)	Diagnosis & Detection
3857	10.1002/jbmr.4805	2023	Trauma	Machine Learning (ML); Robotics	Prognosis & Prediction

3858	10.1007/s40520-023-02399-7	2023	Arthroplasty	Machine Learning (ML)	Prognosis & Prediction
3859	10.1109/JBHI.2023.3262164	2023	Arthroplasty	Machine Learning (ML); Deep Learning (DL)	Prognosis & Prediction
3860	10.1016/j.bone.2023.116743	2023	Sports Medicine	Deep Learning (DL)	Diagnosis & Detection
3861	10.1016/j.crad.2023.02.012	2023	Unclassified	Machine Learning (ML)	Prognosis & Prediction
3862	10.1007/s10278-023-00788-y	2023	Unclassified	Machine Learning (ML); Deep Learning (DL)	Segmentation & Measurement
3863	10.1007/s40336-023-00539-7	2023	Unclassified	Machine Learning (ML); Deep Learning (DL)	Diagnosis & Detection
3864	10.1007/s00330-022-09349-3	2023	Trauma	Deep Learning (DL)	Prognosis & Prediction
3865	10.1007/s00330-022-09289-y	2023	Unclassified	Deep Learning (DL)	Diagnosis & Detection
3866	10.1001/jamanetworkopen.2023.15174	2023	Pediatric Orthopaedics; Arthroplasty	Natural Language Processing (NLP); Deep Learning (DL)	Prognosis & Prediction
3867	10.1021/cbmi.3c00035	2023	Arthroplasty	Machine Learning (ML)	Diagnosis & Detection
3868	10.3233/THC-220501	2023	Trauma	Deep Learning (DL)	Diagnosis & Detection
3869	10.1088/1361-6560/aca740	2023	Arthroplasty	Machine Learning (ML); Deep Learning (DL)	Diagnosis & Detection
3870	10.21037/jmai-23-10	2023	Unclassified	Deep Learning (DL)	Unclassified
3871	10.5152/iao.2023.22958	2023	Unclassified	Machine Learning (ML); Deep Learning (DL)	Prognosis & Prediction
3872	10.1007/s00247-023-05588-8	2023	Trauma; Pediatric Orthopaedics	Deep Learning (DL)	Prognosis & Prediction
3873	10.1097/RCT.0000000000001437	2023	Spine Surgery	Deep Learning (DL)	Prognosis & Prediction
3874	10.3390/healthcare11091206	2023	Unclassified	Machine Learning (ML); Deep Learning (DL)	Prognosis & Prediction
3875	10.1016/j.cmpb.2023.107465	2023	Arthroplasty	Deep Learning (DL)	Prognosis & Prediction
3876	10.1016/j.cmpb.2023.107464	2023	Trauma	Machine Learning (ML); Deep Learning (DL)	Prognosis & Prediction
3877	10.1007/s00247-023-05590-0	2023	Trauma; Pediatric Orthopaedics	Deep Learning (DL)	Diagnosis & Detection
3878	10.1007/s00247-022-05569-3	2023	Pediatric Orthopaedics	Unclassified	Prognosis & Prediction

3879	10.1007/s00330-022-09354-6	2023	Sports Medicine; Trauma; Spine Surgery	Deep Learning (DL)	Diagnosis & Detection
3880	10.1007/s00330-022-09330-0	2023	Pediatric Orthopaedics; Arthroplasty	Deep Learning (DL)	Prognosis & Prediction
3881	10.1177/17531934221143434	2023	Trauma	Deep Learning (DL)	Unclassified
3882	10.1016/j.neurad.2022.08.001	2023	Trauma; Spine Surgery	Machine Learning (ML)	Prognosis & Prediction
3883	10.1182/blood.2022017518	2023	Pediatric Orthopaedics	Machine Learning (ML)	Prognosis & Prediction
3884	10.1097/MD.0000000000033125	2023	Unclassified	Machine Learning (ML); Deep Learning (DL)	Diagnosis & Detection
3885	10.1097/MD.0000000000033441	2023	Pediatric Orthopaedics; Spine Surgery	Machine Learning (ML); Deep Learning (DL)	Prognosis & Prediction
3886	10.5812/iranjradiol-136311	2023	Pediatric Orthopaedics; Arthroplasty	Deep Learning (DL)	Prognosis & Prediction
3887	10.3390/medicina59040782	2023	Arthroplasty	Machine Learning (ML)	Prognosis & Prediction
3888	10.3390/ijerph20085575	2023	Unclassified	Machine Learning (ML)	Prognosis & Prediction
3889	10.1259/bjr.20220778	2023	Trauma; Pediatric Orthopaedics	Deep Learning (DL)	Prognosis & Prediction
3890	10.1167/tvst.12.4.7	2023	Sports Medicine; Trauma	Machine Learning (ML); Deep Learning (DL)	Diagnosis & Detection
3891	10.1053/j.ro.2023.03.002	2023	Trauma	Unclassified	Unclassified
3892	10.1097/RLI.0000000000000932	2023	Spine Surgery	Machine Learning (ML); Deep Learning (DL)	Prognosis & Prediction
3893	10.1016/j.cmpb.2023.107343	2023	Unclassified	Deep Learning (DL)	Prognosis & Prediction
3894	10.1016/j.ejrad.2023.110726	2023	Trauma; Spine Surgery	Machine Learning (ML); Deep Learning (DL)	Diagnosis & Detection
3895	10.1007/s10278-022-00741-5	2023	Trauma	Deep Learning (DL)	Diagnosis & Detection
3896	10.1007/s00068-022-02136-1	2023	Trauma	Deep Learning (DL)	Prognosis & Prediction
3897	10.1007/s00068-022-02128-1	2023	Trauma	Deep Learning (DL)	Diagnosis & Detection
3898	10.1177/02841851221122424	2023	Trauma	Machine Learning (ML); Deep Learning (DL)	Diagnosis & Detection
3899	10.1002/cyto.a.24686	2023	Unclassified	Unclassified	Prognosis & Prediction
3900	10.1007/s13139-022-00765-3	2023	Unclassified	Deep Learning (DL)	Segmentation & Measurement

3901	10.1002/lary.30239	2023	Arthroplasty	Unclassified	Prognosis & Prediction
3902	10.1038/s41433-022-02055-w	2023	Arthroplasty	Deep Learning (DL)	Diagnosis & Detection
3903	10.1111/odi.14056	2023	Arthroplasty	Deep Learning (DL)	Prognosis & Prediction
3904	10.1001/jamanetworkopen.2023.3391	2023	Trauma; Arthroplasty	Natural Language Processing (NLP); Machine Learning (ML); Deep Learning (DL)	Prognosis & Prediction
3905	10.3390/cancers15061837	2023	Unclassified	Machine Learning (ML); Deep Learning (DL)	Diagnosis & Detection
3906	10.3390/jcm12062369	2023	Trauma	Machine Learning (ML)	Prognosis & Prediction
3907	10.21037/qims-22-658	2023	Pediatric Orthopaedics	Deep Learning (DL)	Surgical Planning
3908	10.1148/radiol.212922	2023	Spine Surgery	Deep Learning (DL)	Diagnosis & Detection
3909	10.1007/s11547-023-01608-7	2023	Arthroplasty	Machine Learning (ML)	Prognosis & Prediction
3910	10.1148/radiol.220505	2023	Pediatric Orthopaedics	Deep Learning (DL)	Prognosis & Prediction
3911	10.1016/j.orthtr.2022.12.002	2023	Sports Medicine; Trauma	Machine Learning (ML)	Unclassified
3912	10.1016/j.bone.2022.116653	2023	Trauma; Spine Surgery	Natural Language Processing (NLP); Machine Learning (ML)	Prognosis & Prediction
3913	10.1007/s13246-023-01215-w	2023	Sports Medicine; Trauma; Pediatric Orthopaedics	Machine Learning (ML); Deep Learning (DL)	Prognosis & Prediction
3914	10.1007/s00330-022-09205-4	2023	Trauma	Deep Learning (DL)	Prognosis & Prediction
3915	10.1007/s00330-022-09156-w	2023	Trauma; Spine Surgery	Deep Learning (DL)	Diagnosis & Detection
3916	10.1007/s00330-022-09151-1	2023	Sports Medicine; Arthroplasty	Deep Learning (DL)	Diagnosis & Detection
3917	10.1111/jep.13765	2023	Unclassified	Machine Learning (ML)	Prognosis & Prediction
3918	10.1186/s12968-023-00988-z	2023	Arthroplasty	Deep Learning (DL)	Prognosis & Prediction
3919	10.3389/fonc.2023.1125637	2023	Unclassified	Deep Learning (DL)	Prognosis & Prediction
3920	10.3390/jpm13020367	2023	Sports Medicine	Machine Learning (ML)	Prognosis & Prediction
3921	10.1259/dmfr.20220345	2023	Sports Medicine; Trauma	Deep Learning (DL)	Prognosis & Prediction
3922	10.3390/jcm12030942	2023	Arthroplasty	Deep Learning (DL)	Prognosis & Prediction
3923	10.3390/jcm12030744	2023	Trauma; Arthroplasty	Unclassified	Prognosis & Prediction

3924	10.1177/02841851221092164	2023	Arthroplasty	Deep Learning (DL)	Prognosis & Prediction
3925	10.1016/j.semarthrit.2022.152140	2023	Arthroplasty	Machine Learning (ML)	Prognosis & Prediction
3926	10.1038/s41551-022-00940-z	2023	Unclassified	Deep Learning (DL)	Surgical Planning
3927	10.1016/j.diii.2022.08.004	2023	Spine Surgery	Deep Learning (DL)	Diagnosis & Detection
3928	10.1007/s10278-022-00686-9	2023	Unclassified	Deep Learning (DL)	Unclassified
3929	10.1177/08465371221121074	2023	Trauma; Spine Surgery	Natural Language Processing (NLP); Machine Learning (ML)	Unclassified
3930	10.1177/02841851221083519	2023	Trauma	Deep Learning (DL)	Prognosis & Prediction
3931	10.3389/fonc.2023.1097897	2023	Unclassified	Deep Learning (DL)	Prognosis & Prediction
3932	10.3389/fphys.2022.1061911	2023	Spine Surgery	Deep Learning (DL)	Prognosis & Prediction
3933	10.3389/fonc.2022.1054300	2023	Unclassified	Machine Learning (ML)	Prognosis & Prediction
3934	10.3389/fimmu.2022.1072526	2023	Arthroplasty	Machine Learning (ML)	Diagnosis & Detection
3935	10.1001/jamanetworkopen.2022.53820	2023	Spine Surgery	Deep Learning (DL)	Prognosis & Prediction
3936	10.1001/jamanetworkopen.2022.51849	2023	Pediatric Orthopaedics	Deep Learning (DL)	Prognosis & Prediction
3937	10.2196/44779	2023	Spine Surgery	Natural Language Processing (NLP); Machine Learning (ML); Deep Learning (DL)	Prognosis & Prediction
3938	10.22141/pjs.13.4.2023.394	2023	Trauma; Spine Surgery	Machine Learning (ML); Deep Learning (DL)	Prognosis & Prediction
3939	10.1590/0100-3984.2023.0020-en	2023	Unclassified	Machine Learning (ML); Deep Learning (DL)	Diagnosis & Detection
3940	10.1590/0100-3984.2023.0056-en	2023	Unclassified	Machine Learning (ML); Deep Learning (DL)	Prognosis & Prediction
3941	10.3389/fradi.2023.1241651	2023	Unclassified	Deep Learning (DL)	Segmentation & Measurement
3942	10.3389/fbioe.2023.1255625	2023	Arthroplasty	Machine Learning (ML); Robotics; Deep Learning (DL)	Prognosis & Prediction
3943	10.3389/fradi.2023.1242902	2023	Unclassified	Machine Learning (ML); Deep Learning (DL)	Unclassified

3944	10.3389/fonc.2023.1330977	2023	Pediatric Orthopaedics	Deep Learning (DL)	Surgical Planning
3945	10.21037/qims-23-744	2023	Unclassified	Deep Learning (DL)	Prognosis & Prediction
3946	10.1259/bjr.20230047	2023	Spine Surgery	Deep Learning (DL)	Diagnosis & Detection
3947	10.1177/20552076231216549	2023	Trauma	Deep Learning (DL)	Prognosis & Prediction
3948	10.1177/20552076231216419	2023	Unclassified	Machine Learning (ML)	Prognosis & Prediction
3949	10.3389/fimmu.2023.1291727	2023	Unclassified	Machine Learning (ML); Deep Learning (DL)	Unclassified
3950	10.21569/2222-7415-2023-13-3-5-13	2023	Trauma	Deep Learning (DL)	Diagnosis & Detection
3951	10.26641/2307-0404.2023.3.288965	2023	Trauma	Unclassified	Prognosis & Prediction
3952	10.2196/47590	2023	Unclassified	Natural Language Processing (NLP); Machine Learning (ML); Deep Learning (DL)	Prognosis & Prediction
3953	10.1016/j.ibmed.2023.100111	2023	Arthroplasty	Machine Learning (ML)	Prognosis & Prediction
3954	10.5624/isd.20230092	2023	Unclassified	Unclassified	Prognosis & Prediction
3955	10.2147/DMSO.S422515	2023	Unclassified	Machine Learning (ML)	Prognosis & Prediction
3956	10.15761/0101-60830000000626	2023	Trauma	Machine Learning (ML); Deep Learning (DL)	Unclassified
3957	10.3389/fbioe.2023.1241151	2023	Unclassified	Machine Learning (ML); Deep Learning (DL)	Unclassified
3958	10.3389/fonc.2023.1207175	2023	Unclassified	Machine Learning (ML); Deep Learning (DL)	Prognosis & Prediction
3959	10.2196/47621	2023	Spine Surgery	Natural Language Processing (NLP)	Prognosis & Prediction
3960	10.1016/j.jpi.2023.100333	2023	Unclassified	Deep Learning (DL)	Segmentation & Measurement
3961	10.3389/fmed.2023.1224489	2023	Trauma; Arthroplasty	Deep Learning (DL)	Prognosis & Prediction
3962	10.3389/fmed.2023.1178798	2023	Trauma	Deep Learning (DL)	Prognosis & Prediction
3963	10.3389/fbioe.2023.1215770	2023	Sports Medicine; Arthroplasty	Machine Learning (ML); Deep Learning (DL)	Prognosis & Prediction
3964	10.2147/IDR.S417431	2023	Trauma; Spine Surgery	Machine Learning (ML)	Prognosis & Prediction

3965	10.1080/21681163.2023.2245922	2023	Pediatric Orthopaedics; Spine Surgery	Natural Language Processing (NLP); Machine Learning (ML); Deep Learning (DL)	Diagnosis & Detection
3966	10.3389/fimmu.2023.1168780	2023	Unclassified	Machine Learning (ML)	Prognosis & Prediction
3967	10.4103/jdmimsu.jdmimsu_281_22	2023	Unclassified	Deep Learning (DL)	Surgical Planning
3968	10.3389/fbioe.2023.1208711	2023	Arthroplasty	Machine Learning (ML); Deep Learning (DL)	Prognosis & Prediction
3969	10.3389/fcimb.2023.1206393	2023	Trauma	Machine Learning (ML)	Prognosis & Prediction
3970	10.3290/j.ijcd.b3840535	2023	Unclassified	Deep Learning (DL)	Surgical Planning
3971	10.1259/dmfr.20220413	2023	Unclassified	Deep Learning (DL)	Prognosis & Prediction
3972	10.3389/fpain.2023.1177070	2023	Unclassified	Machine Learning (ML)	Prognosis & Prediction
3973	10.1177/20552076231184048	2023	Arthroplasty	Machine Learning (ML); Deep Learning (DL)	Prognosis & Prediction
3974	10.3389/fendo.2023.1198763	2023	Unclassified	Machine Learning (ML)	Diagnosis & Detection
3975	10.1177/20420986231181337	2023	Pediatric Orthopaedics; Arthroplasty	Machine Learning (ML)	Prognosis & Prediction
3976	10.1177/10760296231186145	2023	Arthroplasty	Machine Learning (ML)	Prognosis & Prediction
3977	10.2174/1573405619666230130143020	2023	Sports Medicine; Arthroplasty	Natural Language Processing (NLP); Machine Learning (ML); Deep Learning (DL)	Diagnosis & Detection
3978	10.3934/mbe.2023585	2023	Pediatric Orthopaedics	Deep Learning (DL)	Prognosis & Prediction
3979	10.3389/fbioe.2023.1108021	2023	Spine Surgery; Arthroplasty	Deep Learning (DL)	Diagnosis & Detection
3980	10.3389/fneur.2023.1195923	2023	Sports Medicine	Deep Learning (DL)	Prognosis & Prediction
3981	10.1177/11769351231165161	2023	Unclassified	Machine Learning (ML)	Prognosis & Prediction
3982	10.3389/fendo.2023.1098898	2023	Spine Surgery	Machine Learning (ML); Deep Learning (DL)	Diagnosis & Detection
3983	10.3389/fphys.2023.1146910	2023	Trauma	Deep Learning (DL)	Prognosis & Prediction
3984	10.3389/fimmu.2023.1134412	2023	Unclassified	Machine Learning (ML)	Diagnosis & Detection

3985	10.2174/1570162X21666221128153942	2023	Trauma	Deep Learning (DL)	Prognosis & Prediction
3986	10.1177/1759720X231165560	2023	Sports Medicine; Trauma; Arthroplasty	Deep Learning (DL)	Prognosis & Prediction
3987	10.15789/2220-7619-UOS-2008	2023	Arthroplasty	Machine Learning (ML); Deep Learning (DL)	Prognosis & Prediction
3988	10.3389/fbioe.2023.1164655	2023	Unclassified	Deep Learning (DL)	Diagnosis & Detection
3989	10.1080/21681163.2023.2203266	2023	Unclassified	Deep Learning (DL)	Diagnosis & Detection
3990	10.3389/fcimb.2023.1139998	2023	Arthroplasty	Machine Learning (ML); Deep Learning (DL)	Unclassified
3991	10.1177/15330338231165131	2023	Unclassified	Machine Learning (ML)	Prognosis & Prediction
3992	10.3389/fendo.2023.1132725	2023	Trauma; Spine Surgery	Deep Learning (DL)	Diagnosis & Detection
3993	10.3389/fendo.2023.1025749	2023	Trauma; Spine Surgery	Deep Learning (DL)	Prognosis & Prediction
3994	10.3389/fonc.2023.1121594	2023	Unclassified	Machine Learning (ML); Deep Learning (DL)	Prognosis & Prediction
3995	10.1016/j.cmpbup.2023.100104	2023	Trauma	Natural Language Processing (NLP); Machine Learning (ML)	Prognosis & Prediction
3996	10.1016/j.imu.2023.101233	2023	Unclassified	Machine Learning (ML); Deep Learning (DL)	Prognosis & Prediction
3997	10.1016/j.imu.2023.101238	2023	Trauma; Spine Surgery	Machine Learning (ML); Deep Learning (DL)	Diagnosis & Detection
3998	10.1177/10732748231167958	2023	Arthroplasty	Machine Learning (ML)	Prognosis & Prediction
3999	10.1016/j.ejro.2023.100482	2023	Pediatric Orthopaedics; Spine Surgery	Deep Learning (DL)	Prognosis & Prediction
4000	10.3233/XST-221343	2023	Trauma; Spine Surgery	Deep Learning (DL)	Diagnosis & Detection
4001	10.1177/1759720X231158198	2023	Arthroplasty	Machine Learning (ML); Deep Learning (DL)	Prognosis & Prediction
4002	10.3389/fphys.2023.1132214	2023	Arthroplasty	Deep Learning (DL)	Diagnosis & Detection
4003	10.3389/fonc.2023.1144039	2023	Unclassified	Machine Learning (ML); Deep Learning (DL)	Prognosis & Prediction
4004	10.2147/JPR.S388219	2023	Spine Surgery	Deep Learning (DL)	Segmentation & Measurement

4005	10.1148/ryai.220050	2023	Arthroplasty	Machine Learning (ML); Deep Learning (DL)	Prognosis & Prediction
4006	10.1177/15330338221150069	2023	Unclassified	Deep Learning (DL)	Prognosis & Prediction
4007	10.3390/jcm12020499	2023	Pediatric Orthopaedics; Spine Surgery	Deep Learning (DL)	Prognosis & Prediction
4008	10.21037/qims-22-470	2023	Sports Medicine; Trauma; Arthroplasty	Deep Learning (DL)	Prognosis & Prediction
4009	10.3390/diagnostics13010112	2023	Unclassified	Machine Learning (ML); Deep Learning (DL)	Diagnosis & Detection
4010	10.1177/10760296221139263	2023	Arthroplasty	Machine Learning (ML); Deep Learning (DL)	Prognosis & Prediction
4011	10.1109/TNSRE.2022.3226860	2023	Arthroplasty	Deep Learning (DL)	Prognosis & Prediction
4012	10.1016/j.ebiom.2022.104426	2023	Unclassified	Machine Learning (ML); Deep Learning (DL)	Prognosis & Prediction
4013	10.1007/s00330-022-08967-1	2023	Sports Medicine; Trauma; Arthroplasty	Machine Learning (ML); Deep Learning (DL)	Prognosis & Prediction
4014	10.1097/RLI.0000000000000928	2023	Spine Surgery; Arthroplasty	Machine Learning (ML); Deep Learning (DL)	Diagnosis & Detection
4015	10.1097/RLI.0000000000000907	2023	Sports Medicine; Trauma	Machine Learning (ML); Deep Learning (DL)	Prognosis & Prediction
4016	10.1002/art.42316	2023	Arthroplasty	Machine Learning (ML)	Prognosis & Prediction
4017	10.1016/j.diii.2022.10.004	2023	Unclassified	Machine Learning (ML); Deep Learning (DL)	Prognosis & Prediction
4018	10.1007/s00330-022-08950-w	2023	Trauma	Deep Learning (DL)	Diagnosis & Detection
4019	10.1097/MD.0000000000032369	2022	Trauma; Spine Surgery	Unclassified	Prognosis & Prediction
4020	10.1017/S0263574722000984	2022	Arthroplasty	Machine Learning (ML); Deep Learning (DL)	Diagnosis & Detection
4021	10.3389/fped.2022.1005099	2022	Trauma; Pediatric Orthopaedics	Deep Learning (DL)	Prognosis & Prediction
4022	10.3389/fonc.2022.1095059	2022	Unclassified	Machine Learning (ML)	Prognosis & Prediction
4023	10.1186/s12877-022-03631-1	2022	Trauma	Machine Learning (ML)	Prognosis & Prediction
4024	10.1016/j.compmedimag.2022.102141	2022	Unclassified	Deep Learning (DL)	Diagnosis & Detection
4025	10.1097/TA.0000000000003791	2022	Trauma	Machine Learning (ML); Deep Learning (DL)	Prognosis & Prediction

4026	10.1148/radiol.212526	2022	Arthroplasty	Deep Learning (DL)	Diagnosis & Detection
4027	10.1016/j.compbiomed.2022.106295	2022	Arthroplasty	Deep Learning (DL)	Unclassified
4028	10.1097/FTD.0000000000001020	2022	Pediatric Orthopaedics; Arthroplasty	Deep Learning (DL)	Prognosis & Prediction
4029	10.1038/s43856-022-00107-6	2022	Unclassified	Deep Learning (DL)	Diagnosis & Detection
4030	10.1186/s13256-022-03653-8	2022	Spine Surgery	Machine Learning (ML)	Prognosis & Prediction
4031	10.1016/j.medntd.2022.100188	2022	Arthroplasty	Machine Learning (ML); Deep Learning (DL)	Unclassified
4032	10.1186/s12911-022-02018-x	2022	Arthroplasty	Machine Learning (ML); Deep Learning (DL)	Prognosis & Prediction
4033	10.1186/s13075-022-02914-7	2022	Arthroplasty	Natural Language Processing (NLP); Deep Learning (DL)	Diagnosis & Detection
4034	10.1016/j.clinimag.2022.09.005	2022	Trauma; Arthroplasty	Deep Learning (DL)	Prognosis & Prediction
4035	10.2214/AJR.22.27873	2022	Trauma; Pediatric Orthopaedics	Deep Learning (DL)	Diagnosis & Detection
4036	10.1115/1.4055238	2022	Arthroplasty	Machine Learning (ML); Deep Learning (DL)	Prognosis & Prediction
4037	10.1186/s12916-022-02491-1	2022	Arthroplasty	Machine Learning (ML)	Prognosis & Prediction
4038	10.1016/j.diii.2022.06.004	2022	Trauma; Pediatric Orthopaedics	Unclassified	Prognosis & Prediction
4039	10.1186/s12888-022-04067-y	2022	Arthroplasty	Machine Learning (ML)	Prognosis & Prediction
4040	10.1007/s10278-022-00669-w	2022	Trauma	Deep Learning (DL)	Diagnosis & Detection
4041	10.1186/s40658-022-00472-0	2022	Unclassified	Deep Learning (DL)	Diagnosis & Detection
4042	10.1186/s13244-022-01234-3	2022	Pediatric Orthopaedics; Trauma; Arthroplasty	Natural Language Processing (NLP); Machine Learning (ML); Deep Learning (DL)	Prognosis & Prediction
4043	10.1007/s00068-022-01981-4	2022	Trauma	Machine Learning (ML)	Prognosis & Prediction
4044	10.1186/s12877-022-03152-x	2022	Trauma	Machine Learning (ML); Deep Learning (DL)	Prognosis & Prediction
4045	10.1186/s12885-022-09491-7	2022	Trauma	Machine Learning (ML)	Prognosis & Prediction

4046	10.1186/s12883-022-02670-w	2022	Spine Surgery	Natural Language Processing (NLP); Machine Learning (ML); Deep Learning (DL)	Prognosis & Prediction
4047	10.1016/j.acra.2022.02.020	2022	Trauma; Spine Surgery	Deep Learning (DL)	Prognosis & Prediction
4048	10.1186/s13075-021-02716-3	2022	Sports Medicine	Machine Learning (ML); Deep Learning (DL)	Unclassified
4049	10.1186/s13075-022-02743-8	2022	Arthroplasty	Natural Language Processing (NLP); Machine Learning (ML); Deep Learning (DL)	Prognosis & Prediction
4050	10.1038/s41413-022-00192-2	2022	Unclassified	Deep Learning (DL)	Prognosis & Prediction
4051	10.1186/s12885-022-09307-8	2022	Unclassified	Deep Learning (DL)	Prognosis & Prediction
4052	10.1186/s12911-022-01751-7	2022	Arthroplasty	Natural Language Processing (NLP); Machine Learning (ML)	Prognosis & Prediction
4053	10.1515/bams-2020-0047	2022	Spine Surgery	Machine Learning (ML); Deep Learning (DL)	Segmentation & Measurement
4054	10.3389/fgene.2022.1078200	2022	Arthroplasty	Machine Learning (ML); Deep Learning (DL)	Prognosis & Prediction
4055	10.3389/fgene.2022.1054035	2022	Unclassified	Natural Language Processing (NLP); Machine Learning (ML)	Prognosis & Prediction
4056	10.3389/fpubh.2022.1015952	2022	Unclassified	Machine Learning (ML)	Prognosis & Prediction
4057	10.3389/fped.2022.986500	2022	Pediatric Orthopaedics	Deep Learning (DL)	Unclassified
4058	10.1148/ryai.220067	2022	Spine Surgery; Arthroplasty	Machine Learning (ML); Deep Learning (DL)	Prognosis & Prediction
4059	10.3899/jrheum.220326	2022	Arthroplasty	Machine Learning (ML); Deep Learning (DL)	Prognosis & Prediction
4060	10.3390/healthcare10112132	2022	Spine Surgery	Machine Learning (ML); Deep Learning (DL)	Segmentation & Measurement
4061	10.1016/j.ejrad.2022.110531	2022	Spine Surgery	Deep Learning (DL)	Unclassified
4062	10.1097/RLI.0000000000000891	2022	Unclassified	Deep Learning (DL)	Prognosis & Prediction
4063	10.1002/jcu.23321	2022	Unclassified	Natural Language Processing (NLP); Machine Learning (ML); Deep Learning (DL)	Diagnosis & Detection
4064	10.1002/mrm.29356	2022	Trauma; Pediatric Orthopaedics	Deep Learning (DL)	Prognosis & Prediction

4065	10.1007/s00330-022-08741-3	2022	Unclassified	Deep Learning (DL)	Diagnosis & Detection
4066	10.1109/JBHI.2021.3066832	2022	Arthroplasty	Deep Learning (DL)	Diagnosis & Detection
4067	10.3389/fgene.2022.957718	2022	Arthroplasty	Machine Learning (ML); Deep Learning (DL)	Diagnosis & Detection
4068	10.3389/fpubh.2022.1019168	2022	Unclassified	Machine Learning (ML); Deep Learning (DL)	Prognosis & Prediction
4069	10.3390/jpm12101706	2022	Arthroplasty	Machine Learning (ML)	Prognosis & Prediction
4070	10.1007/s00247-022-05496-3	2022	Trauma; Pediatric Orthopaedics	Unclassified	Diagnosis & Detection
4071	10.1016/j.artmed.2022.102396	2022	Unclassified	Machine Learning (ML)	Prognosis & Prediction
4072	10.1148/radiol.220076	2022	Spine Surgery	Deep Learning (DL)	Prognosis & Prediction
4073	10.1007/s11914-022-00743-w	2022	Trauma; Spine Surgery	Machine Learning (ML); Deep Learning (DL)	Prognosis & Prediction
4074	10.1016/j.jocd.2022.06.002	2022	Trauma; Spine Surgery	Machine Learning (ML)	Segmentation & Measurement
4075	10.1016/j.rcot.2022.06.027	2022	Spine Surgery	Machine Learning (ML); Robotics; Deep Learning (DL)	Surgical Planning
4076	10.1007/s00234-022-03024-6	2022	Spine Surgery	Deep Learning (DL)	Unclassified
4077	10.1002/psp4.12843	2022	Trauma	Machine Learning (ML); Deep Learning (DL)	Prognosis & Prediction
4078	10.1007/s00330-022-08981-3	2022	Unclassified	Machine Learning (ML); Deep Learning (DL)	Prognosis & Prediction
4079	10.1016/j.mri.2022.06.012	2022	Spine Surgery; Arthroplasty	Deep Learning (DL)	Unclassified
4080	10.1007/s00330-022-08956-4	2022	Trauma	Unclassified	Unclassified
4081	10.1007/s40618-022-01837-z	2022	Trauma; Spine Surgery	Machine Learning (ML)	Prognosis & Prediction
4082	10.1007/s10140-022-02060-2	2022	Arthroplasty	Deep Learning (DL)	Prognosis & Prediction
4083	10.1007/s00247-022-05380-0	2022	Sports Medicine; Trauma; Pediatric Orthopaedics	Deep Learning (DL)	Unclassified
4084	10.1177/00258024221079092	2022	Unclassified	Deep Learning (DL)	Prognosis & Prediction
4085	10.1007/s00247-021-05130-8	2022	Pediatric Orthopaedics; Trauma; Spine Surgery	Unclassified	Diagnosis & Detection

4086	10.3389/fbioe.2022.973275	2022	Unclassified	Deep Learning (DL)	Prognosis & Prediction
4087	10.3389/fpubh.2022.1003976	2022	Unclassified	Machine Learning (ML)	Prognosis & Prediction
4088	10.3389/fpubh.2022.969919	2022	Trauma; Spine Surgery	Machine Learning (ML)	Prognosis & Prediction
4089	10.3389/fpubh.2022.984750	2022	Unclassified	Machine Learning (ML)	Prognosis & Prediction
4090	10.3389/fendo.2022.971877	2022	Spine Surgery	Deep Learning (DL)	Prognosis & Prediction
4091	10.1017/wtc.2022.18	2022	Arthroplasty	Robotics; Deep Learning (DL)	Prognosis & Prediction
4092	10.3389/fbioe.2022.927926	2022	Trauma	Deep Learning (DL)	Diagnosis & Detection
4093	10.3389/fbioe.2022.961108	2022	Unclassified	Machine Learning (ML)	Prognosis & Prediction
4094	10.1016/j.yacr.2022.04.010	2022	Sports Medicine	Machine Learning (ML); Deep Learning (DL)	Segmentation & Measurement
4095	10.2196/36130	2022	Unclassified	Natural Language Processing (NLP); Machine Learning (ML)	Prognosis & Prediction
4096	10.3390/biomedicines10092323	2022	Spine Surgery	Deep Learning (DL)	Prognosis & Prediction
4097	10.1093/mr/roab073	2022	Arthroplasty	Deep Learning (DL)	Diagnosis & Detection
4098	10.1016/j.compbiomed.2022.105885	2022	Sports Medicine; Arthroplasty	Machine Learning (ML)	Prognosis & Prediction
4099	10.1002/jbmr.4649	2022	Arthroplasty	Machine Learning (ML)	Prognosis & Prediction
4100	10.1016/j.ejrad.2022.110447	2022	Pediatric Orthopaedics; Trauma; Arthroplasty	Deep Learning (DL)	Diagnosis & Detection
4101	10.1016/j.ejrad.2022.110445	2022	Spine Surgery	Deep Learning (DL)	Diagnosis & Detection
4102	10.1016/j.cmpb.2022.107019	2022	Pediatric Orthopaedics	Machine Learning (ML); Deep Learning (DL)	Prognosis & Prediction
4103	10.1016/j.ejrad.2022.110434	2022	Trauma	Deep Learning (DL)	Diagnosis & Detection
4104	10.1016/j.ejrad.2022.110414	2022	Spine Surgery; Arthroplasty	Deep Learning (DL)	Unclassified
4105	10.1002/mp.15790	2022	Arthroplasty	Deep Learning (DL)	Unclassified
4106	10.1007/s00330-022-08764-w	2022	Pediatric Orthopaedics	Machine Learning (ML); Deep Learning (DL)	Surgical Planning
4107	10.1053/j.sart.2022.01.003	2022	Arthroplasty	Machine Learning (ML); Deep Learning (DL)	Prognosis & Prediction
4108	10.1007/s00330-022-08729-z	2022	Spine Surgery; Arthroplasty	Deep Learning (DL)	Unclassified
4109	10.1007/s00330-022-08708-4	2022	Spine Surgery; Trauma; Arthroplasty	Deep Learning (DL)	Unclassified
4110	10.3389/fonc.2022.973307	2022	Trauma	Machine Learning (ML)	Prognosis & Prediction

4111	10.3389/fendo.2022.884306	2022	Trauma; Spine Surgery	Deep Learning (DL)	Diagnosis & Detection
4112	10.1161/JAHA.121.023745	2022	Arthroplasty	Machine Learning (ML)	Prognosis & Prediction
4113	10.3803/EnM.2022.1461	2022	Trauma; Spine Surgery	Deep Learning (DL)	Prognosis & Prediction
4114	10.2196/38155	2022	Trauma; Arthroplasty	Natural Language Processing (NLP)	Diagnosis & Detection
4115	10.3390/ijerph19159552	2022	Unclassified	Natural Language Processing (NLP)	Prognosis & Prediction
4116	10.3174/ajnr.A7588	2022	Pediatric Orthopaedics	Deep Learning (DL)	Unclassified
4117	10.3389/fpubh.2022.906774	2022	Unclassified	Machine Learning (ML)	Prognosis & Prediction
4118	10.3390/medicina58080998	2022	Trauma; Spine Surgery	Machine Learning (ML); Deep Learning (DL)	Prognosis & Prediction
4119	10.3390/tomography8040148	2022	Unclassified	Deep Learning (DL)	Diagnosis & Detection
4120	10.1016/j.radi.2022.05.005	2022	Spine Surgery	Natural Language Processing (NLP); Machine Learning (ML); Deep Learning (DL)	Prognosis & Prediction
4121	10.1016/j.bspc.2022.103844	2022	Unclassified	Machine Learning (ML); Deep Learning (DL)	Diagnosis & Detection
4122	10.1016/j.ejmp.2022.06.006	2022	Unclassified	Deep Learning (DL)	Unclassified
4123	10.1016/j.ejrad.2022.110366	2022	Trauma; Pediatric Orthopaedics	Natural Language Processing (NLP); Machine Learning (ML); Deep Learning (DL)	Segmentation & Measurement
4124	10.1016/j.bone.2022.116427	2022	Trauma; Spine Surgery	Machine Learning (ML); Deep Learning (DL)	Prognosis & Prediction
4125	10.1007/s10278-022-00616-9	2022	Arthroplasty	Robotics; Deep Learning (DL)	Prognosis & Prediction
4126	10.1002/jmri.28069	2022	Trauma; Arthroplasty	Deep Learning (DL)	Diagnosis & Detection
4127	10.1097/MD.0000000000029542	2022	Arthroplasty	Machine Learning (ML)	Prognosis & Prediction
4128	10.3389/fonc.2022.908873	2022	Sports Medicine; Trauma	Machine Learning (ML); Deep Learning (DL)	Prognosis & Prediction
4129	10.3389/fpubh.2022.949366	2022	Trauma	Deep Learning (DL)	Prognosis & Prediction
4130	10.3389/fpubh.2022.922510	2022	Unclassified	Machine Learning (ML)	Prognosis & Prediction
4131	10.1259/bjr.20211182	2022	Unclassified	Deep Learning (DL)	Diagnosis & Detection
4132	10.3349/ymj.2022.63.7.683	2022	Sports Medicine; Pediatric Orthopaedics	Deep Learning (DL)	Unclassified
4133	10.1016/j.cmpb.2022.106948	2022	Arthroplasty	Deep Learning (DL)	Prognosis & Prediction
4134	10.1148/radiol.211785	2022	Trauma	Unclassified	Prognosis & Prediction

4135	10.3390/jcm11133612	2022	Arthroplasty	Deep Learning (DL)	Prognosis & Prediction
4136	10.21037/qims-21-791	2022	Unclassified	Deep Learning (DL)	Prognosis & Prediction
4137	10.1016/j.ejrad.2022.110337	2022	Arthroplasty	Deep Learning (DL)	Diagnosis & Detection
4138	10.1002/jmri.28025	2022	Unclassified	Deep Learning (DL)	Prognosis & Prediction
4139	10.1088/1361-6560/ac7378	2022	Arthroplasty	Natural Language Processing (NLP); Deep Learning (DL)	Diagnosis & Detection
4140	10.12659/MSM.936733	2022	Sports Medicine; Trauma; Arthroplasty	Deep Learning (DL)	Segmentation & Measurement
4141	10.1159/000525061	2022	Arthroplasty	Deep Learning (DL)	Diagnosis & Detection
4142	10.3389/fnhum.2022.846205	2022	Pediatric Orthopaedics	Machine Learning (ML)	Prognosis & Prediction
4143	10.2196/37804	2022	Arthroplasty	Natural Language Processing (NLP); Deep Learning (DL)	Diagnosis & Detection
4144	10.3390/biomedicines10061247	2022	Sports Medicine	Natural Language Processing (NLP); Machine Learning (ML)	Prognosis & Prediction
4145	10.1002/acr.24539	2022	Sports Medicine; Arthroplasty	Deep Learning (DL)	Diagnosis & Detection
4146	10.21037/qims-21-939	2022	Spine Surgery; Arthroplasty	Deep Learning (DL)	Diagnosis & Detection
4147	10.1016/j.compbiomed.2022.105510	2022	Unclassified	Machine Learning (ML)	Prognosis & Prediction
4148	10.1016/j.jbo.2022.100423	2022	Unclassified	Natural Language Processing (NLP); Machine Learning (ML)	Prognosis & Prediction
4149	10.1016/j.bonr.2022.101179	2022	Unclassified	Deep Learning (DL)	Prognosis & Prediction
4150	10.1007/s00247-022-05295-w	2022	Pediatric Orthopaedics	Robotics	Unclassified
4151	10.1007/s10278-022-00595-x	2022	Pediatric Orthopaedics; Spine Surgery	Machine Learning (ML); Deep Learning (DL)	Diagnosis & Detection
4152	10.1007/s10278-022-00586-y	2022	Trauma; Spine Surgery	Deep Learning (DL)	Prognosis & Prediction
4153	10.1053/j.sart.2021.09.005	2022	Arthroplasty	Machine Learning (ML)	Prognosis & Prediction
4154	10.1007/s11684-021-0828-7	2022	Trauma	Machine Learning (ML); Deep Learning (DL)	Prognosis & Prediction
4155	10.1007/s00296-021-04916-1	2022	Trauma	Machine Learning (ML)	Prognosis & Prediction
4156	10.3389/fmed.2022.906001	2022	Arthroplasty	Machine Learning (ML); Deep Learning (DL)	Prognosis & Prediction
4157	10.3389/fmed.2022.792900	2022	Sports Medicine	Natural Language Processing (NLP); Deep Learning (DL)	Prognosis & Prediction

4158	10.3389/fgene.2022.905027	2022	Unclassified	Machine Learning (ML)	Prognosis & Prediction
4159	10.3389/fpubh.2022.874672	2022	Trauma; Spine Surgery	Machine Learning (ML)	Prognosis & Prediction
4160	10.3390/jpm12050776	2022	Unclassified	Deep Learning (DL)	Prognosis & Prediction
4161	10.2196/37092	2022	Arthroplasty	Natural Language Processing (NLP); Machine Learning (ML)	Prognosis & Prediction
4162	10.3390/jcm11102845	2022	Unclassified	Natural Language Processing (NLP)	Prognosis & Prediction
4163	10.3390/medicina58050636	2022	Pediatric Orthopaedics	Deep Learning (DL)	Prognosis & Prediction
4164	10.1016/S2589-7500(22)00004-8	2022	Trauma	Deep Learning (DL)	Prognosis & Prediction
4165	10.1016/j.ejrad.2022.110249	2022	Unclassified	Unclassified	Prognosis & Prediction
4166	10.1007/s11547-022-01468-7	2022	Sports Medicine; Spine Surgery	Natural Language Processing (NLP); Machine Learning (ML)	Unclassified
4167	10.1002/mp.15577	2022	Unclassified	Deep Learning (DL)	Prognosis & Prediction
4168	10.1016/j.compbiomed.2022.105351	2022	Trauma; Arthroplasty	Machine Learning (ML)	Prognosis & Prediction
4169	10.1002/mp.15554	2022	Pediatric Orthopaedics; Arthroplasty	Deep Learning (DL)	Prognosis & Prediction
4170	10.1016/j.bspc.2022.103532	2022	Pediatric Orthopaedics	Machine Learning (ML); Deep Learning (DL)	Segmentation & Measurement
4171	10.1016/j.bspc.2022.103557	2022	Arthroplasty	Robotics; Deep Learning (DL)	Prognosis & Prediction
4172	10.1007/s00414-021-02746-1	2022	Pediatric Orthopaedics	Deep Learning (DL)	Diagnosis & Detection
4173	10.1007/s00330-021-08419-2	2022	Spine Surgery	Deep Learning (DL)	Prognosis & Prediction
4174	10.1136/emermed-2020-210776	2022	Trauma	Machine Learning (ML)	Prognosis & Prediction
4175	10.3389/fpubh.2022.891766	2022	Spine Surgery	Natural Language Processing (NLP); Deep Learning (DL)	Diagnosis & Detection
4176	10.3389/fonc.2022.844978	2022	Unclassified	Deep Learning (DL)	Prognosis & Prediction
4177	10.3390/jcm11082147	2022	Trauma; Arthroplasty	Machine Learning (ML)	Prognosis & Prediction
4178	10.3390/jpm12040509	2022	Spine Surgery	Machine Learning (ML); Deep Learning (DL)	Prognosis & Prediction
4179	10.3390/jcm11072021	2022	Trauma	Machine Learning (ML); Deep Learning (DL)	Prognosis & Prediction
4180	10.1007/s11547-022-01476-7	2022	Trauma; Arthroplasty	Deep Learning (DL)	Prognosis & Prediction

4181	10.1007/s10278-021-00564-w	2022	Arthroplasty	Deep Learning (DL)	Diagnosis & Detection
4182	10.1007/s10140-021-02000-6	2022	Trauma; Spine Surgery	Deep Learning (DL)	Diagnosis & Detection
4183	10.1016/j.pathol.2021.07.011	2022	Sports Medicine	Deep Learning (DL)	Segmentation & Measurement
4184	10.3389/fmed.2022.807994	2022	Trauma	Machine Learning (ML)	Prognosis & Prediction
4185	10.3389/fmed.2022.841202	2022	Trauma; Arthroplasty	Deep Learning (DL)	Prognosis & Prediction
4186	10.3389/fnana.2022.817903	2022	Spine Surgery	Natural Language Processing (NLP); Machine Learning (ML); Deep Learning (DL)	Segmentation & Measurement
4187	10.3389/fonc.2022.814667	2022	Pediatric Orthopaedics; Spine Surgery	Deep Learning (DL)	Diagnosis & Detection
4188	10.3389/fmed.2022.850552	2022	Sports Medicine; Arthroplasty	Deep Learning (DL)	Unclassified
4189	10.3389/fgene.2022.848116	2022	Arthroplasty	Machine Learning (ML); Deep Learning (DL)	Diagnosis & Detection
4190	10.1148/RYAI.210099	2022	Spine Surgery	Machine Learning (ML); Deep Learning (DL)	Diagnosis & Detection
4191	10.3348/kjr.2021.0449	2022	Trauma; Pediatric Orthopaedics	Deep Learning (DL)	Prognosis & Prediction
4192	10.1016/j.rcl.2021.11.011	2022	Trauma	Deep Learning (DL)	Prognosis & Prediction
4193	10.1148/radiol.210937	2022	Trauma; Spine Surgery	Unclassified	Diagnosis & Detection
4194	10.1148/radiol.211528	2022	Arthroplasty	Deep Learning (DL)	Prognosis & Prediction
4195	10.1007/s13246-022-01106-6	2022	Sports Medicine; Arthroplasty	Machine Learning (ML)	Prognosis & Prediction
4196	10.1111/1556-4029.14996	2022	Trauma	Machine Learning (ML); Deep Learning (DL)	Prognosis & Prediction
4197	10.1016/j.jocn.2022.01.015	2022	Spine Surgery	Natural Language Processing (NLP); Machine Learning (ML); Deep Learning (DL)	Diagnosis & Detection
4198	10.21037/qims-21-629	2022	Arthroplasty	Unclassified	Diagnosis & Detection
4199	10.1016/j.cmpb.2022.106627	2022	Pediatric Orthopaedics; Spine Surgery	Deep Learning (DL)	Diagnosis & Detection
4200	10.1016/j.bspc.2021.103439	2022	Arthroplasty	Deep Learning (DL)	Diagnosis & Detection
4201	10.1016/j.acra.2021.09.005	2022	Spine Surgery	Natural Language Processing (NLP); Machine Learning (ML)	Prognosis & Prediction
4202	10.1016/j.diii.2021.10.007	2022	Trauma; Pediatric Orthopaedics	Deep Learning (DL)	Prognosis & Prediction

4203	10.1007/s00330-021-08284-z	2022	Spine Surgery	Machine Learning (ML); Deep Learning (DL)	Diagnosis & Detection
4204	10.1007/s00330-021-08247-4	2022	Pediatric Orthopaedics; Trauma; Spine Surgery	Deep Learning (DL)	Prognosis & Prediction
4205	10.3389/fped.2022.818061	2022	Sports Medicine; Pediatric Orthopaedics	Deep Learning (DL)	Unclassified
4206	10.4067/S0717-95022022000100107	2022	Unclassified	Deep Learning (DL)	Unclassified
4207	10.3390/ijerph19031880	2022	Unclassified	Machine Learning (ML)	Prognosis & Prediction
4208	10.3390/ijerph19031177	2022	Pediatric Orthopaedics; Spine Surgery	Machine Learning (ML)	Prognosis & Prediction
4209	10.1002/jcu.23143	2022	Arthroplasty	Deep Learning (DL)	Diagnosis & Detection
4210	10.1016/j.ijmedinf.2021.104670	2022	Arthroplasty	Machine Learning (ML); Deep Learning (DL)	Prognosis & Prediction
4211	10.1016/j.pmedr.2021.101674	2022	Pediatric Orthopaedics; Arthroplasty	Machine Learning (ML)	Prognosis & Prediction
4212	10.1007/s10278-021-00519-1	2022	Trauma	Deep Learning (DL)	Diagnosis & Detection
4213	10.21037/qims-21-587	2022	Spine Surgery	Deep Learning (DL)	Prognosis & Prediction
4214	10.1038/s41584-021-00719-7	2022	Arthroplasty	Deep Learning (DL)	Prognosis & Prediction
4215	10.1007/s00198-021-06130-y	2022	Unclassified	Deep Learning (DL)	Diagnosis & Detection
4216	10.1007/s00330-021-08195-z	2022	Sports Medicine; Pediatric Orthopaedics	Machine Learning (ML); Deep Learning (DL)	Diagnosis & Detection
4217	10.1007/s00068-020-01468-0	2022	Trauma	Deep Learning (DL)	Diagnosis & Detection
4218	10.2196/38226	2022	Trauma	Machine Learning (ML); Deep Learning (DL)	Prognosis & Prediction
4219	10.3389/fmedt.2022.995526	2022	Sports Medicine; Spine Surgery; Trauma; Arthroplasty	Machine Learning (ML); Deep Learning (DL)	Unclassified
4220	10.3389/fimmu.2022.979877	2022	Trauma; Spine Surgery	Machine Learning (ML)	Prognosis & Prediction
4221	10.2147/IJGM.S380197	2022	Trauma	Machine Learning (ML); Robotics; Deep Learning (DL)	Prognosis & Prediction
4222	10.1155/2022/3282409	2022	Trauma; Arthroplasty	Unclassified	Prognosis & Prediction
4223	10.1259/dmfr.20210437	2022	Unclassified	Natural Language Processing (NLP); Deep Learning (DL)	Prognosis & Prediction
4224	10.1016/j.imu.2022.101079	2022	Trauma; Spine Surgery	Machine Learning (ML)	Prognosis & Prediction

4225	10.1001/jamanetworkopen.2022.27423	2022	Arthroplasty	Machine Learning (ML)	Prognosis & Prediction
4226	10.1177/1759720X221104935	2022	Unclassified	Machine Learning (ML); Deep Learning (DL)	Prognosis & Prediction
4227	10.1155/2022/5600190	2022	Arthroplasty	Machine Learning (ML)	Diagnosis & Detection
4228	10.1148/ryai.210206	2022	Arthroplasty	Machine Learning (ML); Deep Learning (DL)	Segmentation & Measurement
4229	10.1155/2022/4393154	2022	Arthroplasty	Deep Learning (DL)	Segmentation & Measurement
4230	10.1155/2022/9107021	2022	Trauma; Spine Surgery	Deep Learning (DL)	Prognosis & Prediction
4231	10.2147/CMAR.S340114	2022	Unclassified	Deep Learning (DL)	Diagnosis & Detection
4232	10.1016/j.imu.2022.100961	2022	Spine Surgery	Natural Language Processing (NLP); Machine Learning (ML); Deep Learning (DL)	Unclassified
4233	10.1136/rapm-2021-103299	2022	Arthroplasty	Machine Learning (ML); Deep Learning (DL)	Prognosis & Prediction
4234	10.2147/CIA.S349978	2022	Trauma; Spine Surgery	Machine Learning (ML)	Prognosis & Prediction
4235	10.26355/eurrev_202203_28220	2022	Arthroplasty	Deep Learning (DL)	Diagnosis & Detection
4236	10.1109/TNSRE.2022.3156786	2022	Arthroplasty	Machine Learning (ML); Deep Learning (DL)	Prognosis & Prediction
4237	10.1155/2022/4138666	2022	Sports Medicine; Arthroplasty	Machine Learning (ML)	Prognosis & Prediction
4238	10.1259/dmfr.20210341	2022	Arthroplasty	Deep Learning (DL)	Prognosis & Prediction
4239	10.14744/tjtes.2020.06944	2022	Trauma	Machine Learning (ML); Deep Learning (DL)	Diagnosis & Detection
4240	10.3390/jpm12010114	2022	Arthroplasty	Machine Learning (ML)	Prognosis & Prediction
4241	10.1259/DMFR.20210185	2022	Arthroplasty	Deep Learning (DL)	Prognosis & Prediction
4242	10.1016/j.eclinm.2021.101252	2022	Pediatric Orthopaedics; Spine Surgery	Deep Learning (DL)	Prognosis & Prediction
4243	10.1016/j.ijmedinf.2021.104627	2022	Arthroplasty	Natural Language Processing (NLP); Machine Learning (ML); Deep Learning (DL)	Prognosis & Prediction
4244	10.1016/j.media.2021.102270	2022	Unclassified	Deep Learning (DL)	Diagnosis & Detection
4245	10.1016/j.clinimag.2021.09.010	2022	Trauma	Deep Learning (DL)	Diagnosis & Detection

4246	10.1038/s41375-021-01408-w	2022	Unclassified	Deep Learning (DL)	Prognosis & Prediction
4247	10.1016/j.bspc.2021.103016	2022	Pediatric Orthopaedics	Deep Learning (DL)	Prognosis & Prediction
4248	10.1016/j.bspc.2021.103087	2022	Arthroplasty	Deep Learning (DL)	Prognosis & Prediction
4249	10.1007/s00259-021-05481-2	2022	Unclassified	Machine Learning (ML); Deep Learning (DL)	Diagnosis & Detection
4250	10.1007/s00330-021-08206-z	2022	Unclassified	Deep Learning (DL)	Diagnosis & Detection
4251	10.1007/s00330-021-08070-x	2022	Arthroplasty	Machine Learning (ML)	Prognosis & Prediction
4252	10.4103/digm.digm_10_21	2022	Unclassified	Machine Learning (ML); Deep Learning (DL)	Diagnosis & Detection
4253	10.1097/MD.0000000000028177	2021	Spine Surgery	Unclassified	Diagnosis & Detection
4254	10.3389/fpubh.2021.812023	2021	Trauma; Spine Surgery	Machine Learning (ML)	Prognosis & Prediction
4255	10.3389/fbioe.2021.747217	2021	Sports Medicine; Arthroplasty	Deep Learning (DL)	Diagnosis & Detection
4256	10.1016/j.jbiomech.2021.110860	2021	Spine Surgery; Trauma; Arthroplasty	Deep Learning (DL)	Prognosis & Prediction
4257	10.1016/j.eclinm.2021.101220	2021	Pediatric Orthopaedics; Spine Surgery	Machine Learning (ML); Deep Learning (DL)	Prognosis & Prediction
4258	10.21037/qims-20-1230	2021	Arthroplasty	Natural Language Processing (NLP); Deep Learning (DL)	Diagnosis & Detection
4259	10.1186/s12880-021-00698-x	2021	Unclassified	Deep Learning (DL)	Surgical Planning
4260	10.21037/qims-20-1239	2021	Unclassified	Deep Learning (DL)	Surgical Planning
4261	10.1002/art.41808	2021	Sports Medicine; Arthroplasty	Deep Learning (DL)	Prognosis & Prediction
4262	10.1186/s41747-021-00242-0	2021	Spine Surgery; Trauma; Arthroplasty	Deep Learning (DL)	Prognosis & Prediction
4263	10.1186/s13075-021-02634-4	2021	Sports Medicine; Arthroplasty	Deep Learning (DL)	Prognosis & Prediction
4264	10.1002/nbm.4609	2021	Pediatric Orthopaedics	Deep Learning (DL)	Segmentation & Measurement
4265	10.1186/s12880-021-00662-9	2021	Unclassified	Deep Learning (DL)	Prognosis & Prediction
4266	10.1186/s13244-021-01044-z	2021	Spine Surgery	Deep Learning (DL)	Prognosis & Prediction

4267	10.1007/s00330-021-08096-1	2021	Pediatric Orthopaedics	Deep Learning (DL)	Prognosis & Prediction
4268	10.1007/s10334-021-00934-z	2021	Sports Medicine; Arthroplasty	Deep Learning (DL)	Segmentation & Measurement
4269	10.1007/s00330-021-08014-5	2021	Trauma; Spine Surgery	Deep Learning (DL)	Prognosis & Prediction
4270	10.3389/fonc.2021.773299	2021	Unclassified	Deep Learning (DL)	Prognosis & Prediction
4271	10.1182/blood.2020010568	2021	Unclassified	Deep Learning (DL)	Diagnosis & Detection
4272	10.3389/fgene.2021.771092	2021	Trauma	Machine Learning (ML); Deep Learning (DL)	Prognosis & Prediction
4273	10.1148/ryai.2021200278	2021	Unclassified	Deep Learning (DL)	Unclassified
4274	10.2196/30079	2021	Trauma	Machine Learning (ML)	Prognosis & Prediction
4275	10.1093/jn/nxab266	2021	Pediatric Orthopaedics; Spine Surgery	Machine Learning (ML)	Prognosis & Prediction
4276	10.3390/jpm11111163	2021	Sports Medicine	Deep Learning (DL)	Prognosis & Prediction
4277	10.1016/j.scijus.2021.10.002	2021	Pediatric Orthopaedics	Machine Learning (ML); Deep Learning (DL)	Prognosis & Prediction
4278	10.1016/j.rcl.2021.07.011	2021	Sports Medicine; Trauma; Arthroplasty	Machine Learning (ML); Deep Learning (DL)	Prognosis & Prediction
4279	10.1148/radiol.2021204531	2021	Unclassified	Deep Learning (DL)	Diagnosis & Detection
4280	10.1016/j.ijmedinf.2021.104572	2021	Trauma	Machine Learning (ML); Deep Learning (DL)	Prognosis & Prediction
4281	10.1016/j.cmpb.2021.106325	2021	Arthroplasty	Deep Learning (DL)	Prognosis & Prediction
4282	10.1016/j.jcot.2021.101573	2021	Trauma	Machine Learning (ML); Deep Learning (DL)	Prognosis & Prediction
4283	10.1007/s00330-021-07850-9	2021	Pediatric Orthopaedics; Trauma; Arthroplasty	Deep Learning (DL)	Segmentation & Measurement
4284	10.1007/s00330-021-07951-5	2021	Arthroplasty	Natural Language Processing (NLP); Machine Learning (ML)	Diagnosis & Detection
4285	10.3389/fneur.2021.687931	2021	Trauma	Deep Learning (DL)	Diagnosis & Detection
4286	10.3389/fonc.2021.735739	2021	Unclassified	Deep Learning (DL)	Prognosis & Prediction
4287	10.3803/EnM.2021.1111	2021	Trauma	Machine Learning (ML)	Prognosis & Prediction
4288	10.1093/rpd/ncab073	2021	Unclassified	Machine Learning (ML); Deep Learning (DL)	Segmentation & Measurement
4289	10.1101/gr.271874.120	2021	Arthroplasty	Deep Learning (DL)	Surgical Planning
4290	10.1259/dmfr.20200611	2021	Trauma	Deep Learning (DL)	Unclassified

4291	10.1007/s10278-021-00499-2	2021	Trauma	Machine Learning (ML); Deep Learning (DL)	Prognosis & Prediction
4292	10.1002/acr.24601	2021	Arthroplasty	Machine Learning (ML)	Prognosis & Prediction
4293	10.1177/17531934211026139	2021	Trauma	Deep Learning (DL)	Diagnosis & Detection
4294	10.3389/fonc.2021.742395	2021	Unclassified	Machine Learning (ML); Deep Learning (DL)	Prognosis & Prediction
4295	10.1016/j.jbiomech.2021.110648	2021	Pediatric Orthopaedics; Arthroplasty	Deep Learning (DL)	Segmentation & Measurement
4296	10.1016/j.fri.2021.200477	2021	Spine Surgery	Machine Learning (ML)	Unclassified
4297	10.1148/ryai.2021200248	2021	Trauma	Deep Learning (DL)	Prognosis & Prediction
4298	10.3390/jcm10184074	2021	Spine Surgery	Machine Learning (ML)	Prognosis & Prediction
4299	10.1016/j.compbiomed.2021.104727	2021	Arthroplasty	Deep Learning (DL)	Diagnosis & Detection
4300	10.1002/art.41735	2021	Sports Medicine; Arthroplasty	Machine Learning (ML)	Prognosis & Prediction
4301	10.1016/j.cmpb.2021.106304	2021	Trauma	Natural Language Processing (NLP); Machine Learning (ML); Deep Learning (DL)	Unclassified
4302	10.1007/s13246-021-01032-z	2021	Pediatric Orthopaedics; Spine Surgery	Deep Learning (DL)	Surgical Planning
4303	10.1016/j.bone.2021.116017	2021	Trauma; Spine Surgery	Machine Learning (ML); Deep Learning (DL)	Prognosis & Prediction
4304	10.1016/j.jbiomech.2021.110552	2021	Arthroplasty	Machine Learning (ML); Deep Learning (DL)	Prognosis & Prediction
4305	10.3389/fmed.2021.694733	2021	Trauma	Machine Learning (ML)	Prognosis & Prediction
4306	10.3174/ajnr.A7179	2021	Trauma; Spine Surgery	Deep Learning (DL)	Prognosis & Prediction
4307	10.3390/jpm11080727	2021	Trauma	Machine Learning (ML)	Prognosis & Prediction
4308	10.3390/healthcare9080948	2021	Unclassified	Machine Learning (ML)	Prognosis & Prediction
4309	10.1159/000518382	2021	Unclassified	Natural Language Processing (NLP); Machine Learning (ML)	Prognosis & Prediction
4310	10.1016/j.jbi.2021.103864	2021	Unclassified	Natural Language Processing (NLP); Machine Learning (ML)	Prognosis & Prediction
4311	10.1097/MNM.0000000000001409	2021	Unclassified	Deep Learning (DL)	Unclassified
4312	10.1016/j.compbiomed.2021.104623	2021	Unclassified	Deep Learning (DL)	Diagnosis & Detection

4313	10.4103/jcvjs.jcvjs_94_21	2021	Spine Surgery	Machine Learning (ML); Robotics; Deep Learning (DL)	Surgical Planning
4314	10.1097/APO.0000000000000421	2021	Arthroplasty	Unclassified	Unclassified
4315	10.1148/ryai.2021200260	2021	Trauma	Machine Learning (ML); Deep Learning (DL)	Prognosis & Prediction
4316	10.1093/aje/kwab004	2021	Arthroplasty	Machine Learning (ML)	Prognosis & Prediction
4317	10.1148/ryai.2021200183	2021	Arthroplasty	Deep Learning (DL)	Surgical Planning
4318	10.1016/j.compmedimag.2021.101937	2021	Trauma; Pediatric Orthopaedics	Deep Learning (DL)	Diagnosis & Detection
4319	10.3174/ajnr.A7094	2021	Trauma; Spine Surgery	Deep Learning (DL)	Prognosis & Prediction
4320	10.3390/jimaging7070105	2021	Trauma	Deep Learning (DL)	Prognosis & Prediction
4321	10.3390/diagnostics11071156	2021	Arthroplasty	Natural Language Processing (NLP); Deep Learning (DL)	Prognosis & Prediction
4322	10.3390/diagnostics11071147	2021	Spine Surgery	Deep Learning (DL)	Prognosis & Prediction
4323	10.1148/radiol.2021204289	2021	Pediatric Orthopaedics; Spine Surgery	Deep Learning (DL)	Diagnosis & Detection
4324	10.1016/j.jseint.2021.02.011	2021	Arthroplasty	Machine Learning (ML); Deep Learning (DL)	Prognosis & Prediction
4325	10.1016/j.bspc.2021.102657	2021	Arthroplasty	Deep Learning (DL)	Unclassified
4326	10.1016/j.ejrad.2021.109755	2021	Spine Surgery; Arthroplasty	Natural Language Processing (NLP); Machine Learning (ML)	Diagnosis & Detection
4327	10.1136/jclinpath-2021-207524	2021	Sports Medicine	Machine Learning (ML); Deep Learning (DL)	Diagnosis & Detection
4328	10.1016/j.bone.2021.115943	2021	Sports Medicine; Trauma; Spine Surgery	Machine Learning (ML); Deep Learning (DL)	Prognosis & Prediction
4329	10.1007/s00414-021-02542-x	2021	Pediatric Orthopaedics	Machine Learning (ML); Deep Learning (DL)	Unclassified
4330	10.1016/j.ijrobp.2021.01.042	2021	Arthroplasty	Deep Learning (DL)	Prognosis & Prediction
4331	10.1053/j.sart.2020.12.003	2021	Arthroplasty	Machine Learning (ML)	Prognosis & Prediction
4332	10.1007/s00330-020-07585-z	2021	Arthroplasty	Deep Learning (DL)	Diagnosis & Detection
4333	10.1007/s00330-020-07568-0	2021	Unclassified	Deep Learning (DL)	Prognosis & Prediction
4334	10.1016/j.jbiomech.2021.110439	2021	Arthroplasty	Machine Learning (ML); Deep Learning (DL)	Prognosis & Prediction

4335	10.15441/ceem.20.091	2021	Trauma	Deep Learning (DL)	Prognosis & Prediction
4336	10.47895/AMP.V55I3.2785	2021	Trauma	Machine Learning (ML); Deep Learning (DL)	Diagnosis & Detection
4337	10.1016/j.bonr.2021.101070	2021	Trauma; Spine Surgery	Deep Learning (DL)	Prognosis & Prediction
4338	10.1016/j.ebiom.2021.103402	2021	Pediatric Orthopaedics	Machine Learning (ML); Deep Learning (DL)	Surgical Planning
4339	10.1007/s10278-021-00461-2	2021	Arthroplasty	Machine Learning (ML); Deep Learning (DL)	Prognosis & Prediction
4340	10.1016/j.semss.2021.100871	2021	Spine Surgery	Machine Learning (ML); Deep Learning (DL)	Unclassified
4341	10.1016/j.semss.2021.100876	2021	Spine Surgery	Natural Language Processing (NLP); Machine Learning (ML); Robotics; Deep Learning (DL)	Prognosis & Prediction
4342	10.1016/j.semss.2021.100877	2021	Spine Surgery	Natural Language Processing (NLP)	Unclassified
4343	10.1016/j.semss.2021.100878	2021	Spine Surgery	Machine Learning (ML)	Prognosis & Prediction
4344	10.1016/j.compbiomed.2021.104334	2021	Arthroplasty	Deep Learning (DL)	Diagnosis & Detection
4345	10.1007/s40122-021-00251-2	2021	Unclassified	Machine Learning (ML)	Prognosis & Prediction
4346	10.1177/0192623320981560	2021	Unclassified	Machine Learning (ML); Deep Learning (DL)	Diagnosis & Detection
4347	10.1007/s00330-020-07418-z	2021	Trauma	Deep Learning (DL)	Diagnosis & Detection
4348	10.1097/MD.0000000000026024	2021	Trauma; Spine Surgery	Deep Learning (DL)	Prognosis & Prediction
4349	10.1001/jamanetworkopen.2021.6096	2021	Trauma	Deep Learning (DL)	Prognosis & Prediction
4350	10.3389/fmed.2021.550095	2021	Trauma; Arthroplasty	Machine Learning (ML)	Prognosis & Prediction
4351	10.1158/2643-3230.BCD-20-0162	2021	Unclassified	Natural Language Processing (NLP); Machine Learning (ML); Deep Learning (DL)	Prognosis & Prediction
4352	10.3390/diagnostics11050902	2021	Spine Surgery	Deep Learning (DL)	Prognosis & Prediction
4353	10.3348/kjr.2020.0941	2021	Unclassified	Deep Learning (DL)	Unclassified
4354	10.1002/mp.14705	2021	Arthroplasty	Deep Learning (DL)	Prognosis & Prediction

4355	10.1016/j.compbiomed.2021.104298	2021	Trauma	Machine Learning (ML); Deep Learning (DL)	Segmentation & Measurement
4356	10.4103/jcvjs.jcvjs_186_20	2021	Spine Surgery	Machine Learning (ML); Deep Learning (DL)	Segmentation & Measurement
4357	10.1259/bjr.20201119	2021	Pediatric Orthopaedics	Unclassified	Segmentation & Measurement
4358	10.1016/j.rcot.2021.01.025	2021	Trauma	Deep Learning (DL)	Prognosis & Prediction
4359	10.1002/cam4.3776	2021	Unclassified	Machine Learning (ML)	Prognosis & Prediction
4360	10.1016/j.ejrad.2021.109586	2021	Pediatric Orthopaedics; Spine Surgery	Machine Learning (ML); Deep Learning (DL)	Diagnosis & Detection
4361	10.1136/annrheumdis-2020-217160	2021	Arthroplasty	Machine Learning (ML)	Prognosis & Prediction
4362	10.1007/s00455-020-10124-z	2021	Spine Surgery	Machine Learning (ML); Deep Learning (DL)	Prognosis & Prediction
4363	10.1016/j.ocarto.2020.100135	2021	Trauma; Arthroplasty	Machine Learning (ML); Deep Learning (DL)	Prognosis & Prediction
4364	10.3390/jcm10051009	2021	Unclassified	Natural Language Processing (NLP); Machine Learning (ML); Deep Learning (DL)	Diagnosis & Detection
4365	10.3390/healthcare9030260	2021	Unclassified	Machine Learning (ML)	Prognosis & Prediction
4366	10.21873/anticanres.14905	2021	Unclassified	Machine Learning (ML)	Prognosis & Prediction
4367	10.1016/j.artmed.2021.102035	2021	Arthroplasty	Natural Language Processing (NLP); Deep Learning (DL)	Prognosis & Prediction
4368	10.1007/s00198-020-05735-z	2021	Trauma; Spine Surgery	Machine Learning (ML); Deep Learning (DL)	Prognosis & Prediction
4369	10.1016/j.cmpb.2020.105862	2021	Trauma; Arthroplasty	Deep Learning (DL)	Prognosis & Prediction
4370	10.1001/jamanetworkopen.2020.37107	2021	Trauma; Arthroplasty	Unclassified	Prognosis & Prediction
4371	10.1259/bjr.20200870	2021	Trauma; Pediatric Orthopaedics	Deep Learning (DL)	Diagnosis & Detection
4372	10.1007/s10278-020-00399-x	2021	Trauma	Deep Learning (DL)	Prognosis & Prediction
4373	10.1016/j.jamda.2020.09.025	2021	Trauma	Machine Learning (ML); Deep Learning (DL)	Prognosis & Prediction
4374	10.1177/0846537120947148	2021	Sports Medicine; Trauma; Spine Surgery	Machine Learning (ML); Deep Learning (DL)	Diagnosis & Detection

4375	10.1177/24715492211038172	2021	Arthroplasty	Machine Learning (ML)	Prognosis & Prediction
4376	10.4103/jmsr.jmsr_132_20	2021	Trauma	Machine Learning (ML); Deep Learning (DL)	Diagnosis & Detection
4377	10.18019/1028-4427-2021-27-6-813-820	2021	Spine Surgery	Natural Language Processing (NLP); Machine Learning (ML); Deep Learning (DL)	Prognosis & Prediction
4378	10.1155/2021/1765404	2021	Sports Medicine; Arthroplasty	Machine Learning (ML); Deep Learning (DL)	Prognosis & Prediction
4379	10.3348/KJR.2020.1468	2021	Sports Medicine; Pediatric Orthopaedics	Deep Learning (DL)	Unclassified
4380	10.2147/CMAR.S330591	2021	Unclassified	Machine Learning (ML)	Prognosis & Prediction
4381	10.1155/2021/6231116	2021	Trauma	Deep Learning (DL)	Unclassified
4382	10.1155/2021/5586529	2021	Unclassified	Deep Learning (DL)	Diagnosis & Detection
4383	10.2147/RMHP.S330555	2021	Trauma	Machine Learning (ML)	Prognosis & Prediction
4384	10.1155/2021/2254594	2021	Sports Medicine; Trauma; Arthroplasty	Deep Learning (DL)	Prognosis & Prediction
4385	10.3390/diagnostics11081484	2021	Spine Surgery	Deep Learning (DL)	Unclassified
4386	10.1155/2021/9687591	2021	Spine Surgery; Arthroplasty	Deep Learning (DL)	Unclassified
4387	10.1155/2021/2584291	2021	Sports Medicine; Arthroplasty	Deep Learning (DL)	Diagnosis & Detection
4388	10.12669/pjms.37.6-WIT.4857	2021	Sports Medicine; Trauma; Spine Surgery	Deep Learning (DL)	Surgical Planning
4389	10.1155/2021/5294379	2021	Spine Surgery	Deep Learning (DL)	Prognosis & Prediction
4390	N/A	2021	Spine Surgery; Arthroplasty	Machine Learning (ML)	Prognosis & Prediction
4391	10.1136/rapm-2021-102715	2021	Arthroplasty	Machine Learning (ML); Deep Learning (DL)	Prognosis & Prediction
4392	10.1080/24725838.2021.1938760	2021	Spine Surgery; Arthroplasty	Deep Learning (DL)	Prognosis & Prediction
4393	10.1504/IJMEI.2021.114890	2021	Spine Surgery	Machine Learning (ML); Deep Learning (DL)	Prognosis & Prediction
4394	10.1200/CCI.20.00128	2021	Unclassified	Machine Learning (ML)	Prognosis & Prediction
4395	10.2174/1573405616666200528153453	2021	Unclassified	Deep Learning (DL)	Prognosis & Prediction
4396	10.1177/1759720X21993254	2021	Arthroplasty	Machine Learning (ML)	Prognosis & Prediction
4397	10.14366/usg.20080	2021	Unclassified	Machine Learning (ML); Deep Learning (DL)	Prognosis & Prediction
4398	10.1016/j.imu.2020.100452	2021	Trauma	Deep Learning (DL)	Diagnosis & Detection

4399	10.3390/ijerph18010155	2021	Unclassified	Machine Learning (ML)	Prognosis & Prediction
4400	10.1016/j.ejro.2020.100303	2021	Arthroplasty	Machine Learning (ML); Deep Learning (DL)	Surgical Planning
4401	10.1080/21681163.2020.1817792	2021	Trauma	Machine Learning (ML); Deep Learning (DL)	Prognosis & Prediction
4402	10.1007/s13755-020-00111-x	2020	Trauma	Machine Learning (ML); Deep Learning (DL)	Diagnosis & Detection
4403	10.2196/22765	2020	Arthroplasty	Machine Learning (ML)	Prognosis & Prediction
4404	10.1186/s13244-020-00932-0	2020	Trauma	Deep Learning (DL)	Prognosis & Prediction
4405	10.1038/s41746-020-00352-w	2020	Trauma; Pediatric Orthopaedics	Deep Learning (DL)	Prognosis & Prediction
4406	10.1016/j.ejrad.2020.109373	2020	Trauma	Deep Learning (DL)	Diagnosis & Detection
4407	10.1016/j.compmedimag.2020.101793	2020	Sports Medicine; Arthroplasty	Machine Learning (ML); Deep Learning (DL)	Diagnosis & Detection
4408	10.1007/s12149-020-01524-0	2020	Unclassified	Deep Learning (DL)	Prognosis & Prediction
4409	10.1080/0284186X.2020.1819563	2020	Unclassified	Natural Language Processing (NLP); Machine Learning (ML)	Prognosis & Prediction
4410	10.1016/j.inat.2020.100837	2020	Spine Surgery; Arthroplasty	Natural Language Processing (NLP); Deep Learning (DL)	Diagnosis & Detection
4411	10.1016/j.bonr.2020.100295	2020	Trauma	Deep Learning (DL)	Prognosis & Prediction
4412	10.1038/s42003-020-1057-3	2020	Unclassified	Deep Learning (DL)	Prognosis & Prediction
4413	10.1186/s41747-019-0139-9	2020	Pediatric Orthopaedics	Unclassified	Unclassified
4414	10.1002/jmri.27001	2020	Pediatric Orthopaedics; Spine Surgery; Trauma; Arthroplasty	Deep Learning (DL)	Prognosis & Prediction
4415	10.2478/raon-2020-0068	2020	Unclassified	Deep Learning (DL)	Diagnosis & Detection
4416	10.1097/MD.0000000000023154	2020	Pediatric Orthopaedics	Deep Learning (DL)	Prognosis & Prediction
4417	10.1016/j.ejrad.2020.109303	2020	Sports Medicine; Trauma; Arthroplasty	Deep Learning (DL)	Segmentation & Measurement
4418	10.1007/s12149-020-01510-6	2020	Unclassified	Deep Learning (DL)	Prognosis & Prediction
4419	10.1016/j.bone.2020.115561	2020	Spine Surgery	Deep Learning (DL)	Prognosis & Prediction
4420	10.1159/000509524	2020	Unclassified	Unclassified	Diagnosis & Detection
4421	10.1016/j.cmpb.2020.105583	2020	Unclassified	Deep Learning (DL)	Diagnosis & Detection

4422	10.3389/fbioe.2020.559387	2020	Pediatric Orthopaedics; Spine Surgery	Machine Learning (ML)	Prognosis & Prediction
4423	10.12968/hmed.2020.0309	2020	Arthroplasty	Robotics	Prognosis & Prediction
4424	10.3390/jcm9103341	2020	Unclassified	Deep Learning (DL)	Prognosis & Prediction
4425	10.2196/22550	2020	Trauma; Spine Surgery	Natural Language Processing (NLP); Machine Learning (ML); Deep Learning (DL)	Prognosis & Prediction
4426	10.1001/jamanetworkopen.2020.20961	2020	Spine Surgery	Deep Learning (DL)	Prognosis & Prediction
4427	10.3390/diagnostics10100781	2020	Unclassified	Natural Language Processing (NLP); Machine Learning (ML); Deep Learning (DL)	Prognosis & Prediction
4428	10.1016/j.media.2020.101784	2020	Pediatric Orthopaedics; Arthroplasty	Machine Learning (ML); Deep Learning (DL)	Diagnosis & Detection
4429	10.1007/s00223-020-00734-y	2020	Trauma	Machine Learning (ML); Deep Learning (DL)	Prognosis & Prediction
4430	10.1016/j.nano.2020.102245	2020	Unclassified	Machine Learning (ML); Deep Learning (DL)	Diagnosis & Detection
4431	10.1007/s10278-020-00366-6	2020	Arthroplasty	Deep Learning (DL)	Diagnosis & Detection
4432	10.1007/s10278-020-00364-8	2020	Trauma	Deep Learning (DL)	Prognosis & Prediction
4433	10.1007/s11282-019-00409-x	2020	Trauma	Deep Learning (DL)	Prognosis & Prediction
4434	10.1017/S0263574719000985	2020	Arthroplasty	Deep Learning (DL)	Diagnosis & Detection
4435	10.3389/fendo.2020.00612	2020	Spine Surgery	Deep Learning (DL)	Prognosis & Prediction
4436	10.1016/j.ocarto.2020.100069	2020	Unclassified	Machine Learning (ML)	Prognosis & Prediction
4437	10.21037/jmai-20-30	2020	Unclassified	Machine Learning (ML); Deep Learning (DL)	Prognosis & Prediction
4438	10.1016/j.yacr.2020.05.005	2020	Trauma	Machine Learning (ML); Deep Learning (DL)	Diagnosis & Detection
4439	10.2196/18846	2020	Pediatric Orthopaedics	Machine Learning (ML)	Unclassified
4440	10.5624/ISD.2020.50.3.237	2020	Pediatric Orthopaedics	Deep Learning (DL)	Unclassified
4441	10.3390/cancers12092654	2020	Unclassified	Deep Learning (DL)	Prognosis & Prediction
4442	10.1148/radiol.2020192091	2020	Arthroplasty	Machine Learning (ML); Deep Learning (DL)	Prognosis & Prediction

4443	10.1002/jum.15270	2020	Unclassified	Deep Learning (DL)	Diagnosis & Detection
4444	10.1016/j.ejrad.2020.109188	2020	Trauma	Deep Learning (DL)	Diagnosis & Detection
4445	10.1016/j.bspc.2020.102051	2020	Arthroplasty	Deep Learning (DL)	Unclassified
4446	10.1016/j.ejrad.2020.109139	2020	Trauma	Machine Learning (ML); Deep Learning (DL)	Diagnosis & Detection
4447	10.1016/j.crad.2020.05.021	2020	Trauma	Deep Learning (DL)	Diagnosis & Detection
4448	10.1002/hbm.25039	2020	Arthroplasty	Deep Learning (DL)	Segmentation & Measurement
4449	10.1109/JBHI.2020.2986376	2020	Arthroplasty	Deep Learning (DL)	Segmentation & Measurement
4450	10.1016/j.cmpb.2020.105484	2020	Trauma	Machine Learning (ML); Deep Learning (DL)	Prognosis & Prediction
4451	10.1016/j.diii.2020.01.008	2020	Spine Surgery	Natural Language Processing (NLP); Machine Learning (ML)	Prognosis & Prediction
4452	10.3389/fonc.2020.01209	2020	Unclassified	Unclassified	Prognosis & Prediction
4453	10.3390/diagnostics10080518	2020	Unclassified	Machine Learning (ML); Deep Learning (DL)	Prognosis & Prediction
4454	10.1016/j.bspc.2020.102024	2020	Arthroplasty	Robotics; Deep Learning (DL)	Unclassified
4455	10.1016/j.media.2020.101743	2020	Pediatric Orthopaedics	Deep Learning (DL)	Diagnosis & Detection
4456	10.3348/kjr.2019.0653	2020	Trauma	Deep Learning (DL)	Diagnosis & Detection
4457	10.3348/kjr.2019.0651	2020	Trauma; Spine Surgery	Deep Learning (DL)	Diagnosis & Detection
4458	10.1016/j.ejrad.2020.109043	2020	Unclassified	Natural Language Processing (NLP); Machine Learning (ML)	Prognosis & Prediction
4459	10.1016/j.compbiomed.2020.103796	2020	Spine Surgery	Machine Learning (ML)	Prognosis & Prediction
4460	10.1016/j.compbiomed.2020.103767	2020	Unclassified	Deep Learning (DL)	Prognosis & Prediction
4461	10.1007/s00330-020-06677-0	2020	Spine Surgery	Deep Learning (DL)	Prognosis & Prediction
4462	10.1111/aos.14306	2020	Arthroplasty	Deep Learning (DL)	Diagnosis & Detection
4463	10.2196/16896	2020	Unclassified	Natural Language Processing (NLP); Deep Learning (DL)	Unclassified
4464	10.3390/JCM9051298	2020	Trauma; Arthroplasty	Deep Learning (DL)	Prognosis & Prediction
4465	10.3390/medicina56050243	2020	Trauma	Deep Learning (DL)	Prognosis & Prediction

4466	10.1016/j.media.2020.101685	2020	Arthroplasty	Deep Learning (DL)	Prognosis & Prediction
4467	10.1016/j.ejrad.2020.108925	2020	Trauma	Deep Learning (DL)	Diagnosis & Detection
4468	10.3389/fbioe.2020.00320	2020	Arthroplasty	Natural Language Processing (NLP); Machine Learning (ML)	Segmentation & Measurement
4469	10.2196/15963	2020	Unclassified	Deep Learning (DL)	Prognosis & Prediction
4470	10.4103/jcvjs.JCVJS_37_20	2020	Spine Surgery; Arthroplasty	Machine Learning (ML); Robotics; Deep Learning (DL)	Segmentation & Measurement
4471	10.1016/j.compmedimag.2020.101715	2020	Pediatric Orthopaedics	Machine Learning (ML); Deep Learning (DL)	Prognosis & Prediction
4472	10.1007/s00247-019-04587-y	2020	Pediatric Orthopaedics	Deep Learning (DL)	Prognosis & Prediction
4473	10.1002/jbm4.10337	2020	Trauma; Spine Surgery	Machine Learning (ML)	Prognosis & Prediction
4474	10.21037/QIMS.2020.02.20	2020	Pediatric Orthopaedics; Arthroplasty	Deep Learning (DL)	Unclassified
4475	10.1016/j.jbi.2020.103384	2020	Arthroplasty	Natural Language Processing (NLP); Deep Learning (DL)	Unclassified
4476	10.1016/j.crad.2019.10.022	2020	Pediatric Orthopaedics; Trauma; Spine Surgery	Deep Learning (DL)	Diagnosis & Detection
4477	10.1002/jmri.26872	2020	Sports Medicine	Natural Language Processing (NLP); Machine Learning (ML); Deep Learning (DL)	Diagnosis & Detection
4478	10.1097/RLI.0000000000000615	2020	Trauma; Pediatric Orthopaedics	Deep Learning (DL)	Prognosis & Prediction
4479	10.1088/1361-6560/ab5da0	2020	Arthroplasty	Deep Learning (DL)	Unclassified
4480	10.5535/ARM.20071	2020	Arthroplasty	Machine Learning (ML)	Unclassified
4481	10.1148/RYAI.2020190023	2020	Trauma; Arthroplasty	Deep Learning (DL)	Prognosis & Prediction
4482	10.1148/ryai.2020190116	2020	Arthroplasty	Deep Learning (DL)	Segmentation & Measurement
4483	10.1148/ryai.2020190198	2020	Sports Medicine; Trauma; Pediatric Orthopaedics	Machine Learning (ML); Deep Learning (DL)	Prognosis & Prediction
4484	10.3390/healthcare8040493	2020	Unclassified	Machine Learning (ML); Deep Learning (DL)	Diagnosis & Detection
4485	10.1155/2020/8880786	2020	Trauma	Machine Learning (ML)	Prognosis & Prediction
4486	10.1148/RYAI.2020190065	2020	Arthroplasty	Deep Learning (DL)	Prognosis & Prediction
4487	10.1177/1759720X20933468	2020	Sports Medicine; Trauma; Arthroplasty	Machine Learning (ML)	Prognosis & Prediction

4488	10.1615/CritRevPhysRehabilMed.2020033776	2020	Trauma; Pediatric Orthopaedics	Machine Learning (ML); Robotics; Deep Learning (DL)	Prognosis & Prediction
4489	10.1148/radiol.2020190925	2020	Arthroplasty	Deep Learning (DL)	Segmentation & Measurement
4490	10.1097/RTI.0000000000000484	2020	Spine Surgery	Unclassified	Diagnosis & Detection
4491	10.4258/hir.2020.26.1.61	2020	Pediatric Orthopaedics; Spine Surgery	Deep Learning (DL)	Prognosis & Prediction
4492	10.1055/s-0039-3400266	2020	Trauma; Spine Surgery	Deep Learning (DL)	Diagnosis & Detection
4493	10.1055/s-0039-3400268	2020	Unclassified	Natural Language Processing (NLP); Machine Learning (ML); Deep Learning (DL)	Prognosis & Prediction
4494	10.1155/2020/7163453	2020	Unclassified	Machine Learning (ML)	Prognosis & Prediction
4495	10.1002/jbmr.3849	2020	Trauma	Deep Learning (DL)	Diagnosis & Detection
4496	10.1038/s41746-019-0105-1	2019	Trauma	Natural Language Processing (NLP); Deep Learning (DL)	Prognosis & Prediction
4497	10.1038/s42003-019-0635-8	2019	Pediatric Orthopaedics	Deep Learning (DL)	Prognosis & Prediction
4498	10.1186/s13244-019-0785-8	2019	Arthroplasty	Machine Learning (ML); Robotics	Prognosis & Prediction
4499	10.1016/j.compbiomed.2019.103490	2019	Unclassified	Deep Learning (DL)	Prognosis & Prediction
4500	10.1002/jmri.26761	2019	Arthroplasty	Machine Learning (ML); Deep Learning (DL)	Segmentation & Measurement
4501	10.1002/ima.22323	2019	Unclassified	Robotics; Deep Learning (DL)	Prognosis & Prediction
4502	10.1093/jamia/ocz158	2019	Arthroplasty	Natural Language Processing (NLP); Deep Learning (DL)	Unclassified
4503	10.1148/ryai.2019190053	2019	Pediatric Orthopaedics	Machine Learning (ML); Deep Learning (DL)	Prognosis & Prediction
4504	10.1016/j.jacr.2019.07.028	2019	Arthroplasty	Machine Learning (ML); Robotics	Prognosis & Prediction
4505	10.1016/j.carj.2019.08.010	2019	Arthroplasty	Machine Learning (ML); Robotics	Prognosis & Prediction
4506	10.1016/j.compbiomed.2019.103457	2019	Unclassified	Deep Learning (DL)	Prognosis & Prediction
4507	10.1002/pds.4882	2019	Arthroplasty	Machine Learning (ML)	Prognosis & Prediction
4508	10.1007/s00330-019-06163-2	2019	Pediatric Orthopaedics; Arthroplasty	Deep Learning (DL)	Prognosis & Prediction
4509	10.1007/s00330-019-06082-2	2019	Pediatric Orthopaedics	Machine Learning (ML); Deep Learning (DL)	Prognosis & Prediction

4510	10.1007/s00330-019-06167-y	2019	Trauma	Machine Learning (ML); Deep Learning (DL)	Prognosis & Prediction
4511	10.1007/s12149-019-01386-1	2019	Spine Surgery	Deep Learning (DL)	Prognosis & Prediction
4512	10.1002/mrm.27813	2019	Arthroplasty	Machine Learning (ML); Deep Learning (DL)	Unclassified
4513	10.1016/j.yacr.2019.04.013	2019	Unclassified	Machine Learning (ML); Deep Learning (DL)	Unclassified
4514	10.1016/j.bspc.2019.101614	2019	Arthroplasty	Machine Learning (ML); Deep Learning (DL)	Unclassified
4515	10.1016/j.compbiomed.2019.103365	2019	Unclassified	Deep Learning (DL)	Unclassified
4516	10.1109/JBHI.2018.2876916	2019	Pediatric Orthopaedics	Deep Learning (DL)	Diagnosis & Detection
4517	10.1007/s10278-018-0167-7	2019	Trauma	Machine Learning (ML); Deep Learning (DL)	Prognosis & Prediction
4518	10.1016/j.cmpb.2019.05.003	2019	Spine Surgery	Machine Learning (ML); Deep Learning (DL)	Segmentation & Measurement
4519	10.1016/j.compmedimag.2019.06.002	2019	Arthroplasty	Deep Learning (DL)	Prognosis & Prediction
4520	10.1097/BOR.0000000000000607	2019	Trauma	Machine Learning (ML); Deep Learning (DL)	Prognosis & Prediction
4521	10.1007/s00247-019-04408-2	2019	Pediatric Orthopaedics	Machine Learning (ML); Deep Learning (DL)	Diagnosis & Detection
4522	10.1016/j.jocd.2018.08.006	2019	Unclassified	Deep Learning (DL)	Diagnosis & Detection
4523	10.1007/s10278-018-0098-3	2019	Arthroplasty	Machine Learning (ML); Deep Learning (DL)	Prognosis & Prediction
4524	10.1016/j.compbiomed.2019.03.015	2019	Pediatric Orthopaedics	Deep Learning (DL)	Prognosis & Prediction
4525	10.1002/mp.13468	2019	Spine Surgery	Deep Learning (DL)	Prognosis & Prediction
4526	10.1007/s00330-018-5846-8	2019	Trauma; Spine Surgery	Natural Language Processing (NLP); Machine Learning (ML); Deep Learning (DL)	Prognosis & Prediction
4527	10.1088/1361-6560/ab0606	2019	Arthroplasty	Deep Learning (DL)	Diagnosis & Detection
4528	10.1186/s12911-019-0780-5	2019	Trauma	Natural Language Processing (NLP)	Prognosis & Prediction
4529	10.3390/ijerph16071281	2019	Spine Surgery	Deep Learning (DL)	Prognosis & Prediction
4530	10.1016/j.cmpb.2019.02.006	2019	Trauma	Deep Learning (DL)	Diagnosis & Detection

4531	10.1016/j.compmedimag.2019.01.007	2019	Unclassified	Machine Learning (ML)	Prognosis & Prediction
4532	10.1016/j.ejrad.2019.01.028	2019	Spine Surgery	Deep Learning (DL)	Segmentation & Measurement
4533	10.1002/jmri.26280	2019	Trauma	Machine Learning (ML)	Prognosis & Prediction
4534	10.1148/ryai.2019180037	2019	Spine Surgery	Machine Learning (ML); Robotics	Unclassified
4535	10.1148/radiol.2018180736	2019	Pediatric Orthopaedics	Machine Learning (ML); Deep Learning (DL)	Diagnosis & Detection
4536	10.1016/j.bone.2018.11.008	2019	Unclassified	Machine Learning (ML); Deep Learning (DL)	Prognosis & Prediction
4537	10.1016/j.media.2018.11.009	2019	Sports Medicine	Deep Learning (DL)	Diagnosis & Detection
4538	10.1111/1754-9485.12828	2019	Trauma	Machine Learning (ML); Deep Learning (DL)	Diagnosis & Detection
4539	10.1002/jmri.26246	2019	Sports Medicine; Trauma; Arthroplasty	Deep Learning (DL)	Prognosis & Prediction
4540	10.1148/ryai.2019180015	2019	Pediatric Orthopaedics; Trauma; Arthroplasty	Deep Learning (DL)	Prognosis & Prediction
4541	10.1093/rap/rkz047	2019	Arthroplasty	Machine Learning (ML); Deep Learning (DL)	Diagnosis & Detection
4542	10.1148/ryai.2019180001	2019	Trauma	Deep Learning (DL)	Diagnosis & Detection
4543	10.2174/1573405615666190724101600	2019	Unclassified	Deep Learning (DL)	Segmentation & Measurement
4544	10.1148/radiol.2019190201	2019	Trauma; Spine Surgery	Deep Learning (DL)	Prognosis & Prediction
4545	10.1148/radiol.2019191586	2019	Arthroplasty	Robotics	Prognosis & Prediction
4546	10.3174/ajnr.A6174	2019	Spine Surgery	Machine Learning (ML)	Diagnosis & Detection
4547	10.2214/AJR.19.21117	2019	Trauma	Machine Learning (ML); Deep Learning (DL)	Prognosis & Prediction
4548	10.1259/bjr.20190286	2019	Spine Surgery	Natural Language Processing (NLP); Machine Learning (ML)	Prognosis & Prediction
4549	10.7150/thno.33841	2019	Pediatric Orthopaedics	Machine Learning (ML)	Prognosis & Prediction
4550	10.1055/s-0039-1684024	2019	Unclassified	Machine Learning (ML); Deep Learning (DL)	Diagnosis & Detection
4551	10.1038/s41584-018-0130-5	2019	Unclassified	Machine Learning (ML); Deep Learning (DL)	Prognosis & Prediction
4552	10.1111/1556-4029.13858	2019	Spine Surgery	Machine Learning (ML)	Prognosis & Prediction
4553	10.12968/hmed.2018.79.12.676	2018	Unclassified	Unclassified	Diagnosis & Detection

4554	10.2214/AJR.18.19974	2018	Pediatric Orthopaedics; Trauma; Arthroplasty	Machine Learning (ML); Deep Learning (DL)	Diagnosis & Detection
4555	10.1016/j.compbiomed.2018.10.012	2018	Unclassified	Machine Learning (ML); Deep Learning (DL)	Segmentation & Measurement
4556	10.1002/mrm.27229	2018	Sports Medicine; Arthroplasty	Natural Language Processing (NLP); Deep Learning (DL)	Segmentation & Measurement
4557	10.1016/j.acra.2018.03.008	2018	Spine Surgery	Natural Language Processing (NLP); Machine Learning (ML)	Prognosis & Prediction
4558	10.1002/mrm.27178	2018	Sports Medicine	Deep Learning (DL)	Diagnosis & Detection
4559	10.1007/s10278-018-0066-y	2018	Unclassified	Natural Language Processing (NLP); Machine Learning (ML); Deep Learning (DL)	Diagnosis & Detection
4560	10.1016/j.jofri.2018.08.001	2018	Trauma	Deep Learning (DL)	Prognosis & Prediction
4561	10.1007/s10278-018-0053-3	2018	Trauma; Pediatric Orthopaedics	Machine Learning (ML); Deep Learning (DL)	Prognosis & Prediction
4562	10.1080/21681163.2016.1261370	2018	Spine Surgery	Machine Learning (ML); Deep Learning (DL)	Prognosis & Prediction
4563	10.1109/TNB.2018.2840082	2018	Sports Medicine; Arthroplasty	Machine Learning (ML); Deep Learning (DL)	Prognosis & Prediction
4564	10.1016/j.compbiomed.2018.05.011	2018	Trauma; Spine Surgery	Deep Learning (DL)	Diagnosis & Detection
4565	10.1016/j.compmedimag.2018.04.009	2018	Arthroplasty	Deep Learning (DL)	Diagnosis & Detection
4566	10.1016/j.jbiomech.2018.04.021	2018	Sports Medicine; Arthroplasty	Machine Learning (ML); Deep Learning (DL)	Prognosis & Prediction
4567	10.3389/fbioe.2018.00075	2018	Unclassified	Machine Learning (ML); Deep Learning (DL)	Prognosis & Prediction
4568	10.1016/j.transci.2018.05.004	2018	Pediatric Orthopaedics; Arthroplasty	Natural Language Processing (NLP)	Prognosis & Prediction
4569	10.1016/j.crad.2017.11.015	2018	Trauma	Machine Learning (ML); Deep Learning (DL)	Prognosis & Prediction
4570	10.1002/mrm.26841	2018	Sports Medicine; Arthroplasty	Deep Learning (DL)	Segmentation & Measurement
4571	10.1007/s10278-017-0021-3	2018	Unclassified	Natural Language Processing (NLP); Machine Learning (ML); Deep Learning (DL)	Diagnosis & Detection
4572	10.1111/1556-4029.13582	2018	Pediatric Orthopaedics	Machine Learning (ML); Deep Learning (DL)	Prognosis & Prediction

4573	10.1155/2018/2391925	2018	Unclassified	Machine Learning (ML); Deep Learning (DL)	Diagnosis & Detection
4574	10.4258/hir.2018.24.1.86	2018	Unclassified	Deep Learning (DL)	Prognosis & Prediction
4575	10.1016/j.nicl.2017.10.015	2018	Arthroplasty	Machine Learning (ML); Deep Learning (DL)	Diagnosis & Detection
4576	10.2214/AJR.17.18224	2017	Pediatric Orthopaedics	Deep Learning (DL)	Unclassified
4577	10.1007/s10278-017-9955-8	2017	Pediatric Orthopaedics	Machine Learning (ML); Deep Learning (DL)	Diagnosis & Detection
4578	10.1016/j.compbiomed.2017.03.017	2017	Arthroplasty	Natural Language Processing (NLP); Machine Learning (ML); Deep Learning (DL)	Diagnosis & Detection
4579	10.1007/s00223-017-0238-7	2017	Trauma	Machine Learning (ML)	Prognosis & Prediction
4580	10.1016/j.media.2016.10.010	2017	Pediatric Orthopaedics	Machine Learning (ML); Deep Learning (DL)	Unclassified
4581	N/A	2017	Arthroplasty	Natural Language Processing (NLP); Deep Learning (DL)	Prognosis & Prediction
4582	10.1259/dmfr.20160107	2017	Trauma	Natural Language Processing (NLP); Deep Learning (DL)	Diagnosis & Detection
4583	10.1007/s11926-017-0629-9	2017	Unclassified	Machine Learning (ML)	Prognosis & Prediction
4584	10.1016/j.media.2016.08.004	2017	Spine Surgery	Deep Learning (DL)	Prognosis & Prediction
4585	10.1016/j.cmpb.2016.07.005	2016	Trauma	Deep Learning (DL)	Unclassified
4586	10.1016/j.jbmt.2016.02.004	2016	Pediatric Orthopaedics	Unclassified	Diagnosis & Detection
4587	10.1016/j.jbiomech.2016.04.010	2016	Trauma	Natural Language Processing (NLP); Machine Learning (ML)	Prognosis & Prediction
4588	10.1007/s00774-015-0668-0	2016	Unclassified	Deep Learning (DL)	Segmentation & Measurement
4589	10.18632/oncotarget.7278	2016	Unclassified	Machine Learning (ML)	Prognosis & Prediction
4590	10.1016/j.jbiomech.2015.11.019	2016	Sports Medicine; Arthroplasty	Deep Learning (DL)	Prognosis & Prediction
4591	10.1186/s12911-016-0306-3	2016	Unclassified	Natural Language Processing (NLP); Machine Learning (ML)	Prognosis & Prediction
4592	10.5114/reum.2016.63664	2016	Arthroplasty	Machine Learning (ML)	Diagnosis & Detection
4593	10.4338/ACI-2016-08-RA-0129	2016	Trauma; Pediatric Orthopaedics	Natural Language Processing (NLP); Machine Learning (ML)	Diagnosis & Detection
